# Fast identification of differential distributions in single-cell RNA-sequencing data with waddR

**DOI:** 10.1093/bioinformatics/btab226

**Published:** 2021-04-01

**Authors:** Roman Schefzik, Julian Flesch, Angela Goncalves

**Affiliations:** German Cancer Research Center (DKFZ), Somatic Evolution and Early Detection Group, 69120 Heidelberg, Germany; German Cancer Research Center (DKFZ), Somatic Evolution and Early Detection Group, 69120 Heidelberg, Germany; German Cancer Research Center (DKFZ), Somatic Evolution and Early Detection Group, 69120 Heidelberg, Germany

## Abstract

**Motivation:**

Single-cell gene expression distributions measured by single-cell RNA-sequencing (scRNA-seq) often display complex differences between samples. These differences are biologically meaningful but cannot be identified using standard methods for differential expression.

**Results:**

Here, we derive and implement a flexible and fast differential distribution testing procedure based on the 2-Wasserstein distance. Our method is able to detect any type of difference in distribution between conditions. To interpret distributional differences, we decompose the 2-Wasserstein distance into terms that capture the relative contribution of changes in mean, variance and shape to the overall difference. Finally, we derive mathematical generalizations that allow our method to be used in a broad range of disciplines other than scRNA-seq or bioinformatics.

**Availability and implementation:**

Our methods are implemented in the R/Bioconductor package waddR, which is freely available at https://github.com/goncalves-lab/waddR, along with documentation and examples.

**Supplementary information:**

Supplementary data are available at *Bioinformatics* online.

## 1 Introduction

**Fig. 1.**
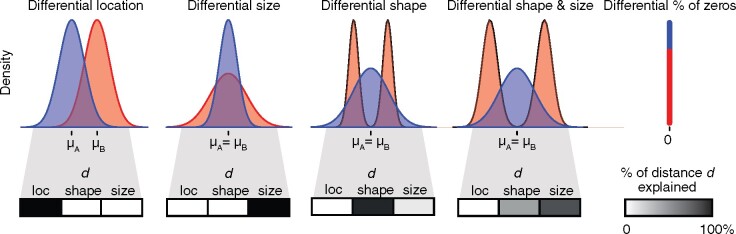
Probability density functions illustrating different types of DDs. The corresponding decomposition of the 2-Wasserstein distance *d* between distributions into location, shape and size terms is shown below each example

A typical task in genomic analyses is to identify genes whose expression varies across biological conditions. When comparing the single-cell expression distribution of a gene between two samples measured with single-cell RNA-sequencing (scRNA-seq), complex differences can often be observed between two distributions (Bacher and Kendziorski, 2016), including: shifts in the mean of the distributions, differences in the variance, differences in the shape of the distribution (e.g. from unimodal to multimodal), changes in the abundance of zeros or a combination of these (Fig. 1). Such differences may be attributable to biologically meaningful changes, including for instance the induction of novel subpopulations or transient cell-states upon treatment of an otherwise homogeneous population of cells (Kolodziejczyk *et al.*, 2015), changes in the proportion of zeros from transcriptional bursting (Marinov *et al.*, 2014) or an increase in transcriptional variance during ageing (Martinez-Jimenez *et al.*, 2017).

Despite the potential of scRNA-seq to reveal such complex patterns, the detection of these differences is beyond the scope of traditional methods for identifying differential expression. In general, statistical tools developed for detecting differential expression, such as DESeq2 (Love *et al.*, 2014), edgeR (Robinson *et al.*, 2010) and most scRNA-seq-specific methods, assume that each gene has a latent unimodal level of expression within a biological condition, and have been designed to detect shifts in the mean of these distributions across conditions only. More flexible approaches are required to better characterize differences between single cells across conditions. Some methods such as BASiCS (Vallejos *et al.*, 2016) have gone beyond testing for differences in means and additionally test for differences in variance.

Here, we present a flexible method for detecting differential distributions (DDs) based on the 2-Wasserstein distance (Panaretos and Zemel, 2019; Rüschendorf, 2001), that is sensitive to testing for any type of difference between two conditions. Our method can be used to test for differences between two expression distributions of a gene, e.g. between two cell-types within a sample (variant A). Alternatively, it can be used to test for differences between two conditions, e.g. when comparing the same cell-type between treatment conditions with multiple replicates (variant B). By simulation, we validate our decomposition approach and demonstrate substantial speed improvements over alternative methods for variant A testing. To demonstrate our novel approach in variant B, we apply our methods in a case study of detecting DDs between natural killer (NK) cells resident in human decidua versus blood (Vento-Tormo *et al.*, 2018), and highlight instances of biologically meaningful but hitherto unseen expression complexity. Our method is implemented in the R/Bioconductor package waddR, and generalizations are provided for the application to continuous data.

## 2 Materials and methods

### 2.1 The 2-Wasserstein distance and its decomposition

For two continuous cumulative distribution functions (CDFs) *F_A_* (with mean *μ_A_* and standard deviation *σ_A_*) and *F_B_* (with mean *μ_B_* and standard deviation *σ_B_*) that model the distributions for conditions *A* and *B*, respectively, the squared 2-Wasserstein distance *d*, referred to as the ‘2-Wasserstein distance’ for convenience throughout the text, is given by (Panaretos and Zemel, 2019; Rüschendorf, 2001)

$$\begin{array}{c} d:=d(F_{A},F_{B})=\int_{0}^{1}|F_{A}^{-1}(u)-F_{B}^{-1}(u){|}^{2}du\\ =\underset{\mathrm{location}}{\underset{︸}{{\left(\mu_{A}-\mu_{B}\right)}^{2}}}+\underset{\mathrm{variability}}{\underset{︸}{\underset{\mathrm{size}}{\underset{︸}{{\left(\sigma_{A}-\sigma_{B}\right)}^{2}}}+\underset{\mathrm{shape}}{\underset{︸}{2\sigma_{A}\sigma_{B}(1-\rho_{A,B})}}}}, \end{array}$$

where $\rho_{A,B}\in [0,1]$ is the Pearson correlation coefficient of the points in the quantile–quantile (Q–Q) plot of *F_A_* and *F_B_*. In this useful decomposition of *d*, differences in location and size are quantified by the squared differences of the means and standard deviations, respectively, while differences in shape may refer to differences with respect to modalities or skewness and are mainly covered by the correlation coefficient $\rho_{A,B}$ (Irpino and Verde, 2015). Typically, the distributions *F_A_* and *F_B_* are not explicitly given and information is only available in the form of samples. Here we use the corresponding empirical CDFs ${\hat{F}}_{A}$ and ${\hat{F}}_{B}$ to approximate the distributions (Supplementary Material S1).

### 2.2 Semi-parametric permutation test with generalized Pareto distribution approximation (waddR SP)

To test whether two distributions (CDFs) *F_A_* and *F_B_* represented by two samples are significantly differentially distributed, we specifically test the null hypothesis $H_{0}:F_{A}=F_{B}$ against the alternative $H_{1}:F_{A}\neq F_{B}$ using the 2-Wasserstein distance. In a semi-parametric (SP) testing procedure we combine a classical permutation test with a generalized Pareto distribution (GPD) approximation to derive a *P*-value (similar but not identical to Matsui *et al.*, 2016). We employ the sample-based 2-Wasserstein distance d≥0 as a test statistic here and test $H_{0}:d=0$ against $H_{1}:d>0$ to identify differences in distributions. As it is usually computationally infeasible to compute all permutations, we use a subset of size $N_{\mathrm{sub}}$ of all the permutations. Moreover, to avoid *P*-values of exactly zero we insert a pseudo-count. Consequently, the *P*-value has a lower bound of $1/(N_{\mathrm{sub}}+1)$. However, in high-dimensional applications, such as in genomics, a very high resolution for the *P*-values is often required, because the threshold for statistical significance may be close to zero due to multiplicity correction (Benjamini and Hochberg, 1995). To address this, we use the approach in Knijnenburg *et al.* (2009) and model the tail of the distribution of the test statistic values obtained by permutations using a GPD (Supplementary Material S1).

### 2.3 Different testing scenarios in scRNA-seq experiments

We distinguish two different scenarios in the context of DD testing for discrete scRNA-seq data: in the first setting A, gene expression data are only available from one replicate per condition (e.g. when comparing two cell types within a sample), while in the second scenario B, there are expression data from multiple replicates per condition (e.g. when comparing the same cell-type under different experimental conditions). As the starting point for our waddR approaches, we assume a pre-processed (normalized) scRNA-seq data matrix consisting of expression values for multiple genes over multiple cells.

#### 2.3.1 Variant A: one replicate per condition

In the setting in which expression data is only available for one replicate per condition, the aim is to test whether two expression distributions of a gene are significantly different. To incorporate the special role of zero expression in scRNA-seq data, i.e. the point mass at zero, we divide the procedure into two parts and propose the following two-stage approach: (i) test for differential proportions of zero expression (DPZ) between the two conditions using a Bayesian logistic regression model, taking account of the cellular detection rate in order to correct for differences in total counts per cell (Finak *et al.*, 2015; Korthauer *et al.*, 2016); and (ii) separately test for differences in non-zero expression by applying the semi-parametric waddR SP testing procedure to the non-zero expression values only. This approach yields two *P*-values, P.zero and $P.\mathrm{nonzero}=P_{\mathrm{SP}}$. The *P*-values P.zero and P.nonzero can be combined into an overall *P*-value P.comb for DD using the classical Fisher method (Supplementary Material S1, Supplementary Fig. S2).

#### 2.3.2 Variant B: testing between two conditions with multiple replicates

In our second setting, expression data is available from multiple replicates per condition. Here, data may be unpaired or paired. In the unpaired setting, data come from two separate sets of independent and identically distributed samples, e.g. when data for condition *A* stem from multiple instances that are different from those in condition *B*. In the paired setting, data for condition *A* arise from the same instances as for condition *B*. A typical example of a paired setting would be data stemming from the same individual tested under two different conditions *A* and *B*.

1. For DPZ testing for the paired setting we employ the Cochran-Mantel-Haenszel (CMH) test, a multi-dimensional generalization of the classical Fisher’s exact proportion test, yielding a respective *P*-value P.zero. For DPZ testing for the unpaired setting, with possibly different numbers of replicates per condition, log-linear models may be used.
2. For non-zero expression testing we employ the following procedure based on 2-Wasserstein distances, which can in principle be applied to both paired and unpaired settings. Based on *R* replicates and two conditions *A* and *B*, we calculate the 2-Wasserstein distances $D_{\mathrm{BC}}$ for all comparisons between the conditions and the 2-Wasserstein distances $D_{\mathrm{WC}}$ for all comparisons within the conditions. Finally, we employ the Wilcoxon rank sum test to test for mean differences between the distance values in $D_{\mathrm{BC}}$ and $D_{\mathrm{WC}}$, yielding a corresponding *P*-value P.nonzero. An overall decomposition of the distance into location, size and shape components can be readily calculated as an average of the $D_{\mathrm{BC}}$ decomposition fractions.

As for variant A, the *P*-values *P*.zero and *P*.non-zero can be combined into an overall *P*-value *P*.comb for DD using the classical Fisher method.

### 2.4 Generalization to applications beyond scRNA-seq

waddR is potentially useful in a diverse range of practical applications beyond scRNA-seq, i.e. whenever the aim is to test whether there are significant differences between two distributions. Examples include methylation array experiments (Matsui *et al.*, 2016) or fluorescence-activated cell sorting (FACS) data, but may also go beyond bioinformatics applications, e.g. ensemble forecasts in modern weather prediction (Buizza, 2018). For applications which can be assumed to come from continuous distributions, we have implemented the waddR ASY variant that makes use of an asymptotic result for the null distribution of the 2-Wasserstein distance (Supplementary Material S1, Supplementary Fig. S1). This variant substitutes the computationally expensive permutation procedure SP, that is used in the context of scRNA-seq data, for continuous data.

## 3 Results

### 3.1 Simulation experiments to validate waddR SP and waddR ASY testing procedures

To validate the decomposition of the 2-Wasserstein distance and the SP and ASY testing procedures, we demonstrate that waddR shows reasonable performances regarding detection power and type I error and is able to identify the causes of the differences between two distributions correctly in a set of simulations studies based on normal (Supplementary Material S2, Supplementary Figs S3–S9, Supplementary Tables S2 and S3) and Gamma distribution models (Supplementary Material S3, Supplementary Figs S10–S12, Supplementary Tables S4 and S5).

### 3.2 Simulation experiments based on scRNA-seq data for waddR variant a

Here we perform investigations based on simulations adapted to the context of scRNA-seq data and in comparison to two alternative methods for testing one replicate per condition (variant A).

#### 3.2.1 Methods for comparison

A number of methods specialized for scRNA-seq differential expression testing between conditions with multiple replicates have been developed in recent years [reviewed in Miao and Zhang (2016), Jaakkola *et al.* (2017), Dal Molin *et al.* (2017), Soneson and Robinson (2018), Wang et al. (2019) and Supplementary Table S6]. Of these, the majority tackle the problem of testing for differences in means while addressing the statistical challenges posed by high levels of technical noise and intrinsic biological variability in single-cell experiments. Some methods such as BASiCS (Vallejos *et al.*, 2016) and DEsingle (Miao *et al.*, 2018), have gone beyond testing for differences in means and additionally test for differences in variance (BASiCS) or differences in the proportion of zeros (DEsingle). Our method tests instead for any change in the full distribution, thus capturing the highly complex expression patterns frequently observed in scRNA-seq datasets (Korthauer *et al.*, 2016). To our knowledge, no other method is readily applicable to testing for DD shape between conditions with replicates (variant B).

For variant A, we compare our approach to two previously published methods, scDD (Korthauer *et al.*, 2016) and SigEMD (Wang and Nabavi, 2018), which have been developed to explicitly test for differences in shape between two distributions. Given that both scDD and SigEMD use the same approach to detect DPZs as waddR, we limit our comparisons to the non-zero part.

scDD (Korthauer *et al.*, 2016) models non-zero, multi-modal expression using a Dirichlet process mixture of normals. It provides a characterization of the type of the DD by explicitly modelling the following patterns: traditional mean-based differential expression (DE), differential modality (DM), differential mode weights (DP) and a combination of the previous two (DB). To calculate a *P*-value, scDD originally uses a permutation test based on Bayes factor scores. As this procedure is computationally expensive, a second, computationally faster, option is provided which employs a KS test instead. However, the KS test as used in scDD is originally designed for continuous data, and is not the best choice for the scRNA-seq setting in which we have expression data derived from discrete read counts.

waddR is conceptually simpler than scDD and requires fewer (virtually no) assumptions or modelling, thus requiring less computation time. While for non-zero differential expression, scDD provides a single category of DD as an output (categorization), waddR provides information on differences via the decomposition of the 2-Wasserstein distance test statistic. For example, it is possible to combine results from different replicates with our method, by averaging the corresponding fractions of location, size and shape components for each replicate as discussed before. This is not directly possible with the categorical output of scDD.

The second reference approach, SigEMD (Wang and Nabavi, 2018), employs the Earth Mover’s distance (EMD) for DD analysis of expression values distributions. Compared to SigEMD, waddR uses the 2-Wasserstein distance including its decomposition and not the EMD, which is in fact the 1-Wasserstein distance and for which there is no similar decomposition readily available. The calculation of the Wasserstein distance in SigEMD is based on solving an optimization problem, following an alternative, original, definition of the Wasserstein distance, which is computationally intensive. In contrast, waddR makes use of an equivalent quantile representation of the Wasserstein distance, which can be conveniently interpreted and directly computed, while allowing for a computationally faster implementation.

#### 3.2.2 Setting of the simulation study

We created simulated scRNA-seq data based on the simulation procedures in Korthauer *et al.* (2016), using the example dataset, default hyperparameter choices and simulation function from the scDD package. In particular, we generate G:=1000 genes across two conditions with *C* cells each, where we here employ the three different numbers of cells (i.e. sample sizes) C∈{50,100,500}.

In each scenario, 100 of the *G *=* *1000 genes are generated as differentially distributed and equally divided into the following four different categories of DDs (i.e. 25 genes per category):

- traditional differential expression (DE): two unimodal distributions with different means,
- differential proportions of cells within each component (DP): two bimodal distributions with the same modes, but with different proportions of cells of each mode,
- differential modality (DM): one unimodal distribution and one bimodal distribution with one mode being identical to that of the univariate distribution,
- both differential modality and different component means (DB): one unimodal distribution and one bimodal distribution with no common modes.

We generated the remaining 900 genes as non-DD genes by creating one half (i.e. 450 genes) from the same unimodal distribution [corresponds to the equivalent expression (EE) category in scDD] and the other half (i.e. 450 genes) from the same negative binomial model with two components [corresponds to the equivalent proportion (EP) category in scDD]. Finally, we consider three different degrees of DDs (weak, medium, strong) by varying the fold changes between the modes for the DP, DM and DB settings from 2 over 4 to 6, while fixing standard deviations of the fold changes to 2, see Korthauer *et al.* (2016) and the scDD package manual for details. For each method, 1000 permutations are used to obtain the respective testing results.

#### 3.2.3 Simulation results

**Fig. 2.**
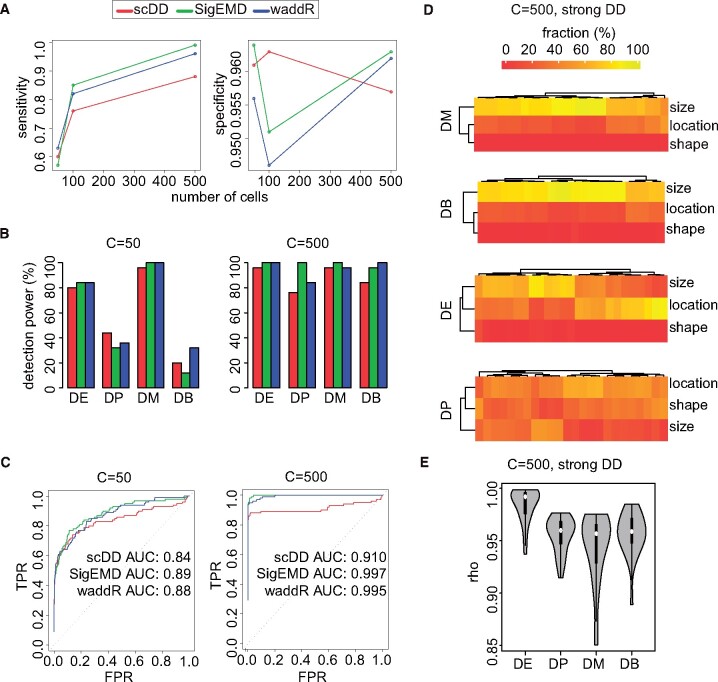
(**A–C**) Performance metrics when simulating a weak degree of DD and with *P*-value threshold of 0.05: (A) Sensitivity and specificity depending on the number of cells. (B) Detection powers for the different DD categories according to Korthauer *et al.* (2016), depending on number of cells *C*. (C) ROC curves with AUC values, depending on number of cells *C*. (**D**) Decomposition of the 2-Wasserstein distance for *C *=* *500 and a strong degree of DD for the different DD categories, shown for those genes that have been detected as DD. Note that there is no exact one-to-one correspondence and interpretation between the scDD categories and the terms of the decomposition of the 2-Wasserstein distance. (**E**) Violin plots of the correlation coefficient *ρ* in the decomposition of the 2-Wasserstein distance for *C *=* *500 and a strong degree of DD for the different DD categories, based on those genes that have been detected as DD

We evaluate the performance of waddR and the reference methods by considering sensitivity, specificity, precision, accuracy and the receiver operating characteristic (ROC) curves with corresponding area under the curve (AUC) values (Fig. 2A, C and Supplementary Figs S13 and S14). Overall, waddR shows good performances that typically get better with increasing number of cells and more pronounced degrees of DD (Supplementary Figs S13 and S14). In particular, waddR outperforms scDD or performs similarly well in the majority of the considered cases. In some cases, SigEMD performs a little better than waddR and scDD. Overall, waddR yields good detection powers (sensitivity) with respect to the four different scDD categories for simulated DD genes, especially when there is a medium or strong degree of DD and a reasonably large number of cells (Fig. 2B and Supplementary Fig. S15). There are only certain weaknesses in detecting DDs of the type DP and DB for small numbers of cells and when the degree of DD is not very pronounced. However, this also holds for the other methods and confirms results obtained in previous simulation studies (Korthauer *et al.*, 2016; Wang and Nabavi, 2018).

The behaviour of the decomposition with respect to the different DD categories DE, DP, DM and DB is shown in Figure 2D. Similar tendencies can also be found in the other settings (Supplementary Fig. S16). For the DE category, both differences in location and size make up the overall difference. In contrast, the shape component is negligible. This is in agreement with the definition and construction of the DE category in scDD, where only shifts in location and size are expected. For the DP category, each of the location, size and shape components may contribute to the overall distance, with the shape component typically taking on a more pronounced role than size, when compared to the other categories. The DM and DB categories show a similar behaviour regarding the decomposition of the 2-Wasserstein distance. In both cases, the size component is very pronounced, while the location and in particular the shape components are rather small. However, when looking at the correlation coefficients *ρ*, a difference with respect to shape is also apparent. The very strong influence of the size component, i.e. the large difference between the standard deviations, causes the shape term to become negligible in the decomposition, even though the values of the correlation coefficient *ρ* suggest that there are also differences with respect to shape (Fig. 2E and Supplementary Fig. S16).

The main advantage of waddR compared to the reference methods scDD and SigEMD is the reduced computational running time (Tables 1 and 2). In general, computation time depends on the number of genes, the number of cells, the degree of the DD and the number of the permutations employed in the analysis method. Regarding our simulation studies here, we have G:=1000 genes and 1000 permutations. All methods were run on the same computer, using the default settings of the reference methods. We report average approximate running times over the three different degrees of DD, for each number of cells separately (Table 1). Interestingly, the running times were typically consistent across the different degrees of DDs for scDD and waddR, whereas they were considerably different for SigEMD, in that the stronger the degree of DD the longer the computation takes. waddR is by far the fastest of all considered permutation-based approaches, due to its simpler underlying concept and implementation, but also as it employs a fast and efficient C++ implementation of the permutation procedure and the calculation of the 2-Wasserstein distance(s). The benefit of waddR becomes more obvious when looking at the running times when using 10000 permutations, which is typically a more reasonable number of permutations to obtain reliable results. Table 2 shows a comparison of the running times based on using 1000 or 10000 permutations, respectively, for the computationally cheapest setting of *C *=* *50 with a weak degree of DD. While an increase in the number of employed permutations increases the running time of scDD drastically (by several days) and that of SigEMD considerably (by approximately a day), the increase for waddR is very moderately (by several minutes). Further, running times are also expected to increase with the number of genes. As typical scRNA-seq datasets comprise much more than 1000 genes as considered in our simulation studies, computational running times may get even more important, with waddR providing the fastest of all considered implementations.

**Table 1.** Approximate running times averaged across all degrees of DD within the simulation studies, depending on the number of cells *C*

	*C *=* *50	*C *=* *100	*C *=* *500
scDD	≈ 10 h	≈ 18 h	≈ 38 h
SigEMD	≈ 41 h	≈ 52 h	≈ 82 h
waddR	≈ 1 min	≈ 1 min	≈ 1 min

**Table 2.** Approximate running times for *C *=* *50 and a weak degree of DD in the simulation study when employing 1000 and 10000 permutations, respectively

	1000 permutations	10000 permutations
scDD	≈ 10 h	≈ 3 days
SigEMD	≈ 5 h	≈ 28 h
waddR	≈ 1 min	≈ 8 min

In summary, in our simulation studies of variant A, waddR is equivalent or outperforms the reference methods scDD and SigEMD with respect to common performance criteria. The most obvious benefit of waddR is a reduced computation time, even though, as scDD and SigEMD, it is based on a permutation testing procedure. waddR also benefits from its conceptual simplicity, as it comes without sophisticated modelling (scDD) or the need to solve optimization problems (SigEMD) that may be computationally intense.

### 3.3 Benchmarking waddR variant B using real scRNA-seq data

To validate the performance of waddR variant B for multiple replicates, we apply our method to the real Fluidigm C1 platform-based scRNA-seq dataset of Tung *et al.* (2017). For this dataset, there exists a matching bulk RNA-seq dataset, from which we derive a list of reference differentially expressed genes that we employ as the ground truth to compare against. Furthermore, we investigate the impact of the number of replicates and the use of some of the most widely used normalization methods on performance (Supplementary Material S5).

In this benchmarking study, waddR shows adequate ROC curves (Supplementary Fig. S17) and FDR curves (Supplementary Fig. S18), with a reasonable balance between sensitivity and specificity (Supplementary Table S7). Moreover, waddR exhibits a good control of type I errors (Supplementary Figs S19 and S20). The results are consistent across different considered normalization methods (Supplementary Figs S17–S20, Supplementary Table S7). Moreover, the performance of waddR variant B meaningfully increases with the number of replicates. This is to be expected, as the powers of the involved testing procedures, in particular the Wilcoxon rank sum test, rise with increasing number of replicates (Supplementary Figs S21–S24, Supplementary Table S8). Along with the benefit of drastically reduced computation times, waddR variant B furthermore outperforms or is competitive to the scDD and SigEMD reference approaches, which are not explicitly designed to handle replicates, with respect to all the evaluation criteria mentioned before (Supplementary Figs S25–S29, Supplementary Table S9).

### 3.4 Case study: detecting DDs between tissue-resident and blood NK cells

#### 3.4.1 Details of the case study

In order to test waddR variant B in a real-data case study, we apply it to a subset of the scRNA-seq dataset in Vento-Tormo *et al.* (2018) for first-trimester placentas with matched maternal blood and decidual cells. Specifically, we consider the subset of those cells that have been classified as natural killer (NK) cells in Vento-Tormo *et al.* (2018), and we use the 10× Genomics sequencing platform-based scRNA-seq data of four replicates for which both measurements in decidua and blood are available. The comparison we look at is NK cells in decidua (condition *A*) versus NK cells in blood (condition *B*). After normalizing and filtering out those genes that show zero expression in all cells across both conditions and across all replicates, this dataset comprises log-normalized expression distributions for 21138 genes (Table 3).

**Table 3.** Setting of the real-data case study

	Decidua	Blood
Donor F19	3249 NK cells	569 NK cells
Donor F20	282 NK cells	107 NK cells
Donor F25	2245 NK cells	360 NK cells
Donor F27	4016 NK cells	325 NK cells

Our goal is to compare the analysis results from waddR to those obtained when applying the edgeR approach (Robinson *et al.*, 2010), which originally has been designed for differential expression analysis for bulk RNA-seq measurements. To mimic bulk values from the single-cell dataset in Vento-Tormo *et al.* (2018), we compute for each gene and for each replicate separately the sum of the expression values (counts) over all respective cells and take this as a bulk input for edgeR. As we want to compare a bulk to a single-cell approach here, no filtering with respect to genes or cells is done here, since otherwise the mimicked bulk counts for edgeR could be distorted.

For the bulk edgeR method, we use the default normalization and the quasi-likelihood F test to check for differential expression as provided by the package, while explicitly accounting for the paired setting encountered.

For the single-cell-based analysis, the Seurat package (Butler *et al.*, 2018) is used for normalizing the data, using the default settings of log-normalization with scale factor 10^4^. Then, the waddR approach for multiple replicates (variant B) is applied to this pre-processed data. Our procedure gives three different *P*-values: a *P*-value P.zero referring to differences in proportions of zero expression (DPZ), a *P*-value P.nonzero referring to difference in non-zero expression (non-zero DD) and a combined *P*-value P.comb of overall DD. These *P*-values are then adjusted for multiple testing using the method of Benjamini and Hochberg (1995), yielding P.adj.zero, P.adj.nonzero and P.adj.comb, respectively. In contrast, the bulk edgeR method offers a single adjusted *P*-value P.adj as an overall result for differential expression.

#### 3.4.2 Genes detected by both methods

**Fig. 3.**
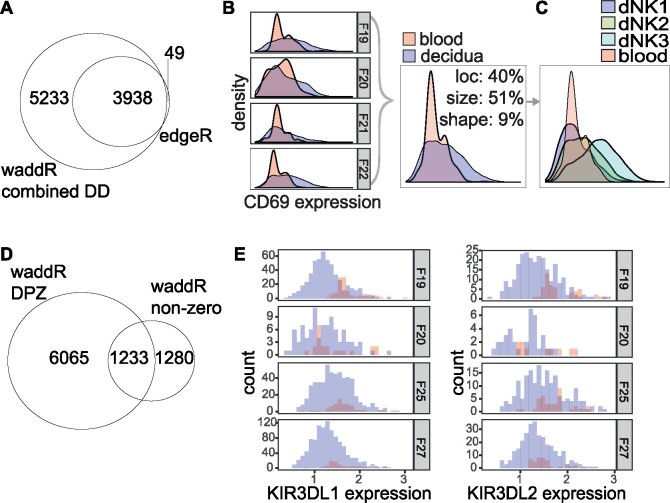
(**A**) Venn diagram of the number of genes detected as DD by waddR (BH-adjusted combined *P*-value ≤0.05) and by edgeR (BH-adjusted *P*-value ≤0.05). (**B**) Expression of CD69 over the NK cells of four donors. Red: NK cells isolated from peripheral blood. Blue: NK cells isolated from the decidua. For purely illustrative purposes, we fit a density to the samples. However, note that the density itself is not used for the actual testing procedures, nor for the calculation of the decomposition. (**C**) Distribution of expression across the cells from all donors, coloured by the newly identified NK subsets from Vento-Tormo *et al.* (2018). (**D**) Venn diagram of the number of genes detected as DD by waddR. (E) Histograms of expression of KIR receptors KIR3DL1 and KIR3DL2 for each of the four donors

Overall, edgeR detected 3987 differentially expressed genes [Benjamini-Hochberg (BH) adjusted *P*-value ≤0.05], 99% of which were also detected by waddR (BH adjusted combined *P*-value ≤0.05; Fig. 3A). Of the genes detected by both tools, 99% showed a significant difference in proportion of zeros (DPZ), while 19% showed a difference in the non-zero part. For such genes, that would otherwise be discovered using conventional tools, new insights can be gained by the decomposition into location, size and shape. For instance, the activation marker CD69 has been previously proposed to be an over-expressed marker of tissue-resident NK cells when compared to conventional, peripheral blood NK cells (Jabrane-Ferrat, 2019). The decomposition of the distribution of CD69 indicates that the Wasserstein distance is mostly explained by size (51%), followed by location (40%, Fig. 3B), suggesting that rather than a difference in expression, decidual NK cells are more variable with regard to CD69 expression (Fig. 3B). Indeed, when subsetting the decidual NK cells by the 3 sub-populations of dNK cells newly identified by Vento-Tormo *et al.* (2018), it becomes apparent that the expression of two sub-populations of decidual NK cells (dNK1 and dNK2) is similar to the expression of blood NK cells and that the increase in variance comes from a change in mean in the third population only (dNK3, Fig. 3C).

#### 3.4.3 Genes detected only by waddR

Compared to edgeR, waddR identified an additional 5233 differential genes, 63% of which showed a significant DPZ, while 30% showed a difference in the non-zero part (Fig. 3D). Among those genes uniquely identified by waddR, there is an enrichment of genes known to be differentially expressed between peripheral-blood NK cells and decidua resident NK cells, including KIR3DL1, KIR3DL2 and CD53. Interestingly, while both KIRs are expected to have higher expression in decidual NK cells compared to blood (Koopman *et al.*, 2003), we observe the opposite: when KIRs are expressed in blood NK cells they are expressed at a higher level than in decidua NK cells (Fig. 3E). The discrepancy between these results can be explained by the observation that proportionally fewer blood cells express KIRs compared to the decidual cells (5% average difference in proportion of zeros), a distinction that is beyond the reach of bulk expression assays.

## 4 Discussion

ScRNA-seq expression distributions typically show multi-modality, an abundance of zeros and increased variability (Bacher and Kendziorski, 2016). The detection of differences in such potentially biologically meaningful patterns is beyond the scope of traditional differential expression testing approaches. Here, we present methods derived from multiple, disconnected literature results for the 2-Wasserstein distance and put them into a unifying and overarching context particularly useful for single-cell applications. Our method can be used in two fundamentally different ways, to test for differences between two (and only two) distributions, treating cells as replicate observations (variant A); or to test for DDs between two conditions with replicate observations (variant B). The latter is the more important level of replication as different samples will necessarily be generated if the experiment is to be replicated (Amezquita *et al.*, 2020). Differential expression analyses that treat cells as replicates fail to properly model sample-to-sample variability (Lun and Marioni, 2017). Our method provides a framework for DD testing that can handle both variants and offers substantial speed improvements over alternative approaches for variant A testing, without compromising on detection performance.

In addition to applications in scRNA-seq data, the waddR framework is applicable to any other domain where two distributions are compared. For continuous data in particular, a newly derived asymptotic testing procedure allows for further speed improvements.

Future extensions of the waddR methods should deal with multiple conditions and multivariate data. In particular, log-linear models may provide an alternative to the Cochran-Mantel-Haenszel test for variant B DPZ testing. Such a frame would allow for the inclusion of covariates, for example by including the cellular detection rate as covariate in order to correct for differences in total counts per cell.

### 4.1 Usage notes for the waddR R package

We implemented our method as a package for the statistical environment R and distribute it within the Bioconductor project. As input for scRNA-seq analysis, it expects a table of pre-filtered and normalised count data. As filtering and normalisation are important steps that can have a profound impact in a scRNA-seq workflow (Cole *et al.*, 2019), these should be tailored to the specific question of interest before applying waddR. waddR is applicable to data from any scRNA-seq platform (demonstrated here for 10× Genomics and Fluidigm C1 Smart-Seq2) normalised using most common methods, such as those implemented in the Seurat (Butler *et al.*, 2018) or scran (Lun *et al.*, 2016) packages. Normalisation approaches that change the shape of the gene distributions (such as quantile normalization) and gene-wise scaling or standardizing should be avoided when using waddR.

## Supplementary Material

btab226_Supplementary_Data
