# Supplementary material for: Fast identification of differential distributions in single-cell RNA-sequencing data with waddR
Source: Bioinformatics. 2021 Apr 1;37(19):3204–11. doi: 10.1093/bioinformatics/btab226 (PMC8504634; doi:10.1093/bioinformatics/btab226)
Supplement: btab226_Supplementary_Data [file btab226_supplementary_data.zip › Supplement_Revision2/Fig7.pdf]

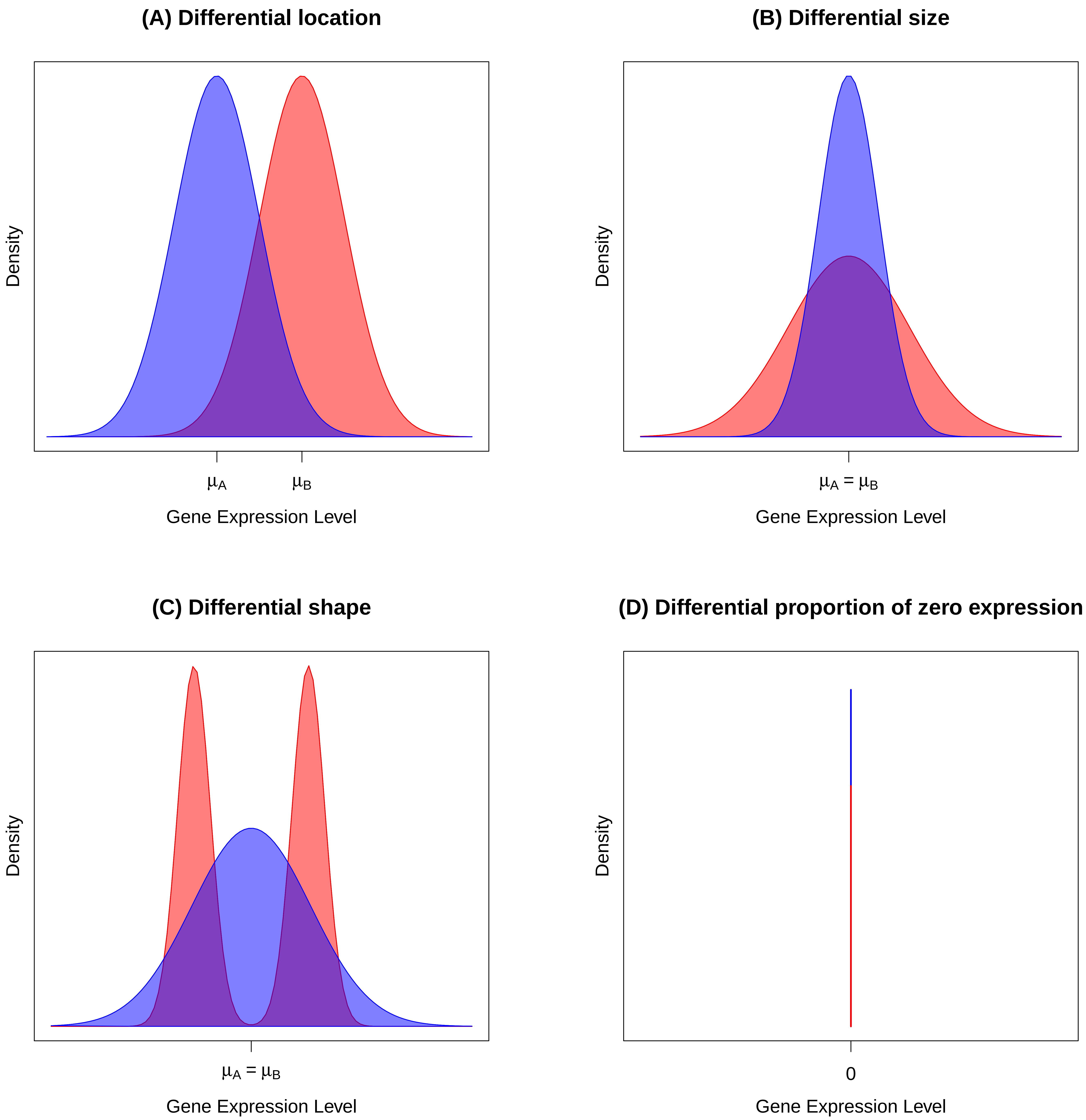

(A) Differential location
Density
μA
μB
Gene Expression Level
(B) Differential size
Density
μA = μB
Gene Expression Level
(C) Differential shape
Density
μA = μB
Gene Expression Level
(D) Differential proportion of zero expression
Density
0
Gene Expression Level
