# Supplementary material for: Fast identification of differential distributions in single-cell RNA-sequencing data with waddR
Source: Bioinformatics. 2021 Apr 1;37(19):3204–11. doi: 10.1093/bioinformatics/btab226 (PMC8504634; doi:10.1093/bioinformatics/btab226)
Supplement: btab226_Supplementary_Data [file btab226_supplementary_data.zip › Supplement_Revision2/Supplement.pdf]

Supplementary Material to

Fast identification of differential distributions in single-cell RNA-sequencing data with `waddR`

Roman Schefzik, Julian Flesch and Angela Goncalves

1 The 2-Wasserstein distance and its decomposition

To compare two cumulative distribution functions (CDFs) F_A (with mean μ_A and standard deviation σ_A) and F_B (with mean μ_B and standard deviation σ_B), we make use of the squared 2-Wasserstein distance d [16], conveniently referred to as the "2-Wasserstein distance" in what follows, which for continuous CDFs is given by

$$d := d(F_A, F_B) := \int_0^1 |F_A^{-1}(u) - F_B^{-1}(u)|^2 \mathrm{d}u = (\mu_A - \mu_B)^2 + (\sigma_A - \sigma_B)^2 + 2\sigma_A\sigma_B(1 - \rho_{A,B}),$$

where $\rho_{A,B} \in [0,1]$ is the Pearson correlation coefficient of the points in the quantile-quantile (Q-Q) plot of F_A and F_B [5]. In particular, $\rho_{A,B} = 0$ when at least one distribution has no variability, and $\rho_{A,B} = 1$ (i.e. the shape term in the decomposition is zero) only in case the two distributions have the same quantiles and thus are identical up to a location shift and scaling (i.e. when the two distributions exhibit the same shape).
For two empirical CDFs $\hat{F}_A$ and $\hat{F}_B$ induced by samples from F_A and F_B, respectively, the 2-Wasserstein distance d may be computed by [11]

$$\begin{aligned} d(\hat{F}_A, \hat{F}_B) &\approx \frac{1}{K}\sum_{k=1}^{K}(Q_A^{\alpha_k} - Q_B^{\alpha_k})^2 \\ &\approx (\hat{\mu}_A - \hat{\mu}_B)^2 + (\hat{\sigma}_A - \hat{\sigma}_B)^2 + 2\hat{\sigma}_A\hat{\sigma}_B(1 - \hat{\rho}_{A,B}), \end{aligned}$$

with $(Q_A^{\alpha_k})_{k=1,\ldots,K}$ and $(Q_B^{\alpha_k})_{k=1,\ldots,K}$ denoting the α_k-quantiles of $\hat{F}_A$ and $\hat{F}_B$, respectively, where $\alpha_k \in (0,1)$. In our implementation, we use equidistant quantile levels of the form $\alpha_k = \frac{k-0.5}{K}, k = 1, \ldots, K$, with $K := 1000$ for the computations, although other options are possible [4].
In the above representation, $\hat{\mu}_A$ and $\hat{\mu}_B$ are the respective empirical means, $\hat{\sigma}_A$ and $\hat{\sigma}_B$ are the respective empirical standard deviations, and $\hat{\rho}_{A,B}$ is the sample Pearson correlation coefficient between the α_k-quantiles $(Q_A^{\alpha_k})_{k=1,\ldots,K}$ and $(Q_B^{\alpha_k})_{k=1,\ldots,K}$ corresponding to F_A and F_B, respectively. Note that in our implementation, we set $\hat{\rho}^{A,B} := 0$ if $\hat{\sigma}_A = 0$ or $\hat{\sigma}_B = 0$ (i.e. in case of a point mass), implying that the standard deviation of $(Q_A^{\alpha_k})_{k=1,\ldots,K}$ or $(Q_B^{\alpha_k})_{k=1,\ldots,K}$ is zero.

1.1 Testing using the 2-Wasserstein distance

To test whether two distributions (CDFs) F_A and F_B represented by two samples of size M_A and M_B, respectively, are significantly differentially distributed, we specifically test the null hypothesis $H_0 : F_A = F_B$ against the alternative $H_1 : F_A \neq F_B$ using the 2-Wasserstein distance, while computing a corresponding p-value. Here, we present two conceptually different testing procedures involving the 2-Wasserstein distance. The first, semi-parametric, one uses a permutation test combined with a generalised Pareto distribution approximation to estimate very small p-values [11, 7], which is computationally slow, but applicable to arbitrary (not necessarily continuous) data. The second one uses an asymptotic result to obtain a null distribution [14], which allows fast computation, but is only reliable for continuous variables. We refer to these two approaches as `waddR` SP and `waddR` ASY, respectively.

1.1.1 Semi-parametric permutation test with generalised Pareto distribution approximation (`waddR` SP)

In the semi-parametric testing procedure we combine a classical permutation test with a generalized Pareto distribution approximation to derive a p-value (similar but not identical to [11]). We employ the sample-based 2-Wasserstein distance $d \geq 0$ as a test statistic here, and test $H_0 : d = 0$ against $H_1 : d > 0$ to identify differences in distributions. First, we derive the 2-Wasserstein distance d_0 based on the original, given samples. To construct a permutation setting, we first pool the sample values from F_A and F_B in a vector v. Then, we randomly assign M_A elements of v to condition A and M_B elements of v to condition B. Finally, we derive the corresponding 2-Wasserstein distance based on these newly obtained samples. In this manner, we can theoretically compute all possible N permutations and the respective 2-Wasserstein distances $\mathcal{D} := \{d_1, \ldots, d_N\}$ to get an exact p-value. However, as it is usually computationally infeasible to compute all permutations, we use a subset $\mathcal{D}^* := \{d_1^*, \ldots, d_{N_{\text{sub}}}^*\} \subseteq \mathcal{D}$ of size $N_{\text{sub}} \leq N$ of all the permutations only and the corresponding approximative p-value

$$P_{\text{perm,approx}}(d_0) = \frac{1}{N_{\text{sub}}} \sum_{n=1}^{N_{\text{sub}}} \mathbb{1}_{\{d_n^* \geq d_0\}},$$

with $\mathbb{1}_E$ denoting the indicator function of the event E. Moreover, to avoid p-values of exactly zero [13], we insert a pseudocount, leading to the approximative p-value formula

$$P'_{\text{perm,approx}}(d_0) = \frac{1}{N_{\text{sub}} + 1} \left(1 + \sum_{n=1}^{N_{\text{sub}}} \mathbb{1}_{\{d_n^* \geq d_0\}} \right).$$

According to the above, the p-value has a lower bound of $1/N_{\text{sub}}$ or $1/(N_{\text{sub}}+1)$, respectively. However, in some application areas such as genomics, it is often desired to obtain rather small p-values, as there might be a very large number of tests and therefore a correction for multiple testing [1] is typically required.

To address this, we use the approach in [7] and model the tail of the distribution of the test statistic values obtained by permutations using a generalized Pareto distribution (GPD). The GPD has CDF

$$F(z) = \begin{cases} 1 - (1 + \frac{\xi z}{a})^{-\frac{1}{\xi}}, & \xi \neq 0 \\ 1 - \exp(-\frac{z}{a}), & \xi = 0 \end{cases},$$

where $a > 0$ is a scale and $\xi \in \mathbb{R}$ a shape parameter, and the range of z is $z \geq 0$ for $\xi \geq 0$ and $0 \leq z \leq -\frac{a}{\xi}$ for $\xi < 0$. In the above model, z are the exceedances. That is, for a threshold τ and if the values in $\mathcal{D}^*$ are ordered such that $d_1^* \geq \ldots \geq d_{N_{\text{sub}}}^*$, a set $\mathcal{Z}$ of N_{exc} exceedances $z_1^*, \ldots, z_{N_{\text{exc}}}^*$ is given by $z_n^* := d_n^* - \tau$ for all n such that $d_n^* > \tau$. To estimate the GPD model parameters a and ξ given $\mathcal{Z}$, maximum likelihood estimation is used. Following [7], the p-value of the GPD approximation is calculated by

$$P_{\text{GPD}}(d_0) = \frac{N_{\text{exc}}}{N_{\text{sub}}}(1 - F(d_0 - \tau)),$$

with the factor $\frac{N_{\text{exc}}}{N_{\text{sub}}}$ compensating that $F(z)$ is estimated only on the tail of the distribution of the test statistic values obtained by permutations.

To test whether the exceedances indeed follow the fitted GPD, a goodness-of-fit test based on an Anderson-Darling test statistic is employed. Under its null hypothesis that the exceedances come from a GPD, small p-values P_{AD} indicate that this cannot be assumed. Here, we consider a GPD to be a good fit if $P_{\text{AD}} > 0.05$.

Regarding the selection of the exceedances threshold, we adopt the proposal in [7] and set $N_{\text{exc}} := 250$. If the 250 largest test statistic values obtained by permutations do not follow a GPD according to the goodness-of-fit test, we can iteratively decrease N_{exc} by ten until we reach an acceptable GPD fit. Note that the GPD approximation should not be employed in case a good GPD fit is never obtained, which can be expected to occur very rarely [7]. If a good GPD fit cannot be reached, we set $P_{\text{GPD}}(d_0) := P'_{\text{perm,approx}}(d_0)$ instead as a p-value estimate. Finally, the exceedances threshold τ is set to $\tau := \frac{d^*_{N_{\text{exc}}} - d^*_{N_{\text{exc}}+1}}{2}$ [7].

Overall, a p-value of our semi-parametric (SP) testing procedure is given by

$$P_{\text{SP}}(d_0) = \begin{cases} P_{\text{perm,approx}}(d_0) & \text{if } \sum\limits_{n=1}^{N_{\text{sub}}} \mathbb{1}_{\{d_n^* \geq d_0\}} \geq 10 \\ P_{\text{GPD}}(d_0) & \text{otherwise} \end{cases},$$

see [7] for details and justifications. Note that technically, we only perform the GPD estimation if $P_{\text{perm,approx}}(d_0)$ reaches to zero, and thus we may use $P_{\text{perm,approx}}(d_0)$ instead of the pseudocount-based version $P'_{\text{perm,approx}}(d_0)$ in the above definition of $P_{\text{SP}}(d_0)$.

1.1.2 Testing based on asymptotic theory (`waddR` ASY)

The permutation test combined with the GPD approximation previously introduced can be computationally expensive. Running time could be drastically reduced if there was an asymptotic result for the null distribution of the 2-Wasserstein distance. According to [14], for continuous distributions F_A and F_B, the following asymptotic result for the 2-Wasserstein distance holds under the null hypothesis $H_0 : F_A = F_B$ against the alternative $H_1 : F_A \neq F_B$ in case the CDF F_A has a differentiable probability density function (PDF) f_A bounded away from zero:

$$\frac{M_A M_B}{M_A + M_B} \int_0^1 (\hat{F}_A^{-1}(u) - \hat{F}_B^{-1}(u))^2 \mathrm{d}u \to_d \int_0^1 \frac{(\mathbb{B}(u))^2}{(f_A \circ F_A^{-1}(u))^2} \mathrm{d}u$$

as $M_A, M_B \to \infty$, where $\mathbb{B}$ denotes a standard Brownian bridge in $[0,1]$, and $\to_d$ weak convergence (convergence in distribution). Unfortunately, the asymptotic distribution in the above result is not distribution-free, as it depends on F_A (via f_A and F_A^{-1}, respectively). As F_A is in practice unknown, the above result is not helpful when constructing a testing procedure involving a sample-based 2-Wasserstein distance. To circumvent this shortcoming, it is proposed [14] to not consider the 2-Wasserstein distance between $\hat{F}_A$ and $\hat{F}_B$ as above, but to alternatively look at the 2-Wasserstein distance between $\hat{F}_B(\hat{F}_A^{-1})$ and the uniform distribution $\mathcal{U}_{[0,1]}$ on $[0,1]$, for which under the null hypothesis $H_0 : F_A = F_B$ against the alternative $H_1 : F_A \neq F_B$ and in case the CDF F_A is assumed to be continuous and strictly increasing (i.e. there are no ties in the corresponding sample) it holds that

$$\frac{M_A M_B}{M_A + M_B} \int_0^1 (\hat{F}_B(\hat{F}_A^{-1}(u)) - u)^2 \mathrm{d}u \to_d \int_0^1 (\mathbb{B}(u))^2 \mathrm{d}u$$

as $M_A, M_B \to \infty$ [14]. Thus, we obtain a distribution-free asymptotic distribution $D := \int_0^1 (\mathbb{B}(u))^2 \mathrm{d}u$ for the test statistic $r := \frac{M_A M_B}{M_A+M_B} \int_0^1 (\hat{F}_B(\hat{F}_A^{-1}(u)) - u)^2 \mathrm{d}u$. Note that, as $r \geq 0$, an equivalent formulation of the above is to test $H_0 : r = 0$ against $H_1 : r > 0$.

Even though several different, quite complex-looking, representations for the distribution of D are available [17, and references thererin], we here use a Monte Carlo integration to generate the distribution of D. Precisely, D is approximated as follows:

$$D = \int_0^1 (\mathbb{B}(u))^2 \mathrm{d}u \approx \frac{1}{N} \sum_{n=1}^{N} (\mathbb{B}(u_n^*))^2, \tag{1}$$

where $\{(\mathbb{B}(u_n^*))^2\}_{n=1,\ldots,N}$ are simulated values of $\mathbb{B}^2$ in $[0,1]$ with $u_n^* := \frac{n}{N}$ for $n = 0, \ldots, N$ (i.e. time steps $1/N$). For our specific implementation here, we take $N := 10000$. To generate the (empirical) distribution of D, we compute 10^6 times the integral (1). The results for the PDF and CDF of D obtained by our Monte Carlo method (Figure 1) are very close to those obtained in [17], confirming the validity of our approach.

To compute the value of the test statistic r, we use the approximation

$$r \approx \frac{M_A M_B}{M_A + M_B} \frac{1}{K+1} \sum_{k=0}^{K} \left(\hat{F}_B \left(\hat{F}_A^{-1} \left(\frac{k}{K} \right) \right) - \frac{k}{K} \right)^2$$

with $K := 10000$. A p-value in the above test can then be computed by

$$P_{\mathrm{ASY}}(r) = 1 - G(r),$$

where G is the (empirical) CDF of D obtained by the Monte Carlo method described above.

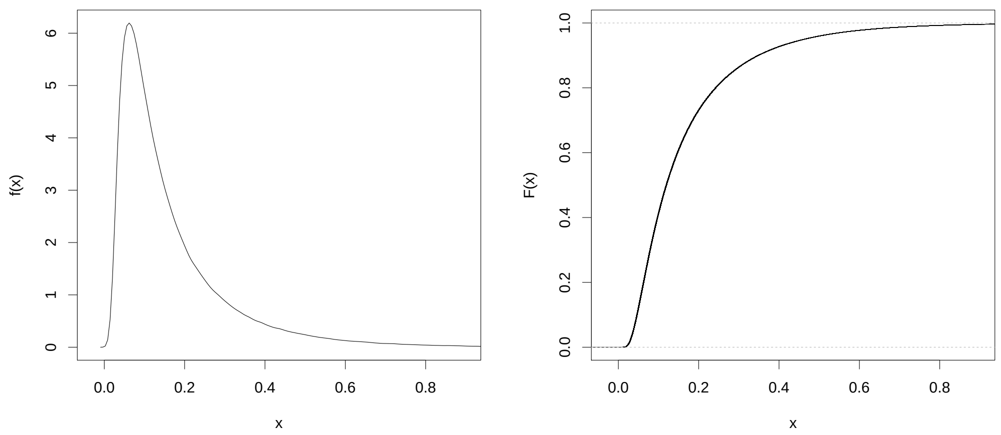

Figure 1: Probability density function f (left) and cumulative distribution function F (right) of $D = \int_0^1 (\mathbb{B}(u))^2 \mathrm{d}u$ obtained by Monte Carlo method

1.2 `waddR` in the context of scRNA-seq data, variant A

We illustrate the `waddR` approach in the context of scRNA-seq data (variant A) in Figure 2. We show one gene for which the method does not detect a DD between two conditions (left-hand side) and one gene for which it does (right-hand side). For the non-DD example, the value of the 2-Wasserstein distance is rather small, combined with a high p-value $P.$nonzero. Accordingly, also the single contributions of location, size and shape are small. This intuitively makes sense, as the two non-zero distributions look fairly similar. Moreover, there is virtually also the same fraction of cells with zero expression across the two conditions (see the point mass at zero), leading to an again high p-value $P.$zero. In contrast, for the DD example, we can see a clear difference in the two non-zero distributions, and also with respect to the fractions of zero expression. This is reflected by a higher value of the 2-Wasserstein distance, along with small p-values. According to the decomposition, differences in location contribute most to the overall difference in non-zero expression.

1.2.1 Discrete-continuous model used for the illustrations in Figure 2

For each condition separately, a distribution F is fitted to the corresponding normalized expression data $x_1, \ldots, x_C \in [0, \infty)$ for a fixed gene g (index omitted here), where C is the respective number of cells. Specifically, we here fit the following discrete-continuous model using an overall density f for gene expression $x \in [0, \infty)$:

$$f(x) = \mathbb{P}(x = 0)\ \mathbb{1}_{\{x=0\}} + (1 - \mathbb{P}(x = 0))\ h(x)\ \mathbb{1}_{\{x>0\}}.$$

The density f is composed of two parts: a discrete part consisting of a point mass at zero, and a continuous part h describing non-zero gene expression in $(0, \infty)$. While the point mass at zero is given

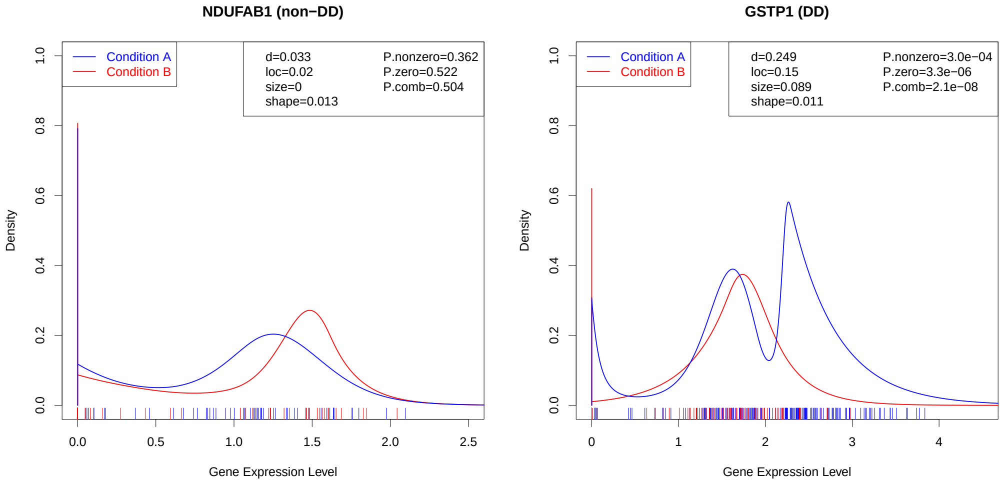

Figure 2: Examples for non-DD (left) and DD (right) genes at a significance level of 5% with respect to the `waddR` test using the 2-Wasserstein distance and its decomposition, based on one replicate in the data set in [20] for the comparison Decidua (condition A) vs. Blood (condition B) for the subset of natural killer cells. For details about the data set, see the main text.
For purely illustrative purposes, we only in this figure here fitted a discrete-continuous model to the gene expression level. However, this model is neither used for the actual testing procedure nor the calculation of the decomposition of the 2-Wasserstein distance. For details concerning the model used for illustration, see Supplement Section 1.2.1. The bars at the bottom represent the actual normalized expression measurements over the cells.

by

$$\mathbb{P}(x=0) = \frac{\#\{x_c \,|\, x_c = 0,\ c = 1, \dots, C\}}{C},$$

for the continuous part, we use a logspline density estimate h for censored data [8] to fit gene expression in $(0, \infty)$.
Note that this discrete-continuous model is only used to create the illustrations in Figure 2, but itself does not play a role in the methods proposed in this paper.

Table 1: Settings for the simulations based on normal distribution models, with case acronyms indicating whether by construction there are differences with respect to location (L), size (Si) and/or shape (Sh) or no differences (N), where ✕ corresponds to "no" and ✓corresponds to "yes"

case	comparison	different location	different size	different shape
L	$\mathcal{N}(0,1)$ vs. $\mathcal{N}(2,1)$	✓	✕	✕
Si	$\mathcal{N}(0,1)$ vs. $\mathcal{N}(0,3)$	✕	✓	✕
Sh	$\mathcal{N}(6.5,\sqrt{13.25})$ vs. $\frac{1}{2}\mathcal{N}(3,1)+\frac{1}{2}\mathcal{N}(10,1)$	✕	✕	✓
LSi	$\mathcal{N}(0,1)$ vs. $\mathcal{N}(2,3)$	✓	✓	✕
LSh	$\mathcal{N}(4.5,\sqrt{13.25})$ vs. $\frac{1}{2}\mathcal{N}(3,1)+\frac{1}{2}\mathcal{N}(10,1)$	✓	✕	✓
SiSh	$\mathcal{N}(6.5,1.5)$ vs. $\frac{1}{2}\mathcal{N}(3,1)+\frac{1}{2}\mathcal{N}(10,1)$	✕	✓	✓
LSiSh	$\mathcal{N}(4.5,1.5)$ vs. $\frac{1}{2}\mathcal{N}(3,1)+\frac{1}{2}\mathcal{N}(10,1)$	✓	✓	✓
N	$\mathcal{N}(0,1)$ vs. $\mathcal{N}(0,1)$	✕	✕	✕

2 Simulations based on normal distribution models

2.1 Normal distribution models

In order to assess the `waddR` SP and ASY testing procedures and to examine the usability of the decomposition of the 2-Wasserstein distance to detect differences in location, size and shape, we first consider a simulation study based on (mixtures of) normal distributions. Normal distributions provide a good initial choice to verify the behaviour of the different components of the decomposition of the 2-Wasserstein distance, due to their convenience and tractability. In particular, it is well-known that a mixture

$$M \sim \omega\mathcal{N}(\mu_1,\sigma_1)+(1-\omega)\mathcal{N}(\mu_2,\sigma_2)$$

of two normal distributions with means μ_1 and μ_2 and standard deviations σ_1 and σ_2, respectively, and weight $\omega \in (0,1)$ has mean

$$\mathbb{E}(M) = \omega\mu_1 + (1-\omega)\mu_2$$

and variance

$$\mathrm{Var}(M) = \omega(\mu_1 - \mathbb{E}(M))^2 + (1-\omega)(\mu_2 - \mathbb{E}(M))^2 + \omega\sigma_1^2 + (1-\omega)\sigma_2^2.$$

With this background, we can conveniently create (mixtures of) normal distributions that differ in location, size or shape or combinations thereof. Table 1 shows all the models we consider for all the possible eight types of differences. Note that, in accordance with the goals of this section, we here consider only comparison models which are supposed to have a clear difference between conditions A and B in the following sense:

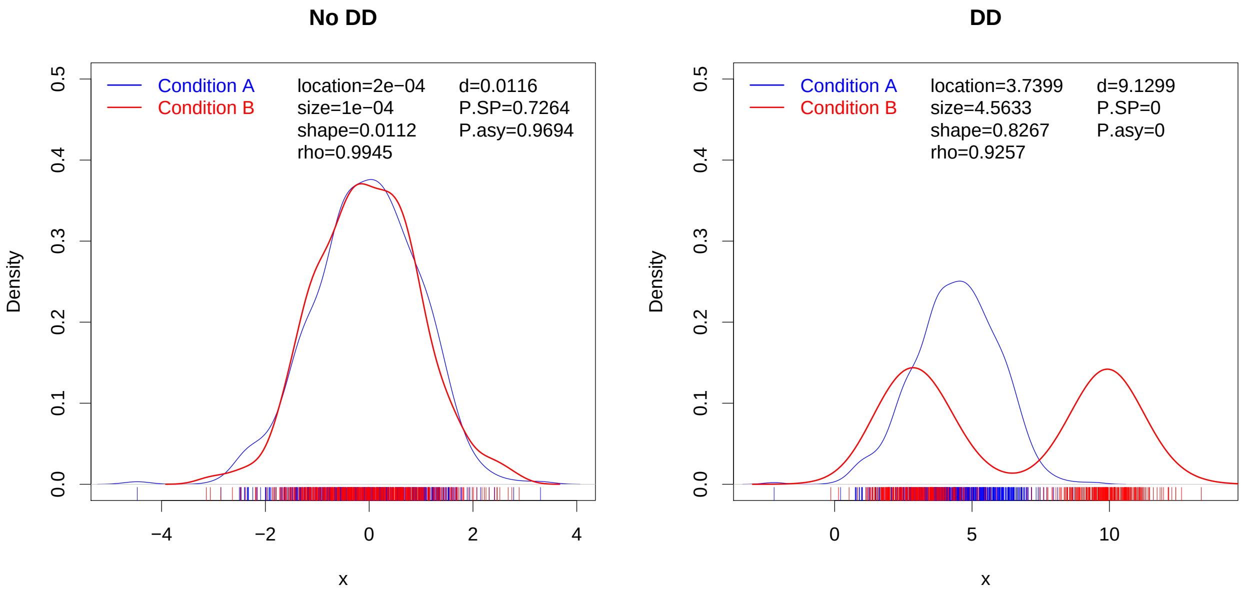

Figure 3: Examples based on normal distribution models for no DD (left) and DD (right) at a significance level of 5% based on the semi-parametric (SP) and asymptotic theory-based (ASY) tests using the 2-Wasserstein distance and its decomposition. For purely illustrative purposes, we fit a density to the samples (using the R function `density`). However, note that the density itself is neither used for the actual testing procedures nor the calculation of the decomposition of the 2-Wasserstein distance. The bars at the bottom represent the actual sample values.

- in case of differential location, the absolute deviation of the means is equal to 2,
- in case of differential size, the absolute deviation of the standard deviations is at least equal to 2, and
- in case of differential shape, we compare a unimodal against a bimodal distribution.

An illustration is given in Figure 3, with one example in which no difference between two distributions is found on the left-hand side (No DD; corresponding to case N in Table 1) and one example in which a difference is detected on the right-hand side (DD; corresponding to case LSiSh in Table 1), where each distribution is represented by a corresponding sample of size $M := M_A = M_B = 500$ here. For the no DD example, the value of the 2-Wasserstein distance is rather small, combined with high p-values. Accordingly, also the single contributions of location, size and shape are small, while the correlation coefficient is very high. This makes sense, as the two distributions (densities) look quite similar, as is to be expected due to the underlying model. In contrast, for the DD example, we can see a clear difference in the two distributions (densities), which is reflected by the rather high value of the 2-Wasserstein distance, along with zero p-values. According to the decomposition, both differences in location and variability (size and shape) contribute to the overall difference, in accordance with the underlying model.

For each case, we report results over 1000 runs of the respective comparison. In Supplement Section 2.2, we present results for the scenario in which we have the same sample size for the conditions A and B, i.e., $M := M_A = M_B$, and we choose $M \in \{25, 50, 75, 100, 500, 1000, 5000\}$ for our simulation studies here.
Results for the case when there are different sample sizes for conditions A and B, i.e., $M_A \neq M_B$, are deferred to Supplement Section 2.3.

2.2 Results for equal sample sizes $M := M_A = M_B$

Results are provided for the two 2-Wasserstein distance-based testing procedures, i.e. the semi-parametric (`waddR` SP) test and the test based on asymptotics (`waddR` ASY), as well as for the classical, well-known Kolmogorov-Smirnov (KS) test which is used as a reference to compare with. The results for `waddR` SP are based on 10000 permutations.
Detection powers and type I errors are summarized in Figure 4 for a relevant range of thresholds between 0% and 5%, where additional results providing more details can be found in Figure 6 and Table 2. The detection powers of all considered tests are very good in practically each case for reasonably large sample sizes ($M \geq 100$). For smaller sample sizes ($M \leq 75$), the results are more diverse. With small sample sizes, the `waddR` tests perform slightly worse than the KS test for case Sh, in which there is the overall lowest detection power for all the tests. This intuitively appears to make sense, as it might be difficult to detect differences only caused by changes in shape based on small samples. In contrast, for the other cases when considering $M \leq 75$, the `waddR` tests have a similar or higher detection power than the KS reference test. The improvement is most visible for case Si involving differences in size. The `waddR` ASY test exhibits some weaknesses in terms of detection power for $M = 25$, but gives (naturally) more reliable results for larger samples with $M \geq 50$. In case of small sample sizes M, the `waddR` SP test typically yields better prediction powers, but is computationally much more expensive. Across all sample sizes, the type I error rates for the two `waddR` tests are fairly similar, see Figure 4. Even though they are typically a bit higher than those for the KS reference test, they are still overall at an acceptable and reasonable level.
Another aim of this simulation study is to confirm the validity of the decomposition of the 2-Wasserstein distance into location, size and shape parts, along with the ability of our tests to identify the major cause(s) of differences between distributions. To this end, Figure 5 shows the average fractions of the location, size and shape parts with respect to the overall 2-Wasserstein distance for the `waddR` SP test based on those (of the 1000) runs with a p-value less than or equal to 5%. For each case, the location, size and shape contributions mirror quite well what is to be expected from theory according to Table 1, where this reflection meaningfully becomes more and more clear the larger the considered sample size M is. For instance, for case Si, in which we have equal location and shape but unequal size by construction, the size component is by far the strongest, while the location and shape components get negligible. Or, in case LSh, where we have equal size but unequal location and shape by design, the location and shape parts clearly set the tone, while the size part can be neglected. Likewise statements hold for the other cases, too. Overall, the `waddR` approach is able to detect and identify the causes of the difference between two distributions correctly, which confirms one of the main aspects discussed in our paper. Problematic results here only occur for case Sh for small sample sizes, which is however in line with what we discussed before, namely that it appears to be quite hard to identify differences solely in shape based on small samples.

A similar simulation study based on Gamma distribution models, which again confirms the soundness and competitiveness of the `waddR` tests, can be found in Supplement Section 3.

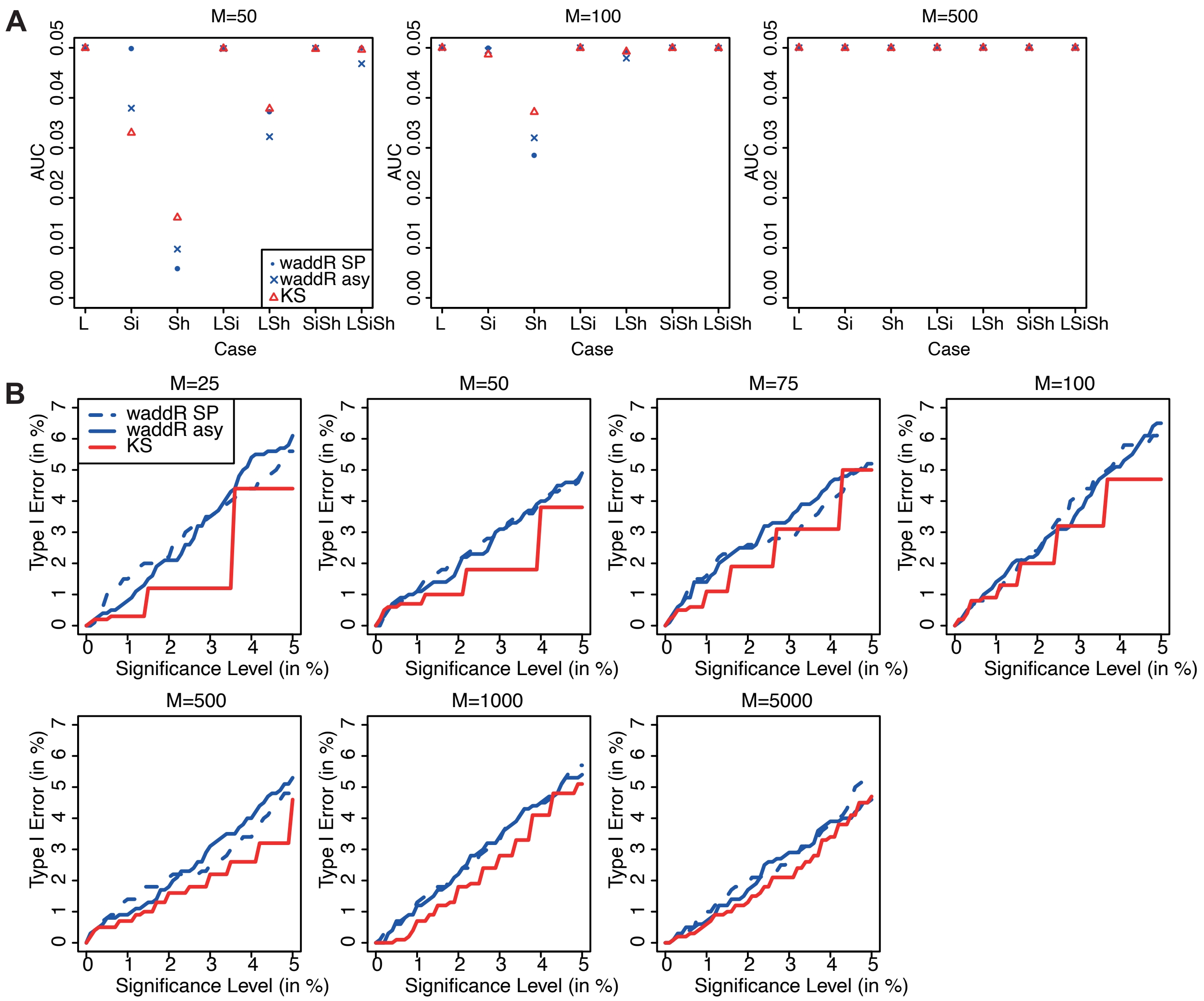

Figure 4: Normal distribution models, cases according to Table 1, results based on unadjusted p-values for sample sizes $M := M_A = M_B$. (A) Detection powers for the `waddR` tests and the KS test, based on 1000 runs. Detection powers are summarized using the area under curve (AUC) values corresponding to the curve obtained when plotting detection powers vs. significance levels for significance levels from 0% to 5%. (B) Type I errors (Case N according to Table 1) vs. significance levels for significance levels from 0% to 5%, based on 1000 runs, for the `waddR` tests and the KS test.

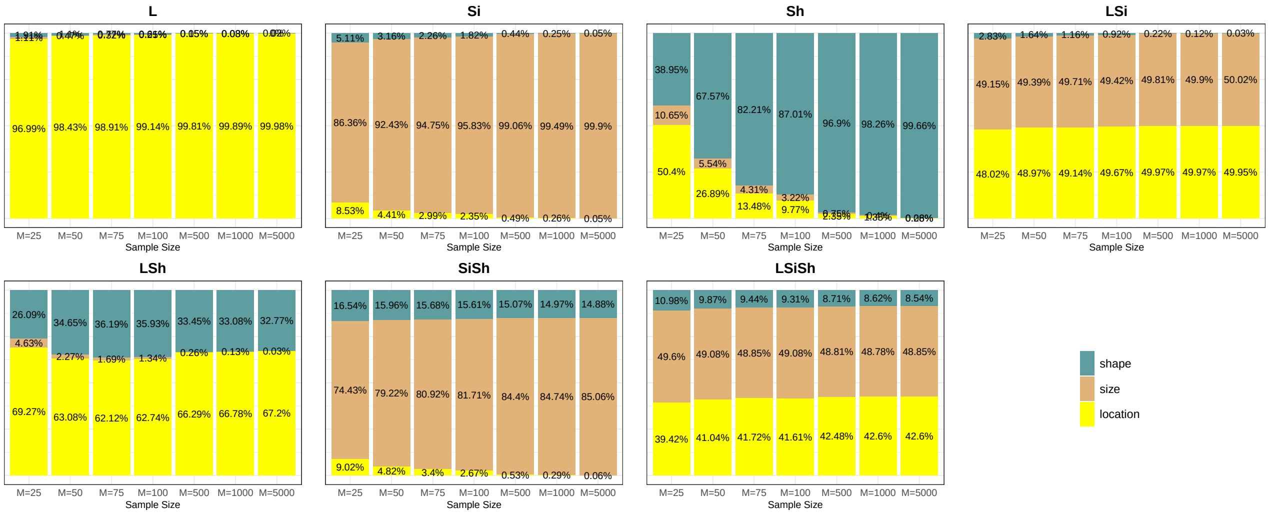

Figure 5: Normal distribution models: Fractions of location, size and shape parts in the 2-Wasserstein distance based on averages over those of 1000 runs with a corresponding unadjusted p-value being less than or equal to 5% in the `waddR` SP test. Cases according to Table 1, sample sizes $M := M_A = M_B$.

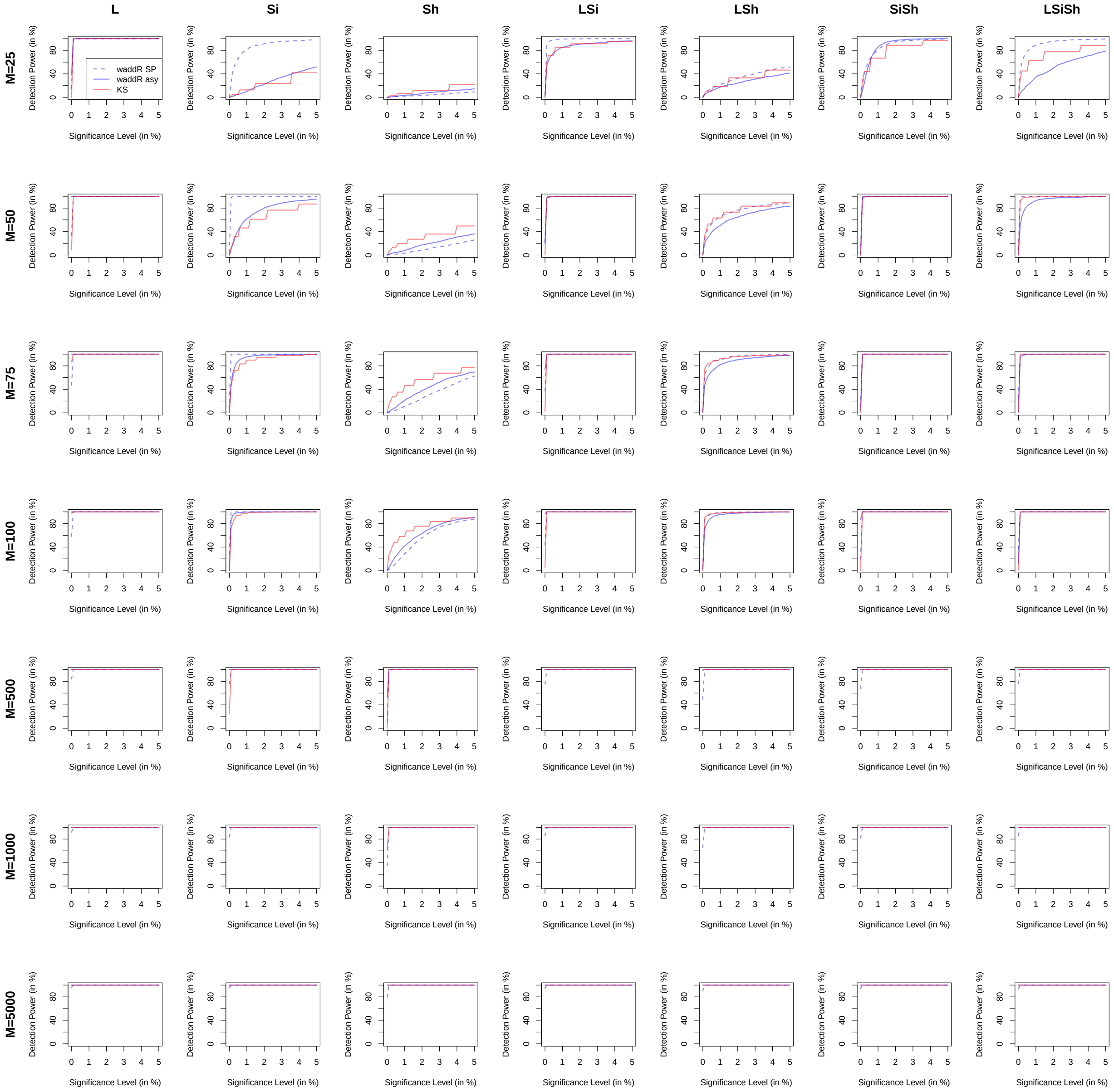

Figure 6: Normal distribution models: Detection powers vs. significance levels for significance levels from 0% to 5%, based on 1000 runs, for the `waddR` tests and the KS test. Results based on unadjusted p-values. Rows: sample sizes $M := M_A = M_B$; columns: cases according to Table 1.

Table 2: Normal distribution models: Detection powers (in %) and type I error (in %; case N), respectively, of the `waddR` tests and the KS test at a 5% significance level, based on 1000 runs, with sample sizes $M := M_A = M_B$. Cases according to Table 1. Results based on unadjusted p-values; in brackets: results based on adjusted p-values for multiple testing according to Benjamini-Hochberg [1].

case \ M		25	50	75	100	500	1000	5000
L	`waddR` SP	100	100	100	100	100	100	100
		(100)	(100)	(100)	(100)	(100)	(100)	(100)
	`waddR` ASY	100	100	100	100	100	100	100
		(100)	(100)	(100)	(100)	(100)	(100)	(100)
	KS	100	100	100	100	100	100	100
		(100)	(100)	(100)	(100)	(100)	(100)	(100)
Si	`waddR` SP	97.7	100	100	100	100	100	100
		(97.7)	(100)	(100)	(100)	(100)	(100)	(100)
	`waddR` ASY	51.7	95.3	99.8	100	100	100	100
		(0.0)	(94.6)	(99.8)	(100)	(100)	(100)	(100)
	KS	42.8	87.0	99.1	99.9	100	100	100
		(12.6)	(87.0)	(99.1)	(99.9)	(100)	(100)	(100)
Sh	`waddR` SP	9.2	25.8	62.4	87.5	100	100	100
		(0.1)	(0.0)	(0.1)	(83.8)	(100)	(100)	(100)
	`waddR` ASY	14.6	36.2	69.6	90.5	100	100	100
		(0.0)	(0.0)	(30.6)	(88.5)	(100)	(100)	(100)
	KS	21.9	49.5	77.6	89.5	100	100	100
		(1.4)	(26.9)	(67.6)	(89.5)	(100)	(100)	(100)
LSi	`waddR` SP	99.9	100	100	100	100	100	100
		(99.9)	(100)	(100)	(100)	(100)	(100)	(100)
	`waddR` ASY	96.2	100	100	100	100	100	100
		(96.0)	(100)	(100)	(100)	(100)	(100)	(100)
	KS	95.5	100	100	100	100	100	100
		(95.5)	(100)	(100)	(100)	(100)	(100)	(100)
LSh	`waddR` SP	51.7	89.6	99.5	99.9	100	100	100
		(23.9)	(87.2)	(99.5)	(99.9)	(100)	(100)	(100)
	`waddR` ASY	41.5	83.4	97.9	99.8	100	100	100
		(12.7)	(79.1)	(97.8)	(99.8)	(100)	(100)	(100)
	KS	46.4	89.0	98.3	99.9	100	100	100
		(33.2)	(89.0)	(98.3)	(99.9)	(100)	(100)	(100)
SiSh	`waddR` SP	99.3	100	100	100	100	100	100
		(99.3)	(100)	(100)	(100)	(100)	(100)	(100)
	`waddR` ASY	100	100	100	100	100	100	100
		(100)	(100)	(100)	(100)	(100)	(100)	(100)
	KS	96.9	100	100	100	100	100	100
		(96.9)	(100)	(100)	(100)	(100)	(100)	(100)
LSiSh	`waddR` SP	99.3	100	100	100	100	100	100
		(99.3)	(100)	(100)	(100)	(100)	(100)	(100)
	`waddR` ASY	78.7	99.7	100	100	100	100	100
		(64.0)	(99.7)	(100)	(100)	(100)	(100)	(100)
	KS	88.5	100	100	100	100	100	100
		(88.5)	(100)	(100)	(100)	(100)	(100)	(100)
N	`waddR` SP	5.6	4.9	5.4	6.3	4.8	5.7	5.6
		(0.0)	(0.0)	(0.0)	(0.1)	(0.0)	(0.0)	(0.0)
	`waddR` ASY	6.1	4.9	5.2	6.5	5.3	5.4	4.6
		(0.0)	(0.0)	(0.1)	(0.1)	(0.0)	(0.0)	(0.0)
	KS	4.4	3.8	5.0	4.7	4.6	5.1	4.7
		(0.0)	(0.0)	(0.1)	(0.1)	(0.0)	(0.0)	(0.0)

2.3 Results for unequal sample sizes $M_A \neq M_B$

Here, we present results of the simulation study using normal distribution models for the case when conditions A and B have different sample sizes, that is, $M_A \neq M_B$. We choose $M_A := 100$ and $M_B \in \{90, 75, 50, 25, 200, 600, 1100\}$ for our simulations, such that the absolute differences $\delta := |M_A - M_B|$ between the sample sizes in the two conditions are $\delta \in \{10, 25, 50, 75, 100, 500, 1000\}$.
For $M_A \neq M_B$, the results are essentially quite similar to those for $M_A = M_B$ from Supplement Section 2.2. More precisely, the results for $M_A \neq M_B$, appear to depend on the magnitudes of M_A and M_B themselves (in particular on M_B, as $M_A = 100$ for each case), but not so much on the magnitude of the difference between the sample sizes, see Figures 7, 8 and 9, as well as Table 3.

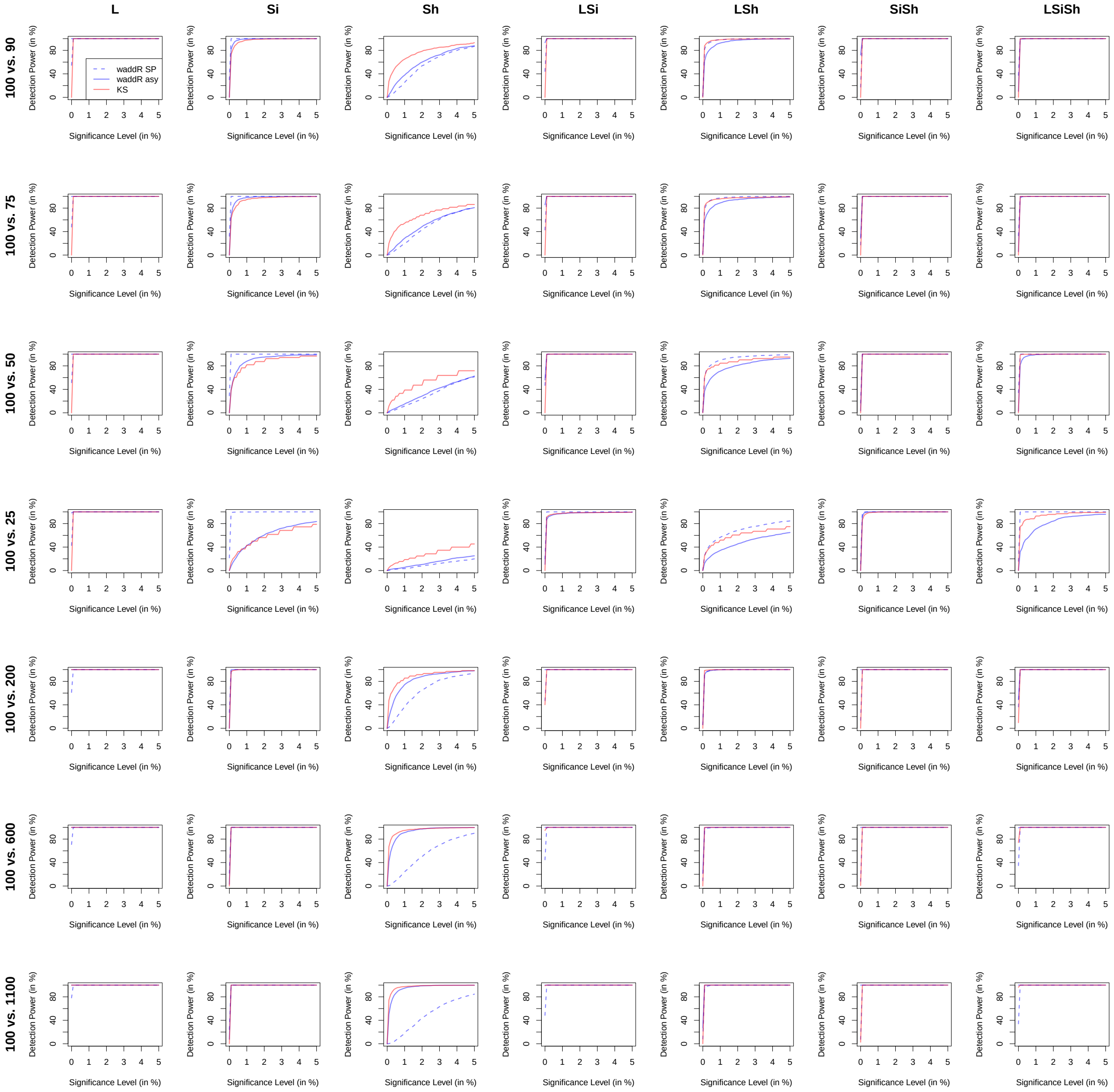

Figure 7: Normal distribution models: Detection powers vs. significance levels for significance levels from 0% to 5%, based on 1000 runs, for the `waddR` tests and the KS test. Results based on unadjusted p-values. Rows: sample sizes $M_A \neq M_B$ for each condition; columns: cases according to Table 1.

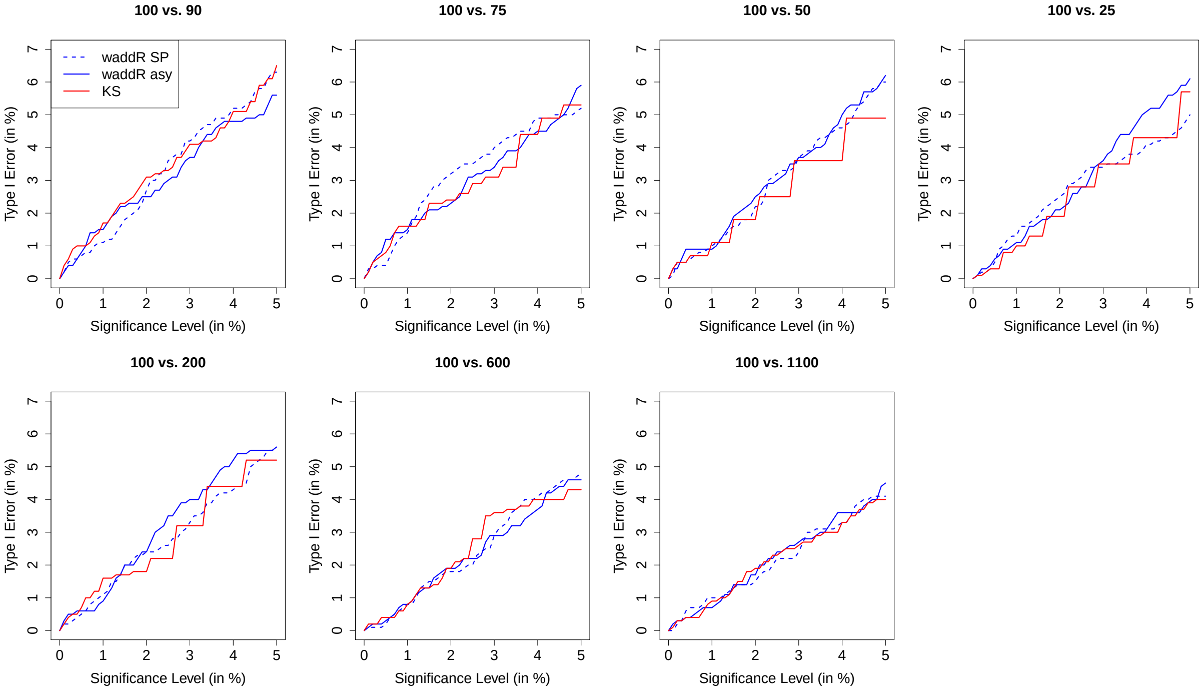

Figure 8: Normal distribution models: Type I errors (Case N according to Table 1) vs. significance levels for significance levels from 0% to 5%, based on 1000 runs, for the `waddR` tests and the KS test. Results based on unadjusted p-values for sample sizes $M_A \neq M_B$ for each condition.

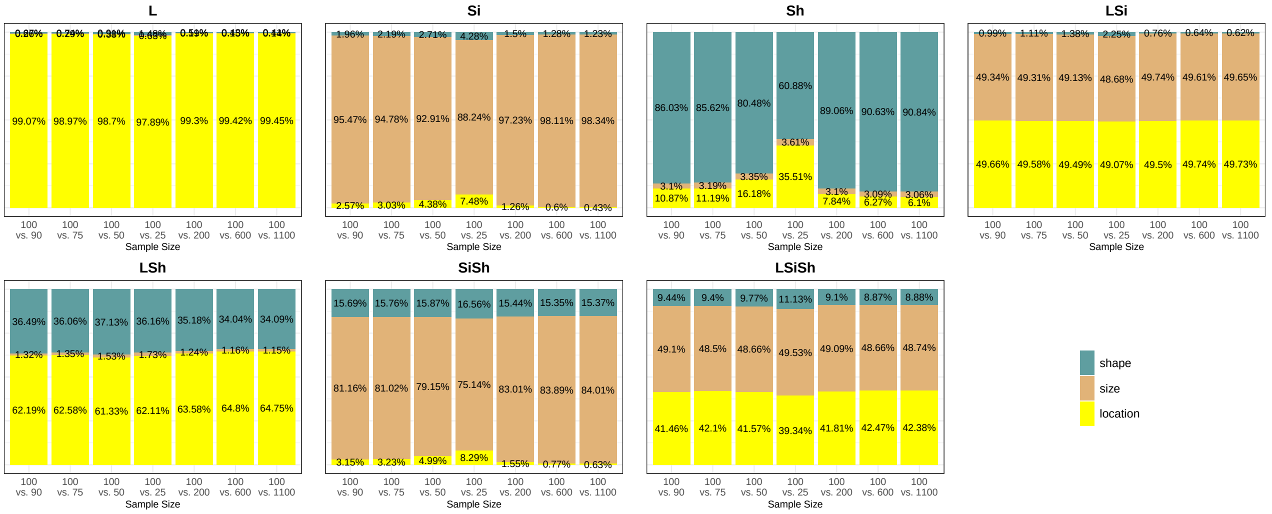

Figure 9: Normal distribution models: Fractions of location, size and shape parts in the 2-Wasserstein distance based on averages over those of 1000 runs with a corresponding unadjusted p-value being less than or equal to 5% in the `waddR` SP test. Cases according to Table 1, sample sizes $M_A \neq M_B$ for each condition.

Table 3: Normal distribution models: Detection powers (in %) and type I error (in %; case N), respectively, of the **waddR** tests and the KS test at a 5% significance level, based on 1000 runs, with sample sizes $M_A \neq M_B$ with difference $\delta := |M_A - M_B|$. Cases according to Table 1. Results based on unadjusted p-values; in brackets: results based on adjusted p-values for multiple testing according to Benjamini-Hochberg [1].

case		$M_A = 100$ $M_B = 90$ $\delta = 10$	$M_A = 100$ $M_B = 75$ $\delta = 25$	$M_A = 100$ $M_B = 50$ $\delta = 50$	$M_A = 100$ $M_B = 25$ $\delta = 75$	$M_A = 100$ $M_B = 200$ $\delta = 100$	$M_A = 100$ $M_B = 600$ $\delta = 500$	$M_A = 100$ $M_B = 1100$ $\delta = 1000$
L	`waddR` SP	100	100	100	100	100	100	100
		(100)	(100)	(100)	(100)	(100)	(100)	(100)
	`waddR` ASY	100	100	100	100	100	100	100
		(100)	(100)	(100)	(100)	(100)	(100)	(100)
	KS	100	100	100	100	100	100	100
		(100)	(100)	(100)	(100)	(100)	(100)	(100)
Si	`waddR` SP	100	100	100	100	100	100	100
		(100)	(100)	(100)	(100)	(100)	(100)	(100)
	`waddR` ASY	100	100	98.8	83.6	100	100	100
		(100)	(100)	(98.8)	(78.5)	(100)	(100)	(100)
	KS	99.9	99.7	96.7	78.8	100	100	100
		(99.9)	(99.7)	(96.7)	(74.5)	(100)	(100)	(100)
Sh	`waddR` SP	87.0	80.8	61.3	19.9	93.8	90.0	84.6
		(80.2)	(67.3)	(0.0)	(0.0)	(92.1)	(84.2)	(70.9)
	`waddR` ASY	87.7	80.8	62.7	25.1	98.0	99.7	99.8
		(85.1)	(68.8)	(3.9)	(0.7)	(97.8)	(99.7)	(99.8)
	KS	93.1	86.2	71.7	45.3	98.3	99.6	99.8
		(91.1)	(83.6)	(63.7)	(18.7)	(98.3)	(99.6)	(99.8)
LSi	`waddR` SP	100	100	100	100	100	100	100
		(100)	(100)	(100)	(100)	(100)	(100)	(100)
	`waddR` ASY	100	100	100	99.4	100	100	100
		(100)	(100)	(100)	(99.4)	(100)	(100)	(100)
	KS	100	100	100	99.5	100	100	100
		(100)	(100)	(100)	(99.5)	(100)	(100)	(100)
LSh	`waddR` SP	100	100	99.1	84.6	100	100	100
		(100)	(100)	(99.1)	(81.2)	(100)	(100)	(100)
	`waddR` ASY	99.6	99.1	92.8	64.8	100	100	100
		(99.6)	(99.1)	(92.1)	(50.5)	(100)	(100)	(100)
	KS	99.8	99.3	95.0	74.9	100	100	100
		(99.8)	(99.3)	(95.0)	(66.9)	(100)	(100)	(100)
SiSh	`waddR` SP	100	100	100	100	100	100	100
		(100)	(100)	(100)	(100)	(100)	(100)	(100)
	`waddR` ASY	100	100	100	100	100	100	100
		(100)	(100)	(100)	(100)	(100)	(100)	(100)
	KS	100	100	100	100	100	100	100
		(100)	(100)	(100)	(100)	(100)	(100)	(100)
LSiSh	`waddR` SP	100	100	100	100	100	100	100
		(100)	(100)	(100)	(100)	(100)	(100)	(100)
	`waddR` ASY	100	100	100	96.0	100	100	100
		(100)	(100)	(100)	(95.9)	(100)	(100)	(100)
	KS	100	100	100	99.2	100	100	100
		(100)	(100)	(100)	(99.2)	(100)	(100)	(100)
N	`waddR` SP	6.3	5.2	6.0	5.0	5.5	4.8	4.1
		(0.1)	(0.1)	(0.0)	(0.0)	(0.1)	(0.1)	(0.0)
	`waddR` ASY	5.6	5.9	6.2	6.1	5.6	4.6	4.5
		(0.1)	(0.1)	(0.1)	(0.0)	(0.1)	(0.1)	(0.1)
	KS	6.5	5.3	4.9	5.7	5.2	4.3	4.0
		(0.1)	(0.1)	(0.0)	(0.0)	(0.1)	(0.1)	(0.0)

3 Simulations based on Gamma distribution models

3.1 Gamma distribution models

After having evaluated the `waddR` methods in simulations based on normal distribution models in Supplement Section 2, we now focus on simulations based on Gamma distributions in a similar manner, to further check the validity and properties of the approaches. We recall that a Gamma-distributed random variable $X \sim \mathcal{G}(a, s)$ with shape parameter $a > 0$ and scale parameter $s > 0$ has probability density function

$$f(x) = \frac{1}{s^a \Gamma(a)} x^{a-1} \exp\left(-\frac{x}{s}\right) \mathbb{1}_{\{x>0\}},$$

with $\Gamma()$ denoting the Gamma function and $\mathbb{1}_E$ the indicator function of the event E. Moreover, it has mean $\mathbb{E}(X) = as$ and variance $\mathrm{Var}(X) = as^2$ (i.e. standard deviation $\mathrm{sd}(X) = \sqrt{a}s$).
As a control, we take the unimodal Gamma distribution $\mathcal{G}(2, 2)$ with mean 4 and standard deviation $2\sqrt{2}$. As for the normal distribution models, we here consider differential distributions of different types which are supposed to have a clear difference between conditions A and B in the following sense:

- in case of differential location, the absolute deviation of the means is equal to 2,
- in case of differential size, the absolute deviation of the standard deviations is at least equal to $\sqrt{2}$, and
- in case of differential shape, we compare a unimodal and skewed Gamma distribution (i.e. $a > 1$) with an exponentially shaped Gamma distribution (i.e. $a \leq 1$).

The specific different case settings are shown in Table 4. For each case, we report results over 1000 runs of the respective comparison and consider a scenario involving equal sample sizes $M := M_A = M_B$, where $M \in \{25, 50, 75, 100, 500, 1000, 5000\}$. The results for `waddR` SP are based on 10000 permutations.

3.2 Results

Detection powers and type I errors are exhibited in Figures 10 and 11, respectively, for a range of thresholds α between 0% and 5%, where the explicit results for the standard level of $\alpha = 5\%$ are listed in Table 5. For large sample sizes ($M \geq 500$), all tests essentially show equal, very good detection powers. For small to moderate sample sizes ($M \leq 100$), the results regarding detection powers are more diverse in the following sense. In case SiSh, it appears to be most difficult for all the tests to detect differences appropriately, with the tests based on asymptotic theory (`waddR` ASY and KS) performing quite similarly and both better than the semi-parametric `waddR` SP approach. This may give a hint to the fact that differences with respect to location, which should not be present according to the theoretical model, might be easier to detect than joint differences in size and shape. In case LSh, both the `waddR` ASY and the KS test already yield good detection powers for $M \leq 100$. They outperform the `waddR` SP test, where the performance differences however become remarkably smaller with increasing sample size. For case LSi, both `waddR` tests perform equally well and slightly outperform the KS test regarding detection powers. For case LSiSh, the `waddR` ASY and the KS test perform very well with respect to detection power, and so does the `waddR` SP test for sample sizes $M \geq 75$.

Table 4: Settings for the simulations based on Gamma distribution models, with case acronyms indicating whether by construction there are differences with respect to location (L), size (Si) and/or shape (Sh) or no differences (N), where ✗ corresponds to "no" and ✓corresponds to "yes"

case	comparison	different location	different size	different shape
SiSh	$\mathcal{G}(2,2)$ vs. $\mathcal{G}(\frac{2}{3},6)$	✗	✓	✓
LSh	$\mathcal{G}(2,2)$ vs. $\mathcal{G}(\frac{1}{2},4)$	✓	✗	✓
LSi	$\mathcal{G}(2,2)$ vs. $\mathcal{G}(2,3)$	✓	✓	✗
LSiSh	$\mathcal{G}(2,2)$ vs. $\mathcal{G}(\frac{1}{6},12)$	✓	✓	✓
N	$\mathcal{G}(2,2)$ vs. $\mathcal{G}(2,2)$	✗	✗	✗

With respect to type I error, the KS test typically performs at least a bit better than the `waddR` tests for small sample sizes ($M \leq 75$), while for larger sample sizes ($M \geq 100$), such differences tend to vanish, with all tests essentially performing similarly. Overall, the type I errors of all tests are at an acceptable and reasonable level.
Figure 12 exhibits the average fractions of the location, size and shape parts in the overall 2-Wasserstein distance for the `waddR` SP test based on those (of the 1000) runs with a p-value less than or equal to 5%. In general, the fractions of location, size and shape parts mirror well what is to be expected from theory according to Table 4. Specifically, the location part in case SiSh, the size part in case LSh and the shape part in case LSi are basically minor to negligible compared to the other components in the corresponding setting, while for case LSiSh, as to be expected, there are relevant contributions from all the three components. These observations can already be witnessed to some extent for small sample sizes ($M \leq 50$), except maybe for case SiSh, and meaningfully, the patterns expected from theory (Table 4) become more obvious the larger the sample size is and are well visible for $M \geq 75$ in all cases.

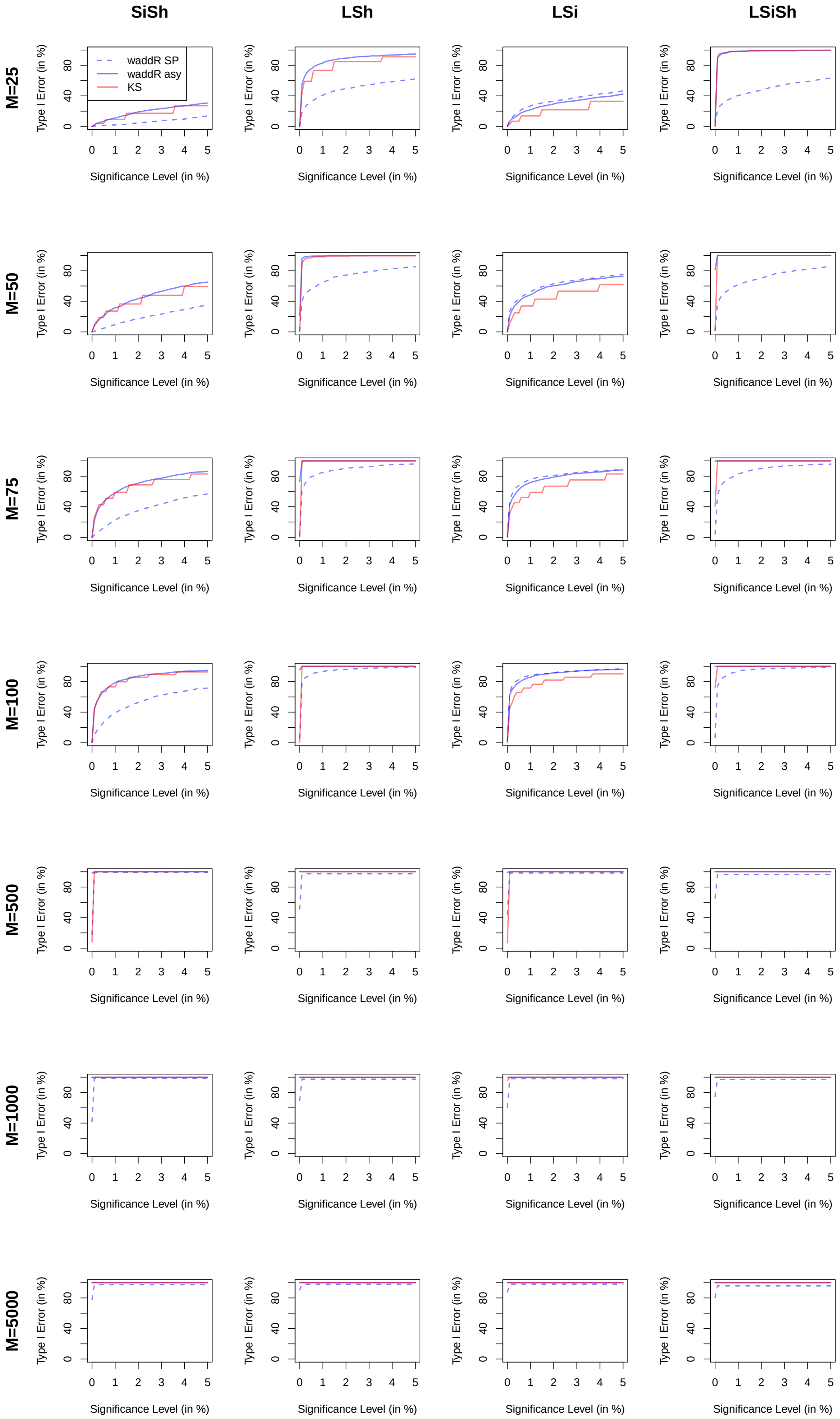

Figure 10: Gamma distribution models: Detection powers vs. significance levels for significance levels from 0% to 5%, based on 1000 runs, for the `waddR` tests and the KS test. Results based on unadjusted p-values. Rows: sample sizes $M := M_A = M_B$; columns: cases according to Table 4.

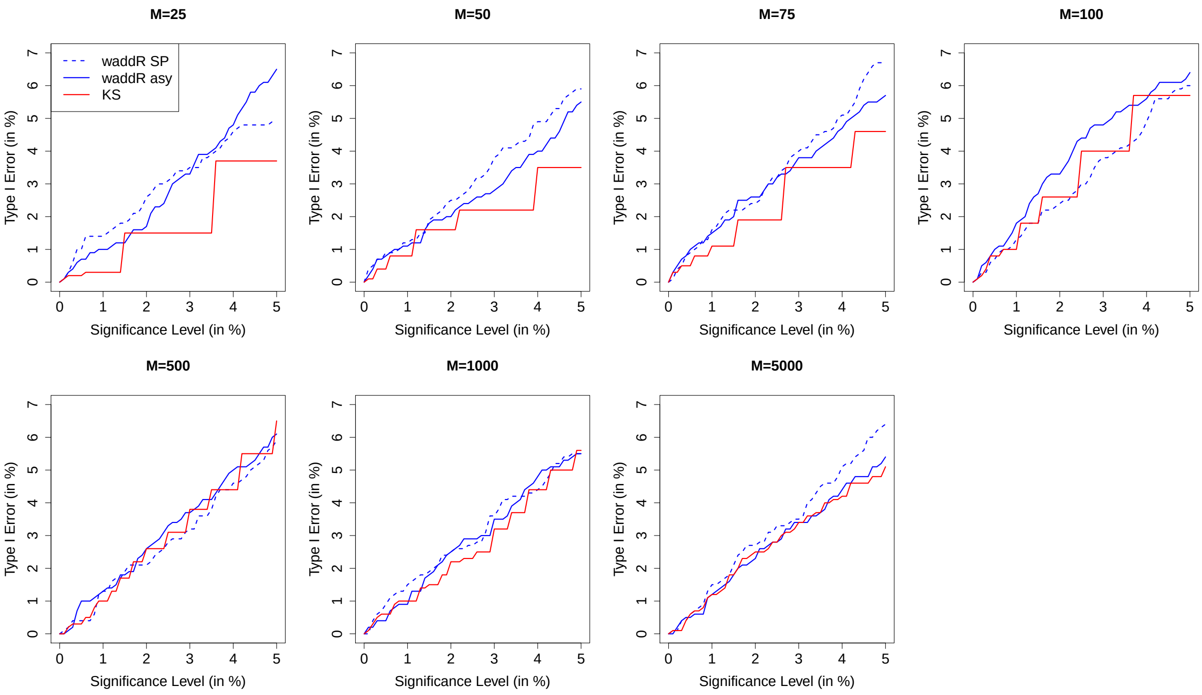

Figure 11: Gamma distribution models: Type I errors (Case N according to Table 4) vs. significance levels for significance levels from 0% to 5%, based on 1000 runs, for the `waddR` tests and the KS test. Results based on unadjusted p-values for sample sizes $M := M_A = M_B$.

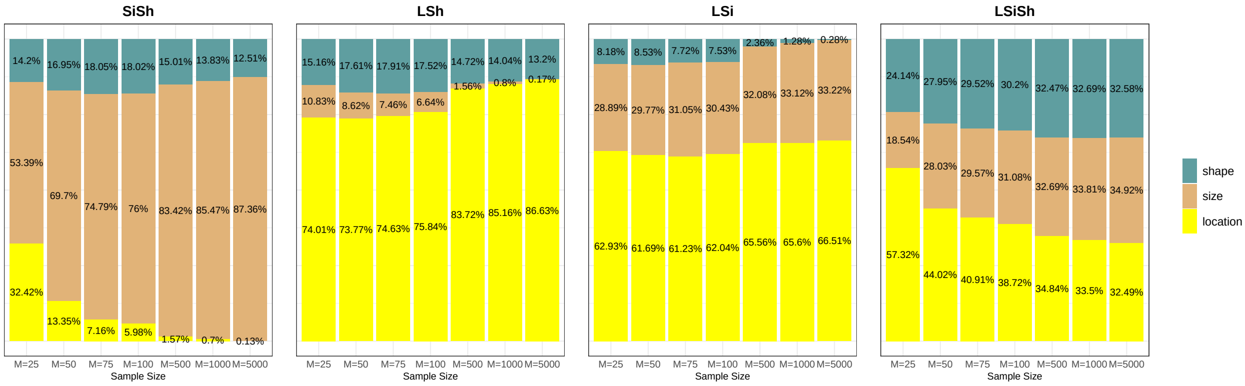

Figure 12: Gamma distribution models: Fractions of location, size and shape parts in the 2-Wasserstein distance based on averages over those of 1000 runs with a corresponding unadjusted p-value being less than or equal to 5% in the `waddR` SP test. Cases according to Table 4, sample sizes $M := M_A = M_B$.

Table 5: Gamma distribution models: Detection powers (in %) and type I error (in %; case N), respectively, of the waddR tests and the KS test at a 5% significance level, based on 1000 runs, with sample sizes $M := M_A = M_B$. Cases according to Table 4. Results based on unadjusted p-values; in brackets: results based on adjusted p-values for multiple testing according to Benjamini-Hochberg [1].

case \ M		25	50	75	100	500	1000	5000
SiSh	waddR SP	13.8	35.9	56.8	71.5	99.3	98.5	97.3
		(0.2)	(0.1)	(26.4)	(62.8)	(99.3)	(98.5)	(97.3)
	waddR ASY	30.7	65.2	86.5	94.6	100	100	100
		(0.6)	(45.9)	(84.2)	(94.3)	(100)	(100)	(100)
	KS	27.1	59.3	82.9	92.7	100	100	100
		(4.0)	(47.8)	(75.6)	(92.7)	(100)	(100)	(100)
LSh	waddR SP	62.3	85.3	95.9	98.5	97.4	97.5	97.9
		(53.1)	(82.9)	(95.7)	(98.5)	(97.4)	(97.5)	(97.9)
	waddR ASY	94.9	99.8	100	100	100	100	100
		(94.6)	(99.8)	(100)	(100)	(100)	(100)	(100)
	KS	91.1	99.6	100	100	100	100	100
		(91.1)	(99.6)	(100)	(100)	(100)	(100)	(100)
LSi	waddR SP	46.4	75.1	88.9	96.9	98.4	98.0	98.0
		(31.0)	(70.0)	(88.1)	(96.8)	(98.4)	(98.0)	(98.0)
	waddR ASY	42.4	72.9	88.2	95.9	100	100	100
		(24.1)	(68.0)	(86.5)	(95.8)	(100)	(100)	(100)
	KS	32.9	61.9	82.9	90.1	100	100	100
		(13.8)	(53.3)	(75.1)	(90.1)	(100)	(100)	(100)
LSiSh	waddR SP	63.2	85.5	95.8	98.6	96.5	97.1	95.8
		(52.2)	(82.7)	(95.6)	(98.6)	(96.4)	(97.1)	(95.8)
	waddR ASY	99.7	100	100	100	100	100	100
		(99.7)	(100)	(100)	(100)	(100)	(100)	(100)
	KS	99.7	100	100	100	100	100	100
		(99.7)	(100)	(100)	(100)	(100)	(100)	(100)
N	waddR SP	4.9	5.9	6.7	6.0	5.9	5.5	6.4
		(0.0)	(0.0)	(0.0)	(0.0)	(0.0)	(0.0)	(0.0)
	waddR ASY	6.5	5.5	5.7	6.4	6.1	5.5	5.4
		(0.0)	(0.0)	(0.0)	(0.0)	(0.0)	(0.0)	(0.0)
	KS	3.7	3.5	4.6	5.7	6.5	5.6	5.1
		(0.0)	(0.1)	(0.1)	(0.0)	(0.0)	(0.0)	(0.0)

4 scRNA-seq simulation study to validate `waddR` variant A

4.1 Overview of approaches to identify differential distributions in scRNA-seq data

Table 6: Overview of differential expression analysis methods for scRNA-seq data and their corresponding features, with ✕ corresponding to "no" and ✓corresponding to "yes"

method	model/short description	permutation-based test	Bayesian method	tests for differences in location	tests for changes in distribution	explicitly tests for DPZ	able to account for replicates
`SCDE` [6]	Poisson and negative binomial model; z-scores (empirical p-values)	✕	✓	✓	✕	✕	✕
`BASiCS` [19]	integrated Bayesian hierarchical model; no p-value (fully Bayesian)	✕	✓	✓	✓	✕	✕
`MAST` [3]	logistic regression and generalized linear hurdle model; likelihood-ratio/Wald test	✕	✕	✓	✕	✓	✕
`BPSC` [21]	Beta-Poisson model; generalized linear model/Wald test	✕	✕	✓	✕	✕	✕
`scDD` [9]	logistic regression and conjugate Dirichlet process mixture; test using Bayes factor scores	✓	✓	✓	✓	✓	✕
`SigEMD` [22]	logistic regression and test using Earth Mover's distance	✓	✕	✓	✓	(✕)	✕
`DESingle` [12]	zero-inflated negative binomial model; likelihood-ratio test	✕	✕	✓	✓	✓	✕

4.2 Results

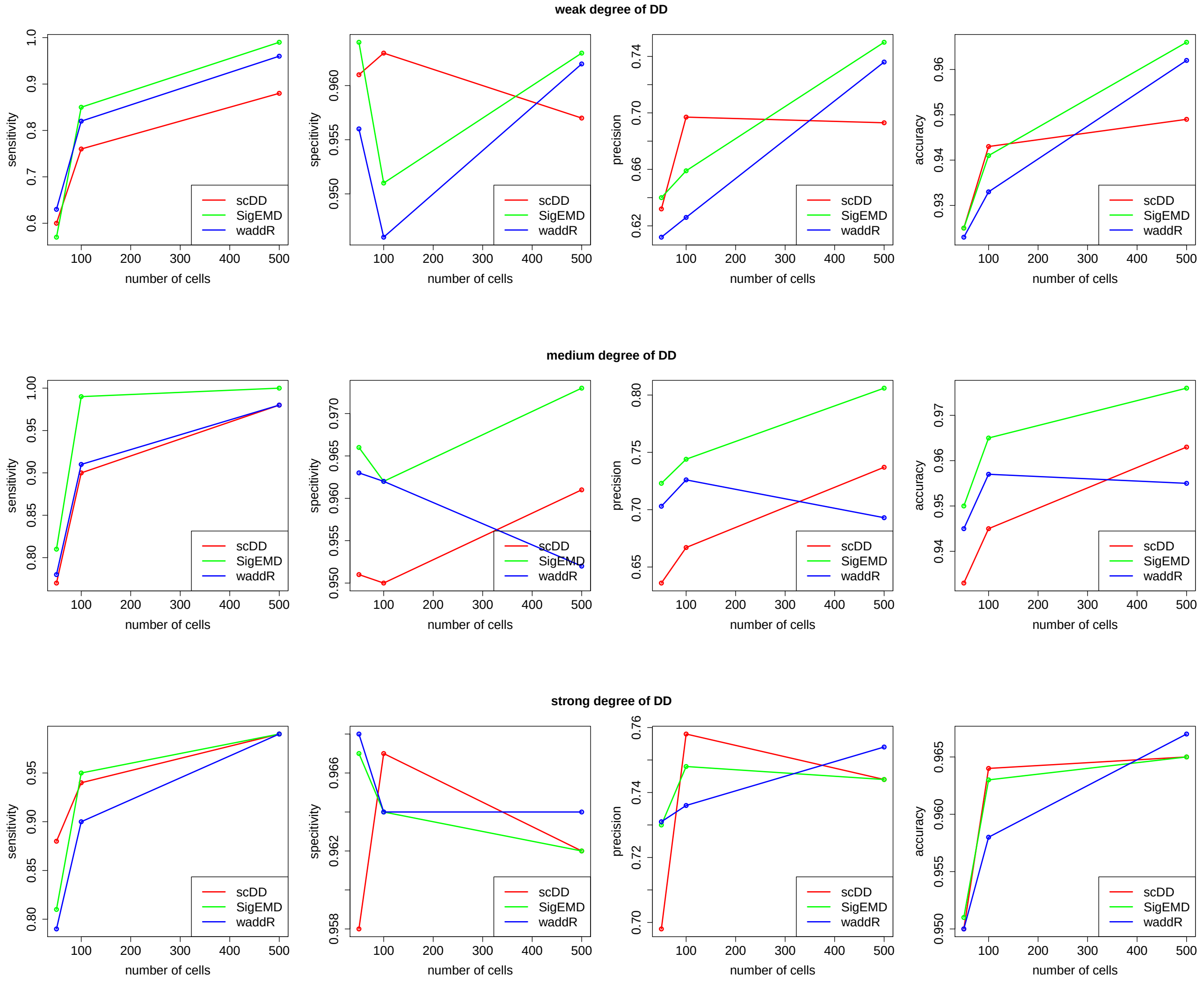

Figure 13: Sensitivity, specificity, precision and accuracy of the methods for a weak, medium and strong degree of DD, respectively, depending on the numbers of cells, for a p-value threshold of 0.05

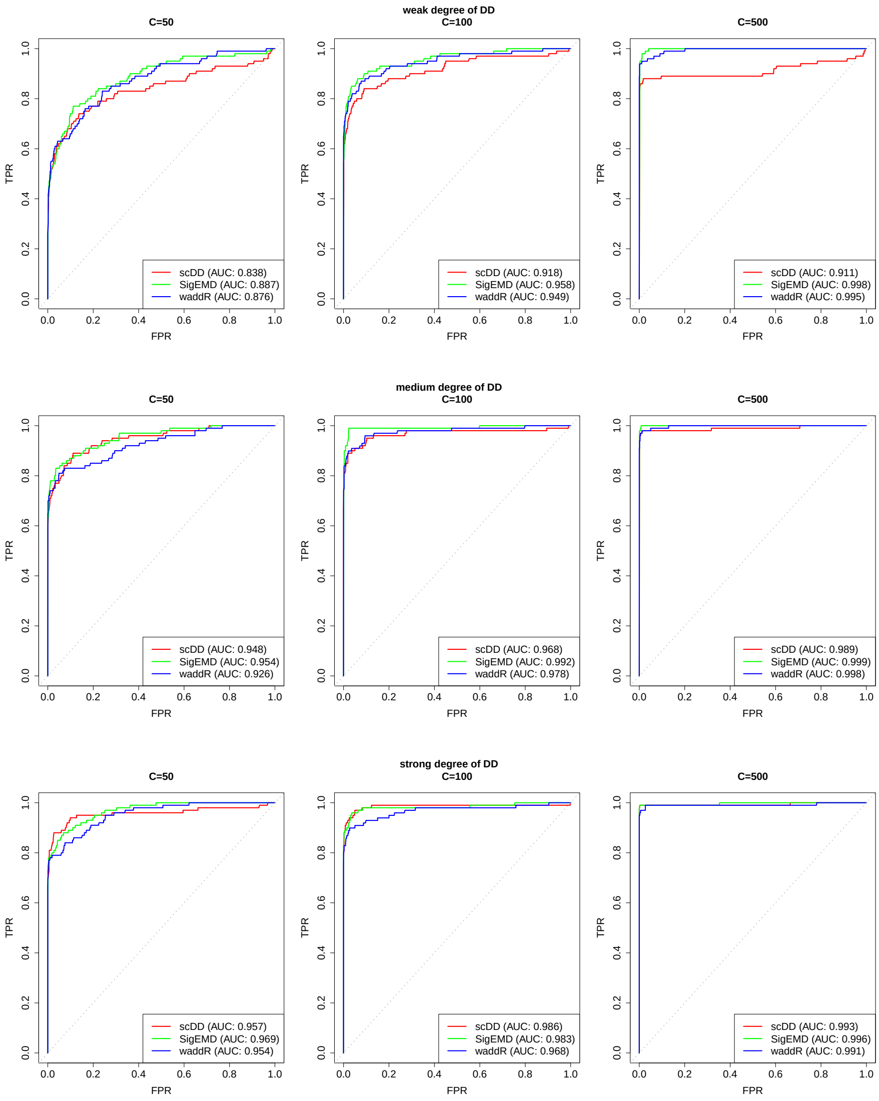

Figure 14: ROC curves with AUC values of the methods for a weak, medium and strong degree of DD, respectively, for different numbers of cells C

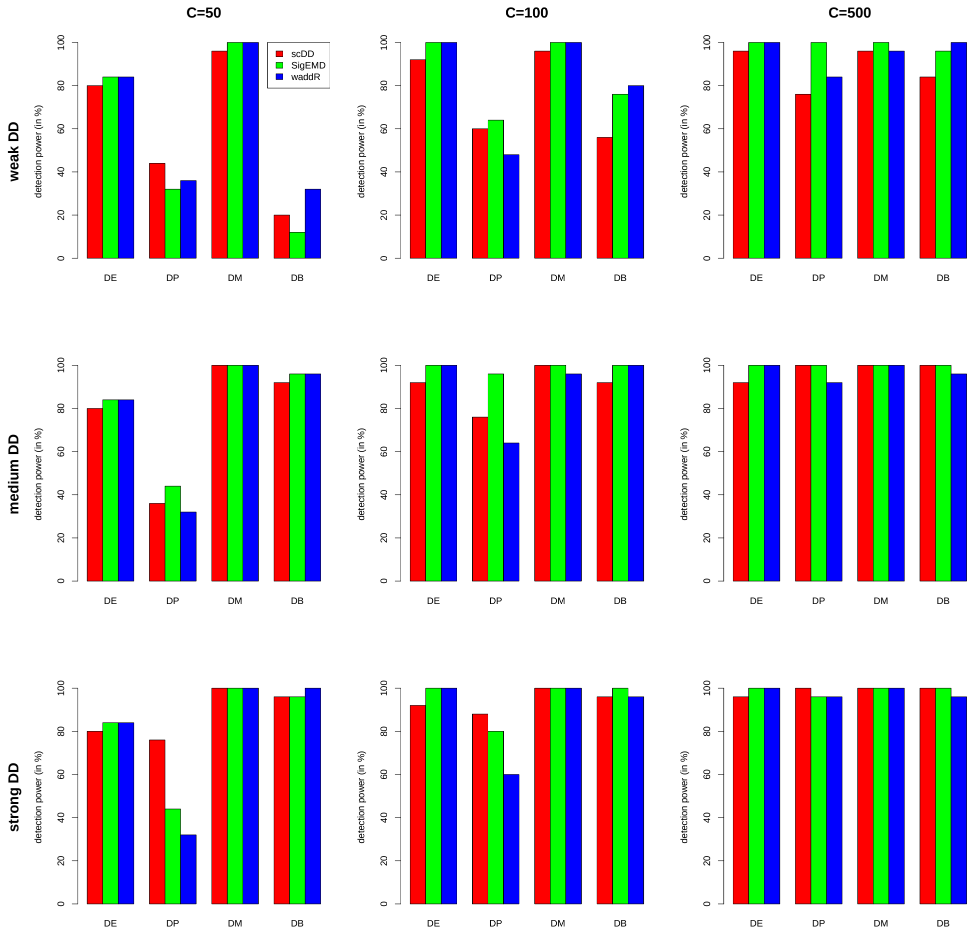

Figure 15: Detection powers of the methods for the different DD categories according to [9], depending on number of cells C and degree of DD

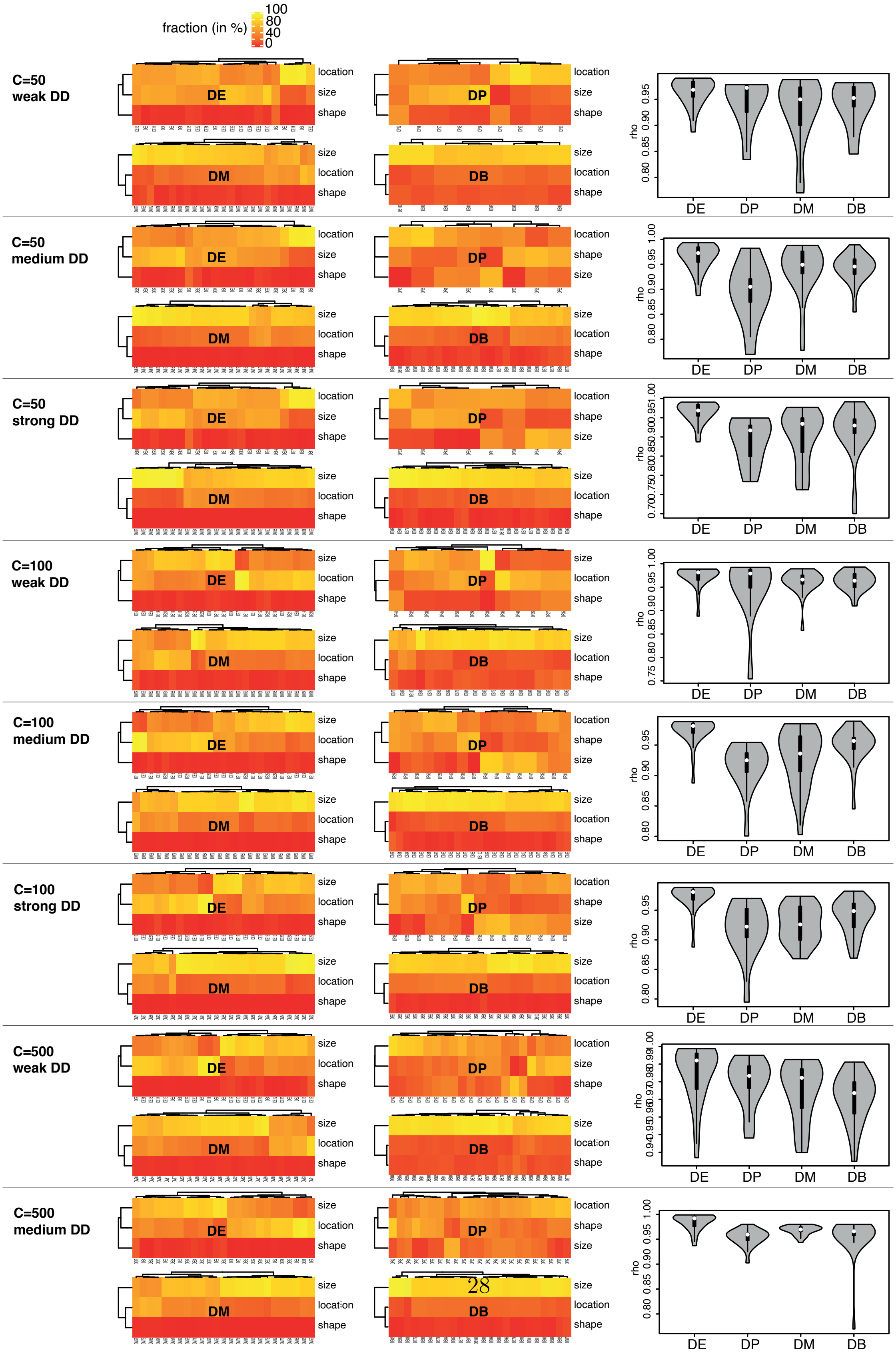

Figure 16: Heatmaps for the decomposition of the 2-Wasserstein distance and values of the correlation coefficients ρ for varying numbers of cells C and degrees of DD for the different DD categories, shown for those genes that have been detected as DD (non-zero DD p-value of ≤ 0.05).

5 Benchmarking for `waddR` variant B using real scRNA-seq data

To validate the variant B of our `waddR` approach for multiple replicates, we perform a benchmarking using the real Fluidigm C1 platform-based scRNA-seq data set by Tung et al. [18], oriented towards similar analyses as performed in [23].

We downloaded the pre-processed, filtered read count matrix (comprising 13106 endogenous genes and ERCC spike-ins) corresponding to the scRNA-seq experiment by Tung et al. [18] from `https://github.com/jdblischak/singleCellSeq`. The full data set consists of three Yoruba (YRI) induced pluripotent stem cell (iPSC) lines, with three replicates per individual. In our analyses here, we focus on a comparison between the two individuals NA19101 (201 cells; replicate 1: 80 cells, replicate 2: 70 cells, replicate 3: 51 cells) and NA19239 (221 cells; replicate 1: 74 cells, replicate 2: 68 cells, replicate 3: 79 cells). Thus, we have a 13106×422 read count matrix.

Tung et al. [18] also used each replicate to generate a matching bulk RNA-seq sample. For our analyses, we use the bulk RNA-seq samples of the two individuals to derive a reliable list of reference differentially expressed genes (DEGs) that we employ as the ground truth. Specifically, we take the 500 genes with the smallest p-values produced by `edgeR` [15], an established method for differential expression analysis for bulk RNA-seq data, as the gold standard in what follows, using `edgeR`'s default normalization and quasi-likelihood F test to check for differential expression.

For `waddR` variant B, we consider a gene to be differentially expressed (distributed) when the respective combined p-value obtained by Fisher's method (derived from the p-values for non-zero differential distributions and differential proportions of zero expression) is ≤ 0.05.

We also investigate the impact of the chosen normalization method on the performance of `waddR` variant B. To this end, we repeatedly perform our analyses based on the following different normalization strategies:

- the log normalization with scale factor 10^4 (feature counts for each cell are divided by the total counts for that cell and multiplied by the scale factor, then natural-log transformed) from the `Seurat` package [2] (LogNorm),
- the centered log ratio (CLR) normalization across features from the `Seurat` package (CLRFeatNorm),
- the CLR normalization across cells from the `Seurat` package (CLRCellsNorm),
- the relative counts normalization with scale factor 10^4 (feature counts for each cell are divided by the total counts for that cell and multiplied by the scale factor, no subsequent log transformation) from the `Seurat` package (RCNorm), and
- the deconvolution approach from the `scran` package [10] (DeconvNorm).

To evaluate `waddR` variant B, we consider

- sensitivity, specificity and accuracy as performance measures,
- the receiver operating characteristics (ROC) curve of true positive rate (TPR) plotted against false positive rate (FPR), along with the area under the ROC curve (AUC) value,

- the false discovery rate (FDR) curve showing the FDR among the top m discovered DEGs, and
- the ability to control type I errors.

To specifically check the ability of controlling type I errors, we generate a scenario in which no genuine DEGs are expected and hence all discovered DEGs are false positives. Specifically, we randomly assign each of the original 422 cells to one (out of two) individual and one (out of three) replicate, where the respective numbers of cells corresponding to the individuals and replicates equal those of the original data matrix (i.e., we randomly permute the column meta data of the original data matrix, which comprises the assignment to an individual and a replicate). Since the split is random, we on average expect no difference between the two considered groups (i.e., the two "newly configured individuals"). The null hypothesis of no differential expression should hold true for all the genes and thus, the nominal p-values obtained can be expected to be standard uniformly distributed. The comparison is performed on $n = 20$ random splits. For each split, we derive the observed proportion of declared DEGs using a combined p-value cut-off of 0.05. This proportion equals the type I error rate and should match the nominal p-value cut-off.
To also investigate the effect of the number of replicates, we perform all our analyses in two settings:

- the three-replicates setting (Supplement Section 5.1), in which all three available replicates for the two individuals NA 19101 and NA19239 are used for the analyses, and
- the two-replicates setting (Supplement Section 5.2), in which only replicates 1 and 2 for the two individuals NA 19101 and NA19239 are used for the analyses.

Finally, for the three-replicates setting, we additionally compare the performance of `waddR` variant B to that of the `scDD` [9] and `SigEMD` [22] reference approaches (Supplement Section 5.3).

Table 7: Three-replicates setting: Sensitivity, specificity and accuracy of `waddR` variant B for different normalization methods, based on a combined p-value cut-off at 0.05

	LogNorm	CLRFeatNorm	CLRCellsNorm	RCNorm	DeconvNorm
sensitivity	0.772	0.804	0.760	0.758	0.762
specificity	0.753	0.711	0.738	0.763	0.742
accuracy	0.753	0.714	0.739	0.763	0.743

5.1 Results for the three-replicates setting

In the three-replicates setting, `waddR` shows adequate ROC curves (Figure 17) with a reasonable balance between sensitivity and specificity (Table 7), as well as FDR curves (Figure 18), consistently across all normalization methods.
Moreover, `waddR` exhibits a good control of type I errors, with observed rates close to 0.05 (Figure 19) and produced p-value distributions as desired (Figure 20), consistently across all normalization methods.

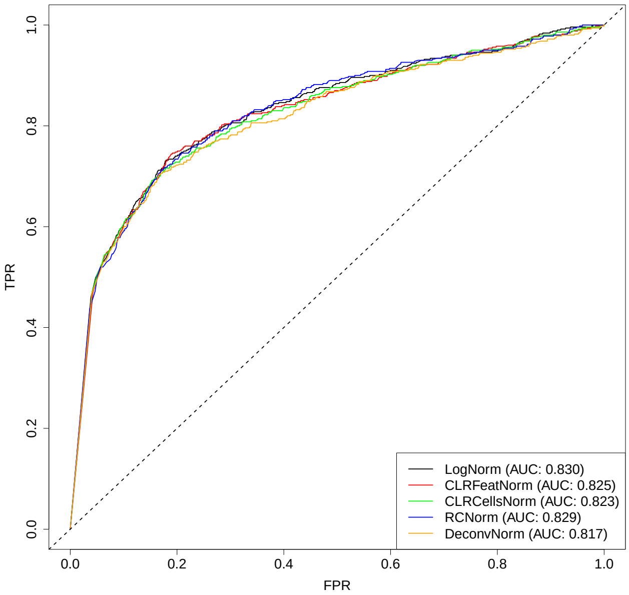

Figure 17: Three-replicates setting: ROC curves with corresponding AUC values of `waddR` variant B for different normalization methods

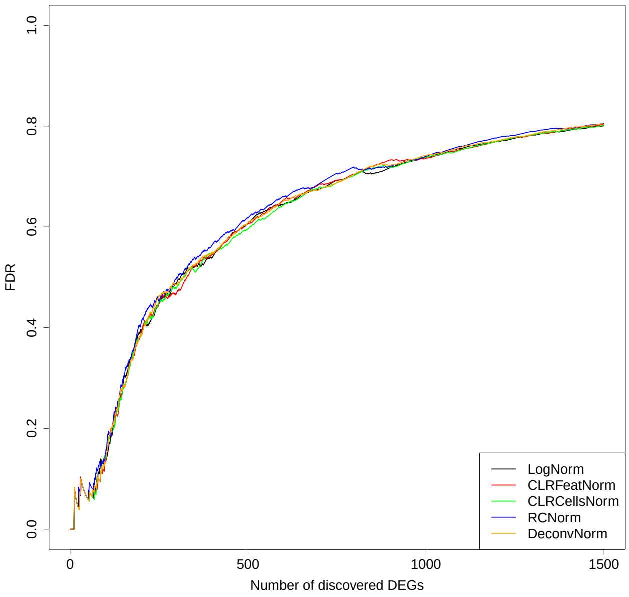

Figure 18: Three-replicates setting: FDR curves of `waddR` variant B for different normalization methods

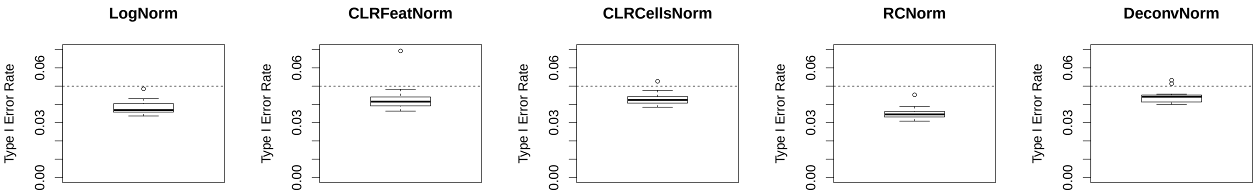

Figure 19: Three-replicates setting: Type I error rate boxplots by using a combined p-value cut-off of 0.05 on nominal p-values produced by `waddR` variant B for different normalization methods. Each box was generated based on the same $n = 20$ comparisons.

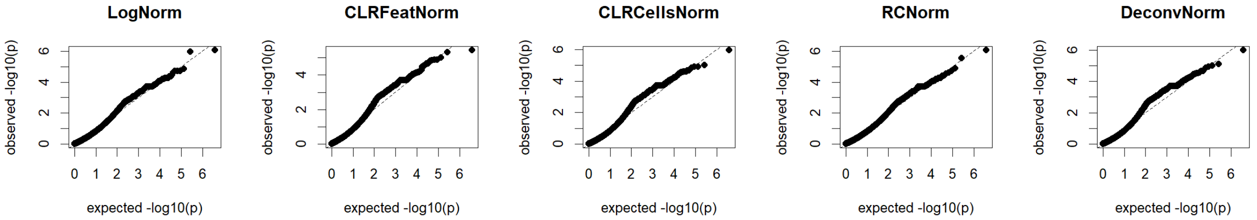

Figure 20: Three-replicates setting: Q-Q plots of nominal p-values p produced by `waddR` variant B for different normalization methods comparing the quantiles of their distribution with the standard uniform distribution

Table 8: Two-replicates setting: Sensitivity, specificity and accuracy of `waddR` variant B for different normalization methods, based on a combined p-value cut-off at 0.05

	LogNorm	CLRFeatNorm	CLRCellsNorm	RCNorm	DeconvNorm
sensitivity	0.582	0.600	0.594	0.582	0.600
specificity	0.835	0.827	0.831	0.838	0.831
accuracy	0.825	0.818	0.822	0.828	0.822

5.2 Results for the two-replicates setting

In the two-replicates setting, `waddR` meaningfully shows a bit weaker performance than in the three-replicates setting (Figures 21 and 22). Specifically, a bit lower AUC values can be observed, across all normalization methods. When considering two replicates, `waddR` appears to be overly conservative with low type I error rates (Figures 23 and 24) and a bit higher specificity, but a considerably lower sensitivity (Table 8) than in the three-replicates setting.
Our studies suggest that the performance of `waddR` variant B meaningfully increases with the number of replicates. This is to be expected, as the powers of the involved testing procedures, in particular the Wilcoxon rank sum test, are supposed to rise with increasing sample size induced by a higher number of replicates.
We have seen that in our benchmarking study, `waddR` performs somewhat okay for two replicates and convincingly well already for three replicates, and thus, we assume that `waddR` also yields reliable results in our real-experiment case study based on the data set in [20] comprising four replicates, for which no ground truth created via bulk RNA-seq is readily available.

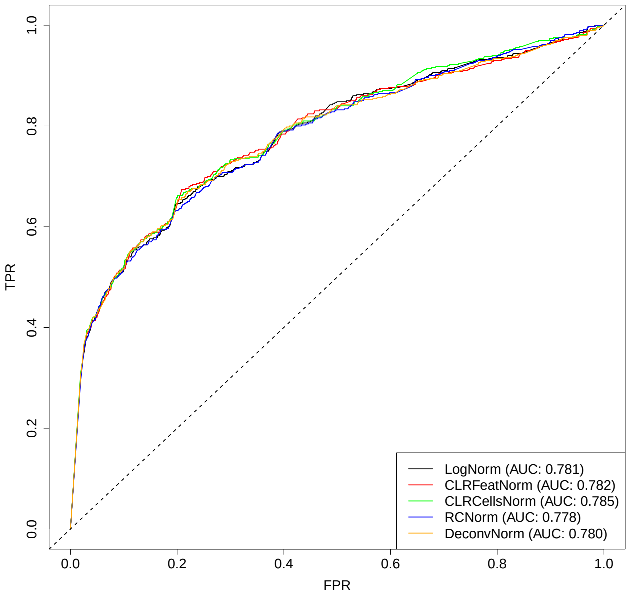

Figure 21: Two-replicates setting: ROC curves with corresponding AUC values of `waddR` variant B for different normalization methods

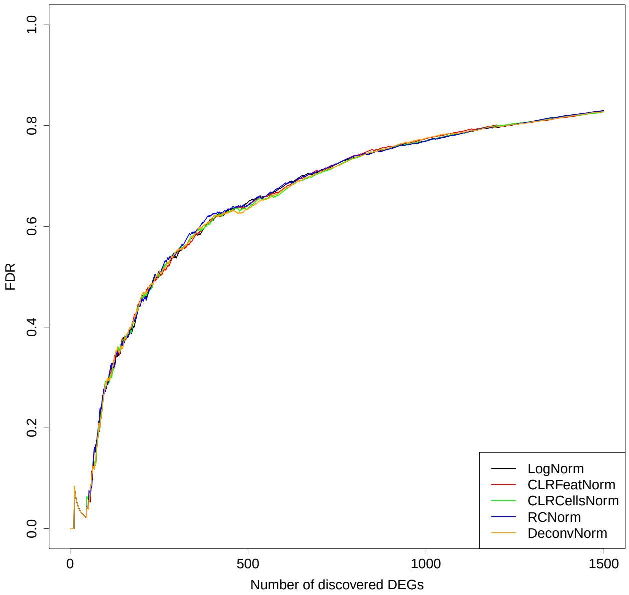

Figure 22: Two-replicates setting: FDR curves of `waddR` variant B for different normalization methods

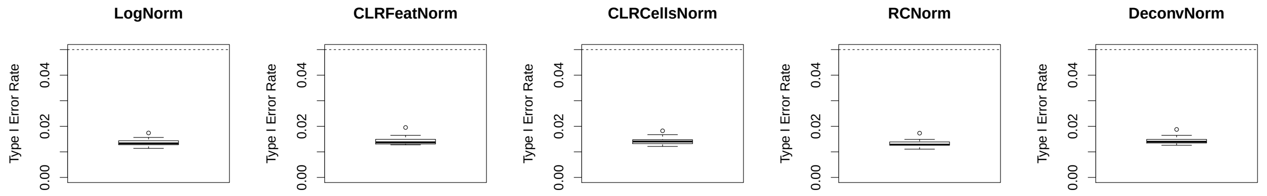

Figure 23: Two-replicates setting: Type I error rate boxplots by using a combined p-value cut-off of 0.05 on nominal p-values produced by `waddR` variant B for different normalization methods. Each box was generated based on the same $n = 20$ comparisons.

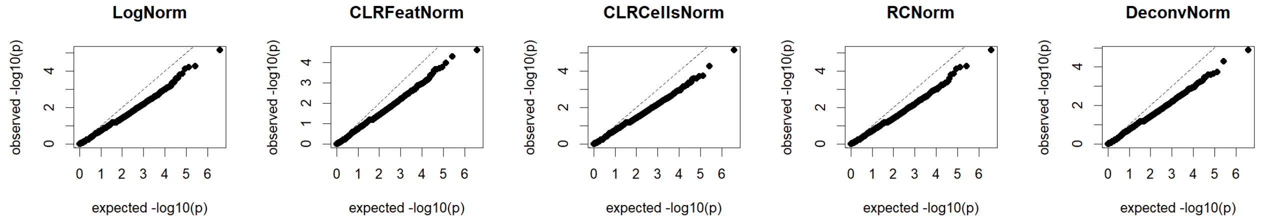

Figure 24: Two-replicates setting: Q-Q plots of nominal p-values p produced by `waddR` variant B for different normalization methods comparing the quantiles of their distribution with the standard uniform distribution

5.3 Comparison to reference methods

Finally, we compare the performance of `waddR` variant B to that of the reference methods `scDDD` [9] and `SigEMD` [22] in the three-replicates setting of our benchmarking study. We apply all considered approaches to the normalized data obtained by LogNorm. Since neither `scDD` nor `SigEMD` are designed to deal with replicates, we pool all replicates among an individual for these approaches to allow for comparison. Due to the long running times of `scDD` and `SigEMD`, we set the number of permutations to 100 in both methods.

`waddR` performs best in terms of ROC curves and the corresponding AUC values (Figure 25), in particular in the relevant range of $0 \leq \text{FPR} \leq 0.1$ (Figure 26). `waddR` also clearly outperforms the other methods regarding FDR curves (Figure 27).

All approaches show a good type I error control, with observed rates close to 0.05 (Figure 28), and reasonable p-value distributions (Figure 29), where `scDD` and `SigEMD` exhibit a slight overabundance of p-values around zero.

With respect to the specificity, accuracy and sensitivity metrics, all three approaches perform similarly, with `waddR` slightly outperforming the other two in specificity and accuracy (Table 9).

Overall, in addition to the benefit of drastically reduced computation times compared to `scDD` and `SigEMD`, `waddR` outperforms these reference approaches or shows a competitive performance with respect to the considered evaluation criteria.

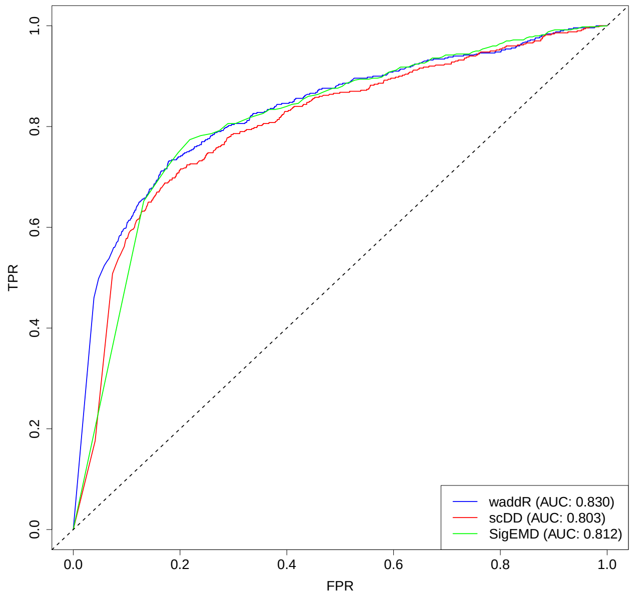

Figure 25: Three-replicates setting: ROC curves with corresponding AUC values of `waddR` variant B, `scDD` and `SigEMD`

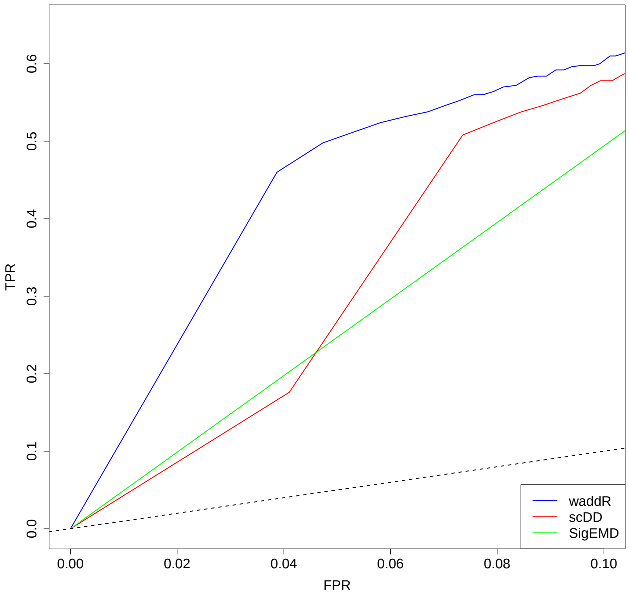

Figure 26: Three-replicates setting: Partial ROC curves of `waddR` variant B, `scDD` and `SigEMD` for the range of $0 \leq \text{FPR} \leq 0.1$

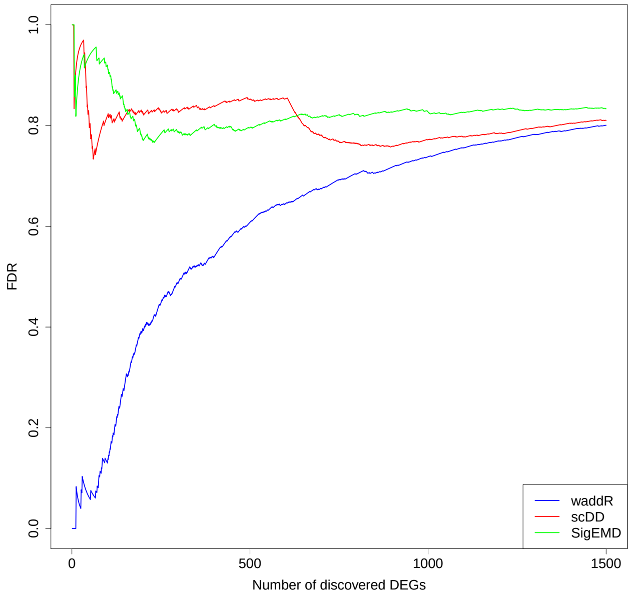

Figure 27: Three-replicates setting: FDR curves of `waddR` variant B, `scDD` and `SigEMD`

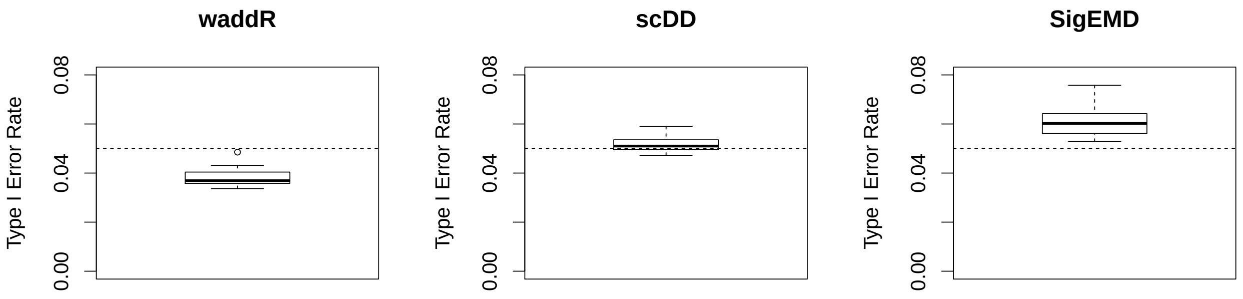

Figure 28: Three-replicates setting: Type I error rate boxplots by using a combined p-value cut-off of 0.05 on nominal p-values produced by `waddR` variant B, `scDD` and `SigEMD`. Each box was generated based on the same $n = 20$ comparisons.

Table 9: Three-replicates setting: Sensitivity, specificity and accuracy of `waddR` variant B, `scDD` and `SigEMD`, based on a combined p-value cut-off at 0.05

	`waddR`	`scDD`	`SigEMD`
sensitivity	0.772	0.778	0.786
specificity	0.753	0.712	0.741
accuracy	0.753	0.714	0.743

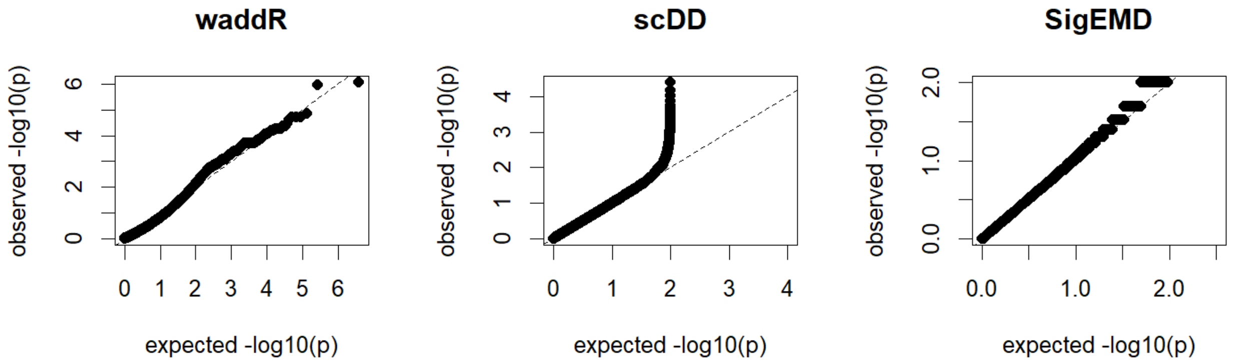

Figure 29: Three-replicates setting: Q-Q plots of nominal p-values p produced by `waddR` variant B, `scDD` and `SigEMD`, comparing the quantiles of their distribution with the standard uniform distribution

References

[1] Y. Benjamini and Y. Hochberg. Controlling the false discovery rate: a practical and powerful approach to multiple testing. *Journal of the Royal Statistical Society, Series B*, 57:289–300, 1995.

[2] A. Butler, P. Hoffman, P. Smibert, E. Papalexi, and R. Satija. Integrating single-cell transcriptomic data across different conditions, technologies, and species. *Nature Biotechnology*, 36:411–420, 2018.

[3] G. Finak, A. McDavid, M. Yajima, J. Deng, V. Gersuk, A. K. Shalek, C. K. Slichter, H. W. Miller, M. J. McElrath, M. Prlic, P. S. Linsley, and R. Gottardo. MAST: a flexible statistical framework for assessing transcriptional changes and characterizing heterogeneity in single-cell RNA sequencing data. *Genome Biology*, 16:278, 2015.

[4] S. Graf and H. Luschgy. *Foundations of Quantization for Probability Distributions.* Springer, Berlin, Heidelberg, 2000.

[5] A. Irpino and R. Verde. Basic statistics for distributional symbolic variables: a new metric-based approach. *Advances in Data Analysis and Classification*, 9:143–175, 2015.

[6] P. V. Kharchenko, L. Silberstein, and D. T. Scadden. Bayesian approach to single-cell differential expression analysis. *Nature Methods*, 11:740–742, 2014.

[7] T. A. Knijnenburg, L. F. A. Wessels, M. J. T. Reinders, and I. Shmulevich. Fewer permutations, more accurate p-values. *Bioinformatics*, 25:i161–i168, 2009.

[8] C. Kooperberg and C. J. Stone. Logspline density estimation for censored data. *Journal of Computational and Graphical Statistics*, 1:301–328, 1992.

[9] K. D. Korthauer, L.-F. Chu, M. A. Newton, Y. Li, J. Thomson, R. Stewart, and C. Kendziorski. A statistical approach for identifying differential distributions in single-cell RNA-seq experiments. *Genome Biology*, 17:222, 2016.

[10] A. T. L. Lun, K. Bach, and J. C. Marioni. Pooling across cells to normalize single-cell RNA sequencing data with many zero counts. *Genome Biology*, 17:75, 2016.

[11] Y. Matsui, M. Mizuta, S. Ito, S. Miyano, and T. Shimamura. D^3M: detection of differential distributions of methylation levels. *Bioinformatics*, 32:2248–2255, 2016.

[12] Z. Miao, K. Deng, X. Wang, and X. Zhang. DEsingle for detecting three types of differential expression in single-cell RNA-seq data. *Bioinformatics*, 34:3223–3224, 2018.

[13] B. Phipson and G. K. Smyth. Permutation p-values should never be zero: calculating exact p-values when permutations are randomly drawn. *Statistical Applications in Genetics and Molecular Biology*, 9:39, 2010.

[14] A. Ramdas, N. García Trillos, and M. Cuturi. On Wasserstein two-sample testing and related families of nonparametric tests. *Entropy*, 19:47, 2017.

[15] M. D. Robinson, D. J. McCarthy, and G. K. Smyth. edgeR: a Bioconductor package for differential expression analysis of digital gene expression data. *Bioinformatics*, 26:139–140, 2010.

[16] L. Rüschendorf. Wasserstein metric. In M. Hazewinkel, editor, *Encyclopaedia of Mathematics*. Springer, New York, 2001.

[17] L. Tolmatz. On the distribution of the square integral of the Brownian bridge. *The Annals of Probability*, 30:253–269, 2002.

[18] P.-Y. Tung, J. D. Blischak, C. J. Hsiao, D. A. Knowles, J. E. Burnett, J. K. Pritchard, and Y. Gilad. Batch effects and the effective design of single-cell gene expression studies. *Scientific Reports*, 7:39921, 2017.

[19] C. A. Vallejos, J. C. Marioni, and S. Richardson. BASiCS: Bayesian analysis of single-cell sequencing data. *PLoS Computational Biology*, 11:e1004333, 2015.

[20] R. Vento-Tormo, M. Efremova, M. Y. Turco, R. A. Botting, K. B. Meyer, J. Park, E. Stephenson, R. P. Payne, A. Goncalves, A. Zou, J. Henriksson, L. Wood, S. Lisgo, A. Filby, G. J. Wright, M. J. T. Stubbington, M. Haniffa, A. Moffett, and S. A. Teichmann. Reconstructing the human first trimester fetal-maternal interface using single cell transcriptomics. *Nature*, 563:347–353, 2018.

[21] T. N. Vu, Q. F. Wills, K. R. Kalari, N. Niu, L. Wang, M. Rantalainen, and Y. Pawitan. Beta-Poisson model for single-cell RNA seq data analyses. *Bioinformatics*, 32:2128–2135, 2016.

[22] T. Wang and S. Nabavi. SigEMD: a powerful method for differential gene expression analysis in single-cell RNA sequencing data. *Methods*, 145:25–32, 2018.

[23] C. Ye, T. P. Speed, and A. Salim. DECENT: differential expression with capture efficiency adjustmeNT for single-cell RNA-seq data. *Bioinformatics*, 35:5155–5162, 2019.
