# Supplementary material for: Fast identification of differential distributions in single-cell RNA-sequencing data with waddR
Source: Bioinformatics. 2021 Apr 1;37(19):3204–11. doi: 10.1093/bioinformatics/btab226 (PMC8504634; doi:10.1093/bioinformatics/btab226)
Supplement: btab226_Supplementary_Data [file btab226_supplementary_data.zip › Supplement_Revision2/TypeIError2Repl.pdf]

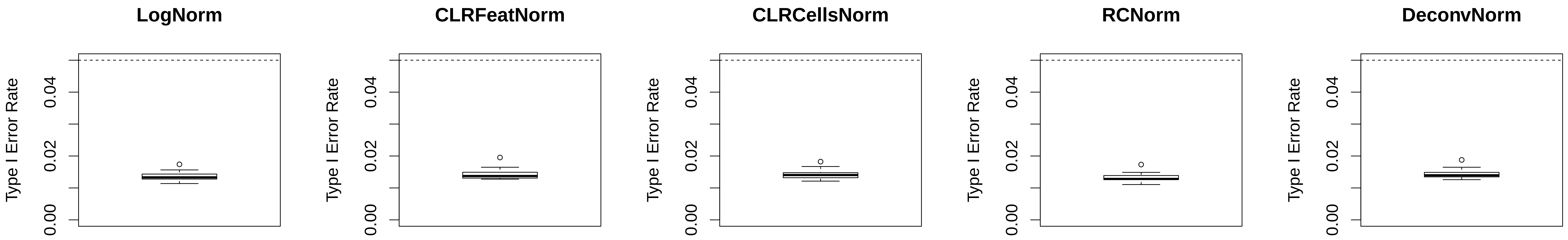
LogNorm
CLRFeatNorm
CLRCellsNorm
RCNorm
DeconvNorm
Type I Error Rate
0.00
0.02
0.04
