# Supplementary figures and images for: Fast identification of differential distributions in single-cell RNA-sequencing data with waddR
Source: Bioinformatics. 2021 Apr 1;37(19):3204–11. doi: 10.1093/bioinformatics/btab226 (PMC8504634; doi:10.1093/bioinformatics/btab226)

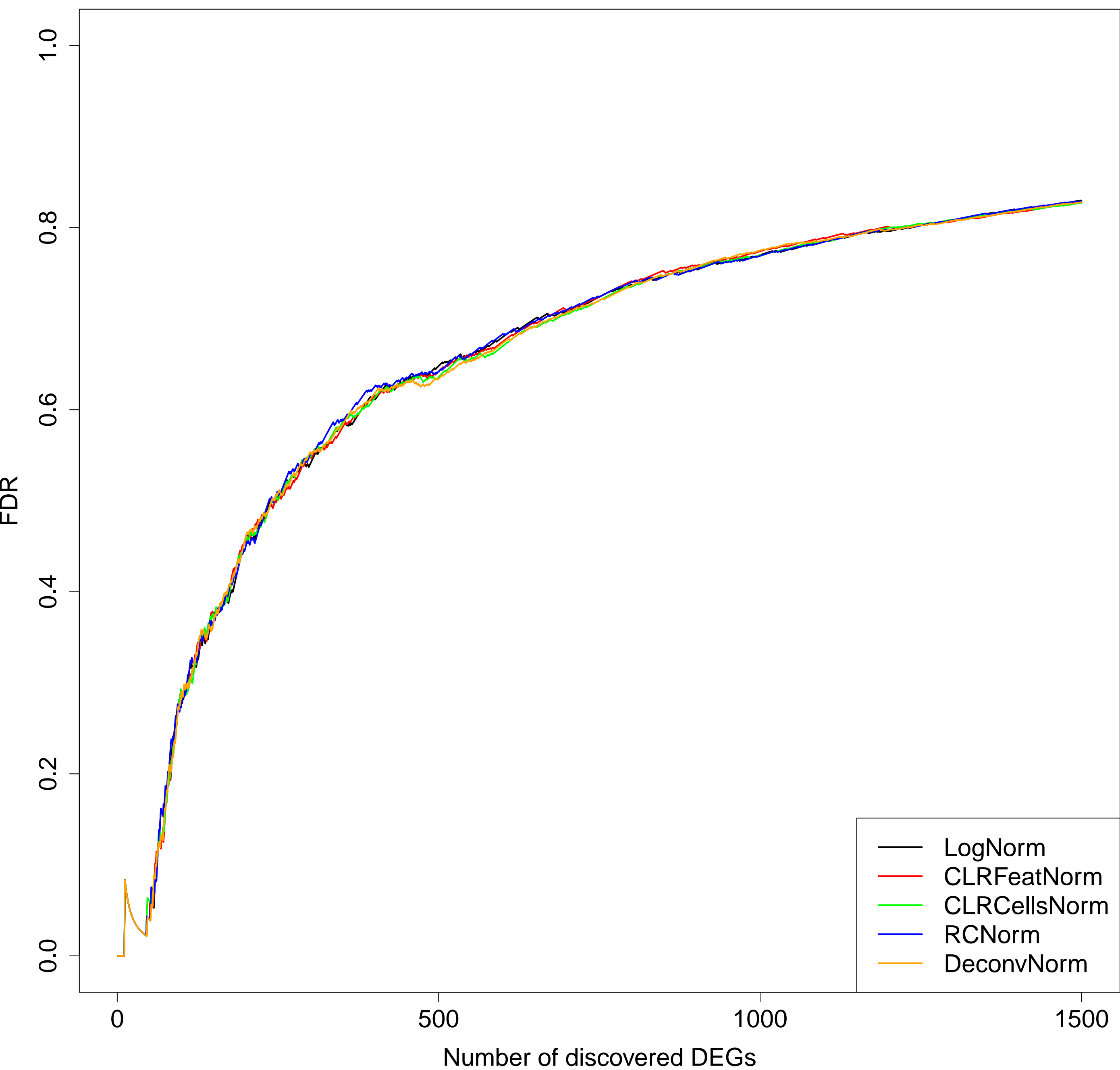

Supplement: btab226_Supplementary_Data [file btab226_supplementary_data.zip › Supplement_Revision2/FDRCurves2Repl.pdf]

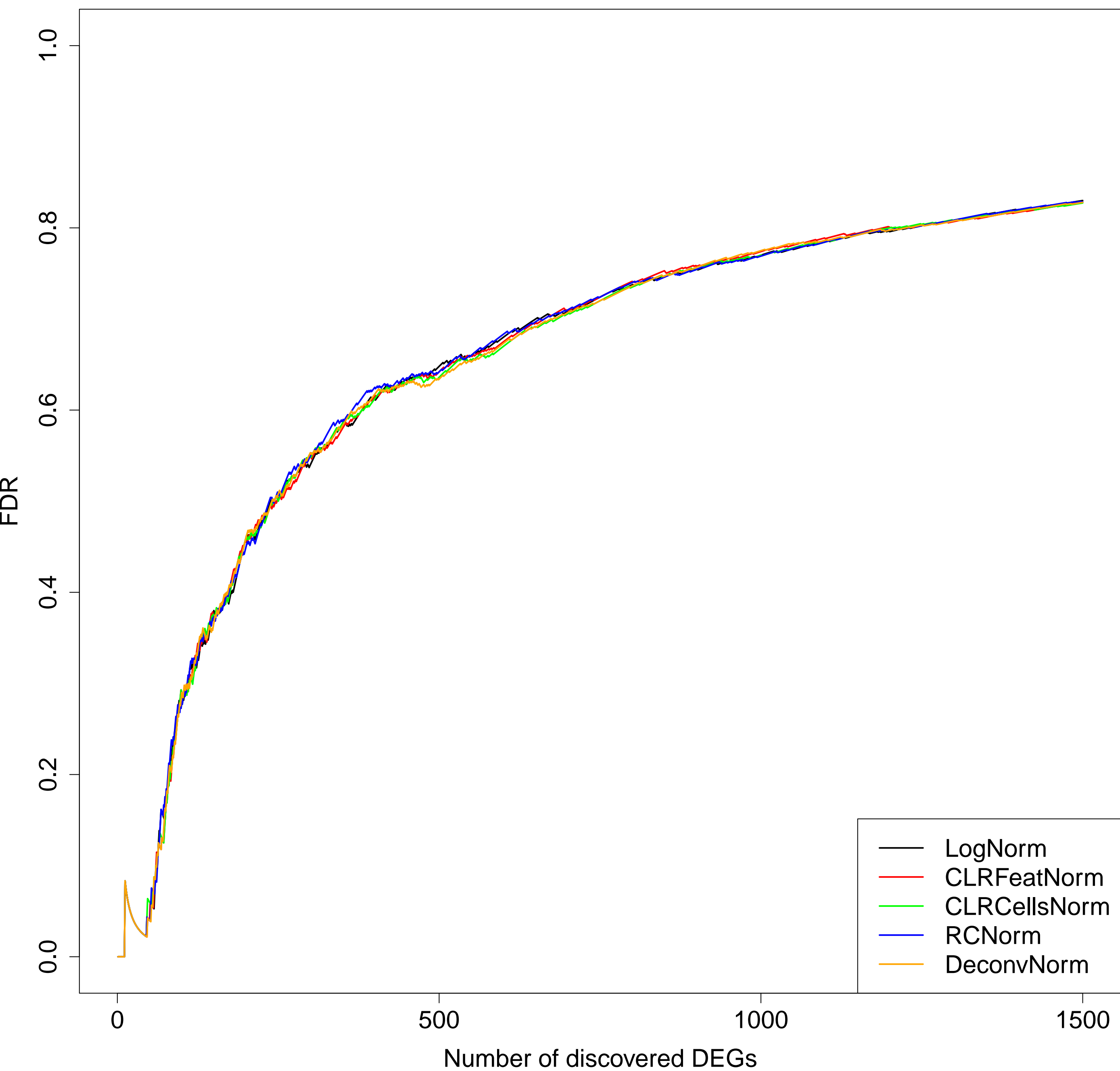

Supplement: btab226_Supplementary_Data [file btab226_supplementary_data.zip › Supplement_Revision2/FDRCurves2Repl_unadjp.pdf]

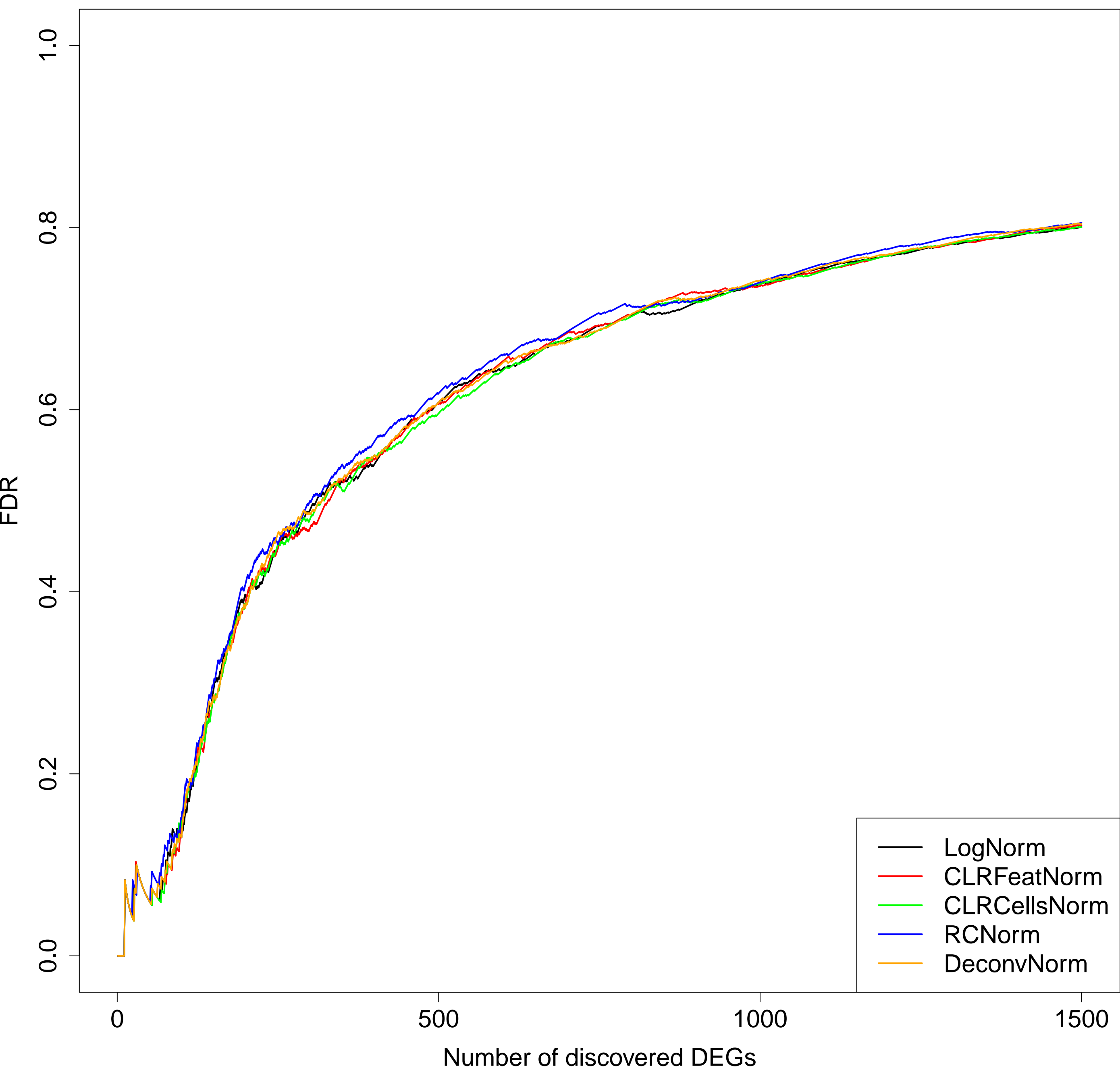

Supplement: btab226_Supplementary_Data [file btab226_supplementary_data.zip › Supplement_Revision2/FDRCurves3Repl.pdf]

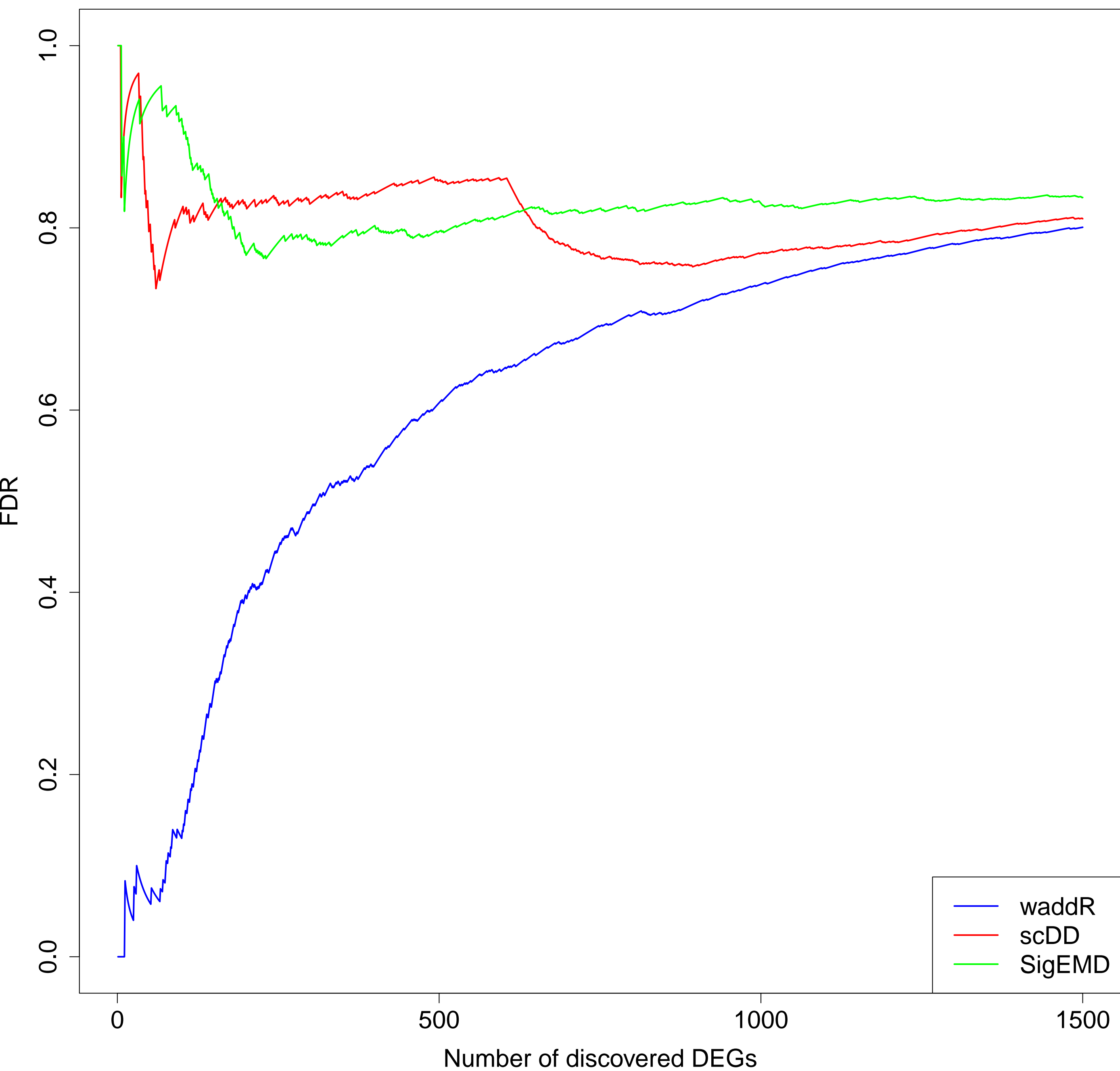

Supplement: btab226_Supplementary_Data [file btab226_supplementary_data.zip › Supplement_Revision2/FDRCurves3Repl_RefMeth.pdf]

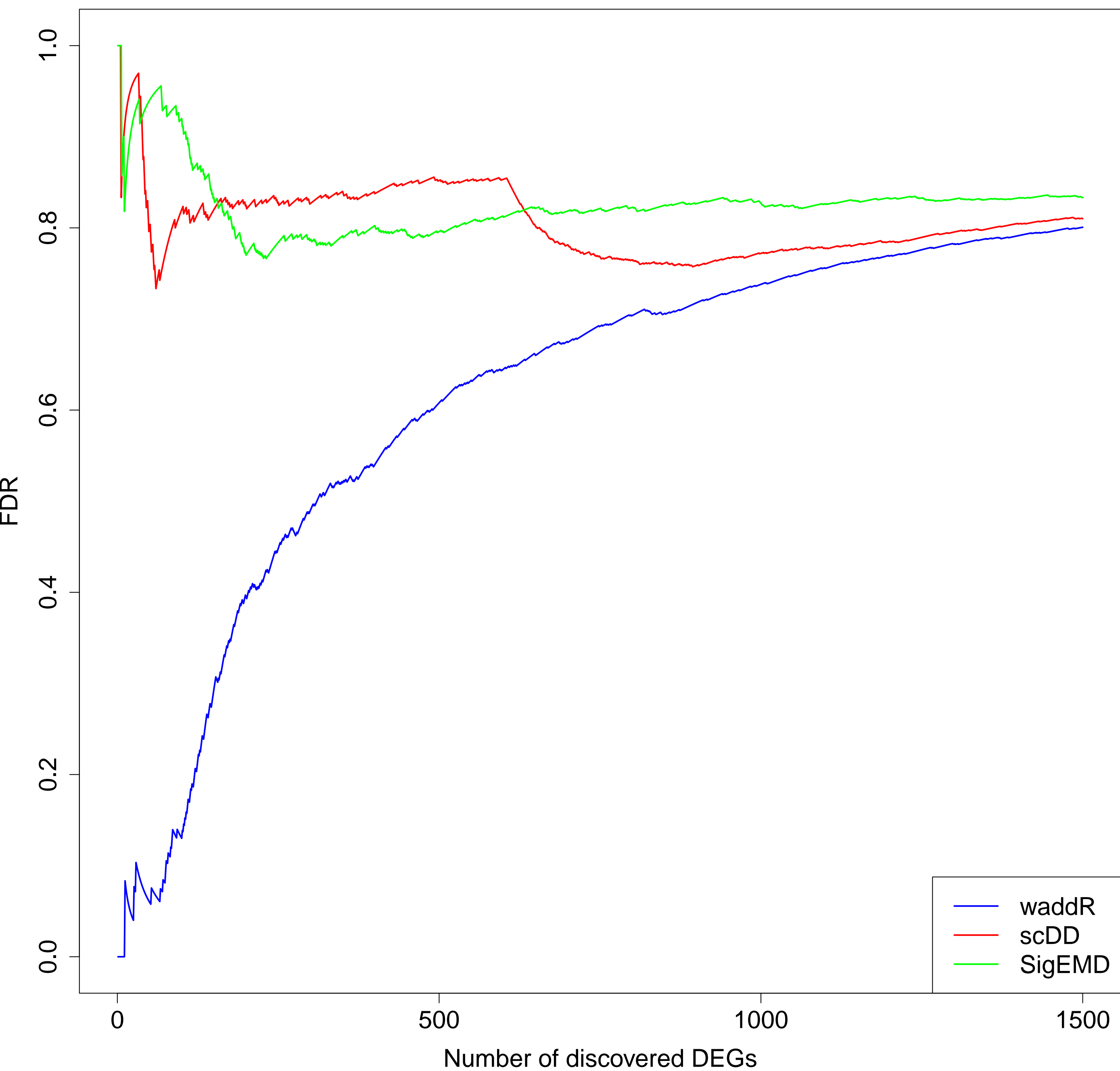

Supplement: btab226_Supplementary_Data [file btab226_supplementary_data.zip › Supplement_Revision2/FDRCurves3Repl_RefMeth_unadjp.pdf]

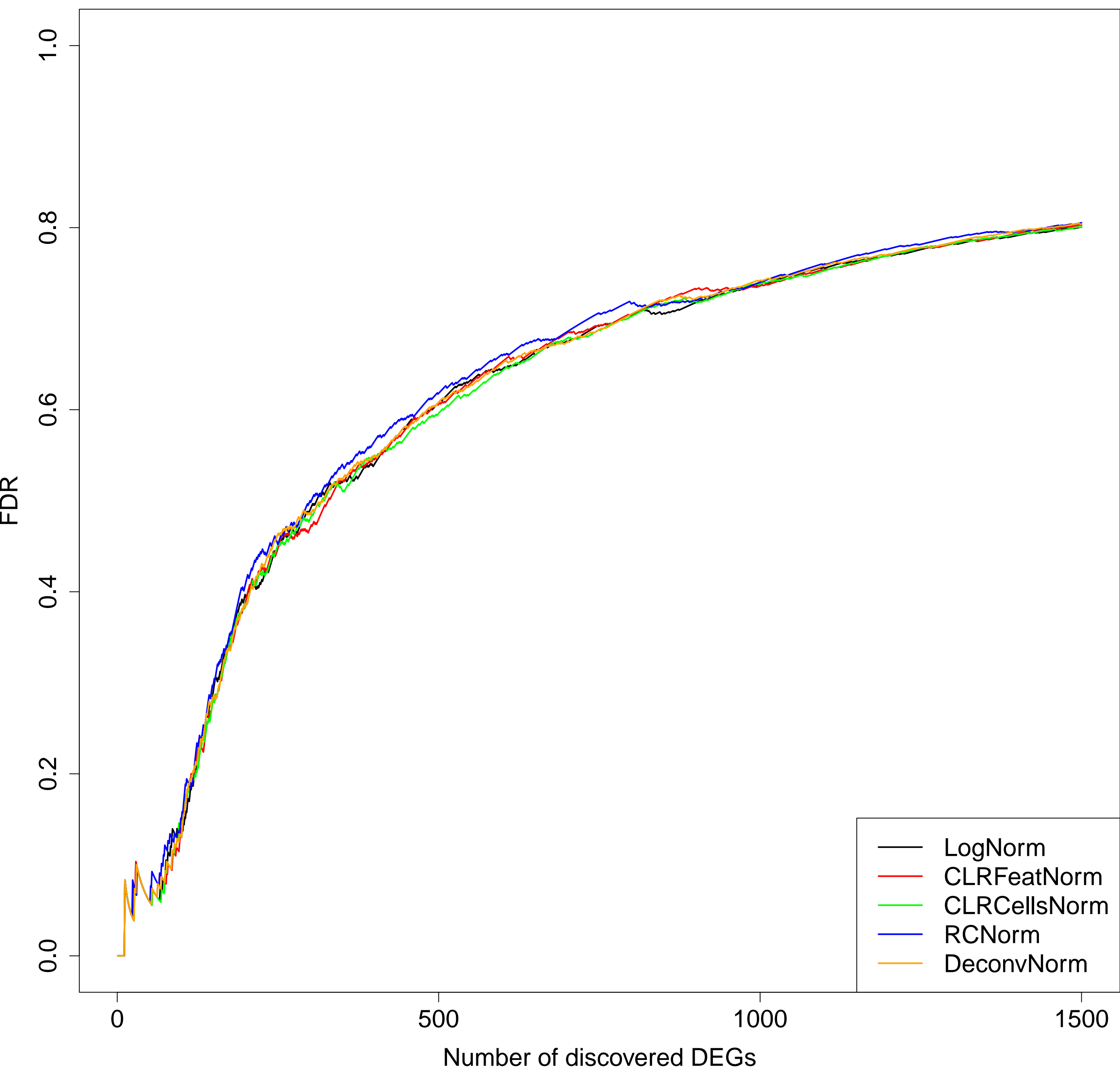

Supplement: btab226_Supplementary_Data [file btab226_supplementary_data.zip › Supplement_Revision2/FDRCurves3Repl_unadjp.pdf]

**(A) Differential location**

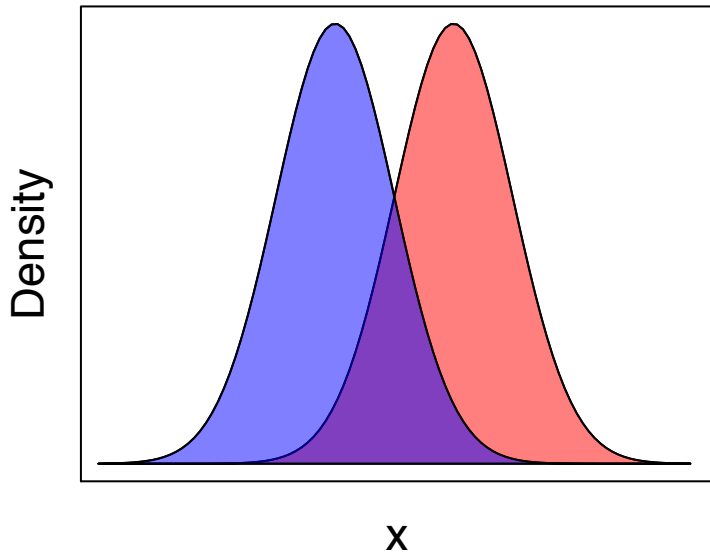

**(B) Differential size**

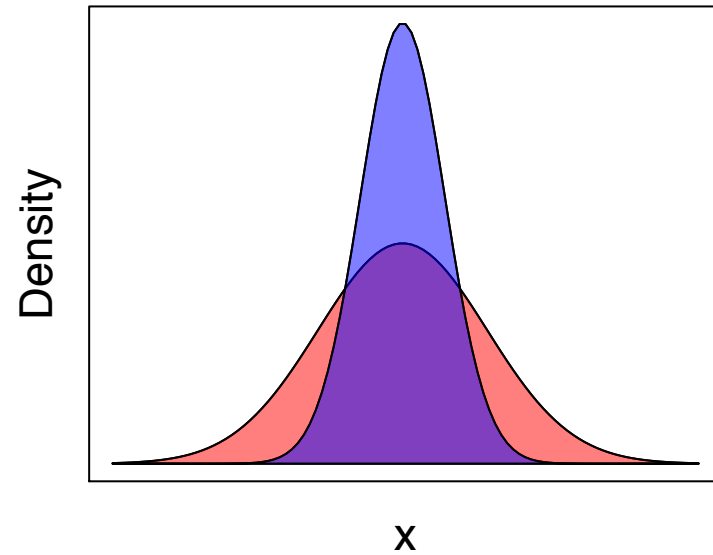

**(C) Differential shape**

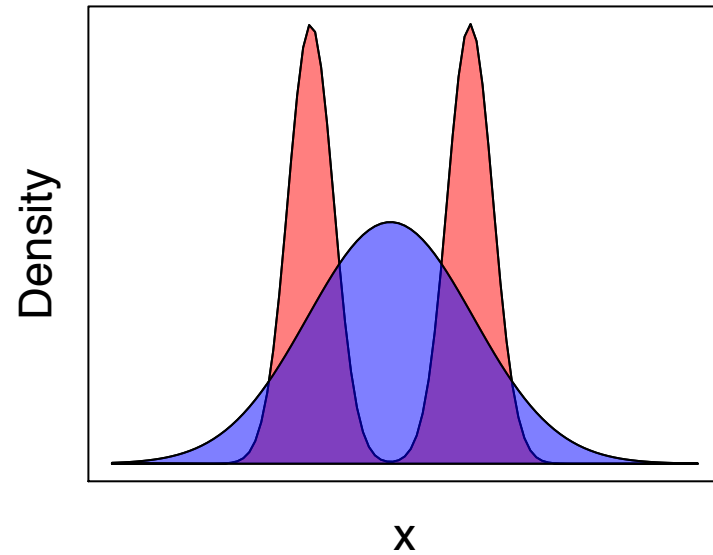

Supplement: btab226_Supplementary_Data [file btab226_supplementary_data.zip › Supplement_Revision2/Fig1.pdf]

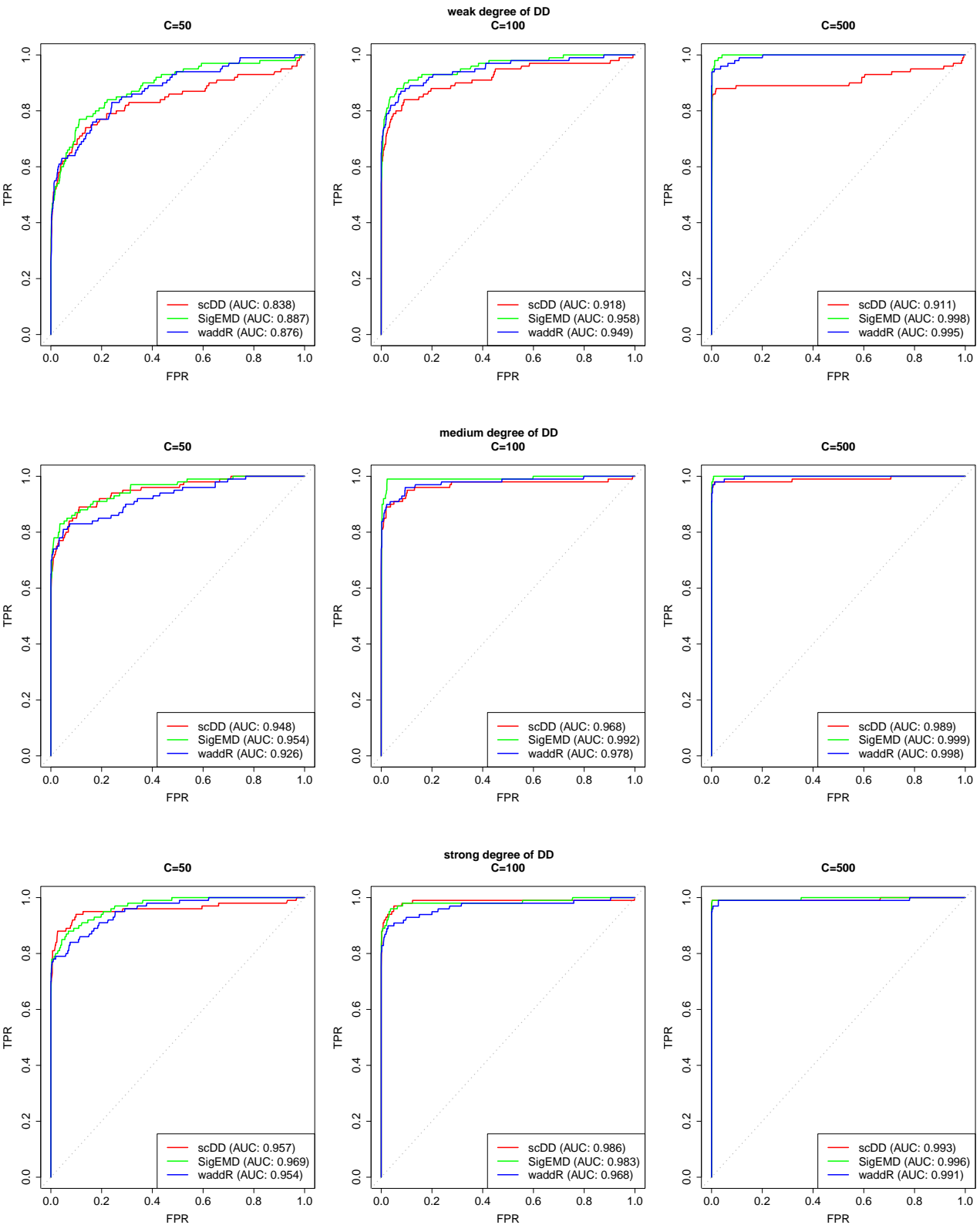

Supplement: btab226_Supplementary_Data [file btab226_supplementary_data.zip › Supplement_Revision2/Fig10.pdf]

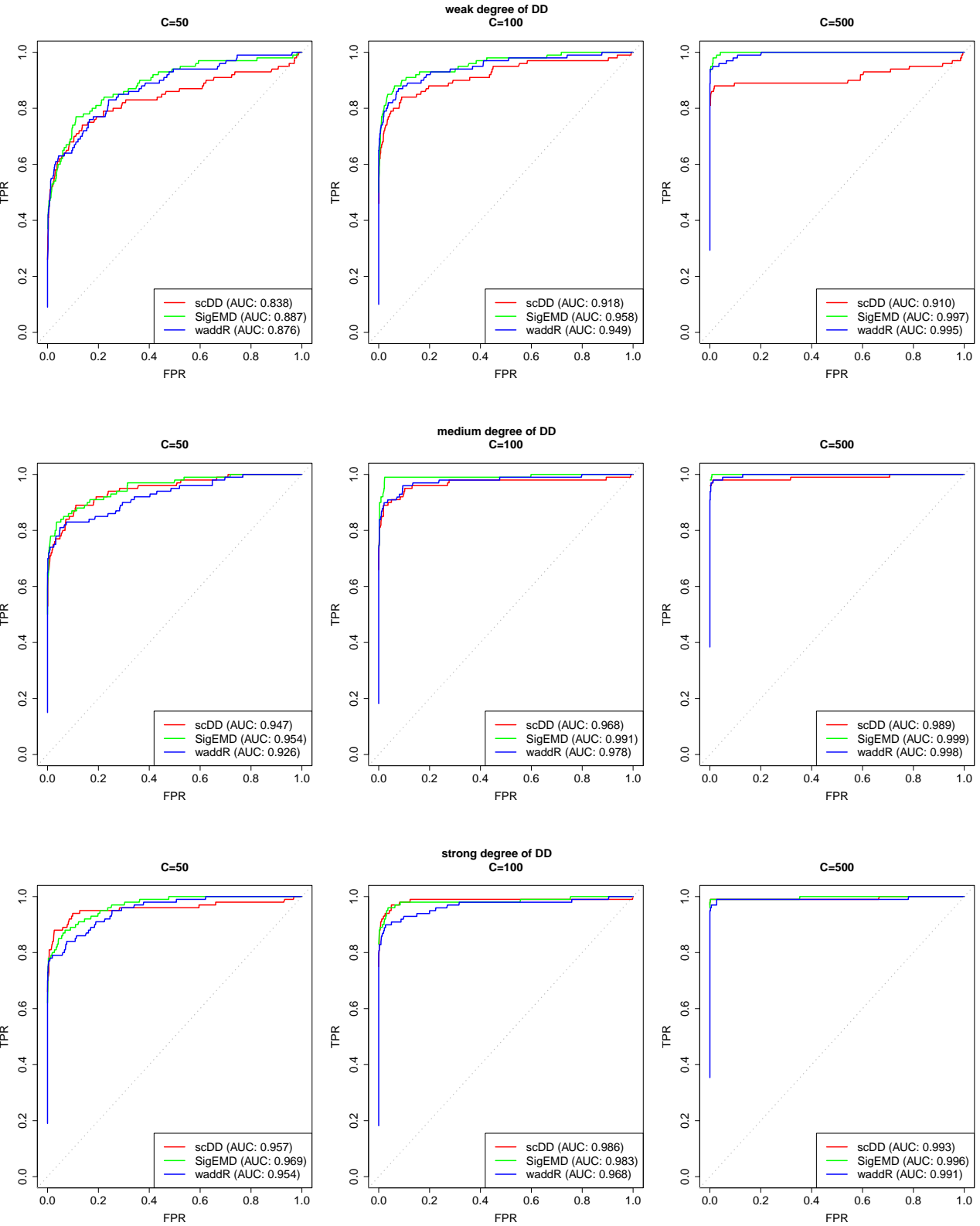

Supplement: btab226_Supplementary_Data [file btab226_supplementary_data.zip › Supplement_Revision2/Fig10_old.pdf]

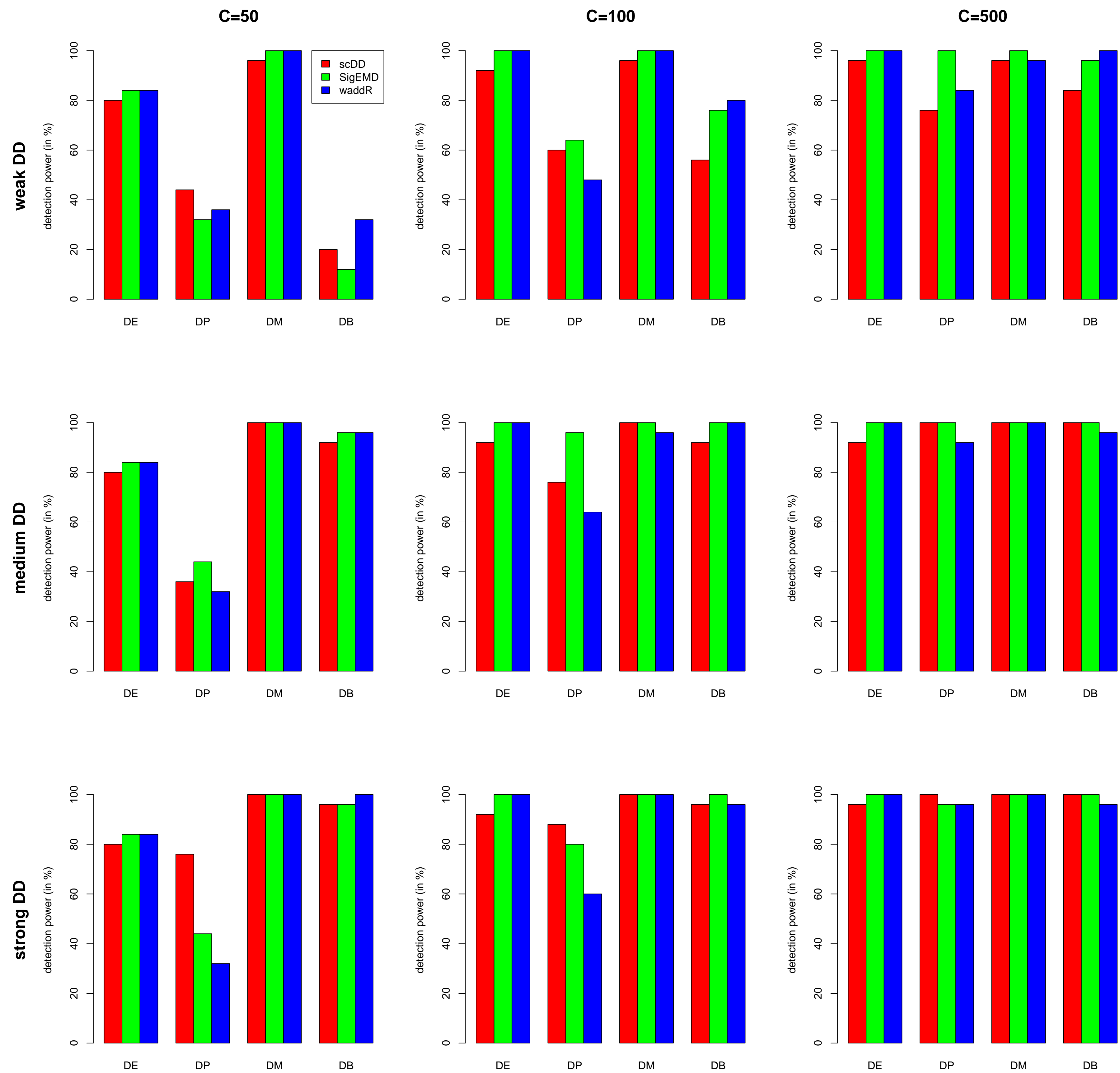

Supplement: btab226_Supplementary_Data [file btab226_supplementary_data.zip › Supplement_Revision2/Fig11.pdf]

## C=500, strong DD: Category DE

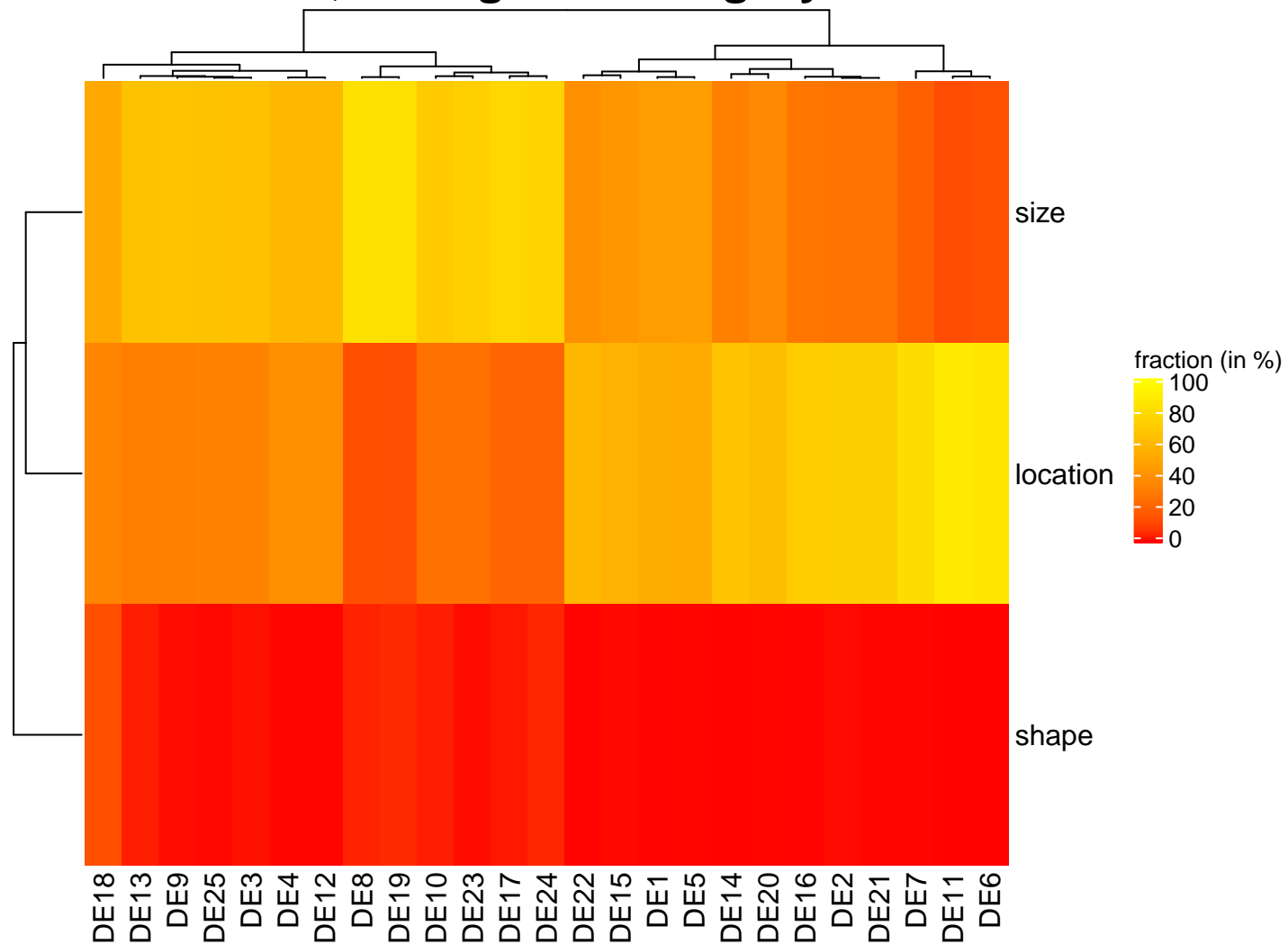

## C=500, strong DD: Category DP

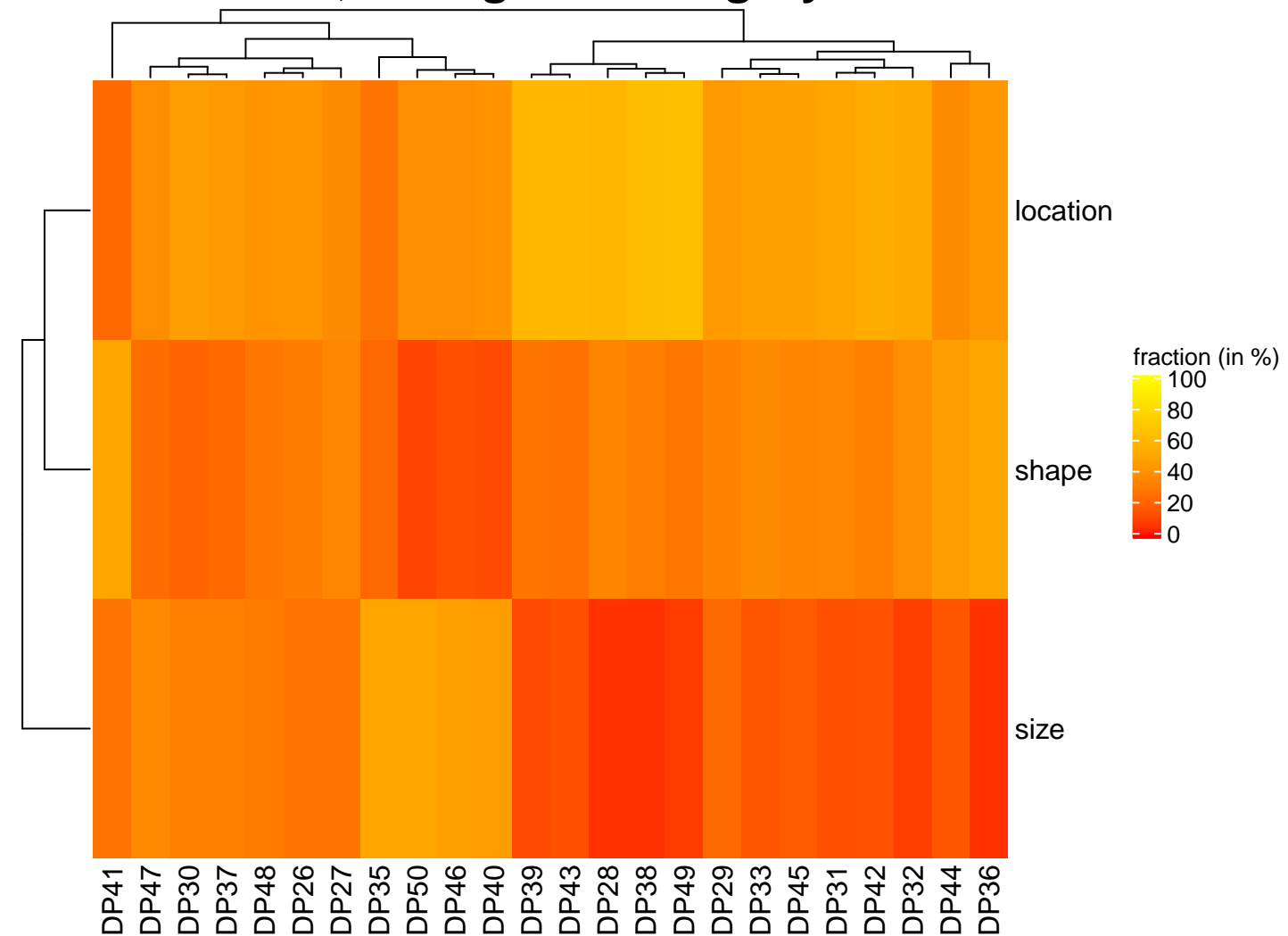

## C=500, strong DD: Category DM

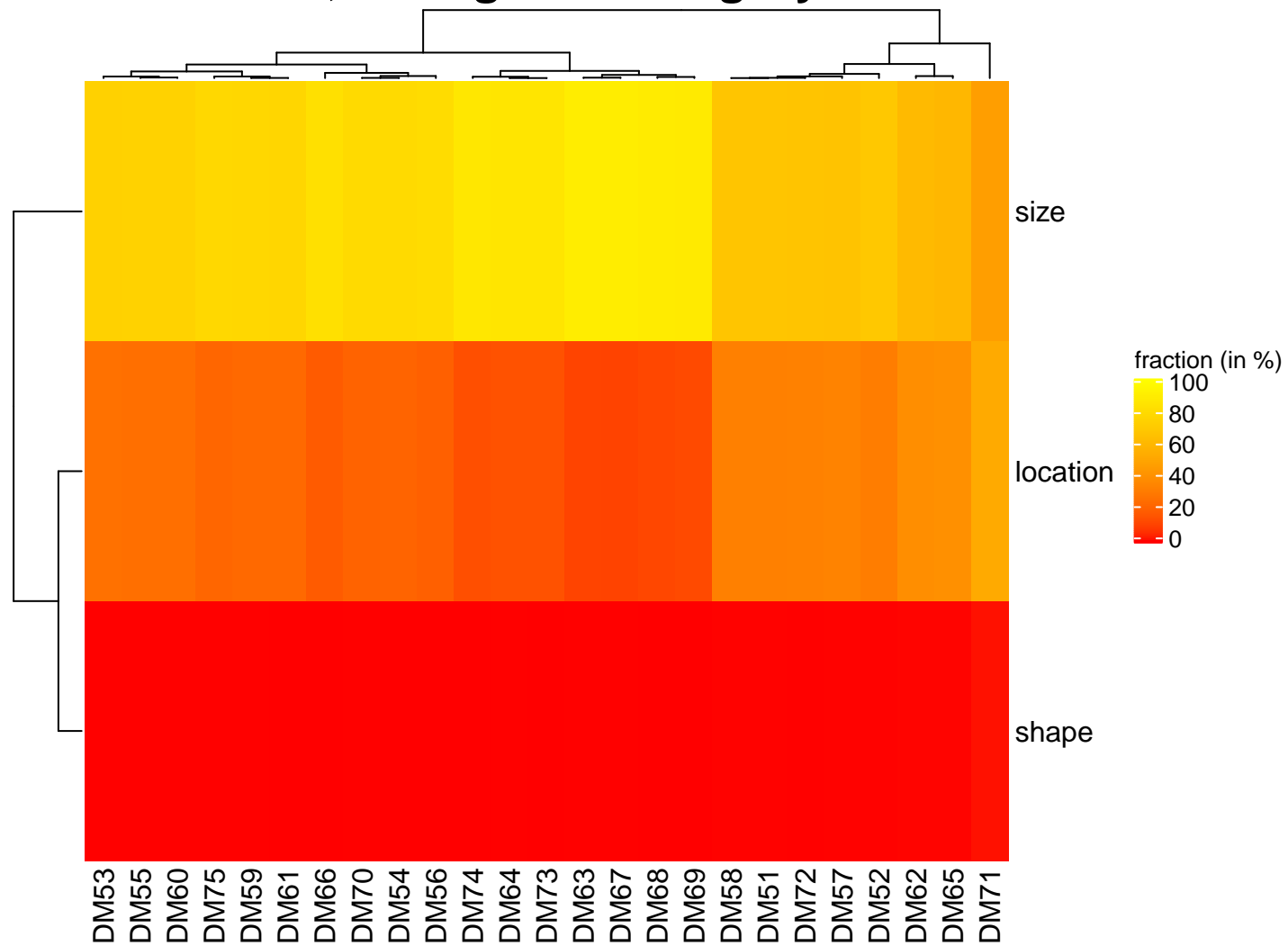

## C=500, strong DD: Category DB

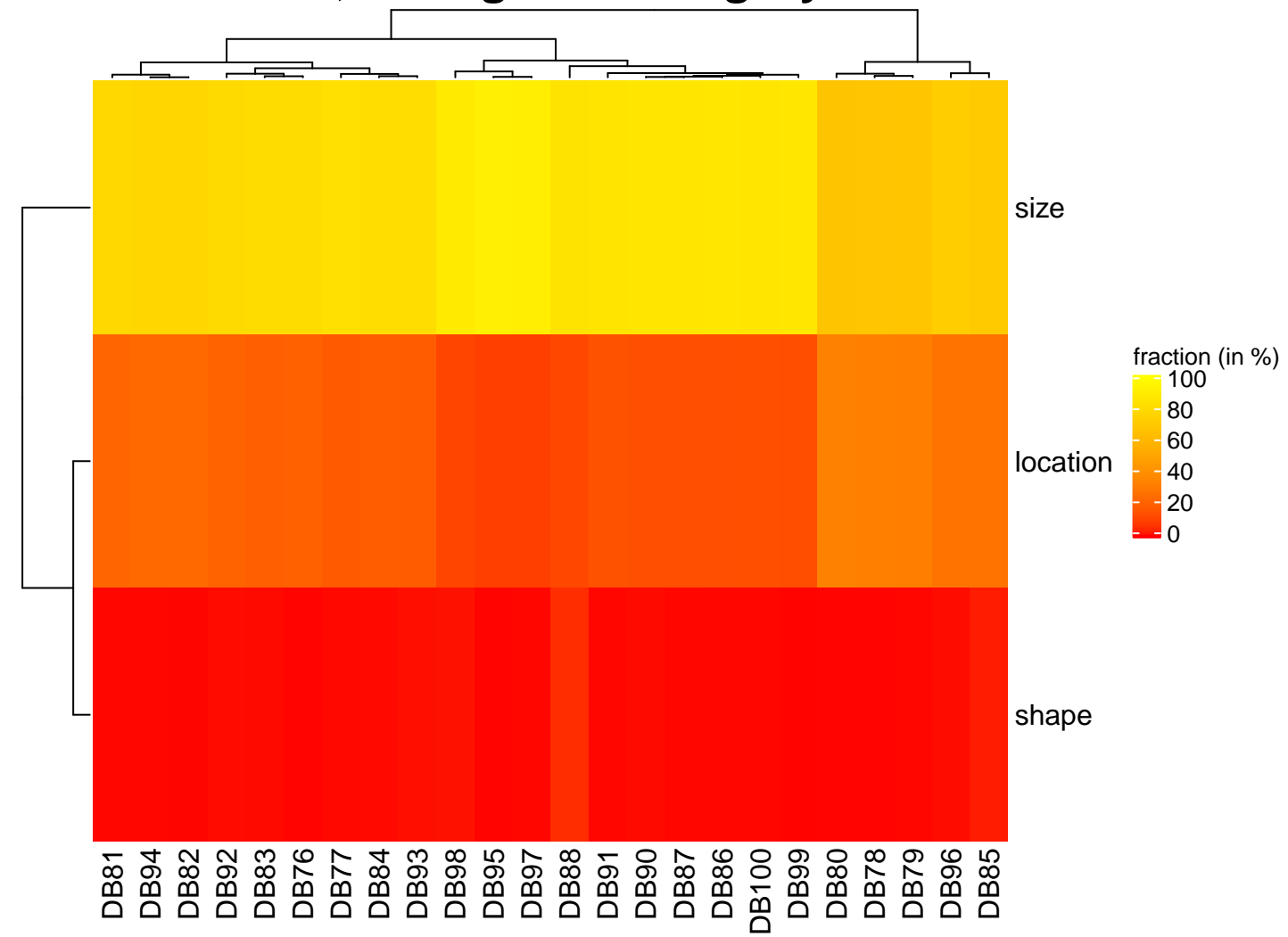

Supplement: btab226_Supplementary_Data [file btab226_supplementary_data.zip › Supplement_Revision2/Fig12.pdf]

C=500, strong DD

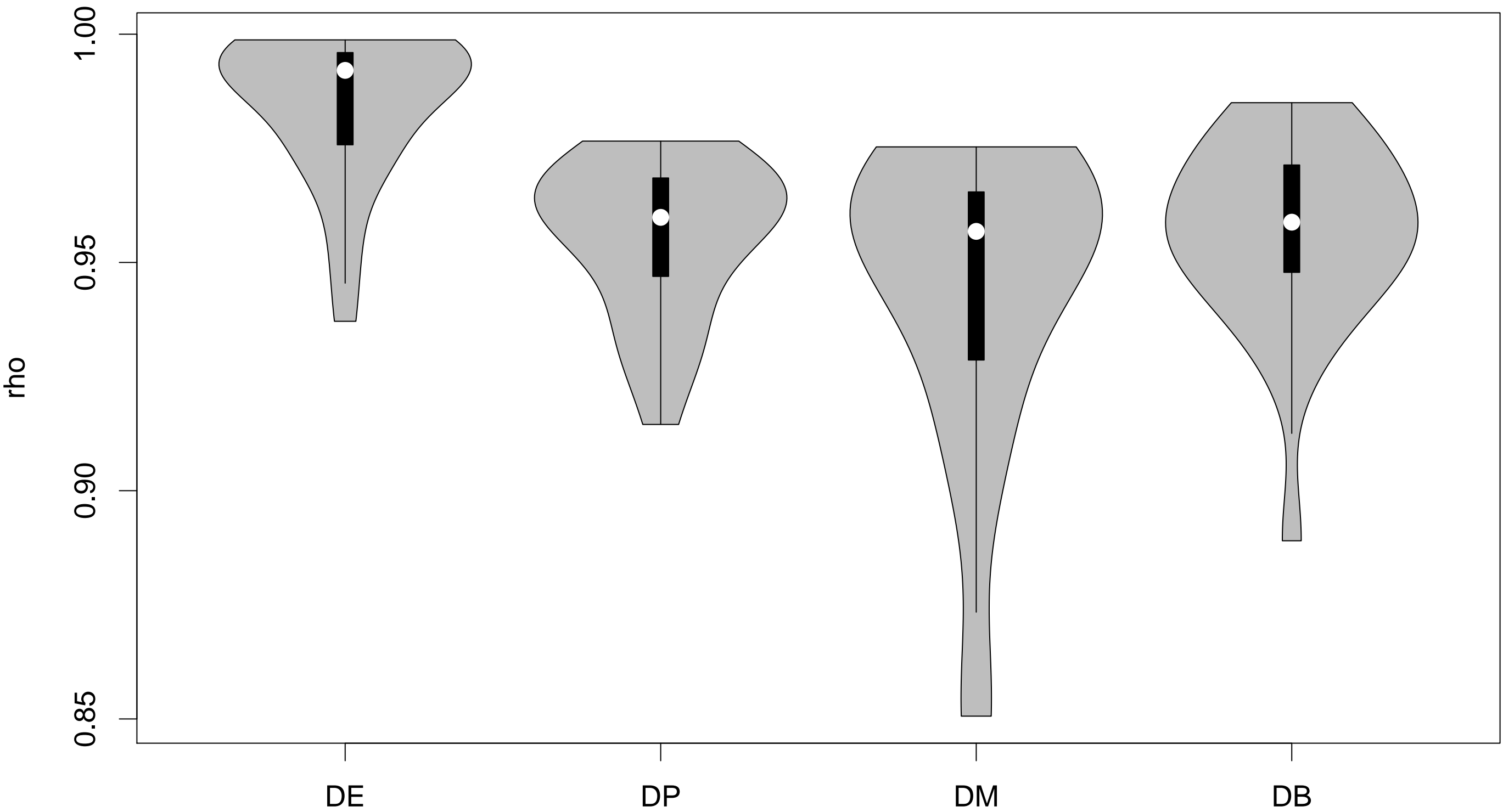

Supplement: btab226_Supplementary_Data [file btab226_supplementary_data.zip › Supplement_Revision2/Fig13.pdf]

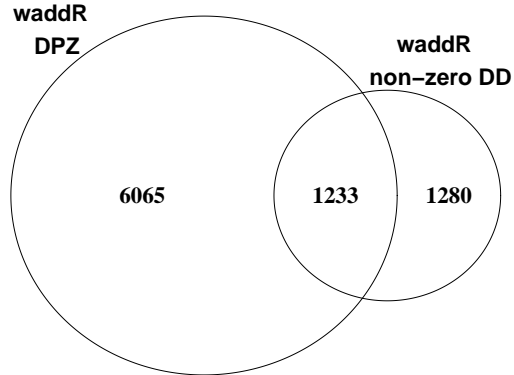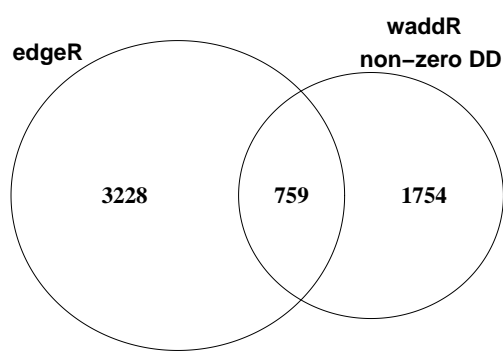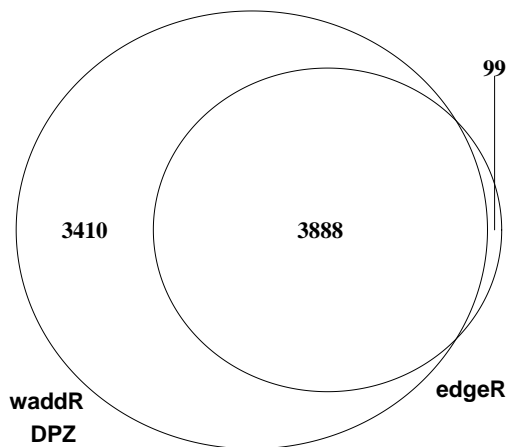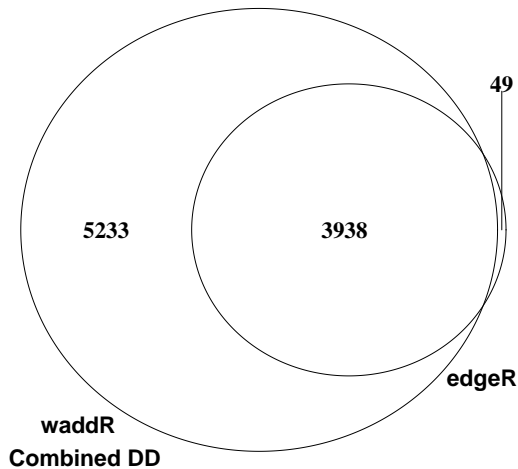

Supplement: btab226_Supplementary_Data [file btab226_supplementary_data.zip › Supplement_Revision2/Fig14.pdf]

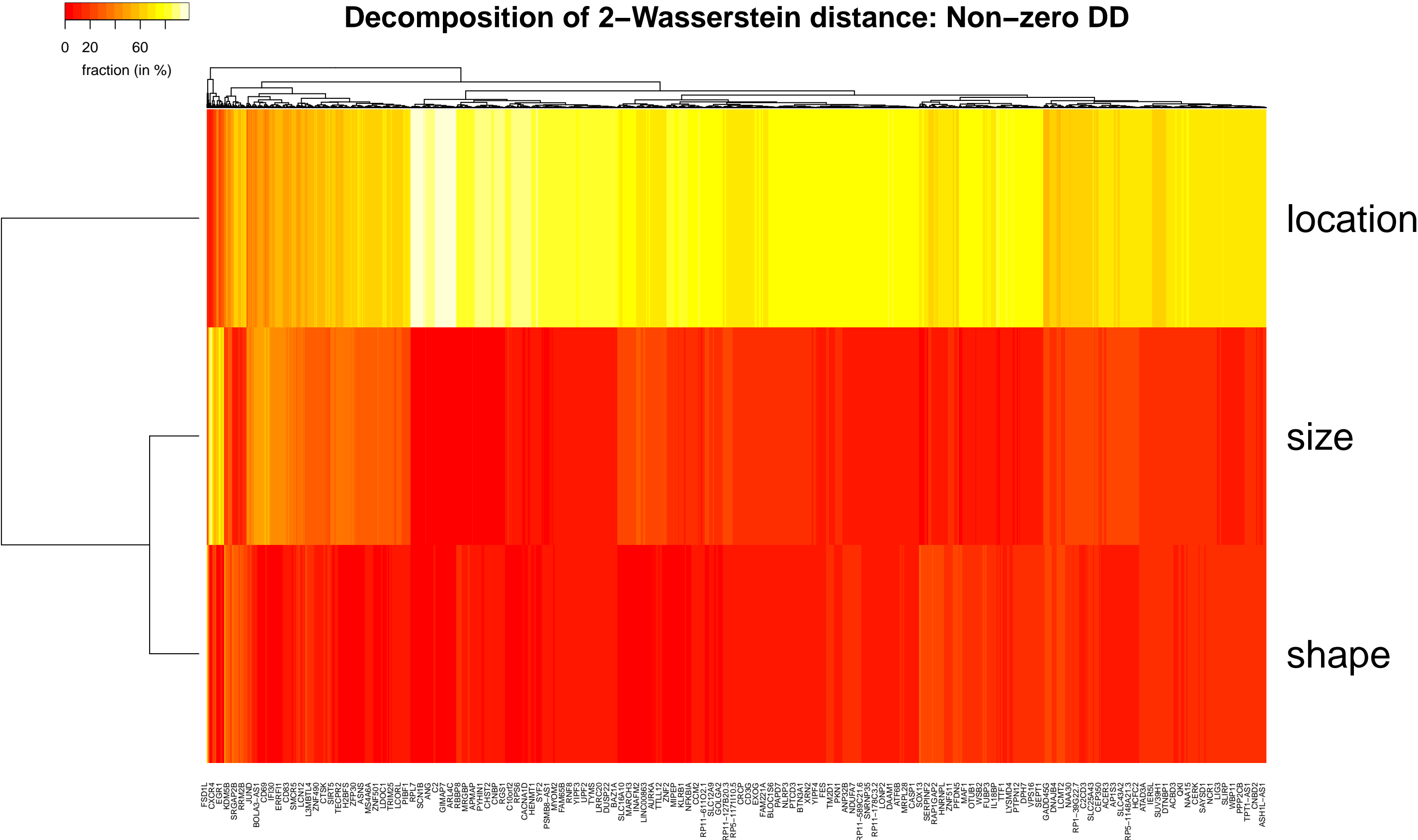

Supplement: btab226_Supplementary_Data [file btab226_supplementary_data.zip › Supplement_Revision2/Fig15.pdf]

The 30 Most Enriched GO Terms

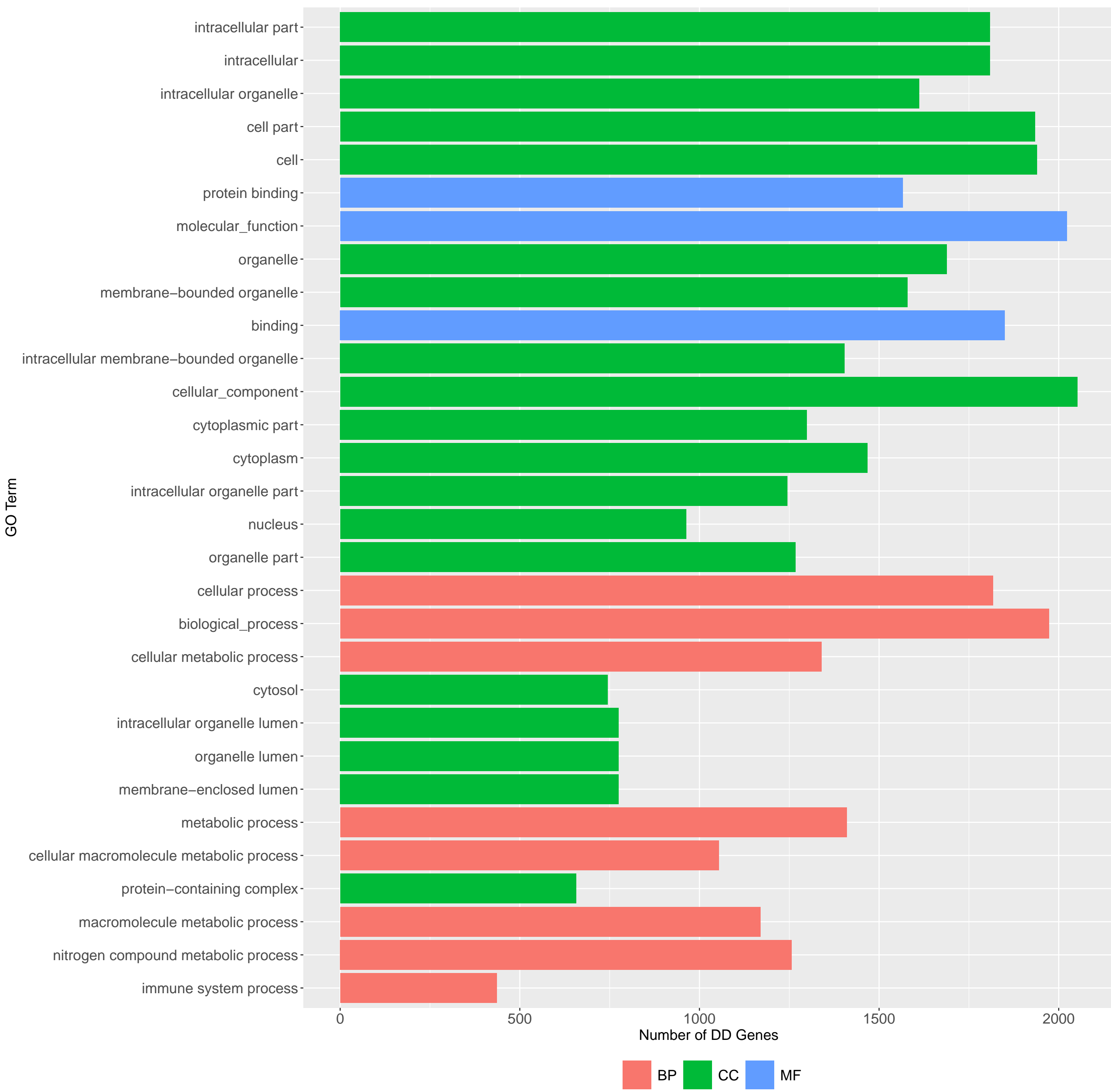

Supplement: btab226_Supplementary_Data [file btab226_supplementary_data.zip › Supplement_Revision2/Fig16.pdf]

Statistics of Pathway Enrichment

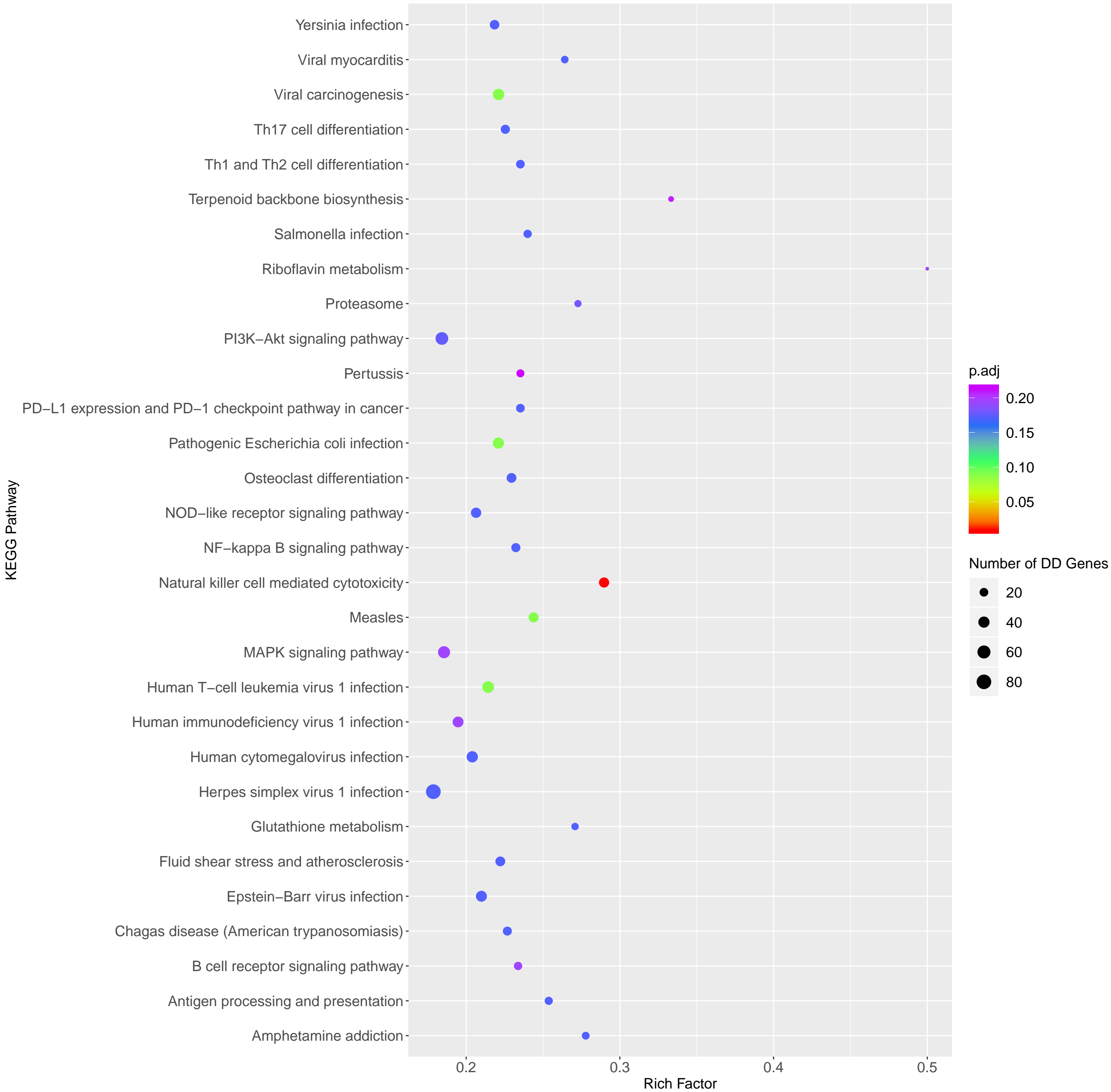

Supplement: btab226_Supplementary_Data [file btab226_supplementary_data.zip › Supplement_Revision2/Fig17.pdf]

The 30 Most Enriched GO Terms

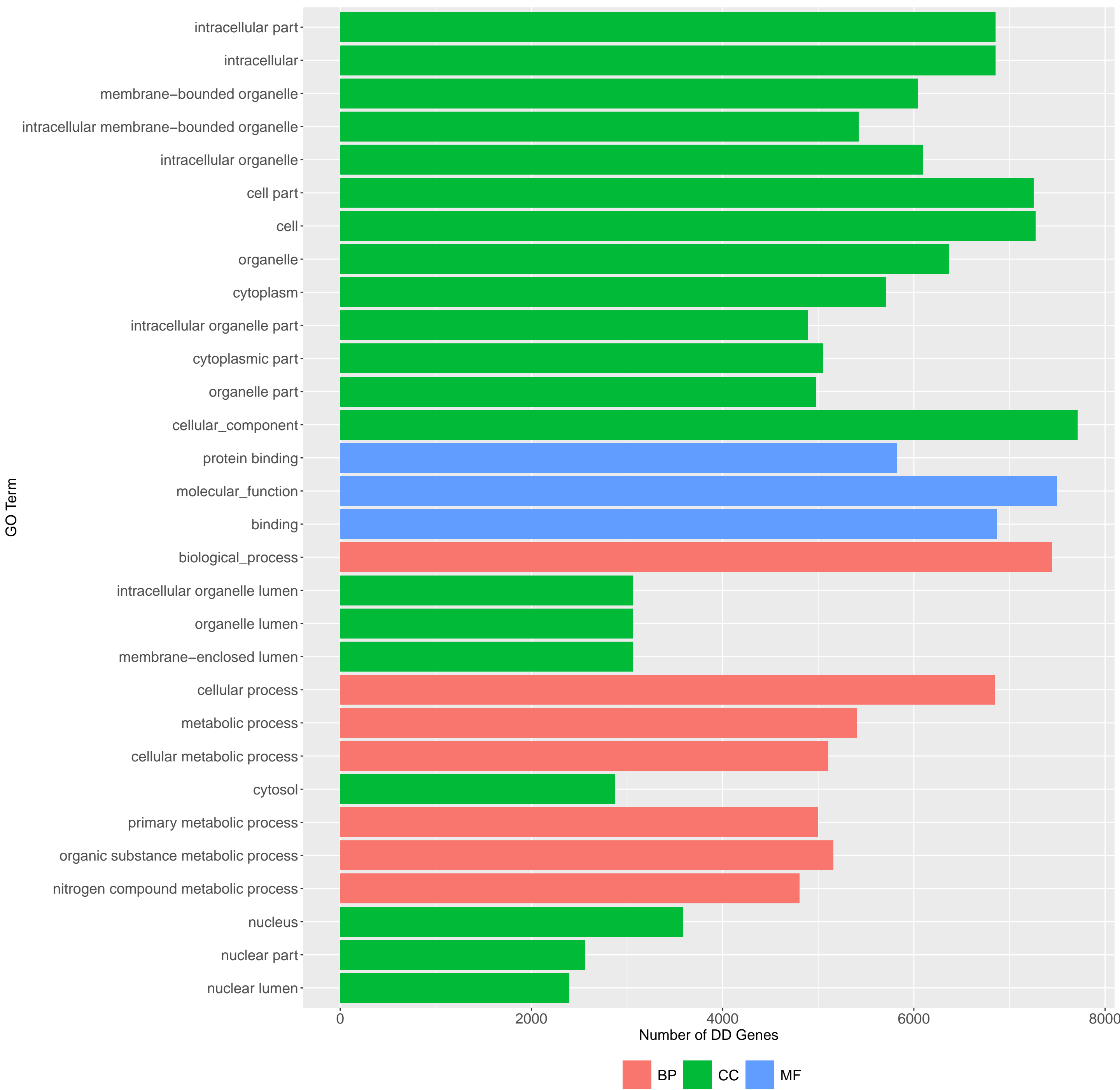

Supplement: btab226_Supplementary_Data [file btab226_supplementary_data.zip › Supplement_Revision2/Fig18.pdf]

Statistics of Pathway Enrichment

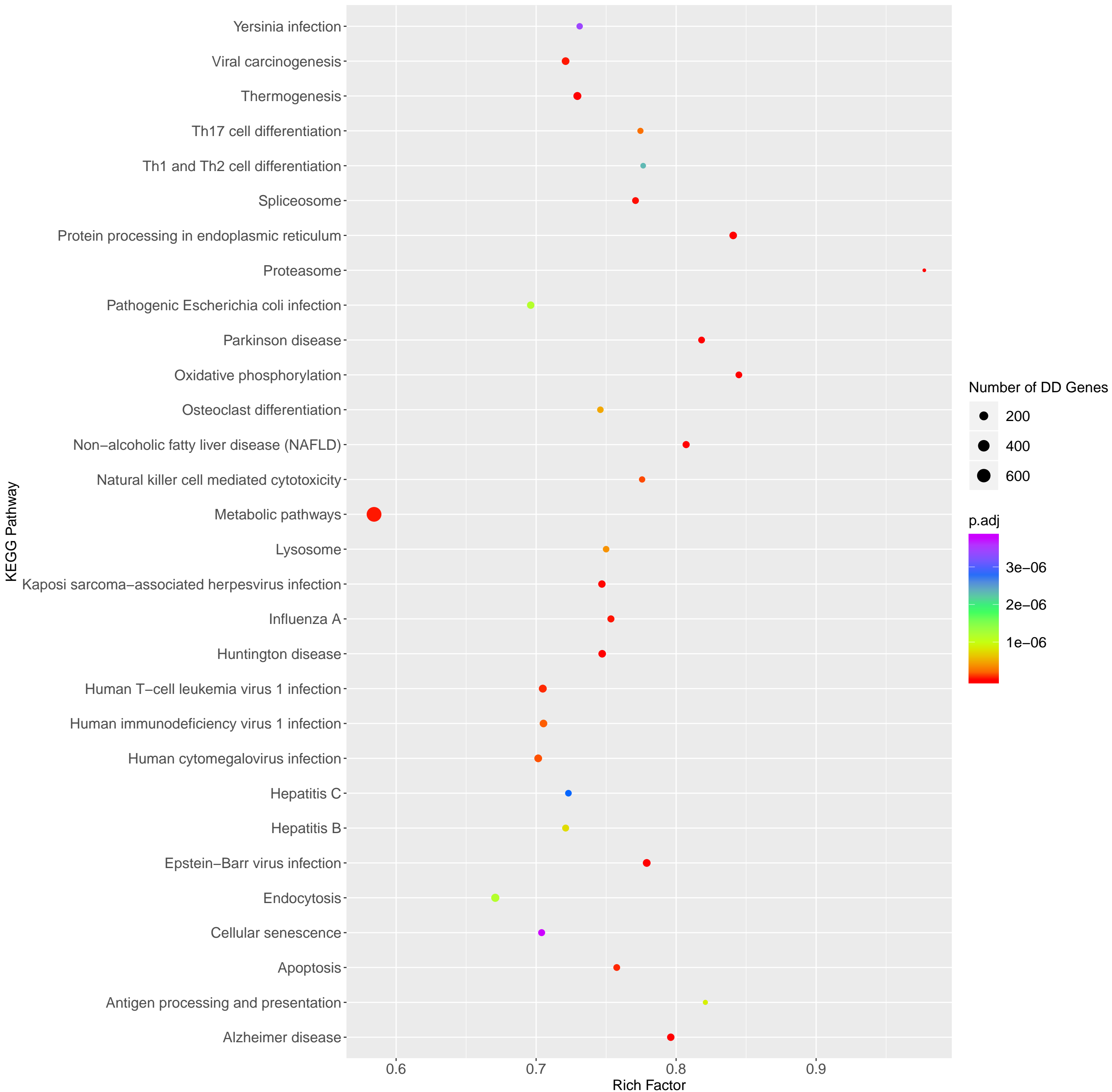

Supplement: btab226_Supplementary_Data [file btab226_supplementary_data.zip › Supplement_Revision2/Fig19.pdf]

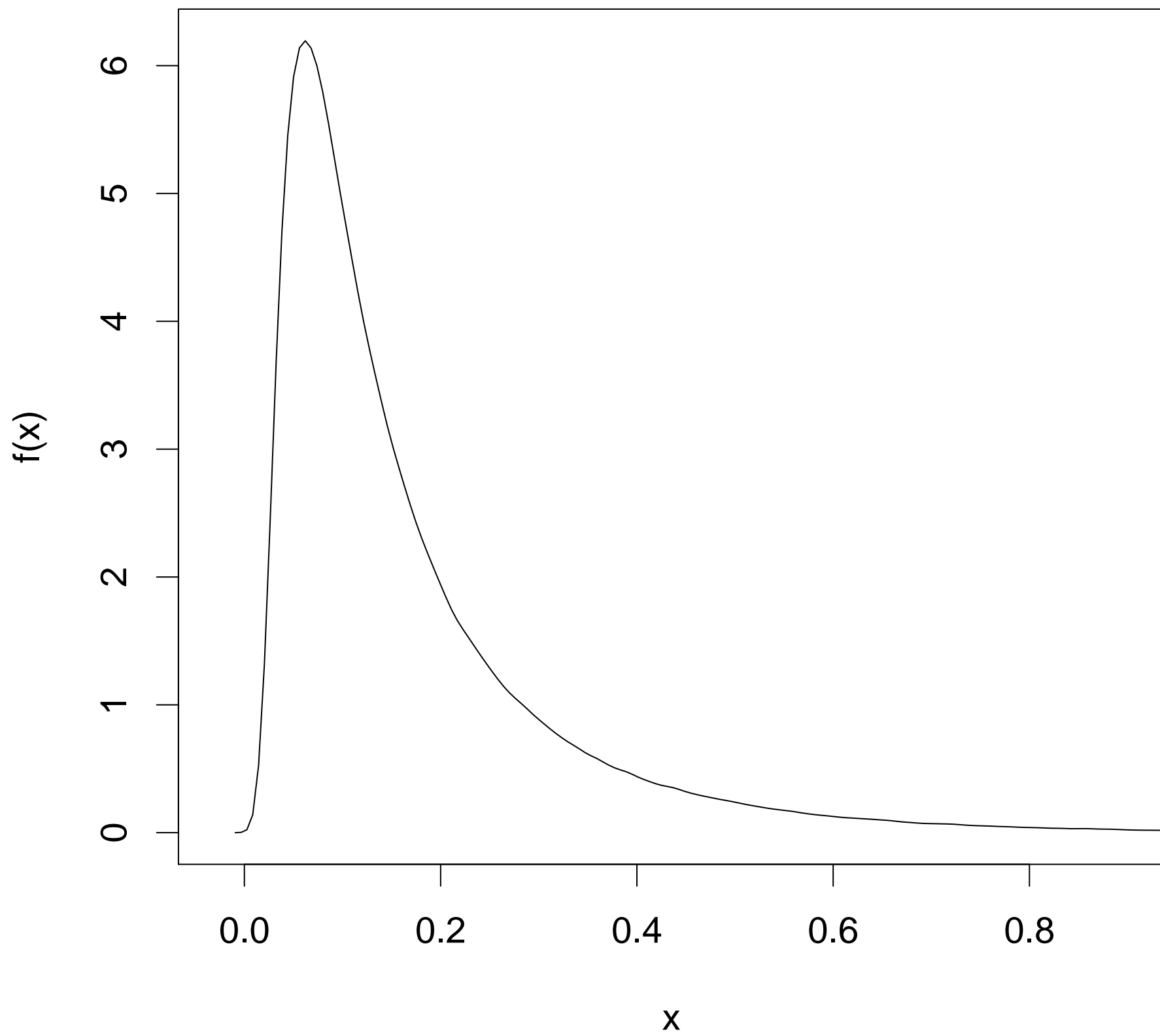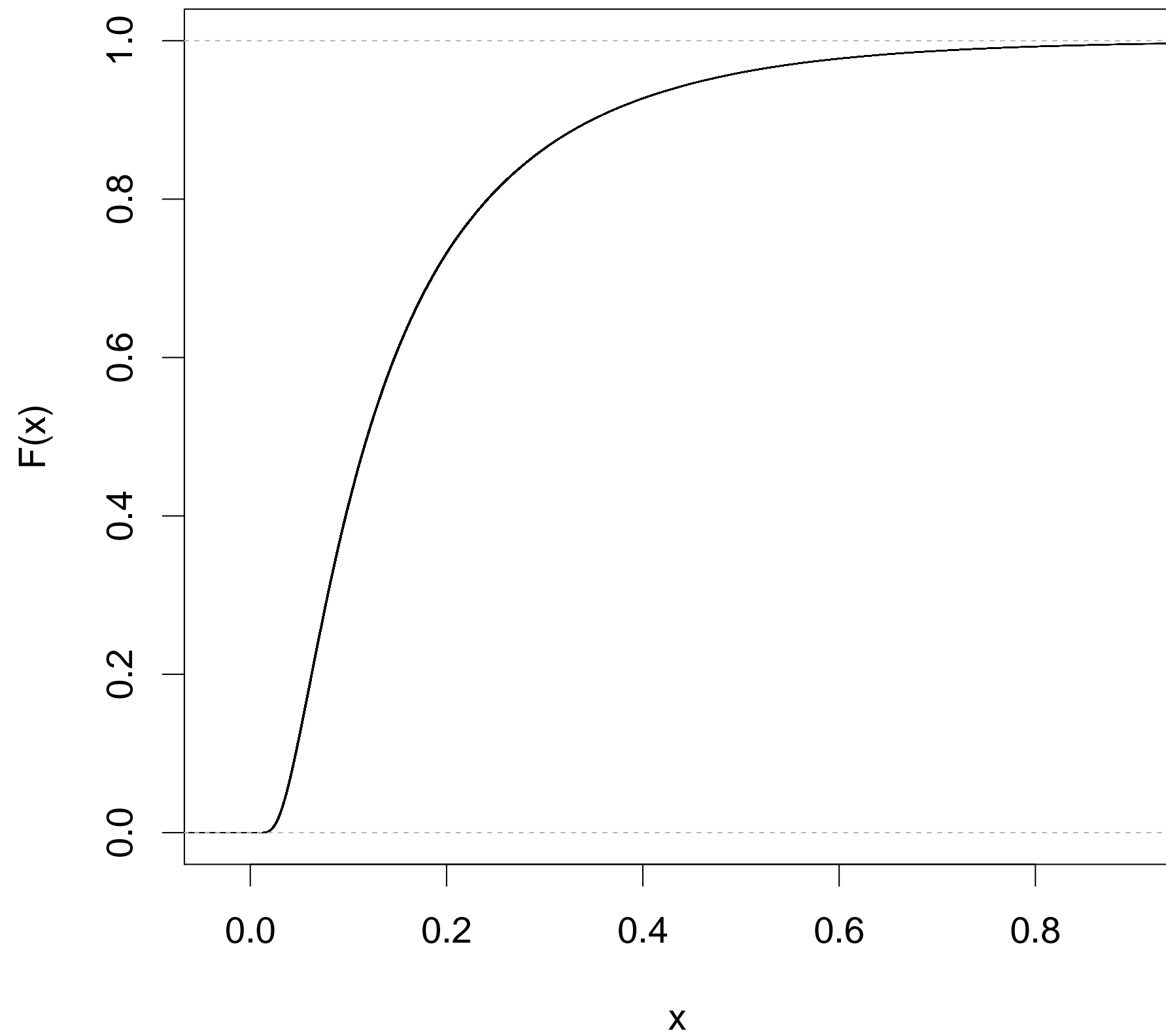

Supplement: btab226_Supplementary_Data [file btab226_supplementary_data.zip › Supplement_Revision2/Fig2.pdf]

No DD

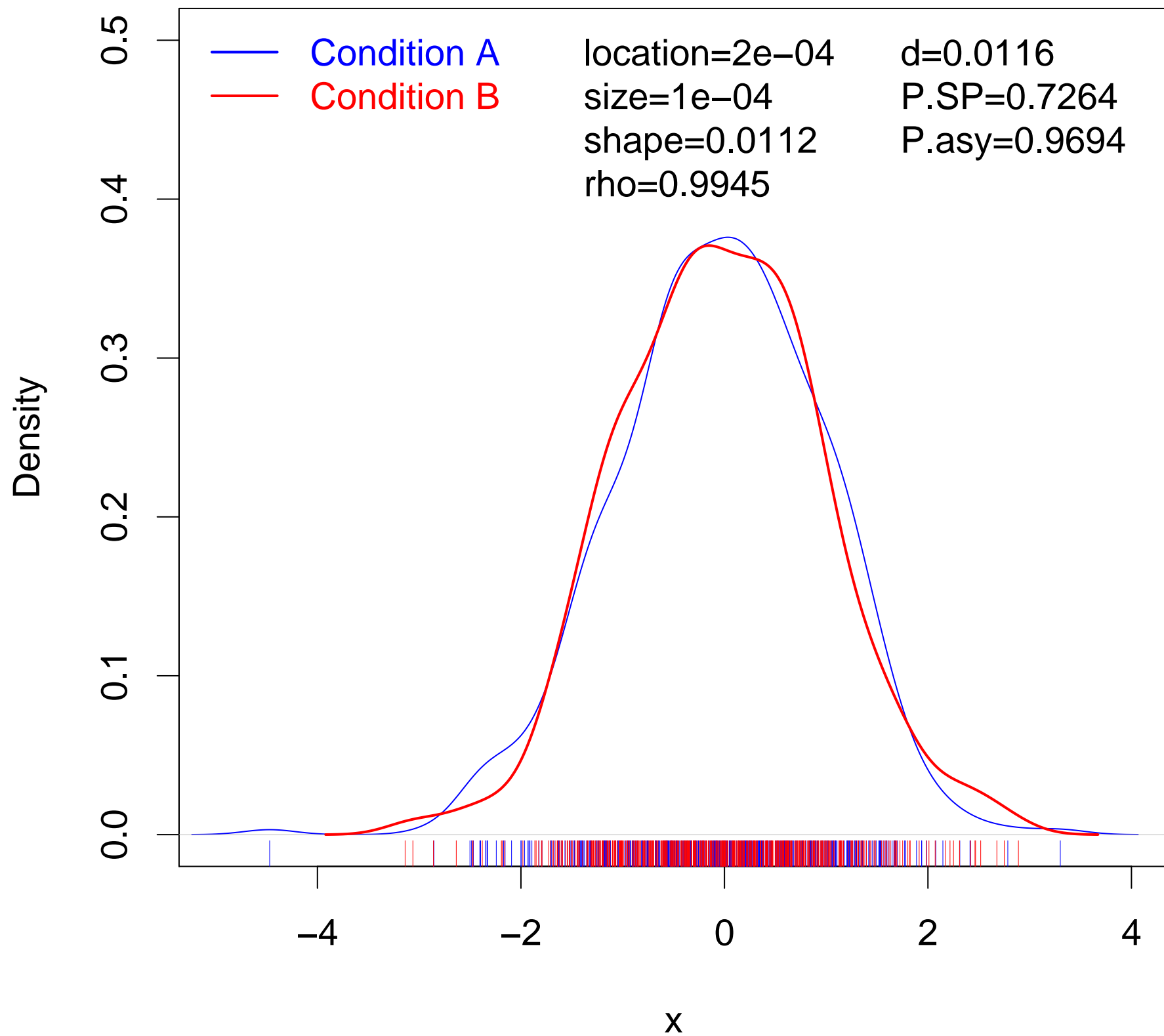

DD

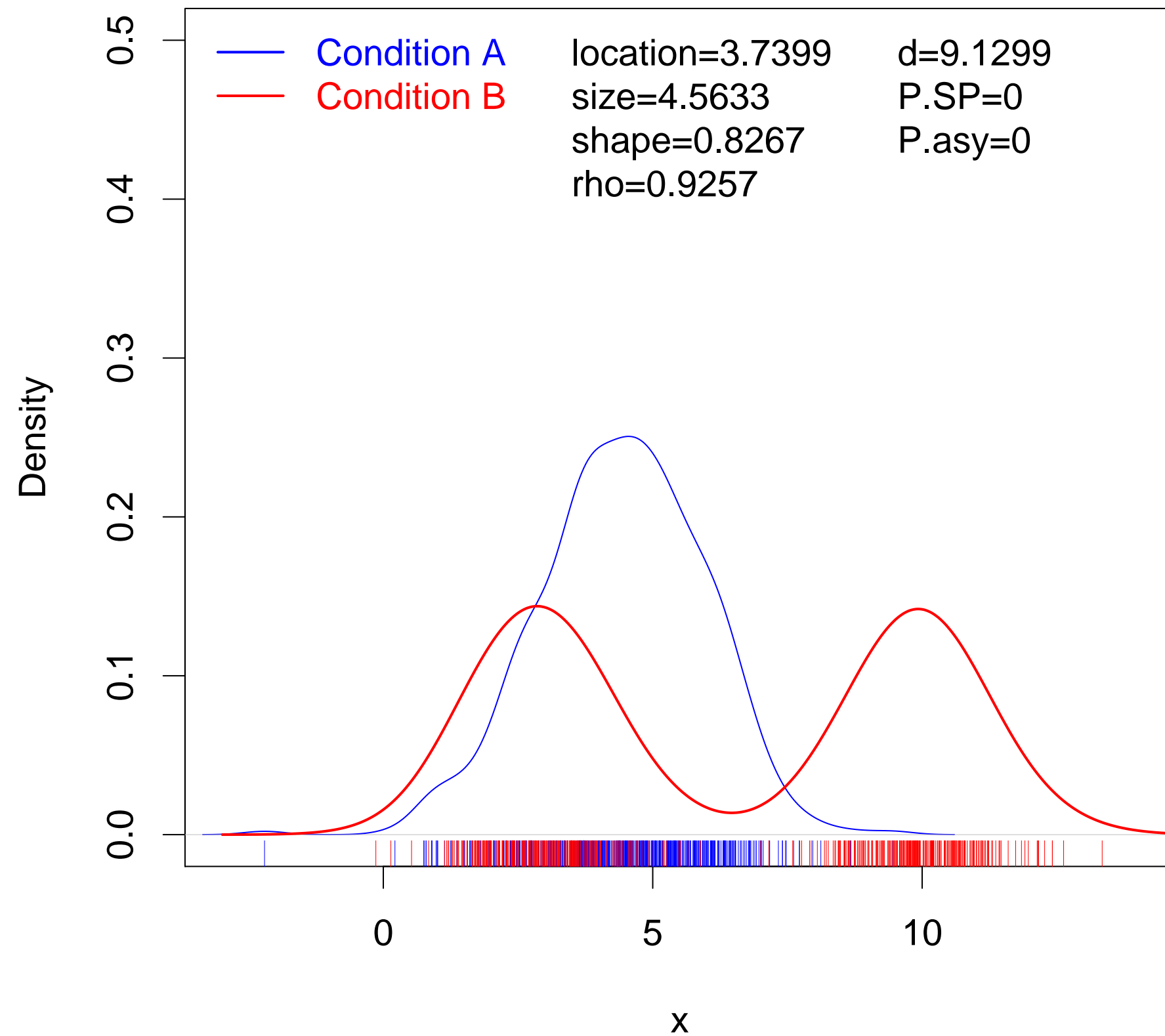

Supplement: btab226_Supplementary_Data [file btab226_supplementary_data.zip › Supplement_Revision2/Fig3.pdf]

M=50

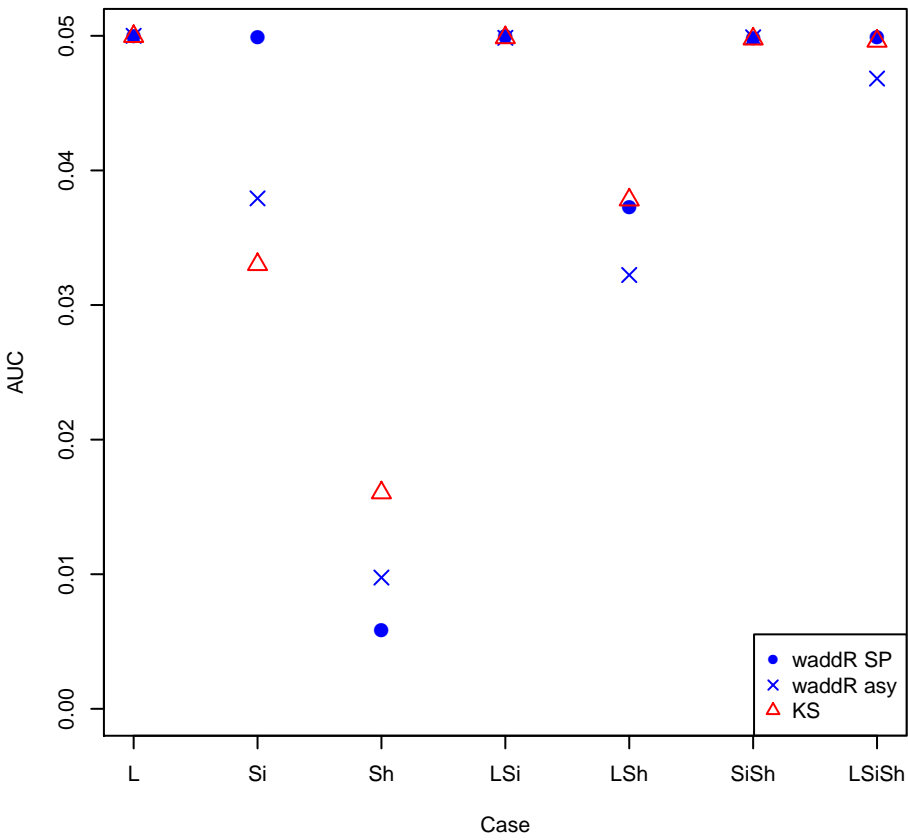

M=100

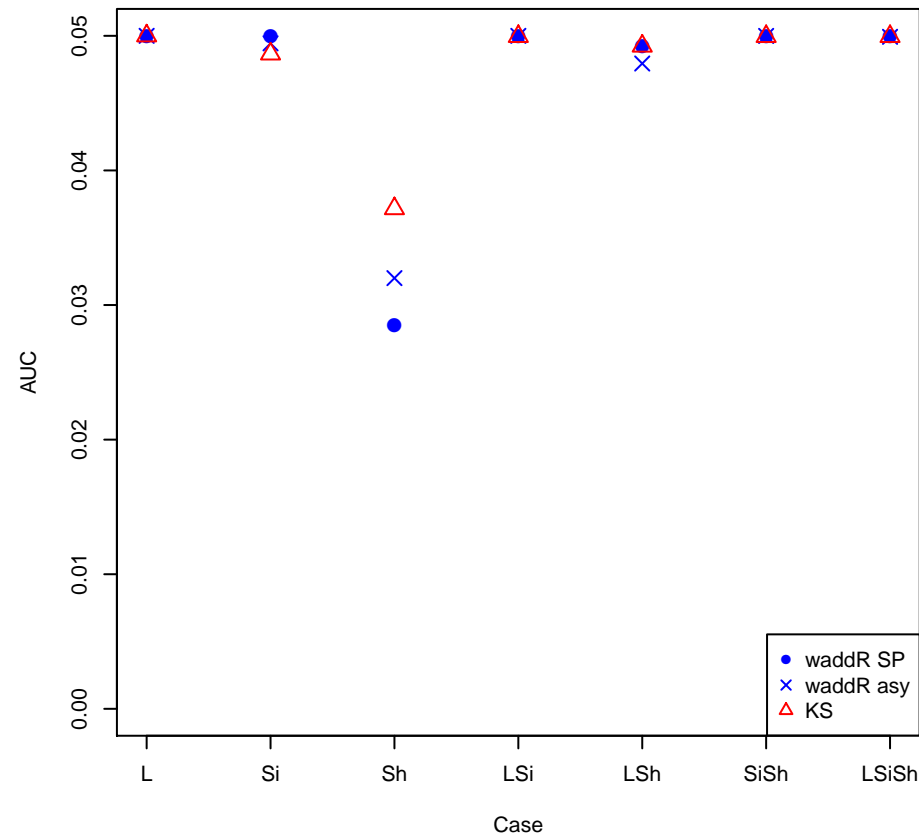

M=500

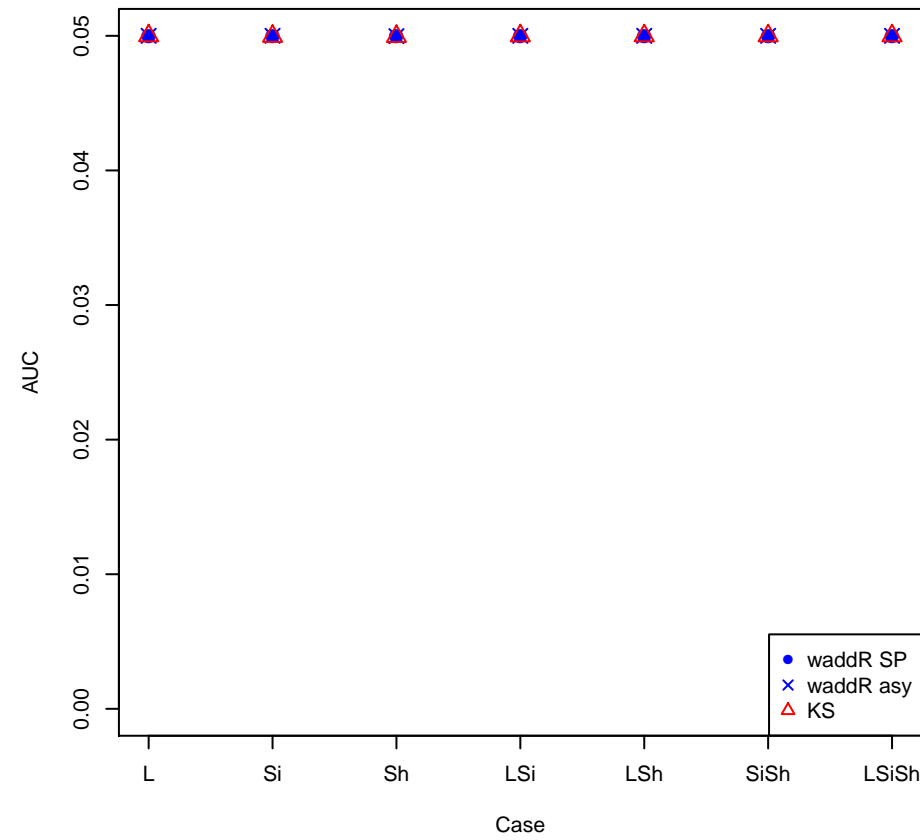

Supplement: btab226_Supplementary_Data [file btab226_supplementary_data.zip › Supplement_Revision2/Fig4.pdf]

**M=25**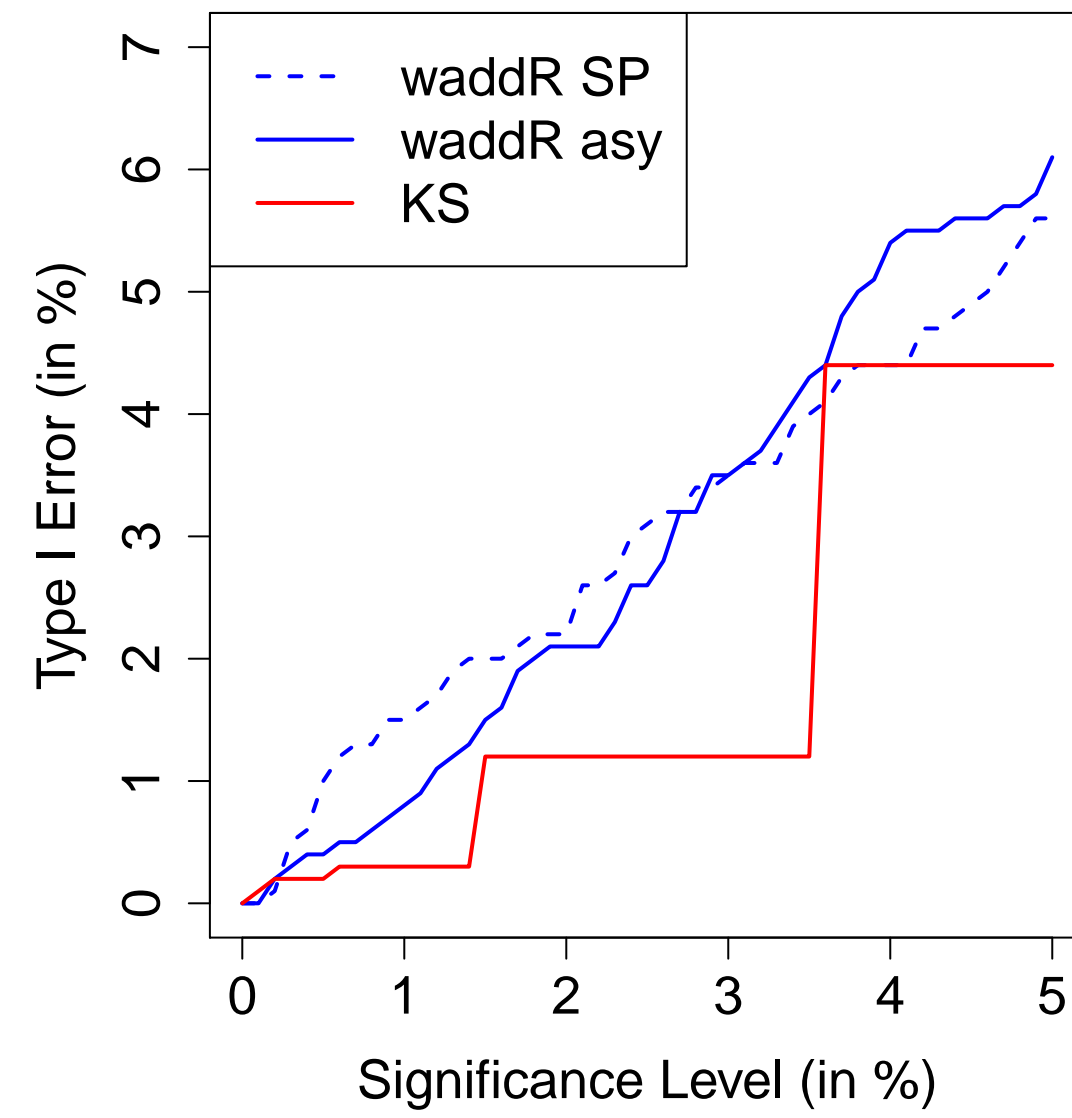**M=50**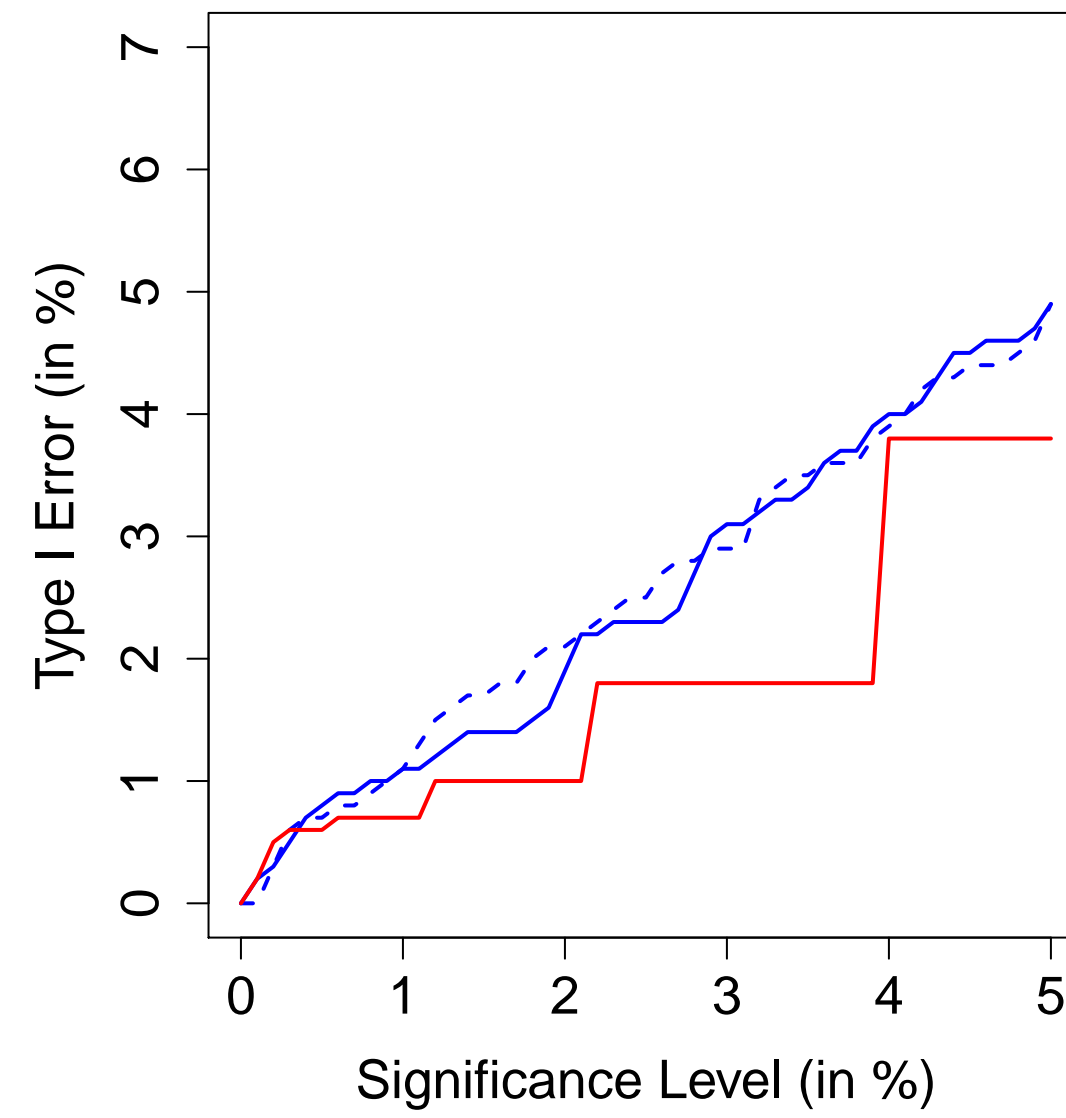**M=75**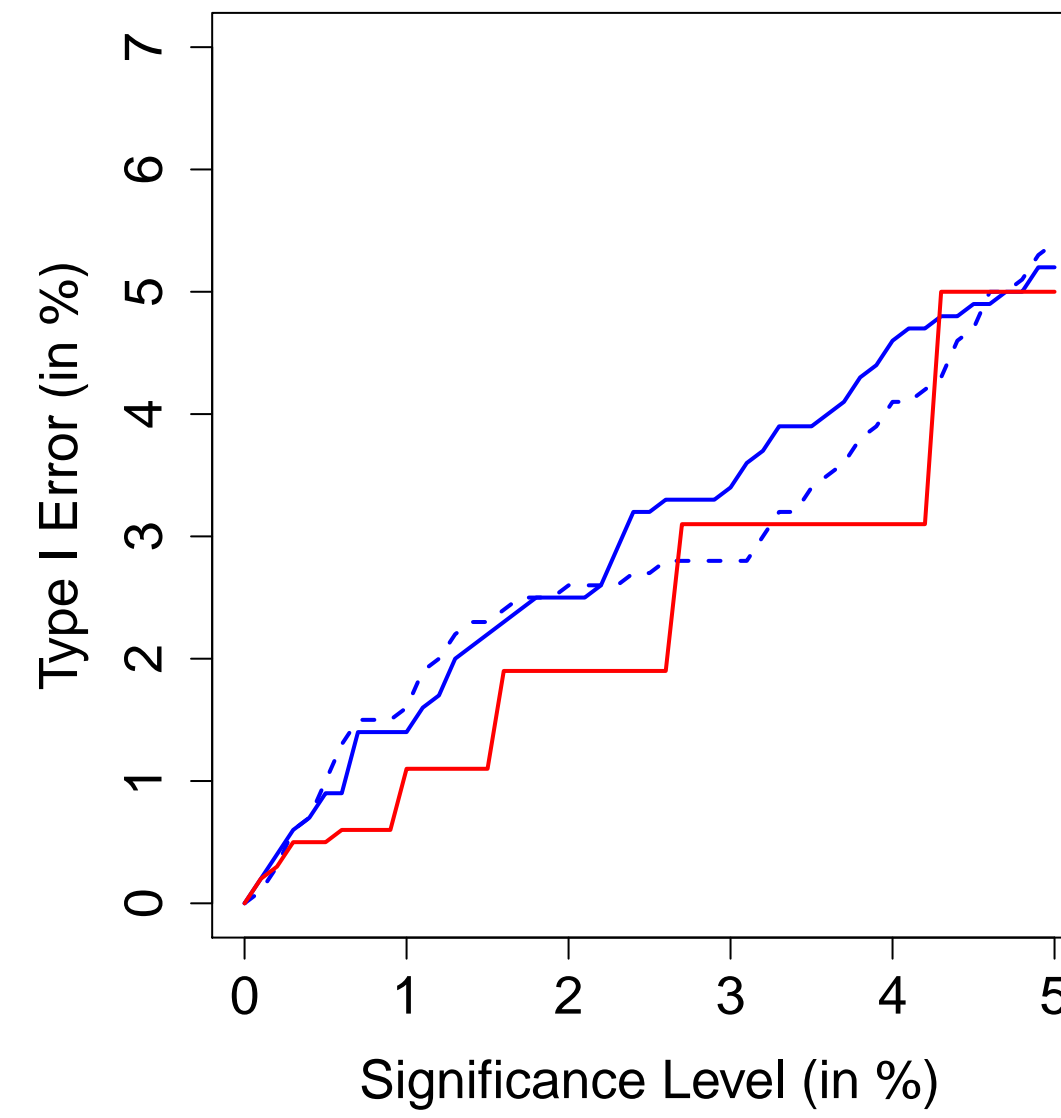**M=100**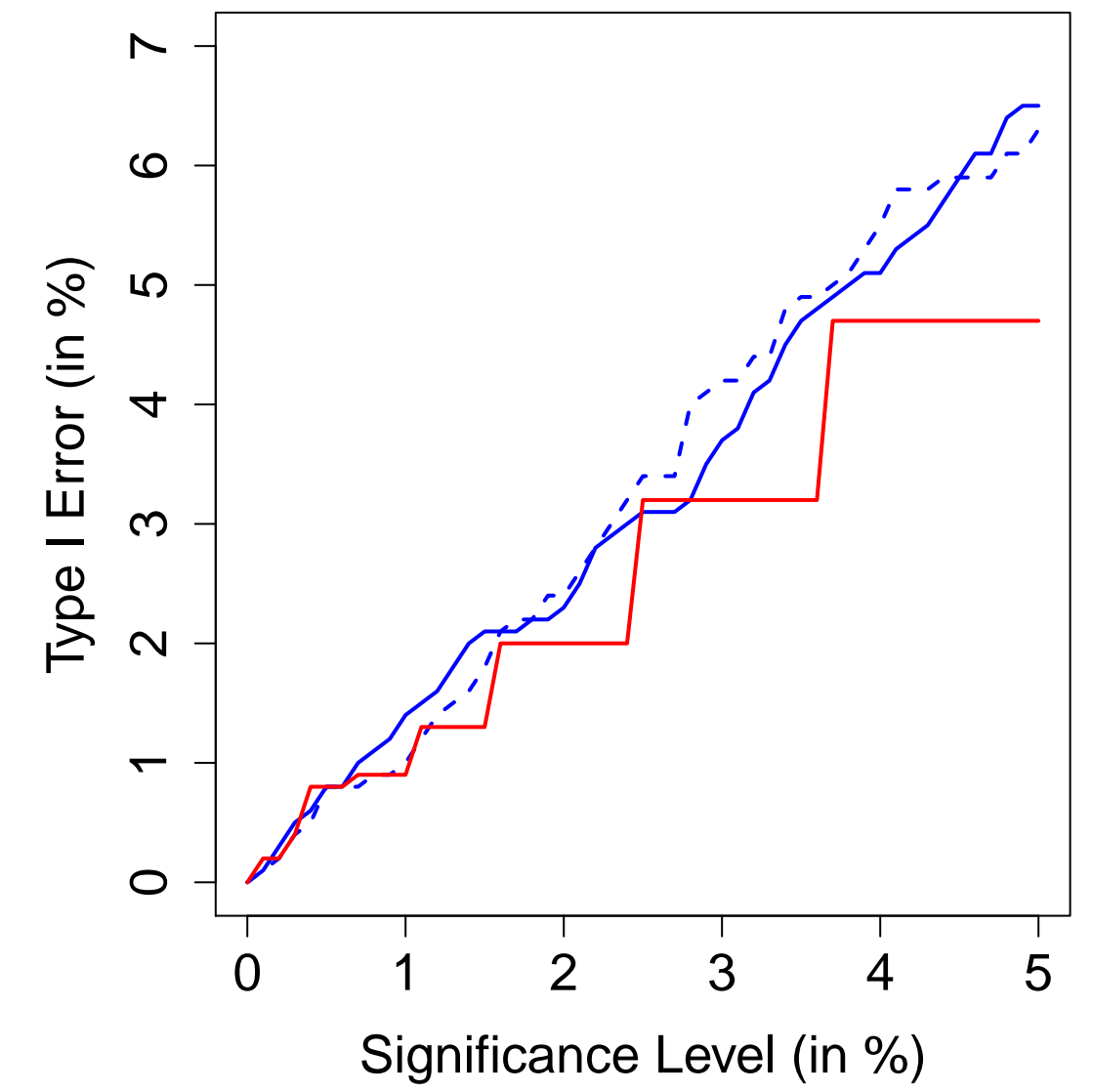**M=500**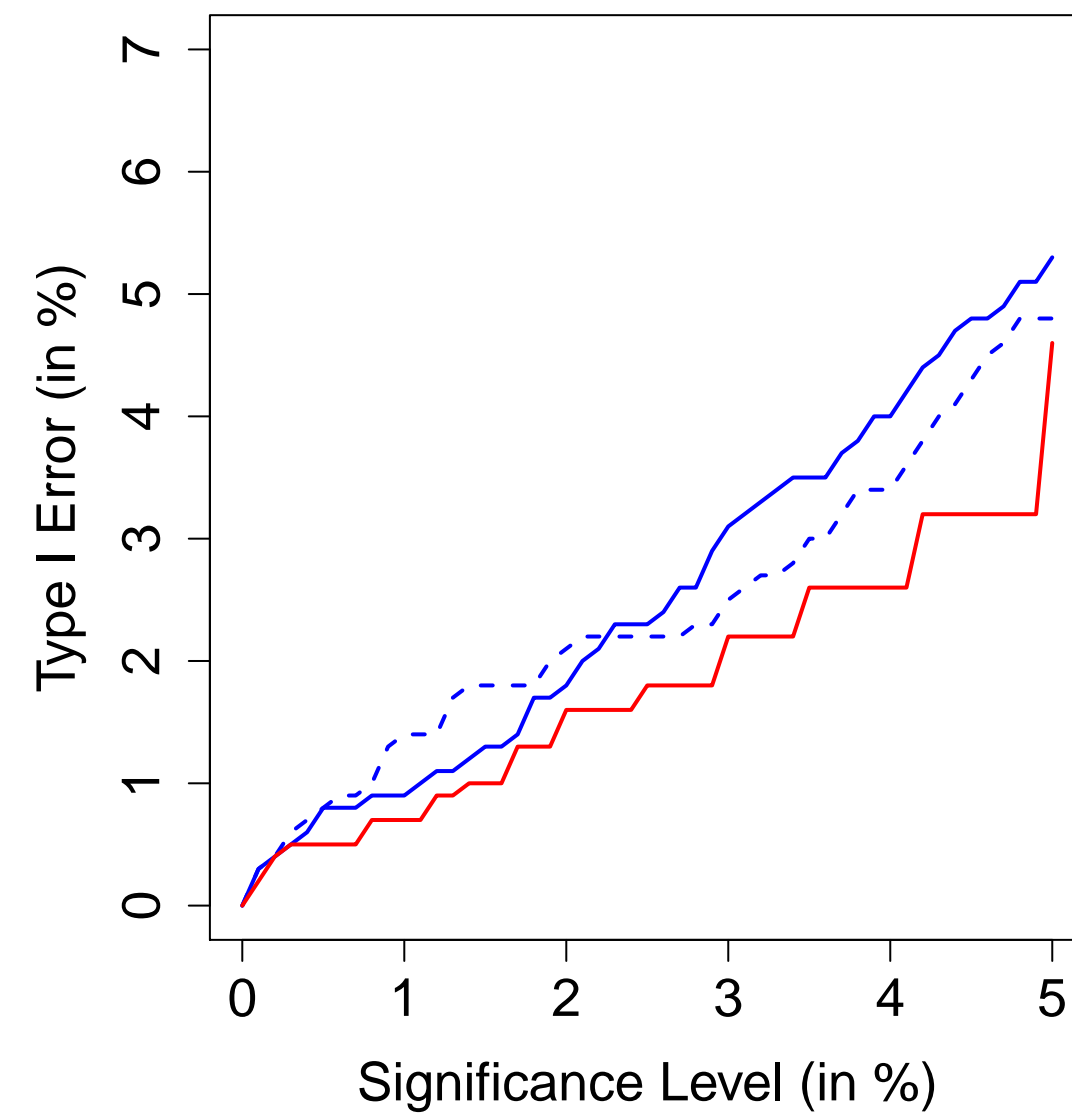**M=1000**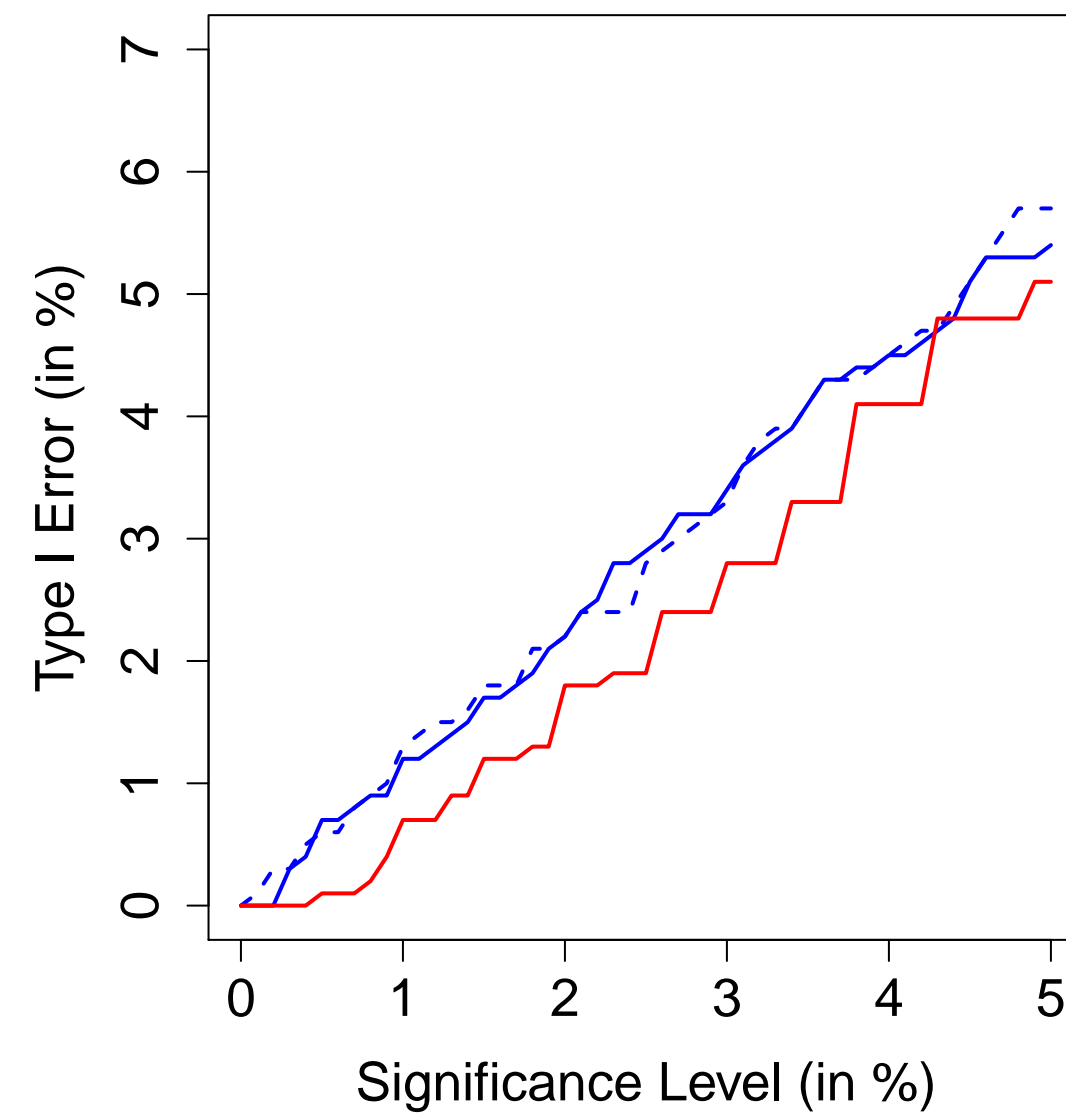**M=5000**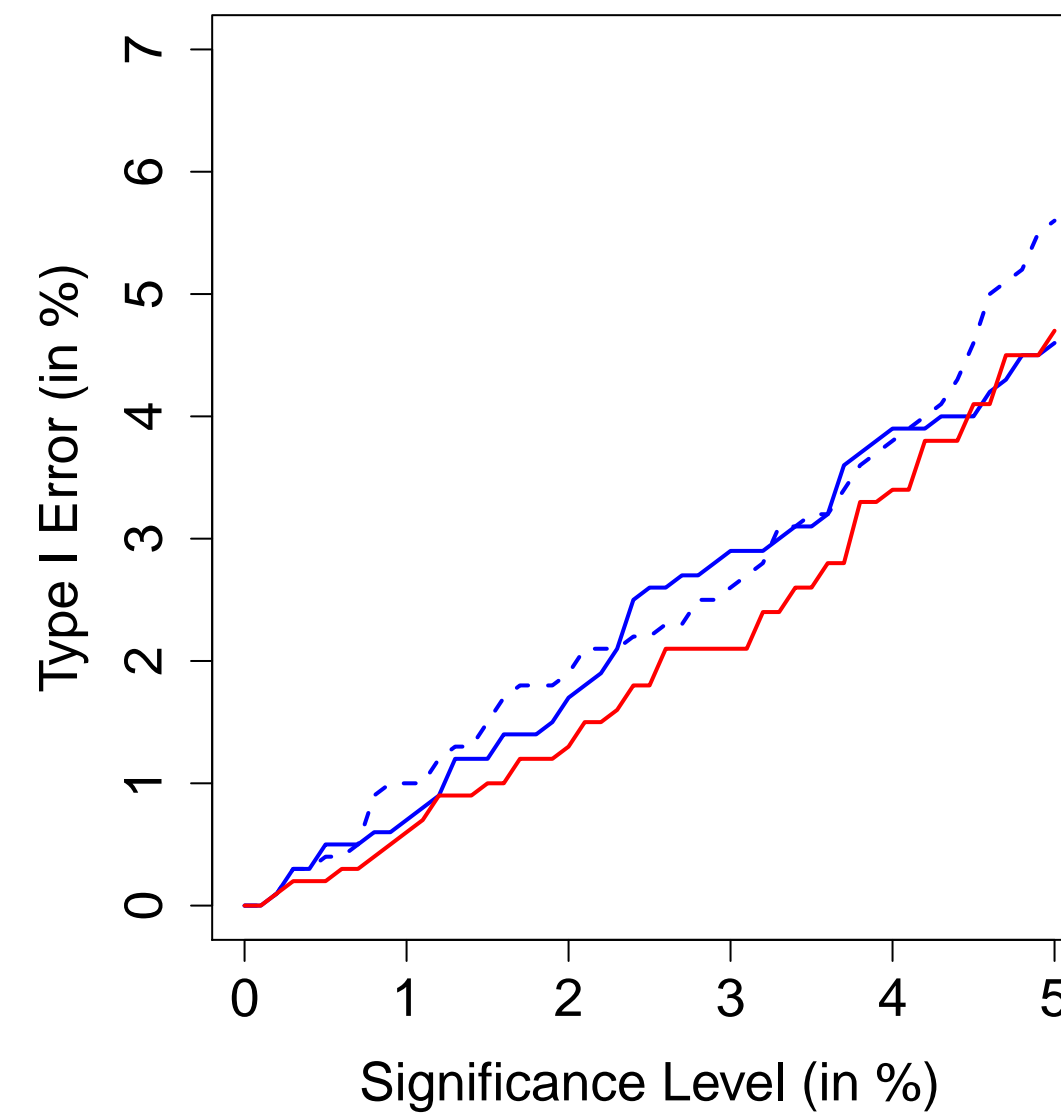

Supplement: btab226_Supplementary_Data [file btab226_supplementary_data.zip › Supplement_Revision2/Fig5.pdf]

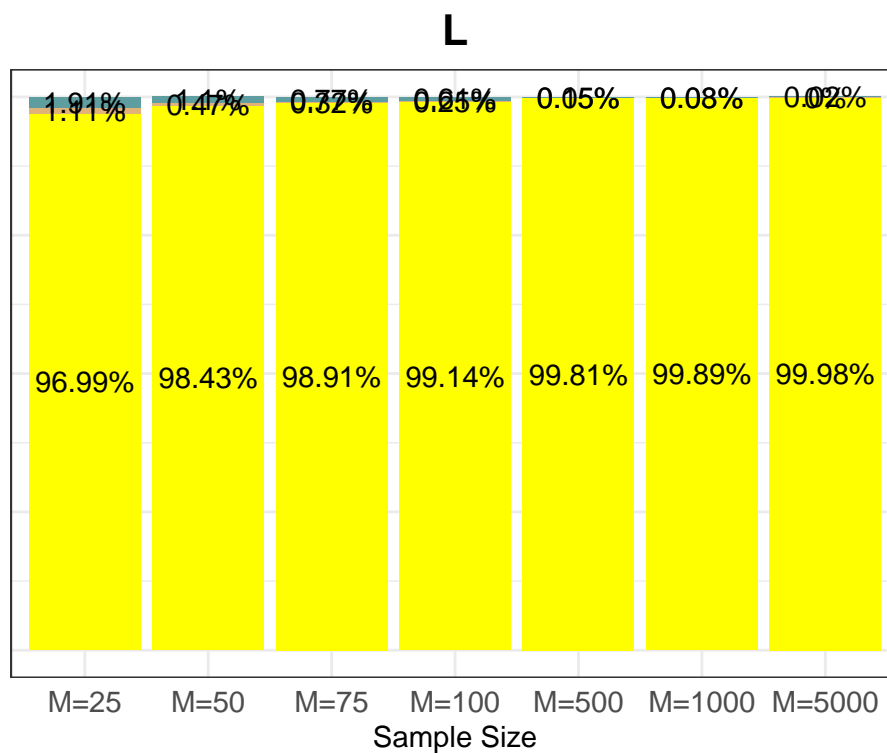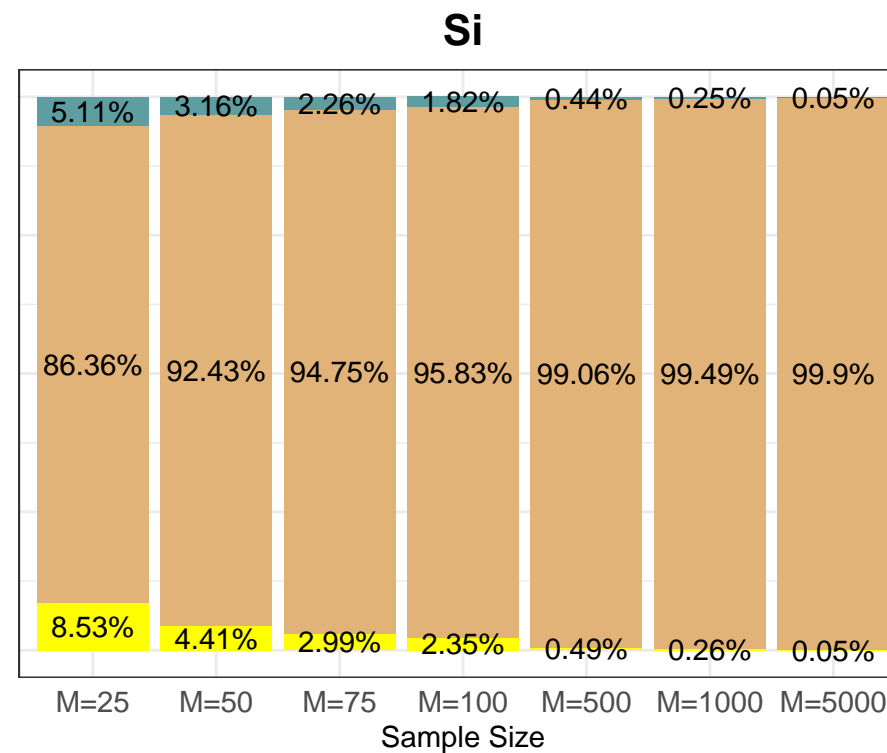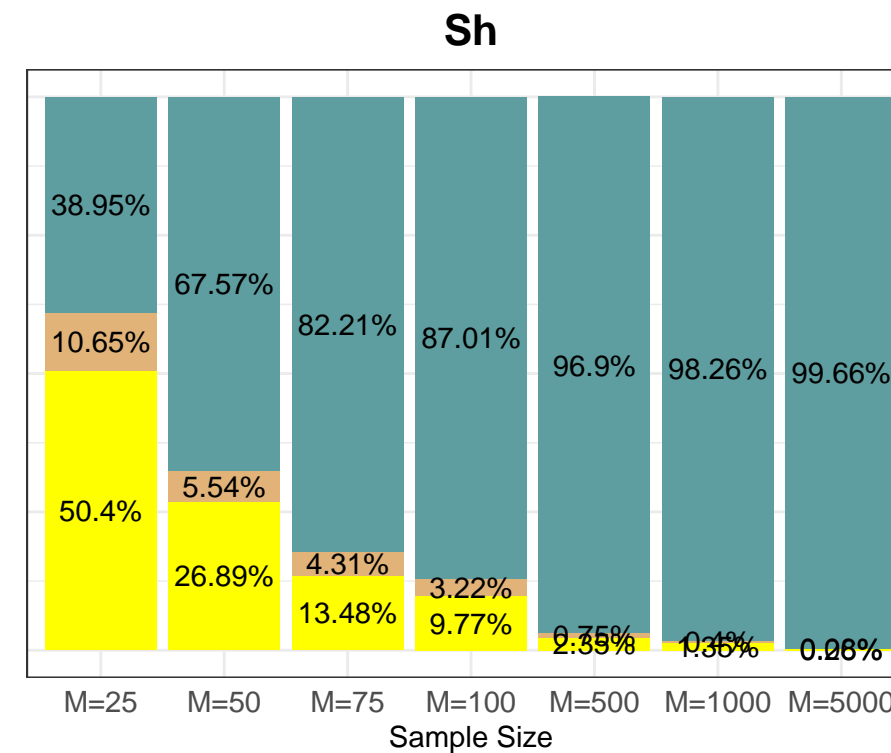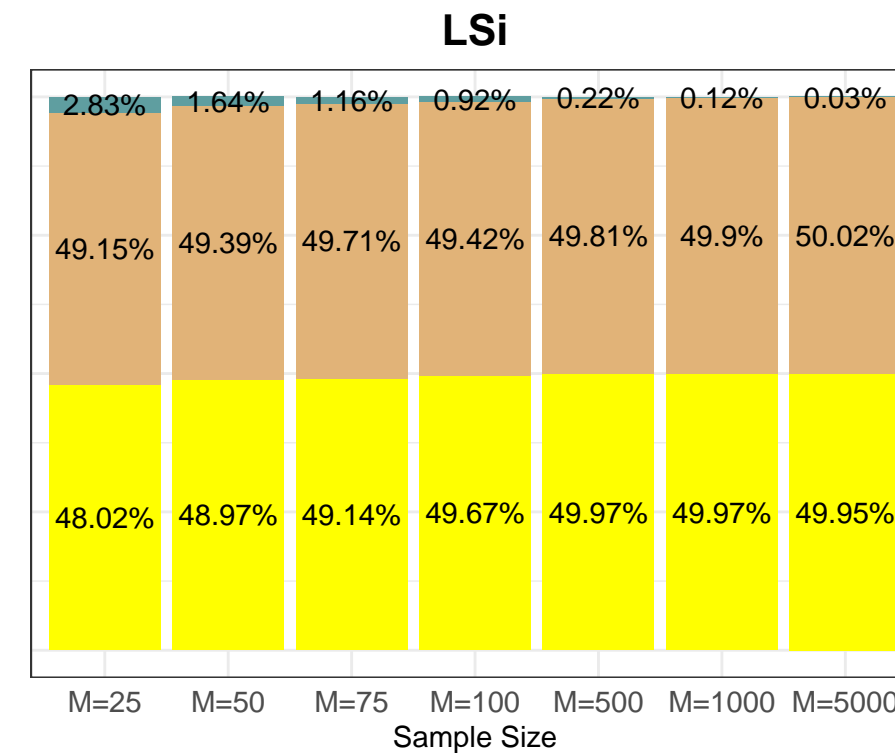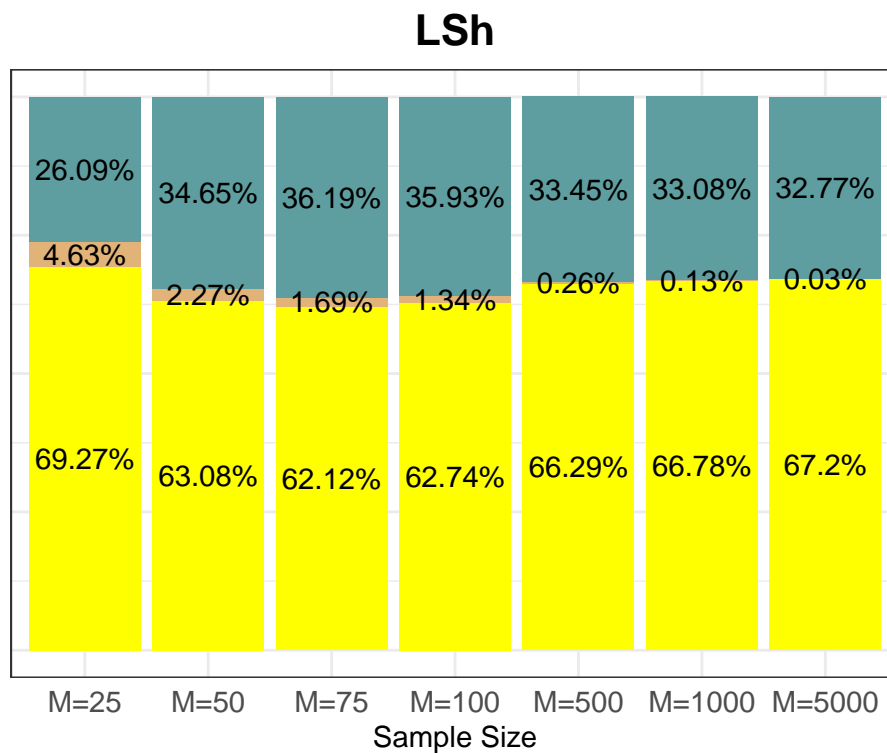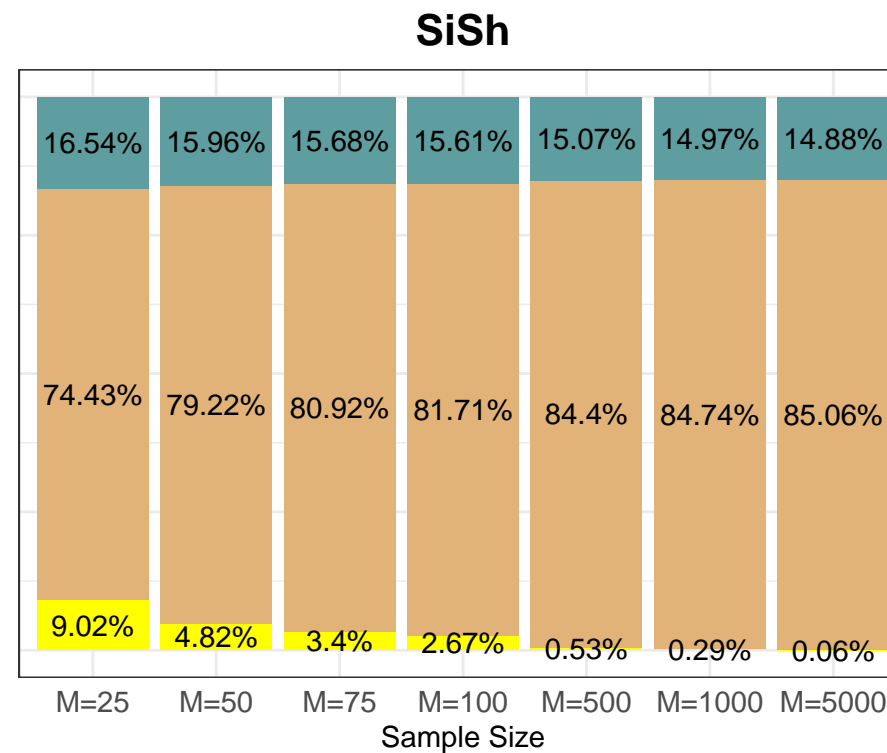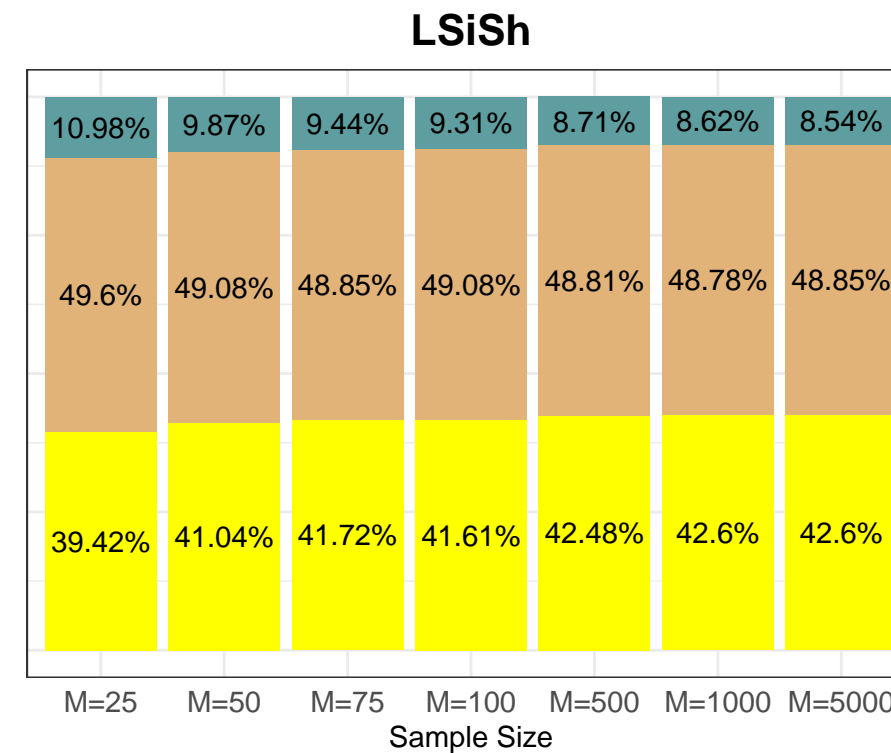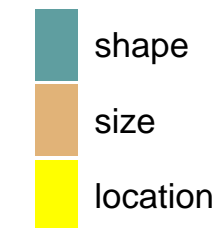

Supplement: btab226_Supplementary_Data [file btab226_supplementary_data.zip › Supplement_Revision2/Fig6.pdf]

**NDUFAB1 (non-DD)**

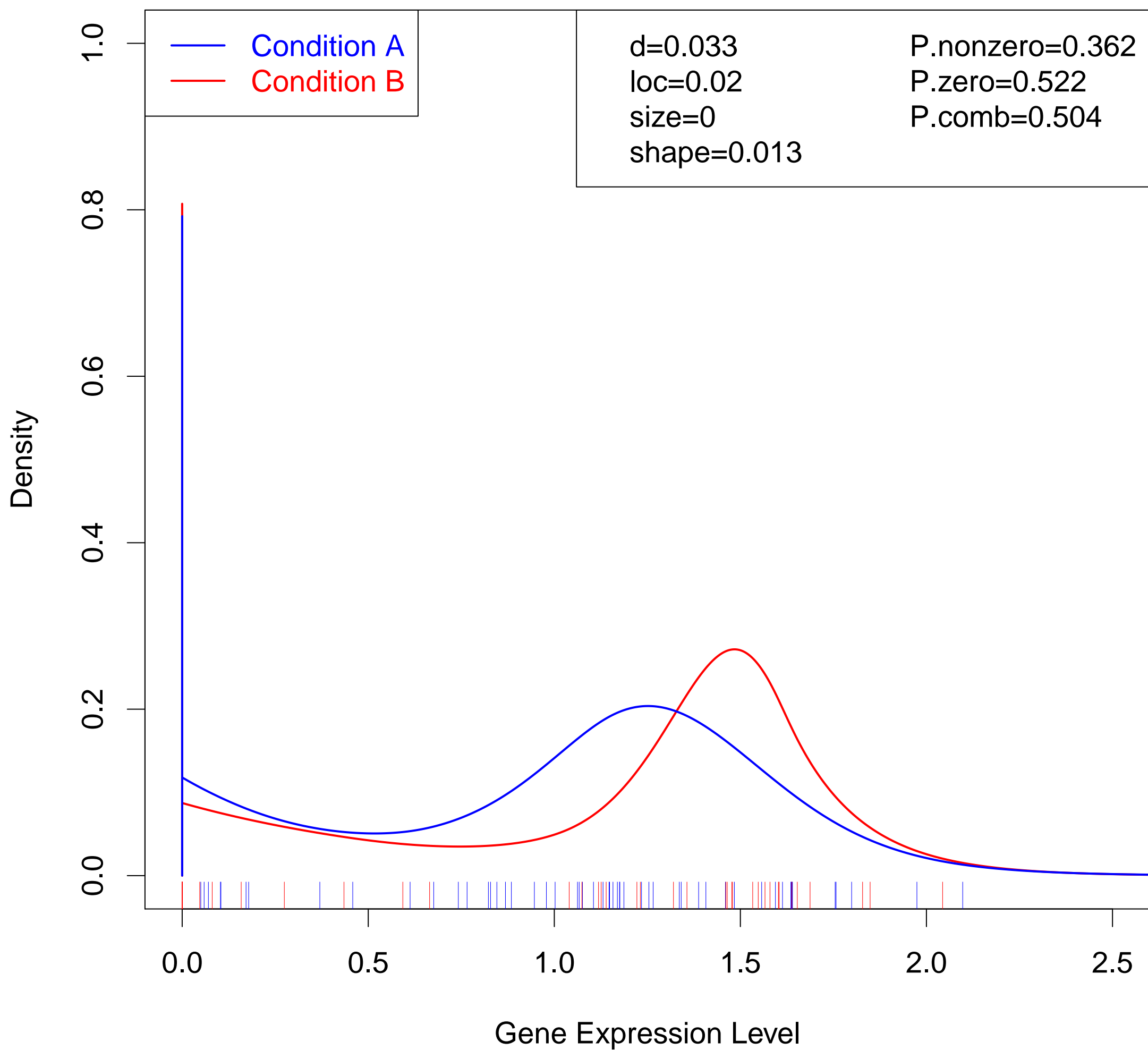

**GSTP1 (DD)**

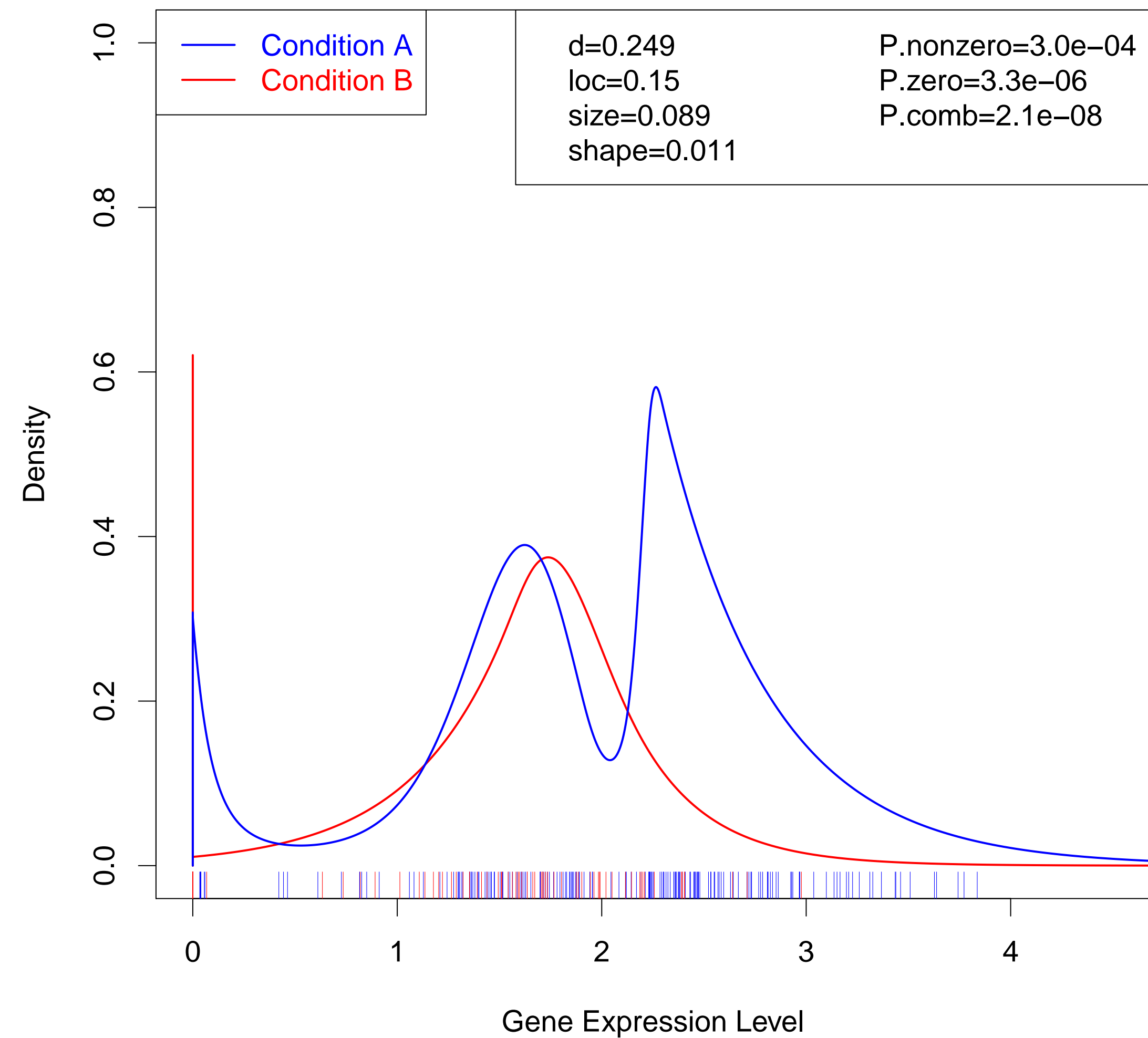

Supplement: btab226_Supplementary_Data [file btab226_supplementary_data.zip › Supplement_Revision2/Fig8.pdf]

weak degree of DD

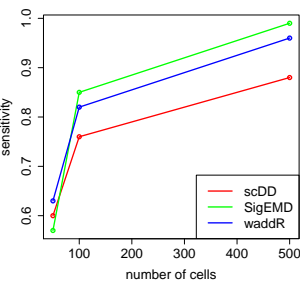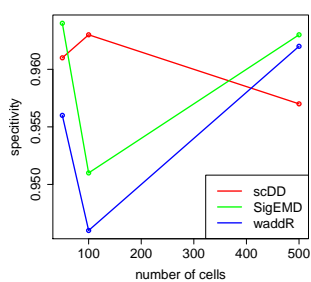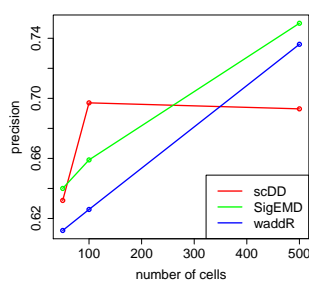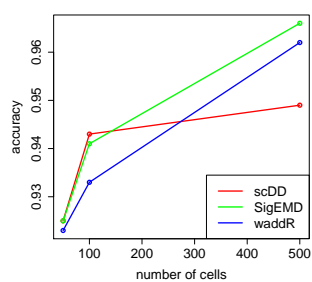

medium degree of DD

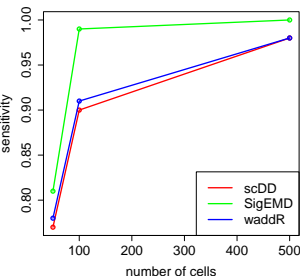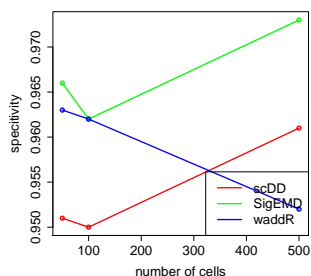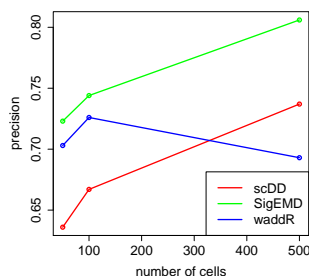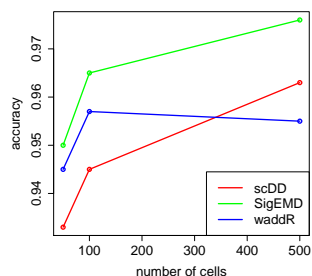

strong degree of DD

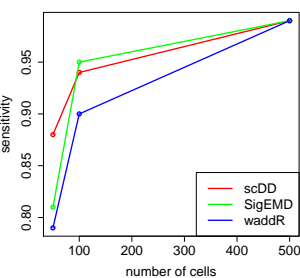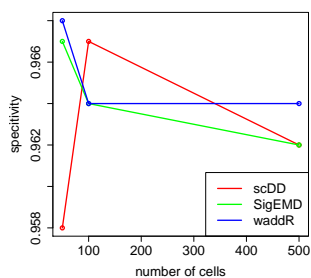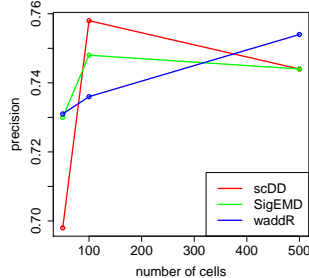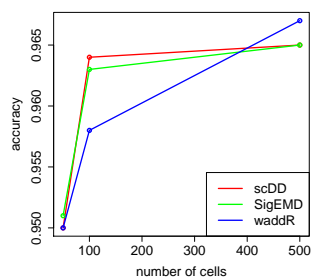

Supplement: btab226_Supplementary_Data [file btab226_supplementary_data.zip › Supplement_Revision2/Fig9.pdf]

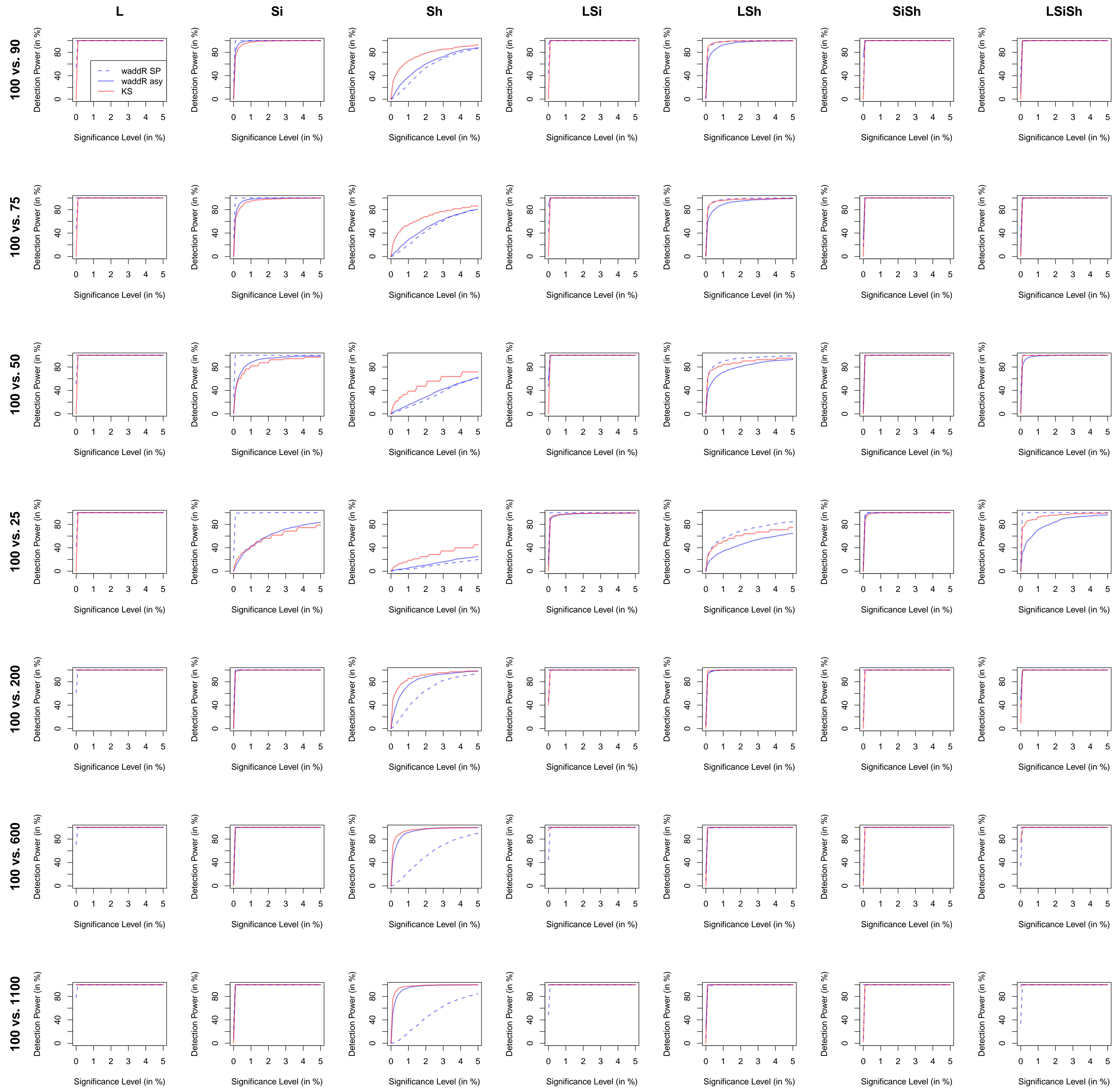

Supplement: btab226_Supplementary_Data [file btab226_supplementary_data.zip › Supplement_Revision2/FigA1.pdf]

100 vs. 90

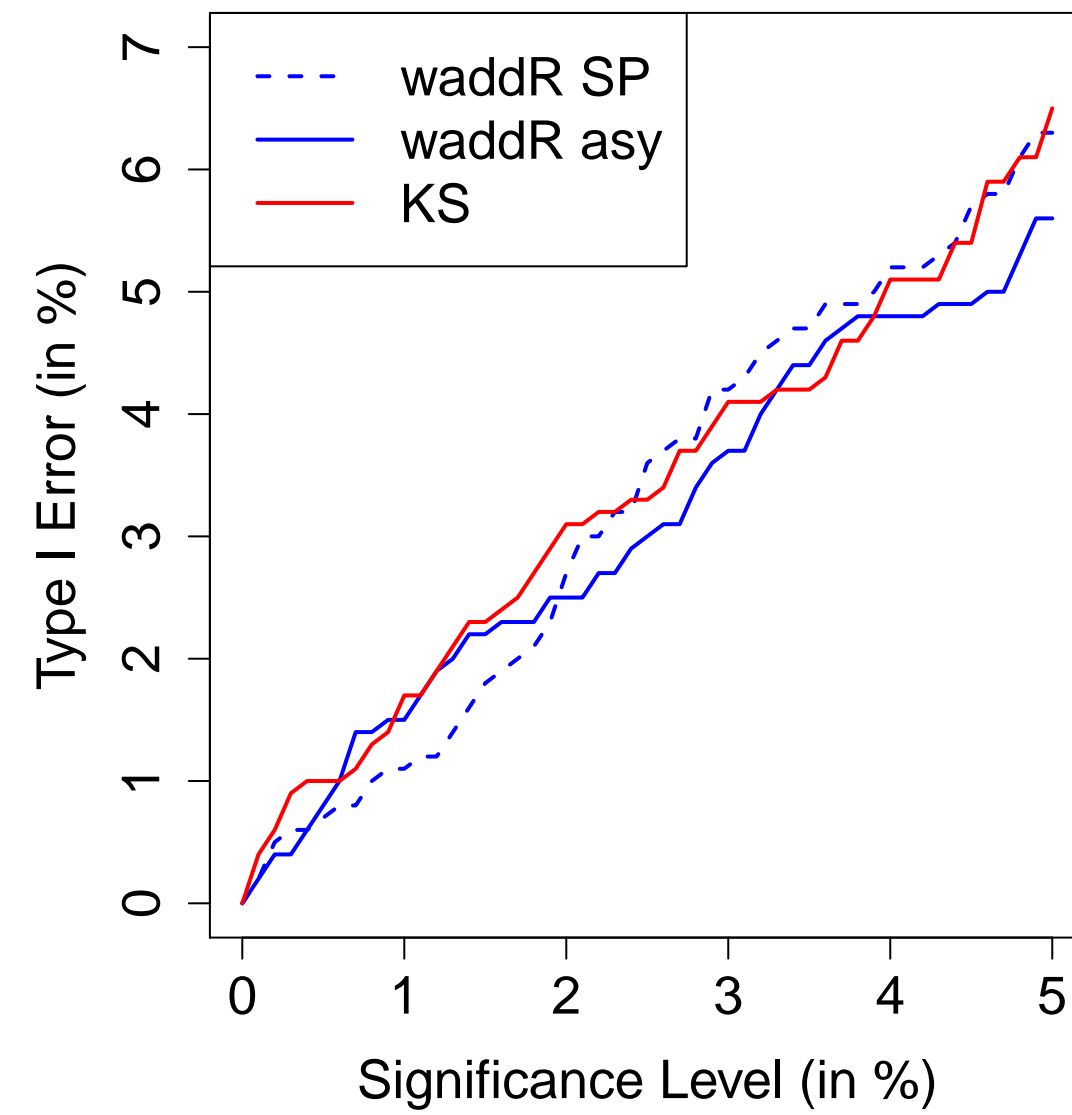

100 vs. 75

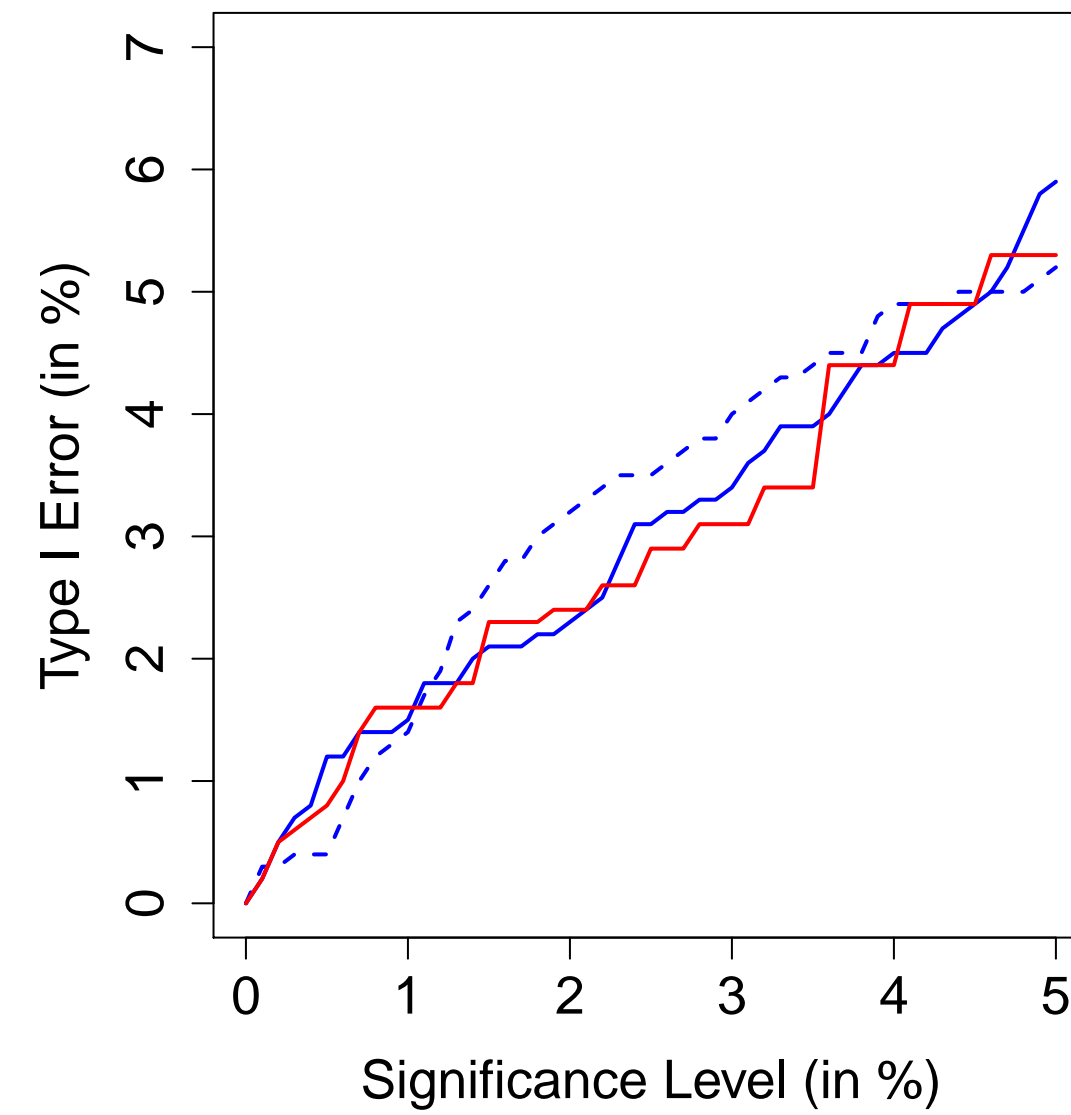

100 vs. 50

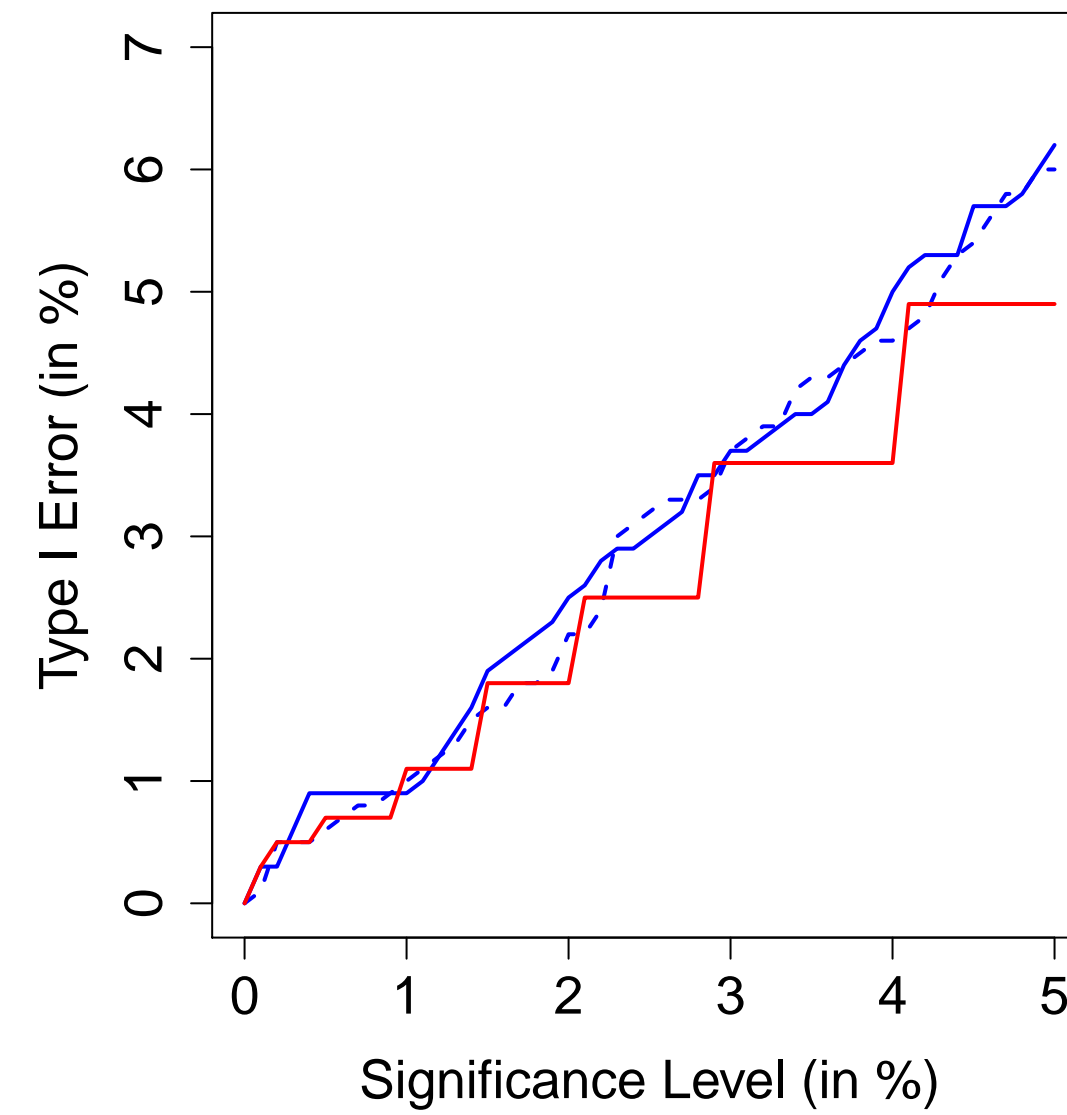

100 vs. 25

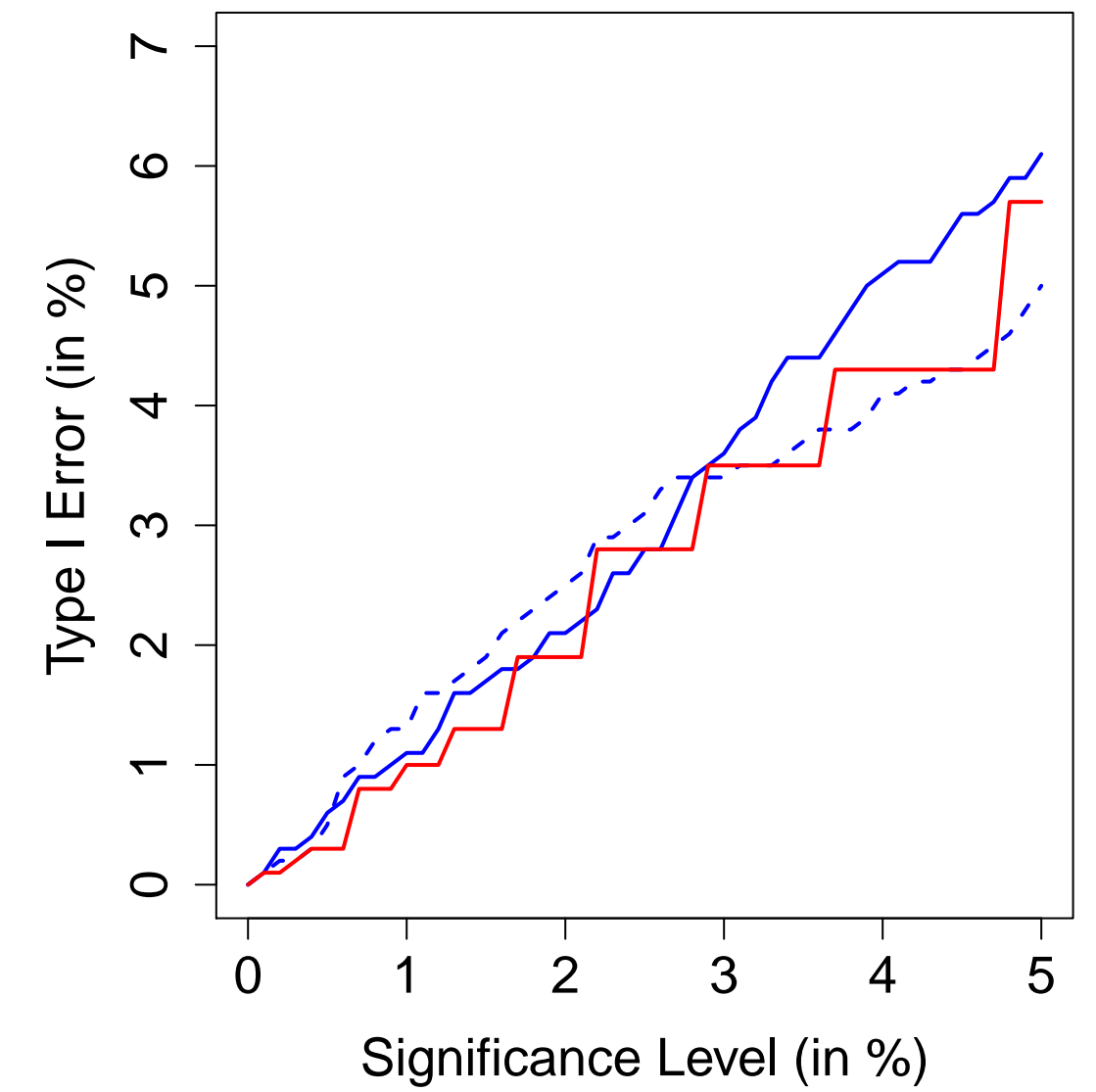

100 vs. 200

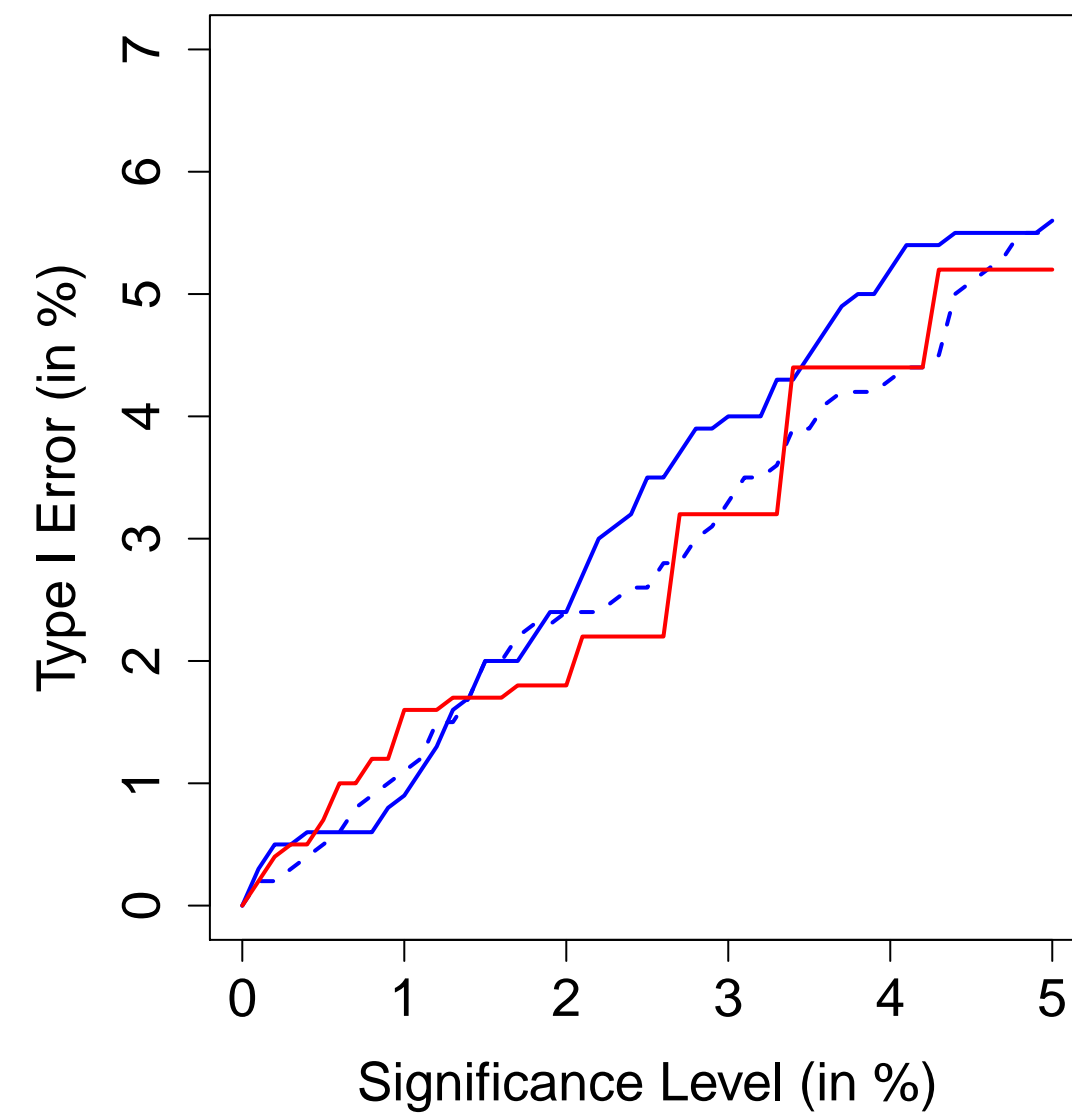

100 vs. 600

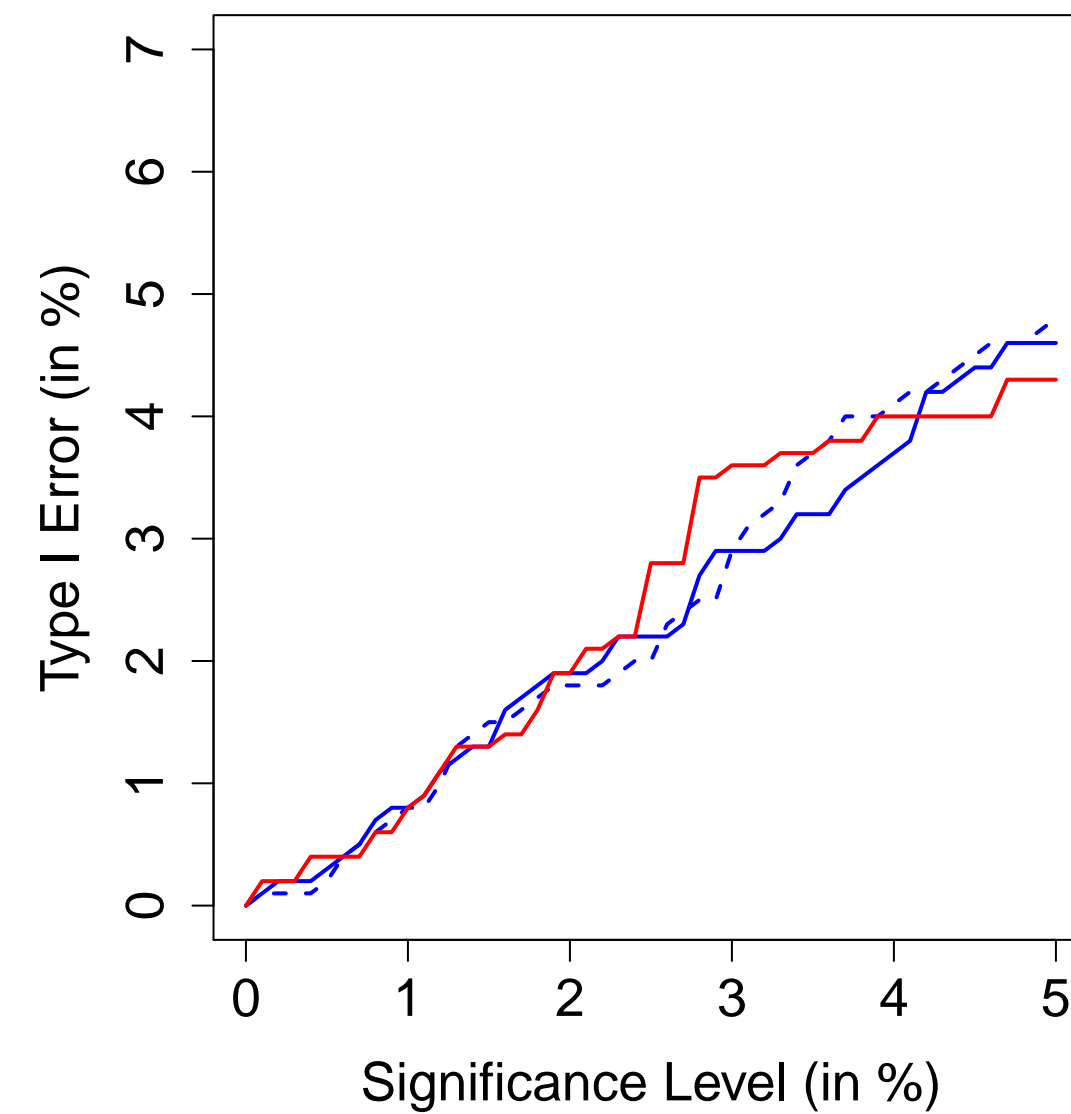

100 vs. 1100

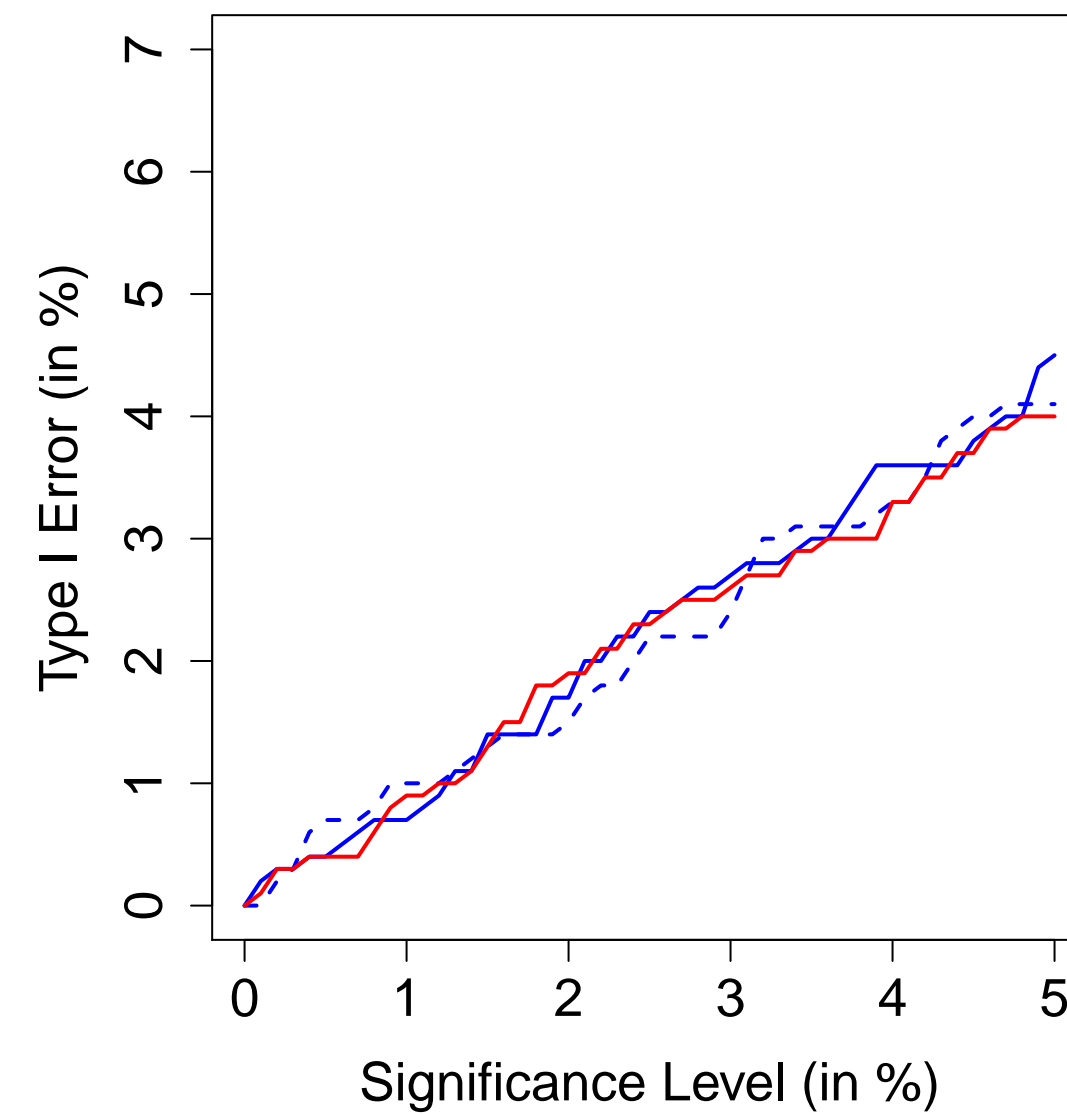

Supplement: btab226_Supplementary_Data [file btab226_supplementary_data.zip › Supplement_Revision2/FigA2.pdf]

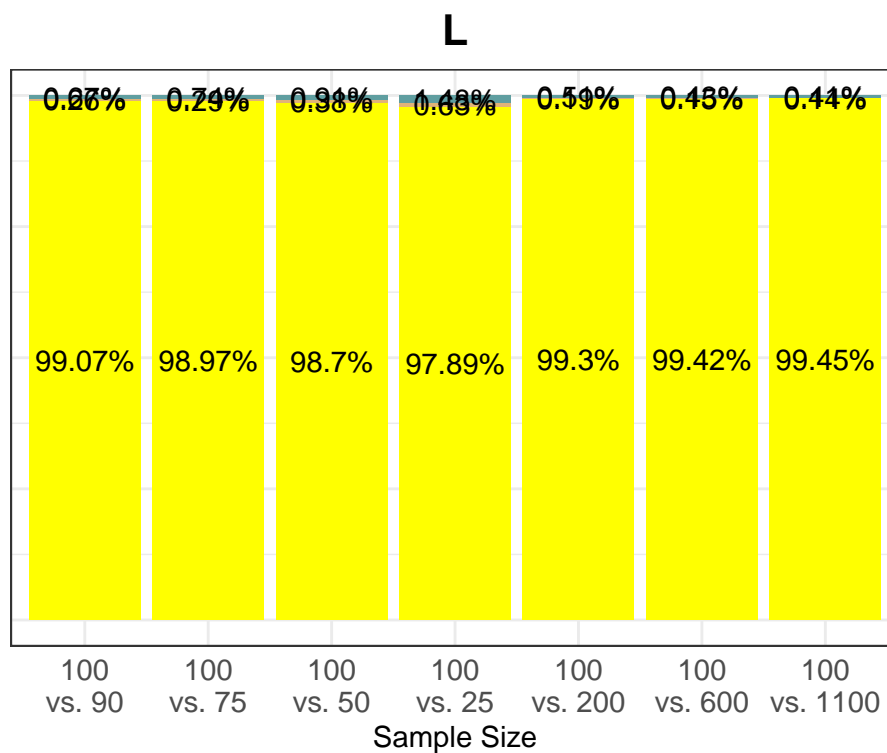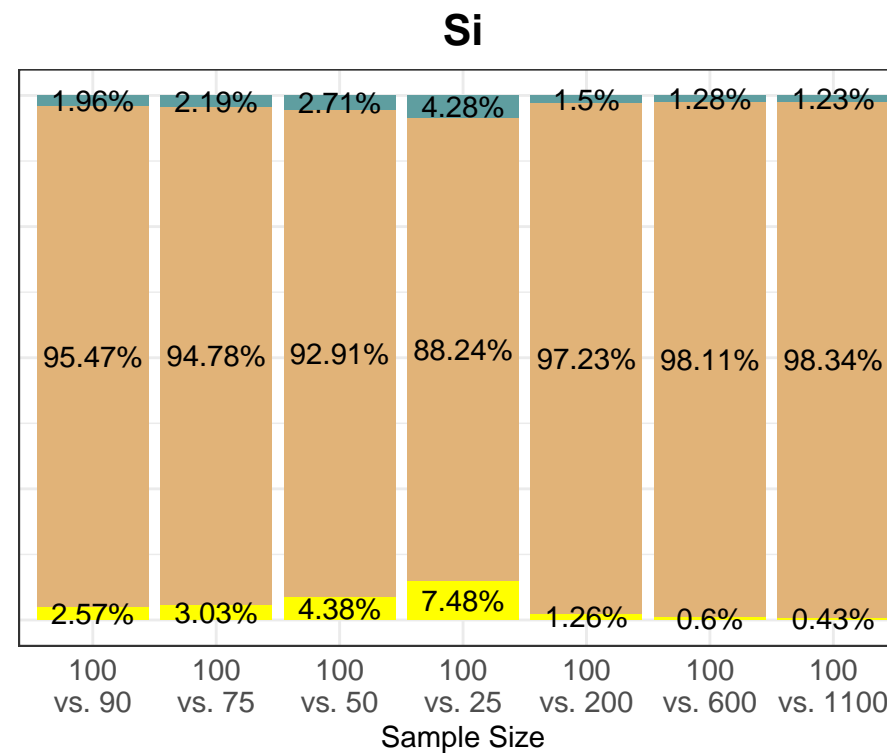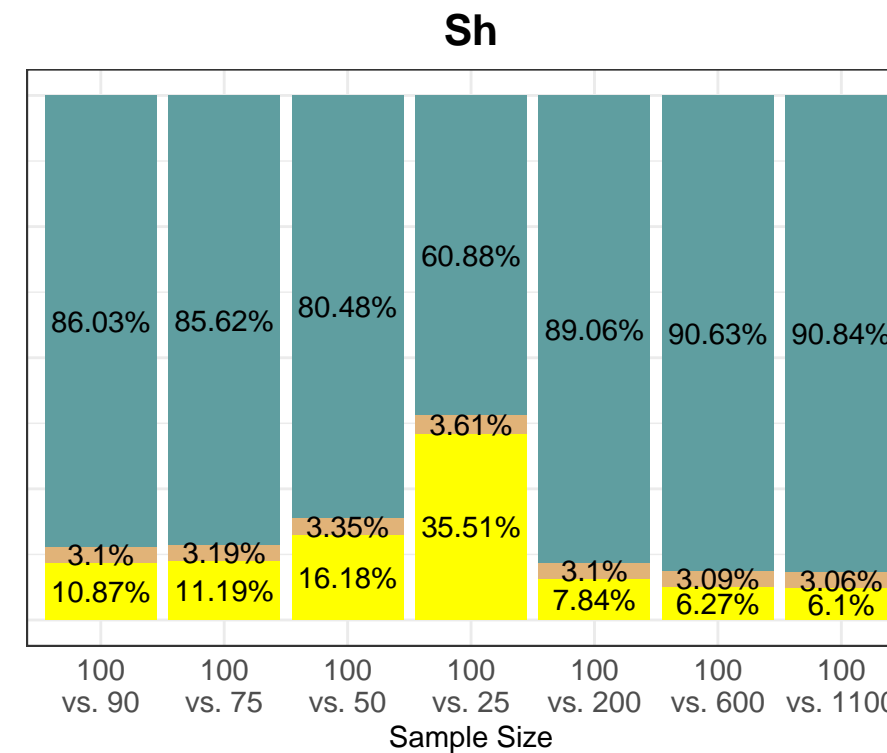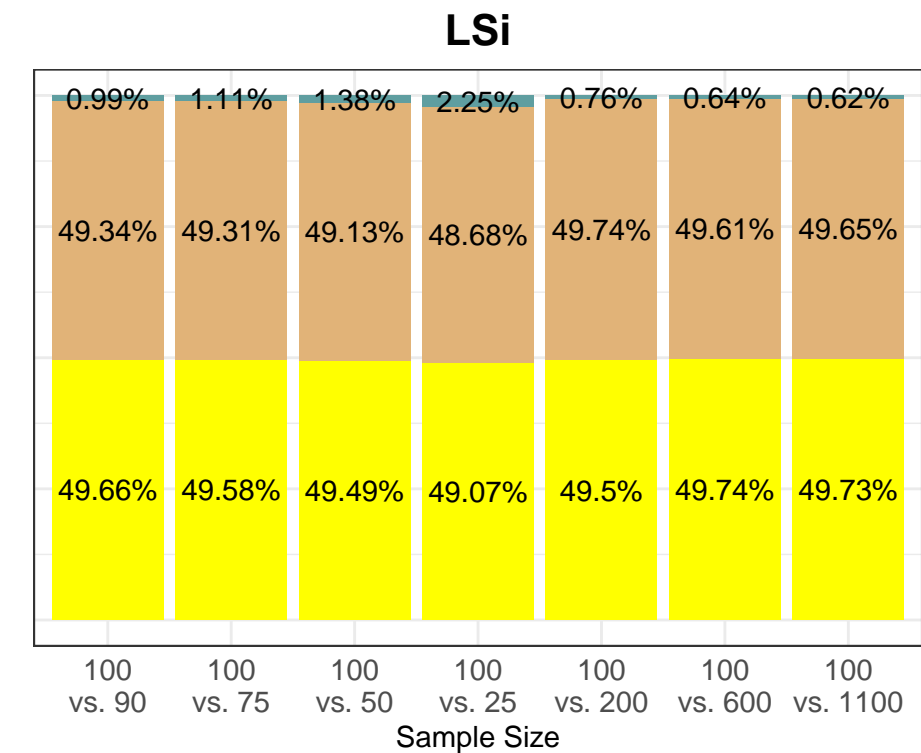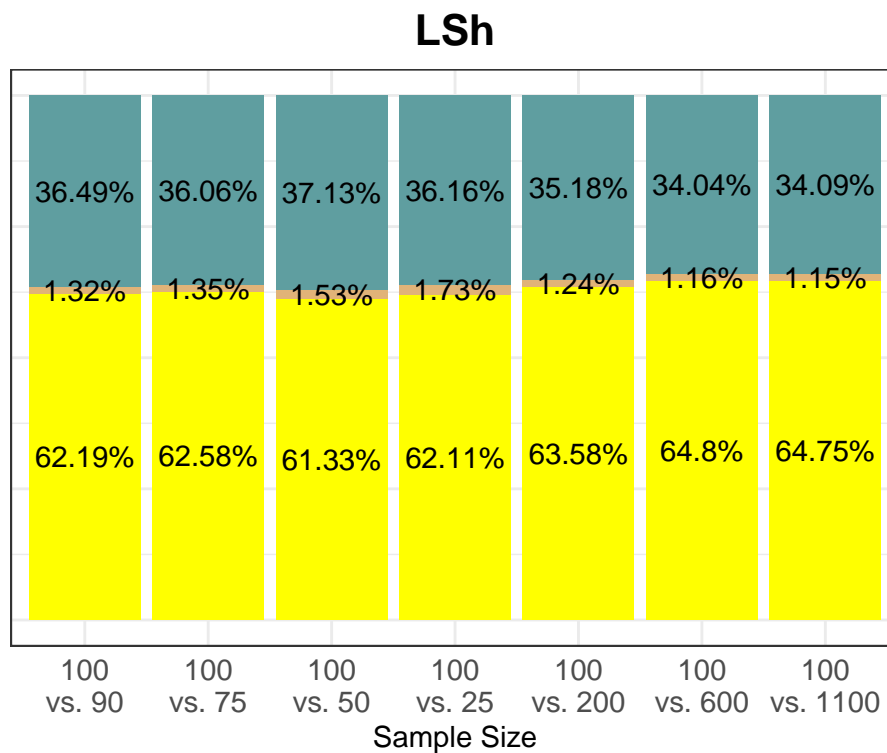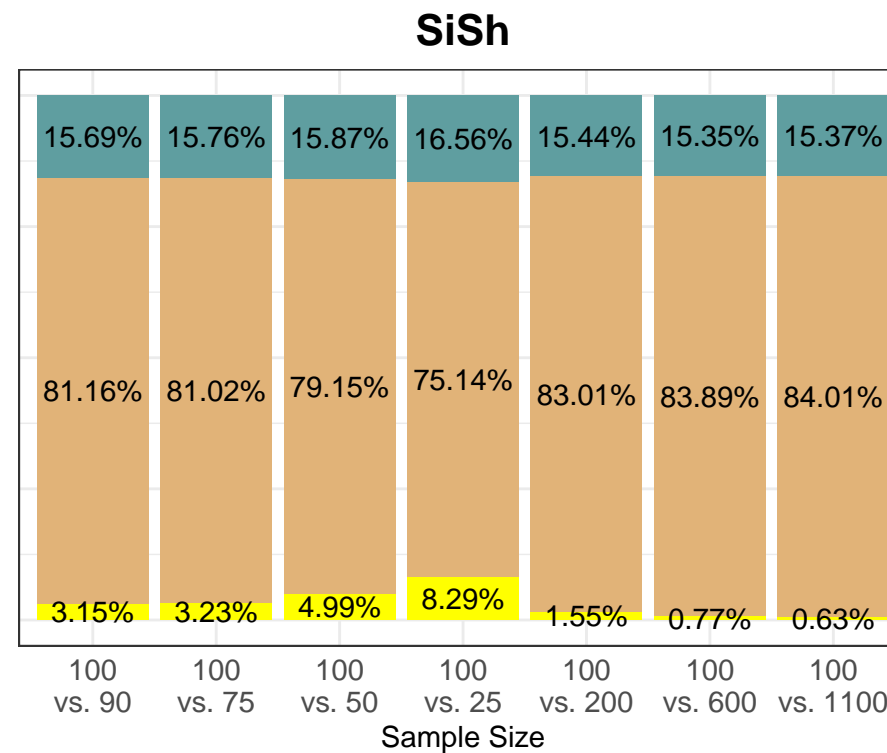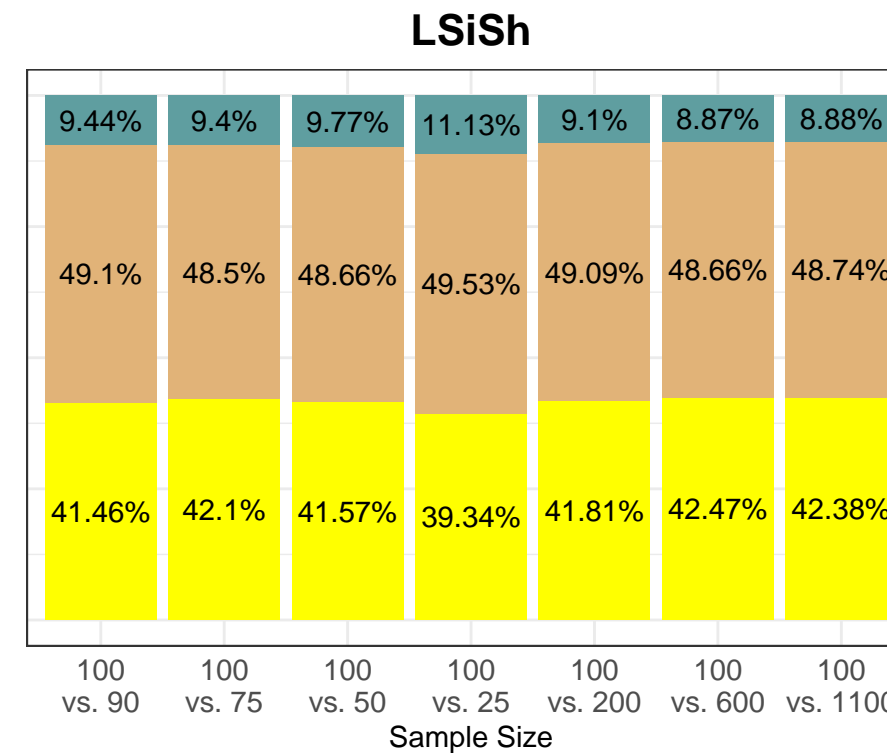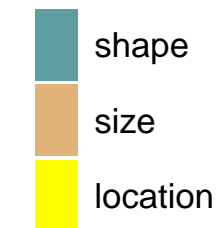

Supplement: btab226_Supplementary_Data [file btab226_supplementary_data.zip › Supplement_Revision2/FigA3.pdf]

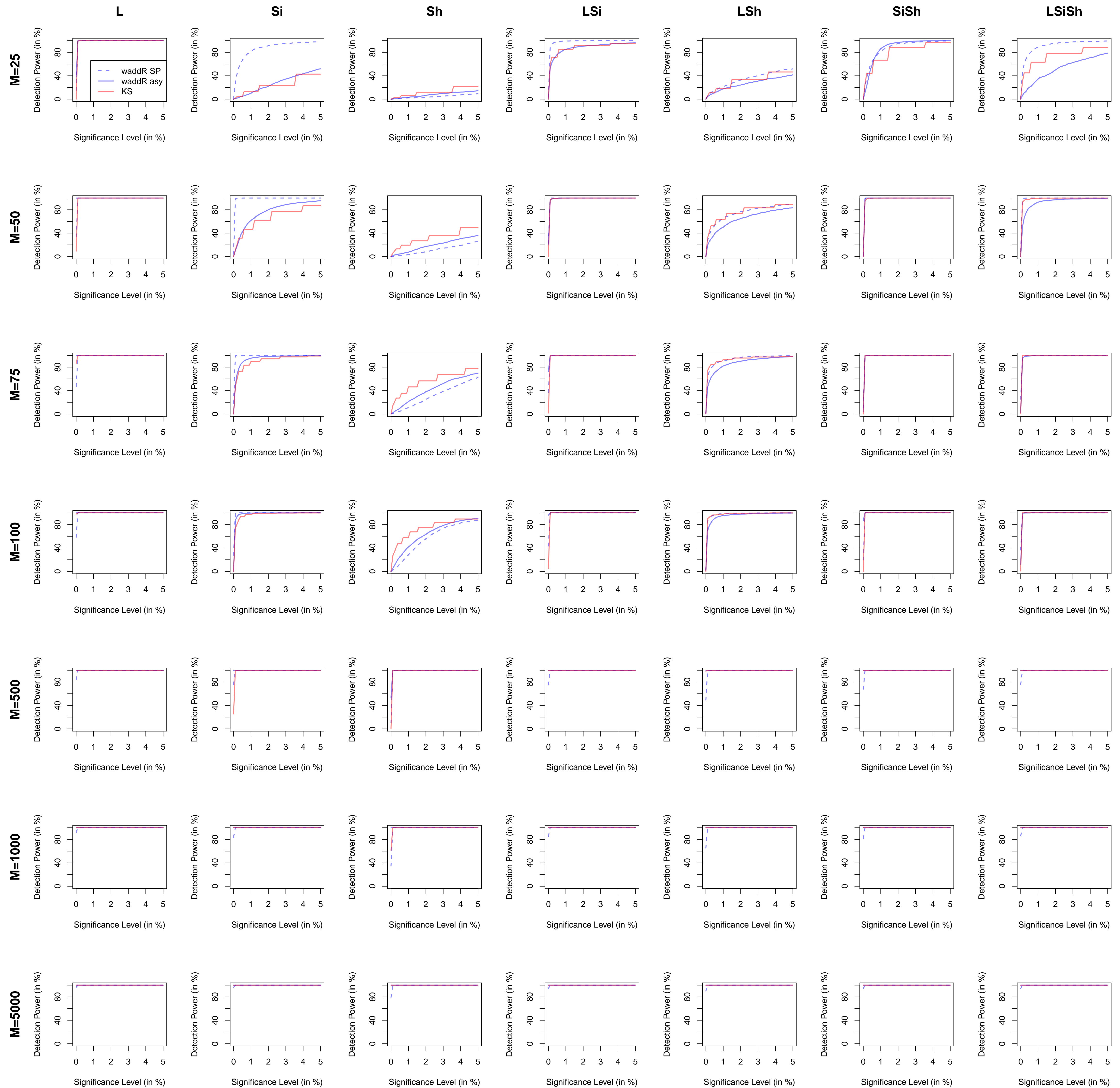

Supplement: btab226_Supplementary_Data [file btab226_supplementary_data.zip › Supplement_Revision2/FigA4.pdf]

**M=25**

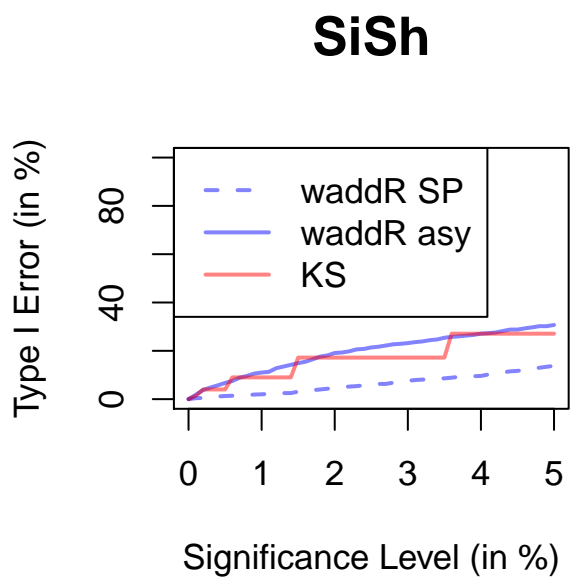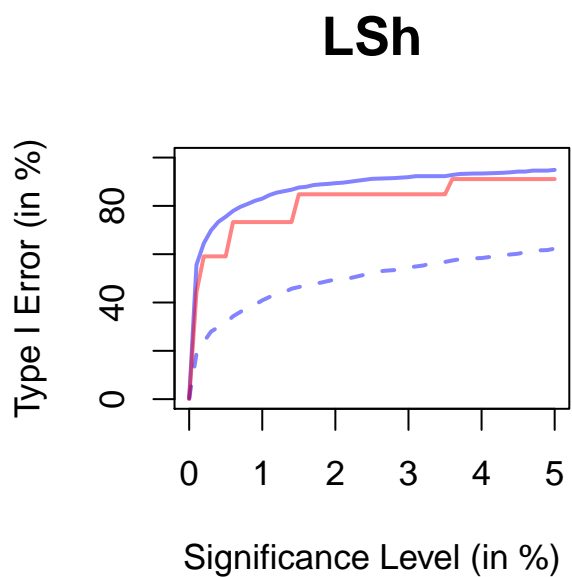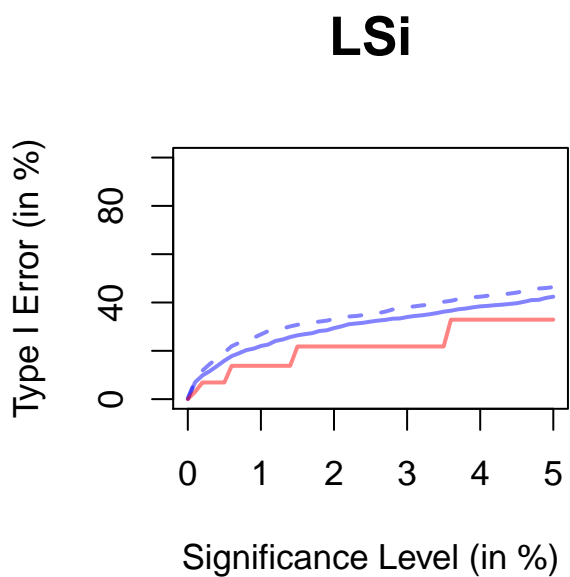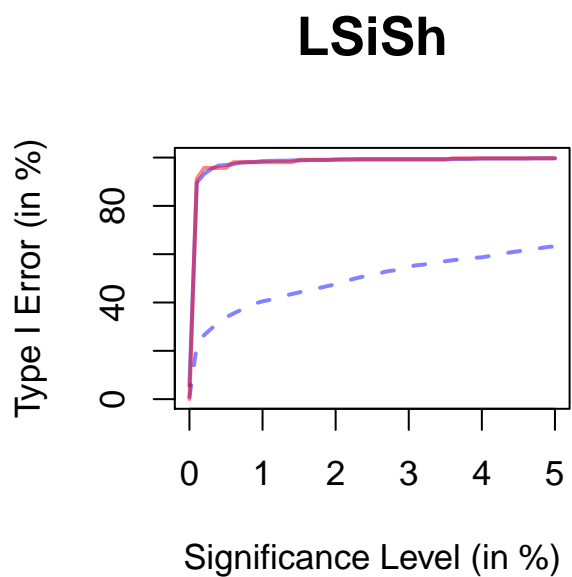

**M=50**

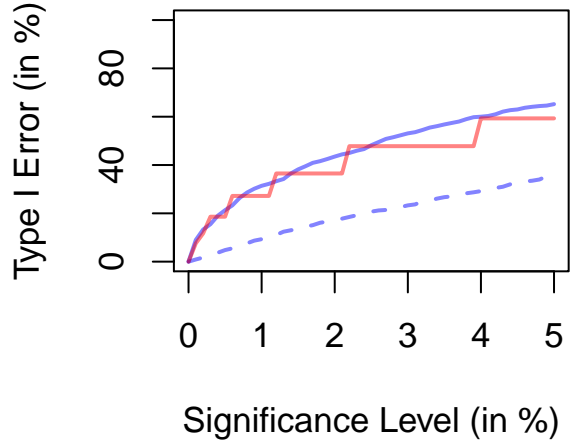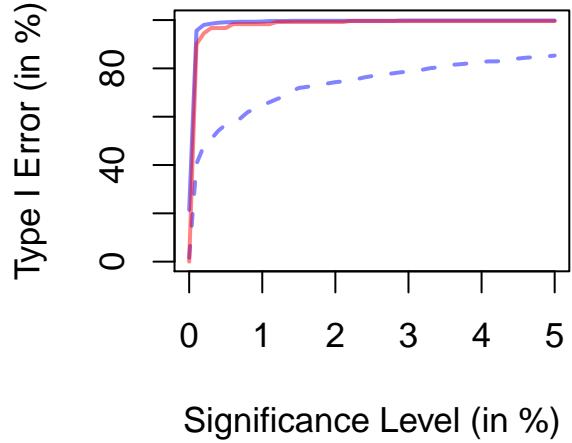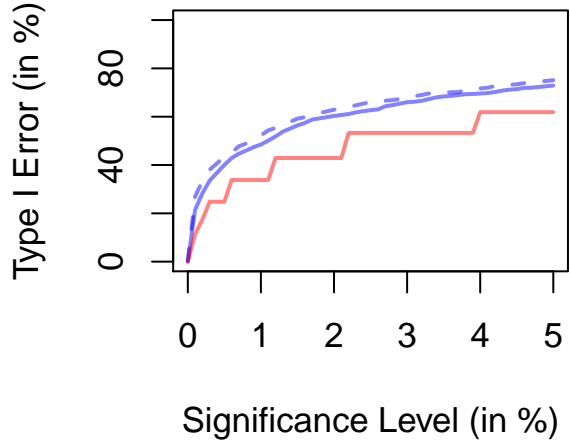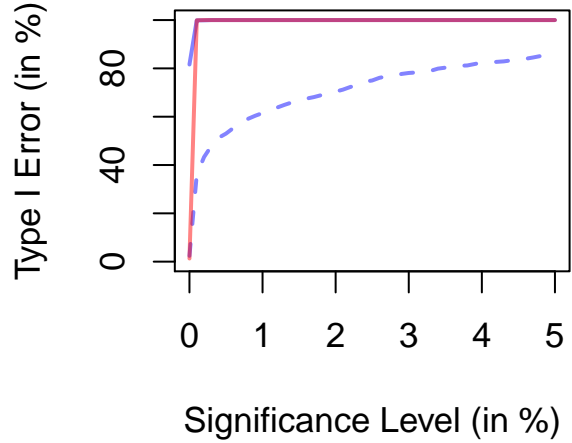

**M=75**

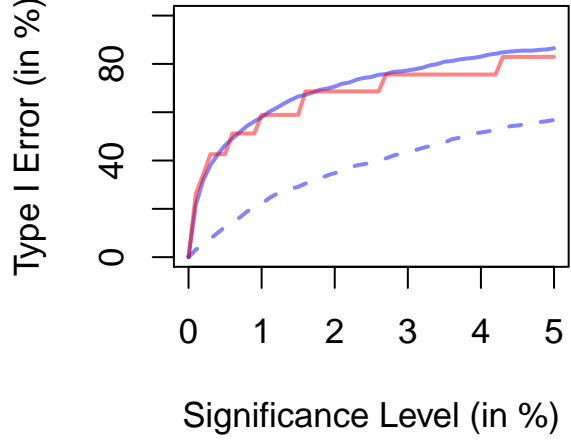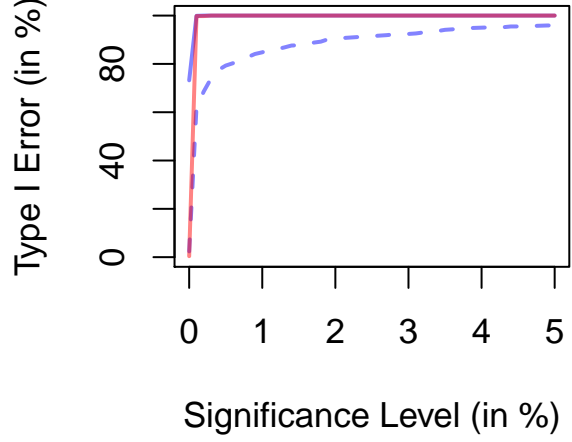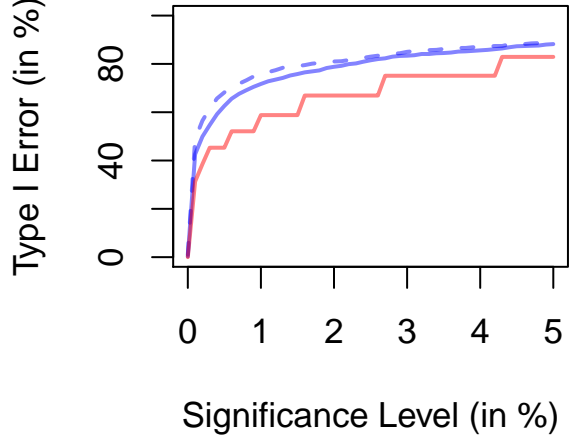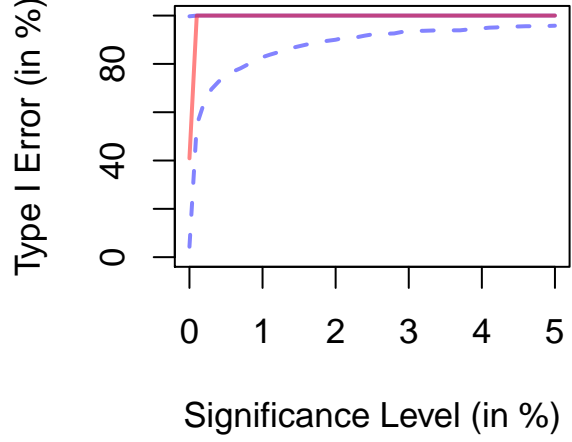

**M=100**

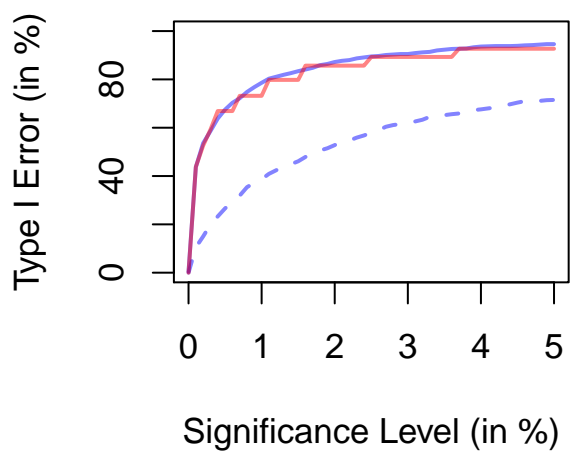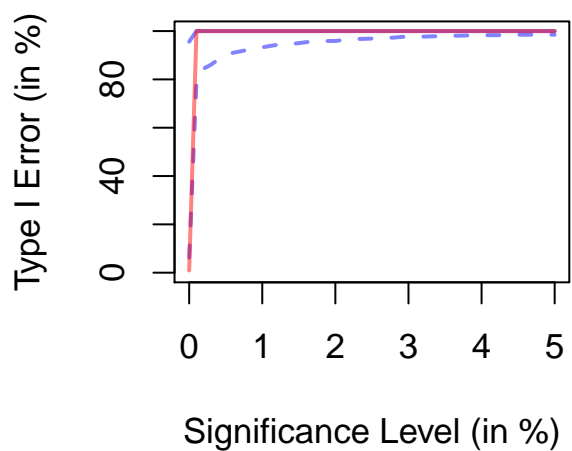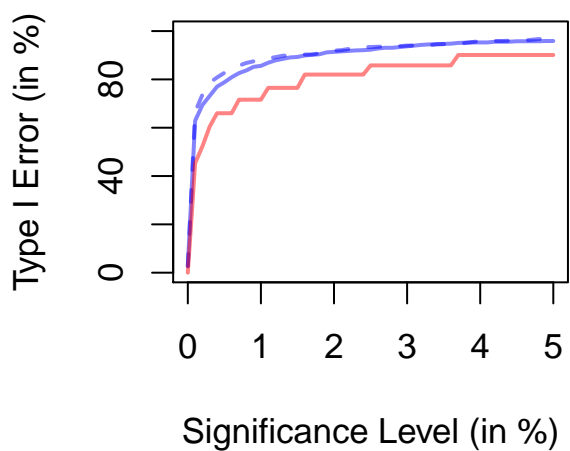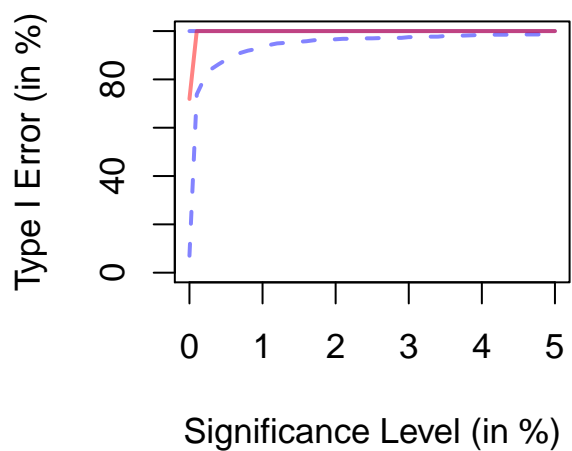

**M=500**

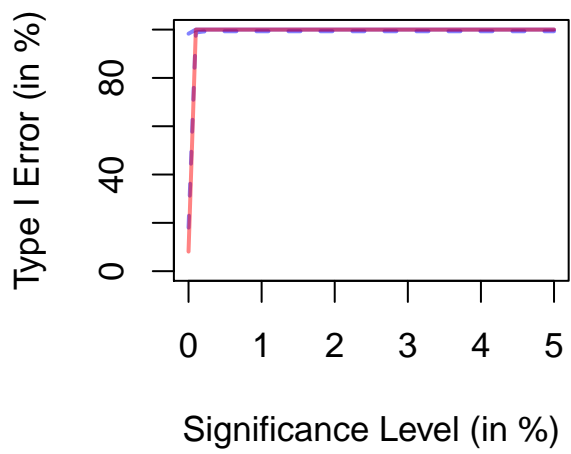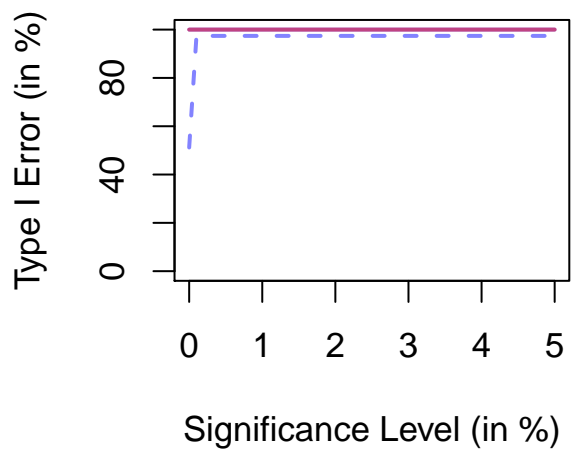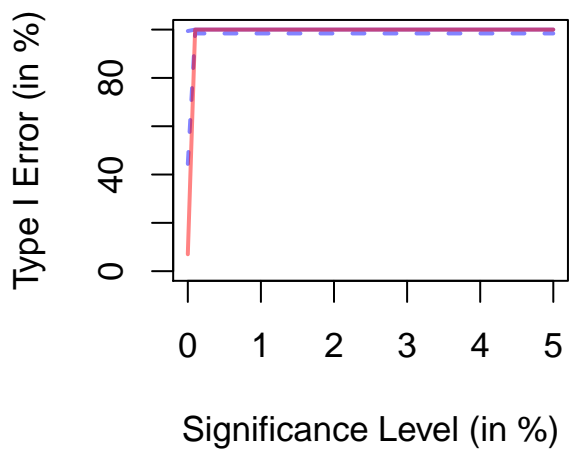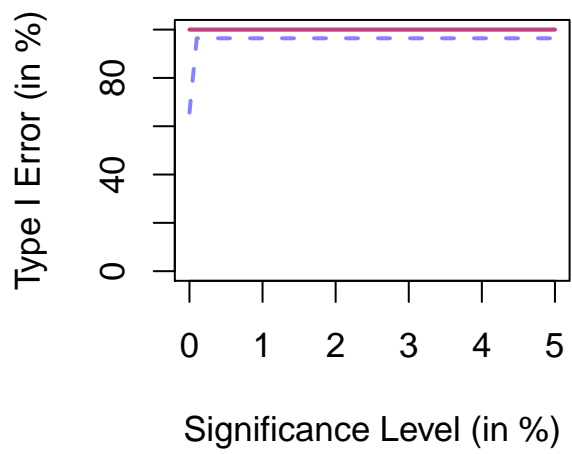

**M=1000**

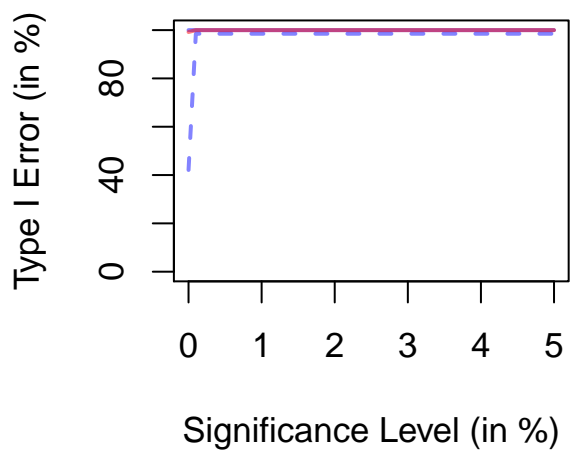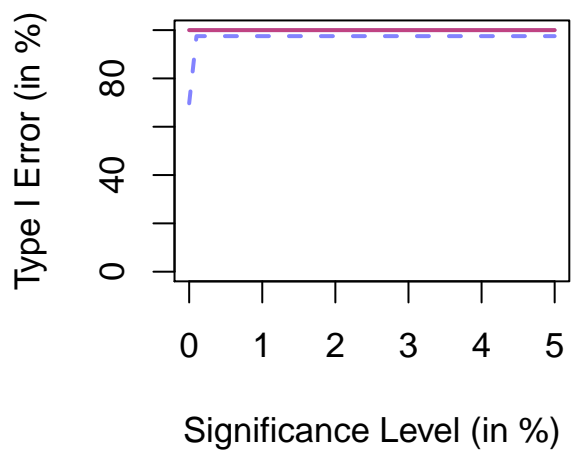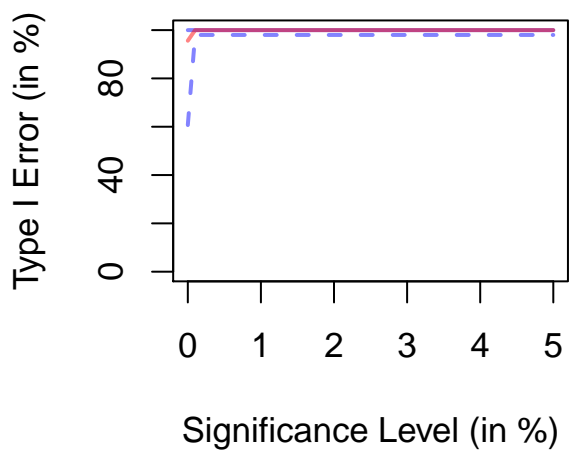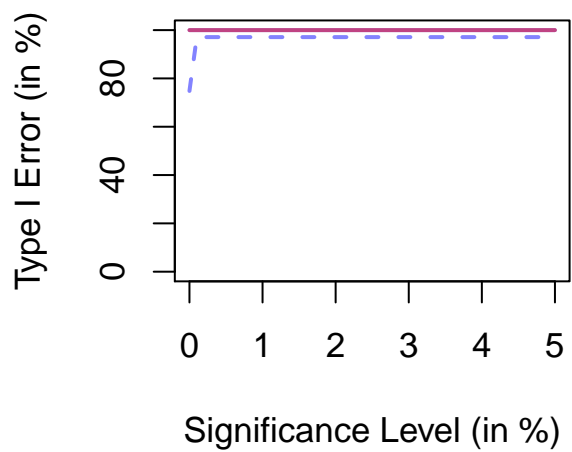

**M=5000**

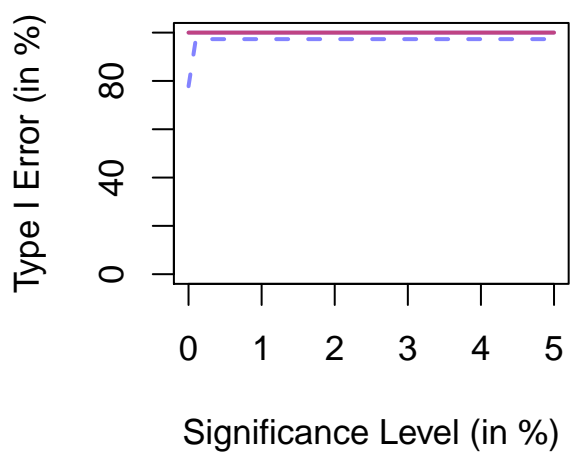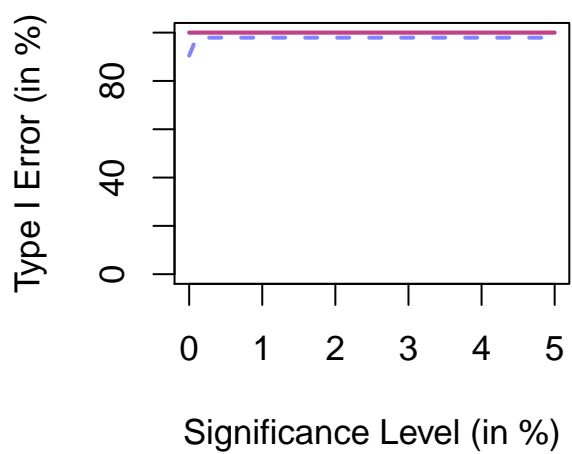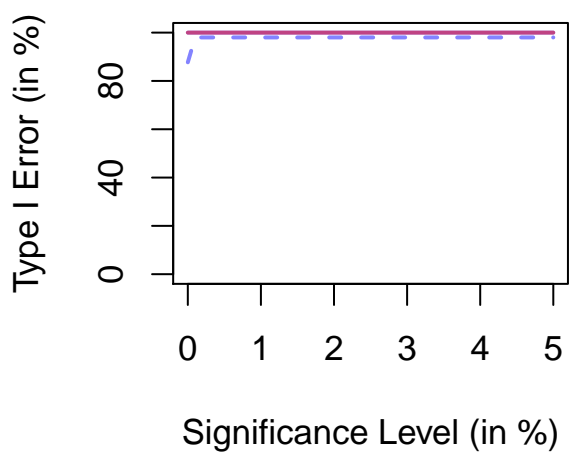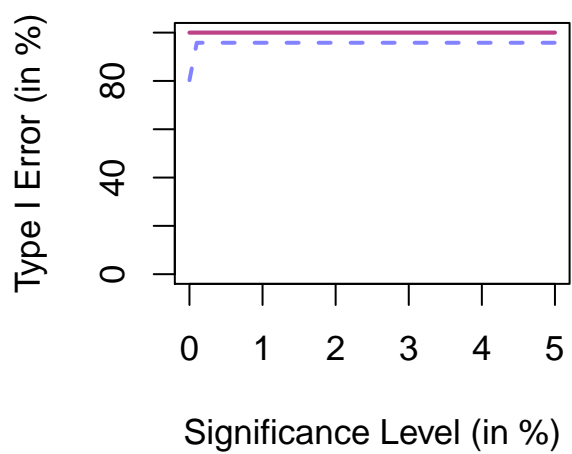

Supplement: btab226_Supplementary_Data [file btab226_supplementary_data.zip › Supplement_Revision2/FigA5.pdf]

**M=25**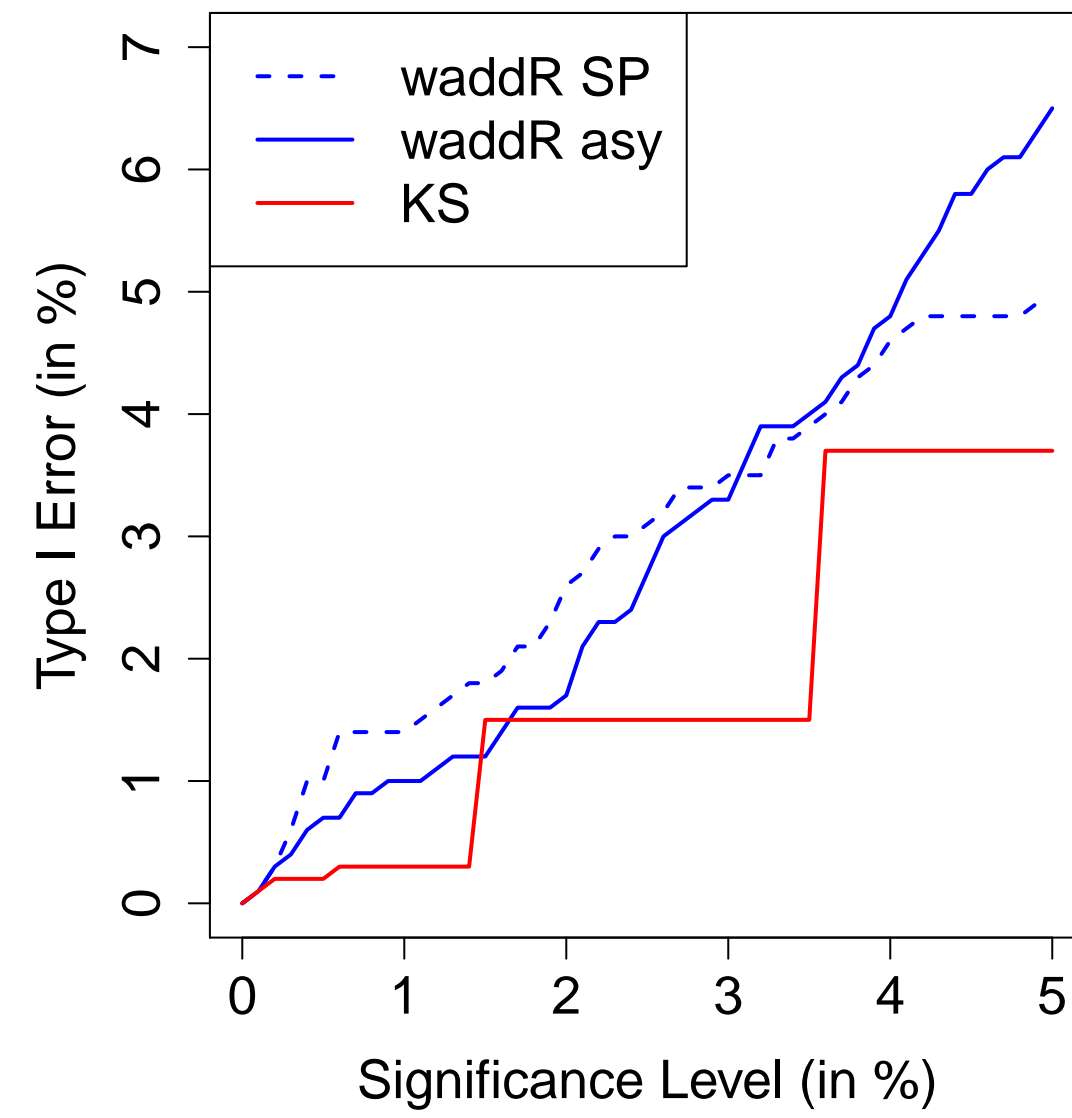**M=50**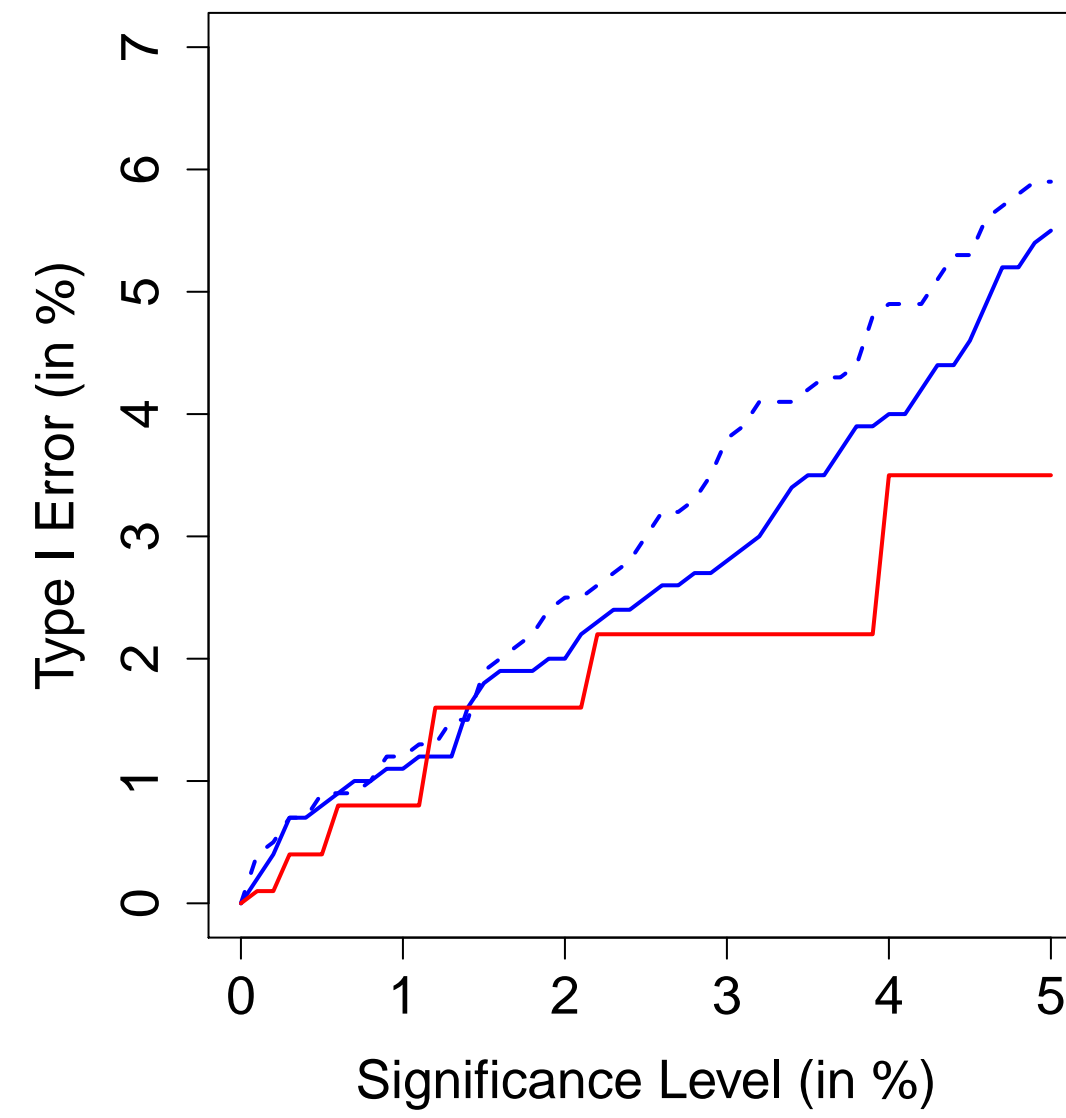**M=75**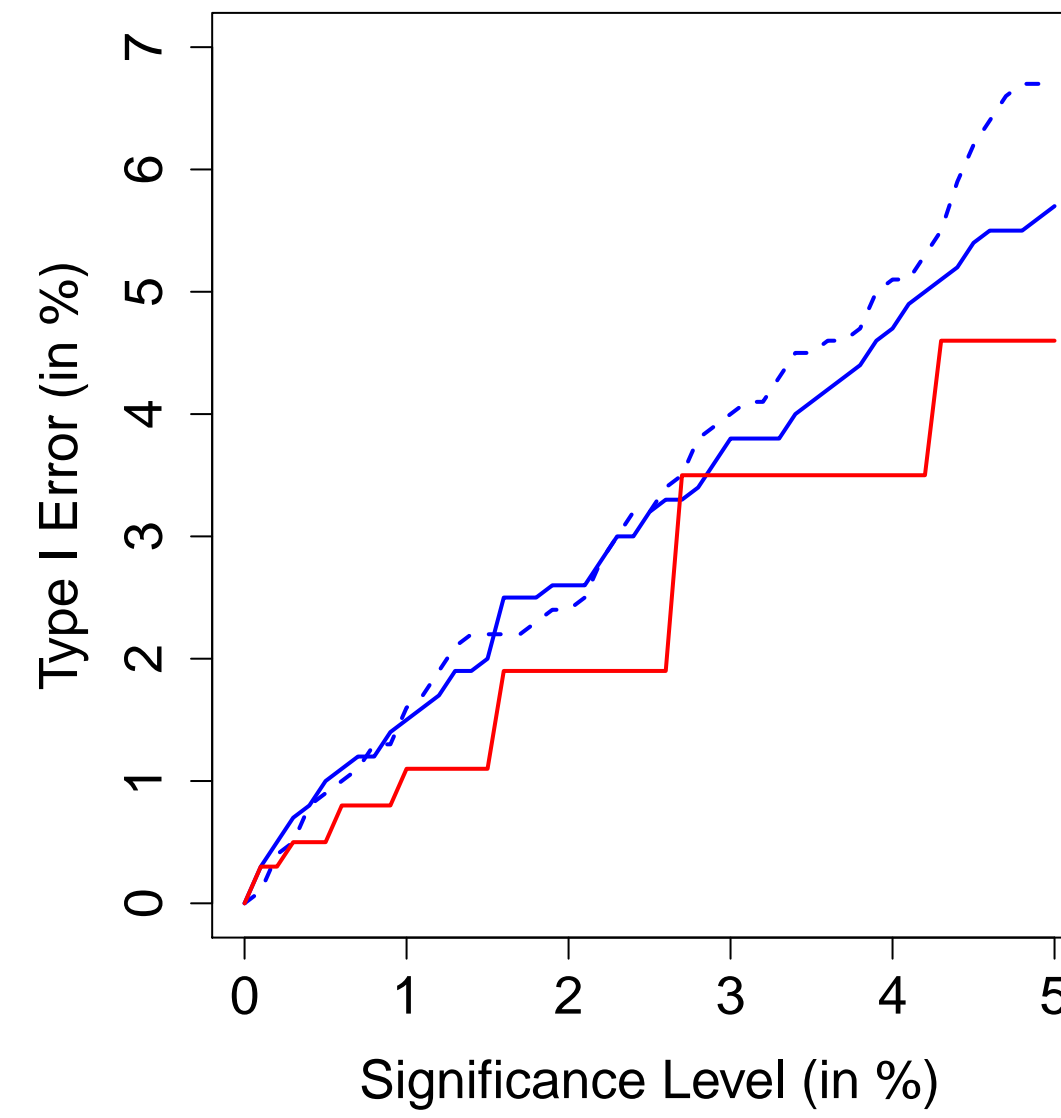**M=100**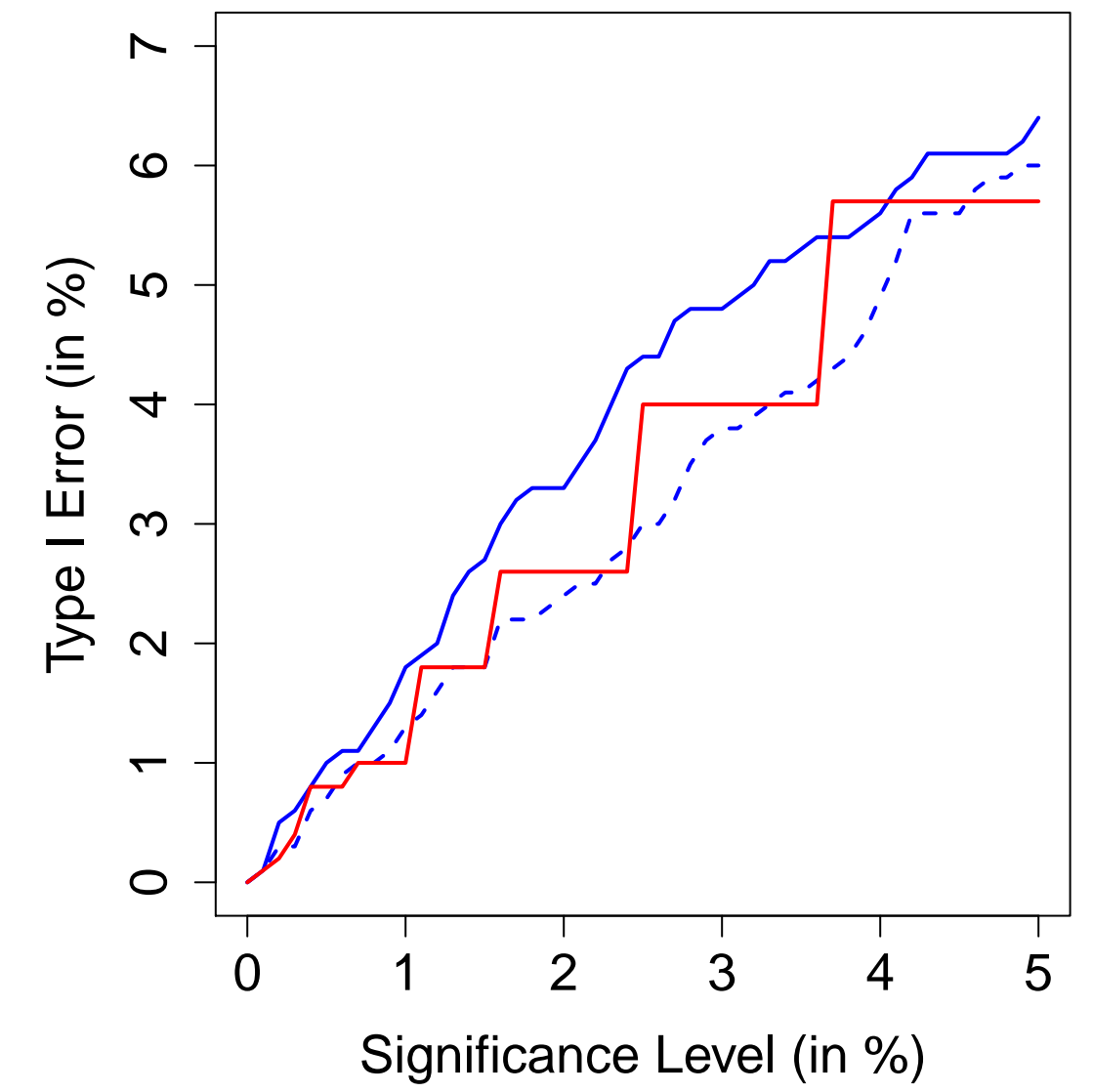**M=500**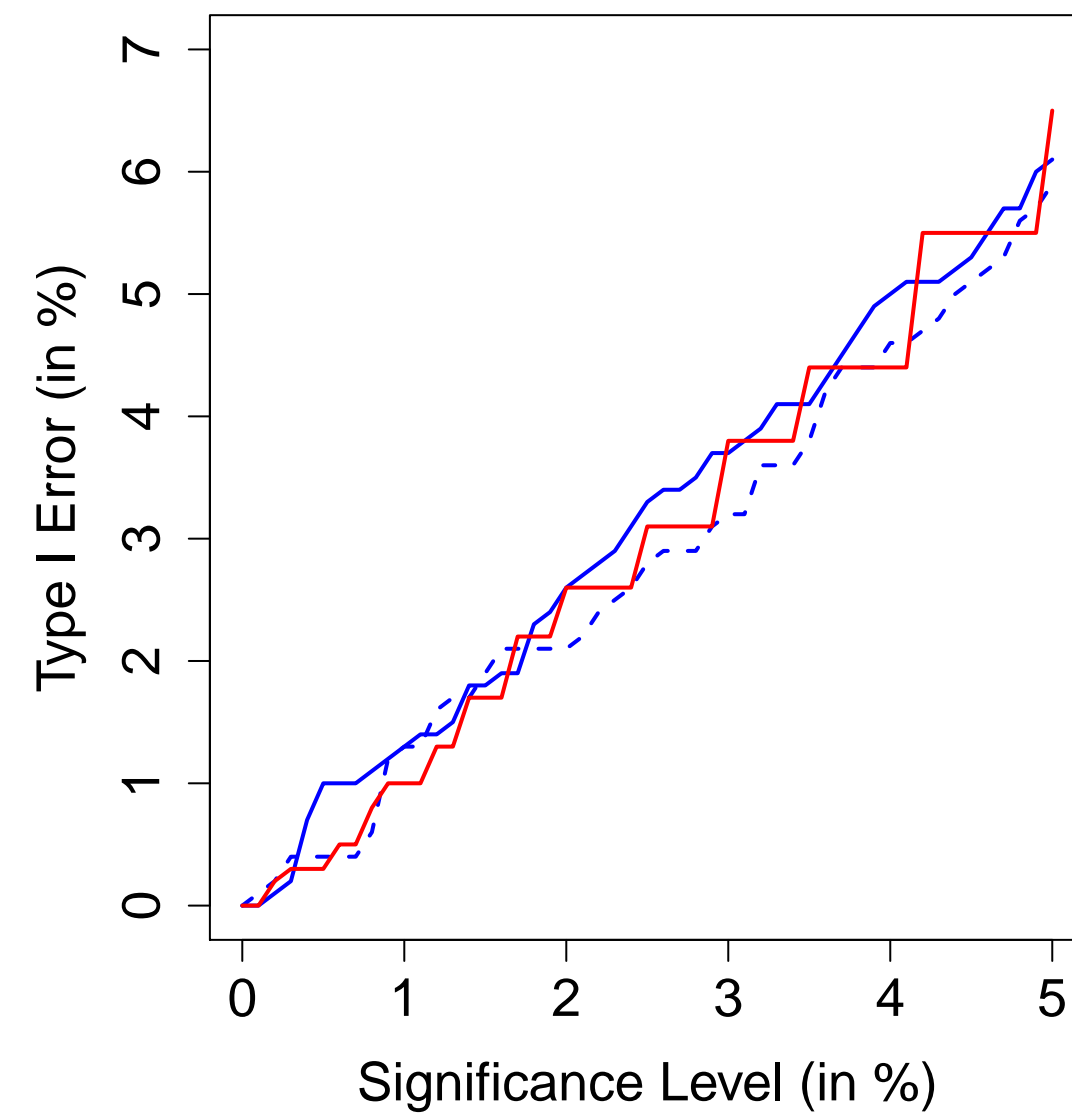**M=1000**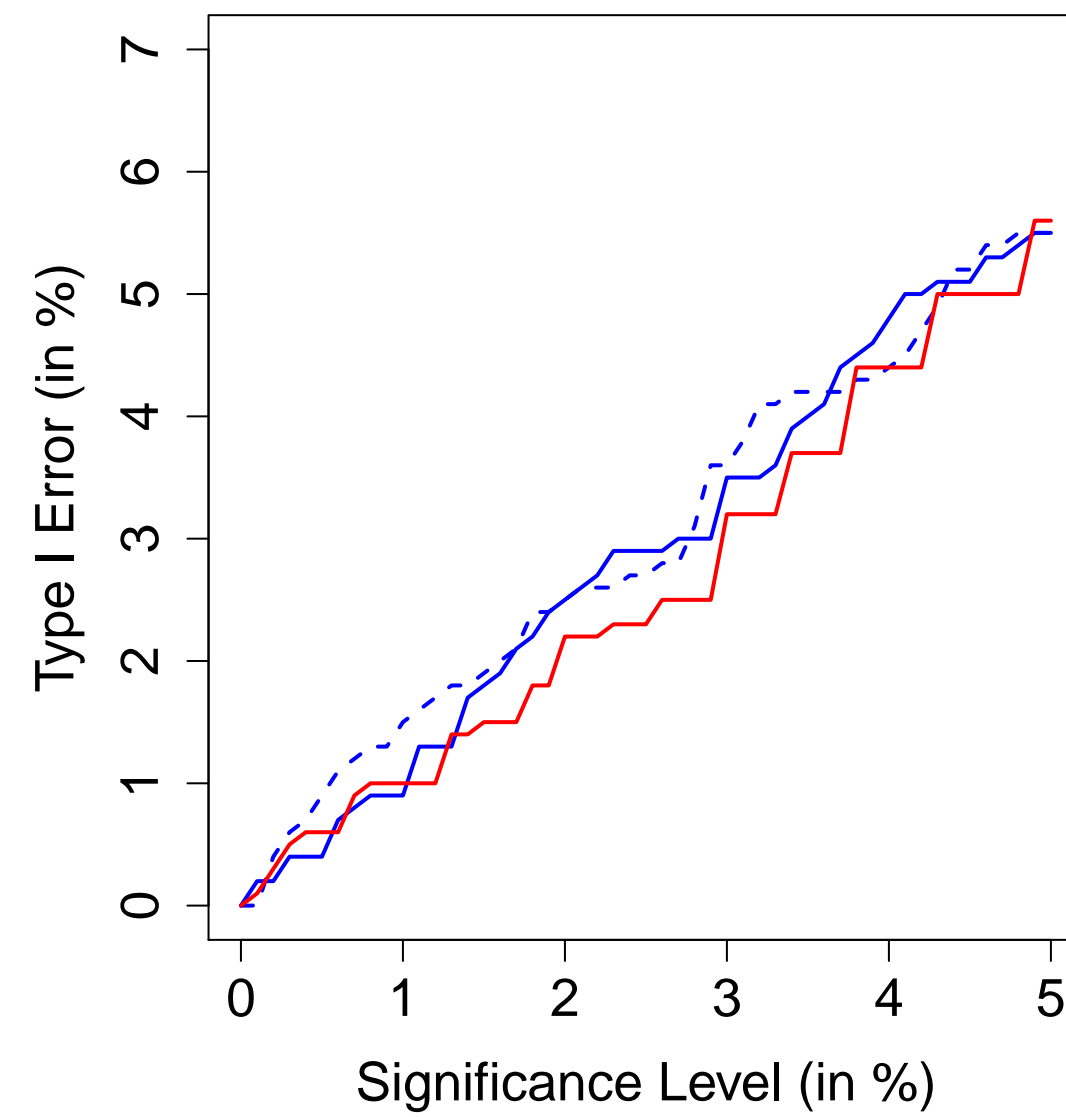**M=5000**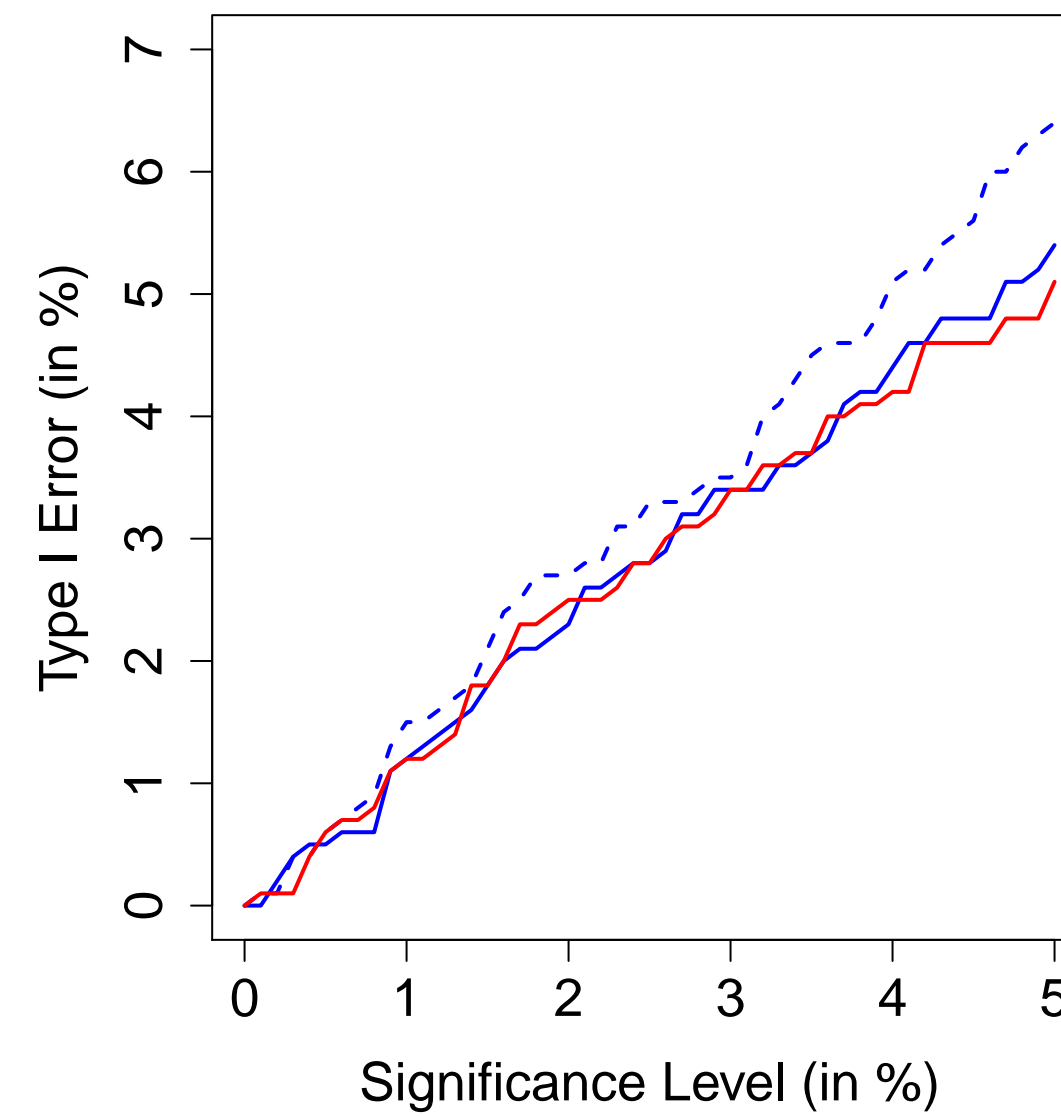

Supplement: btab226_Supplementary_Data [file btab226_supplementary_data.zip › Supplement_Revision2/FigA6.pdf]

SiSh

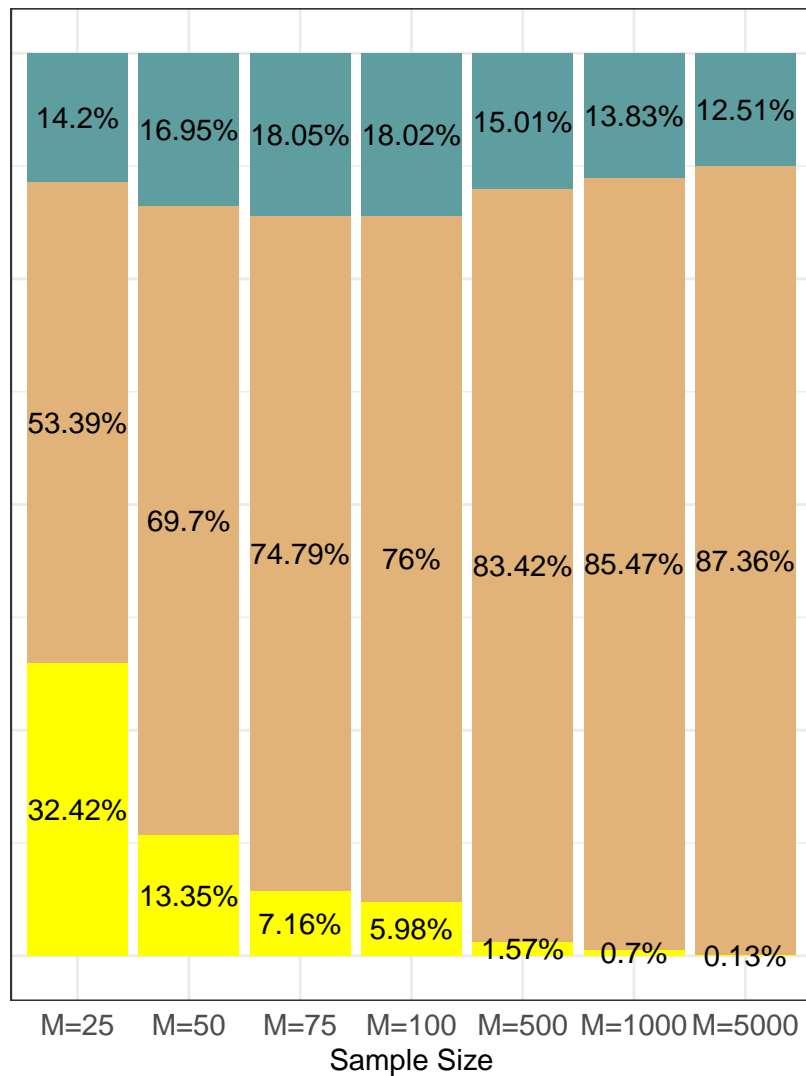

LSh

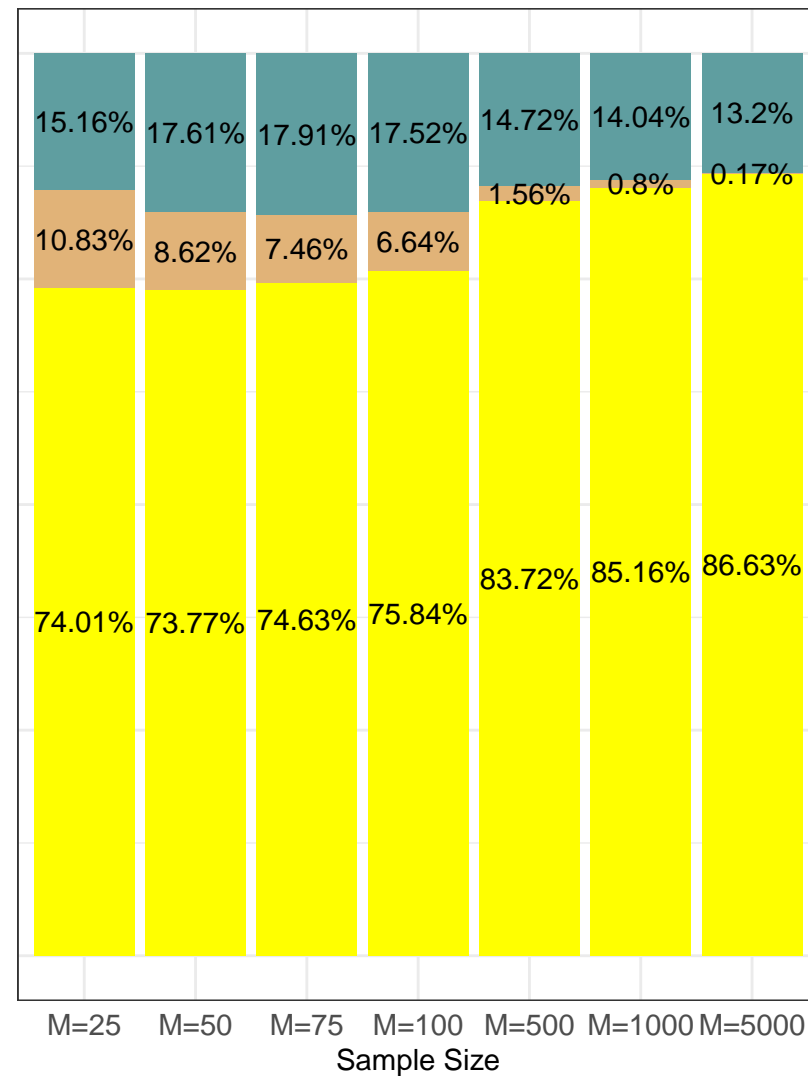

LSi

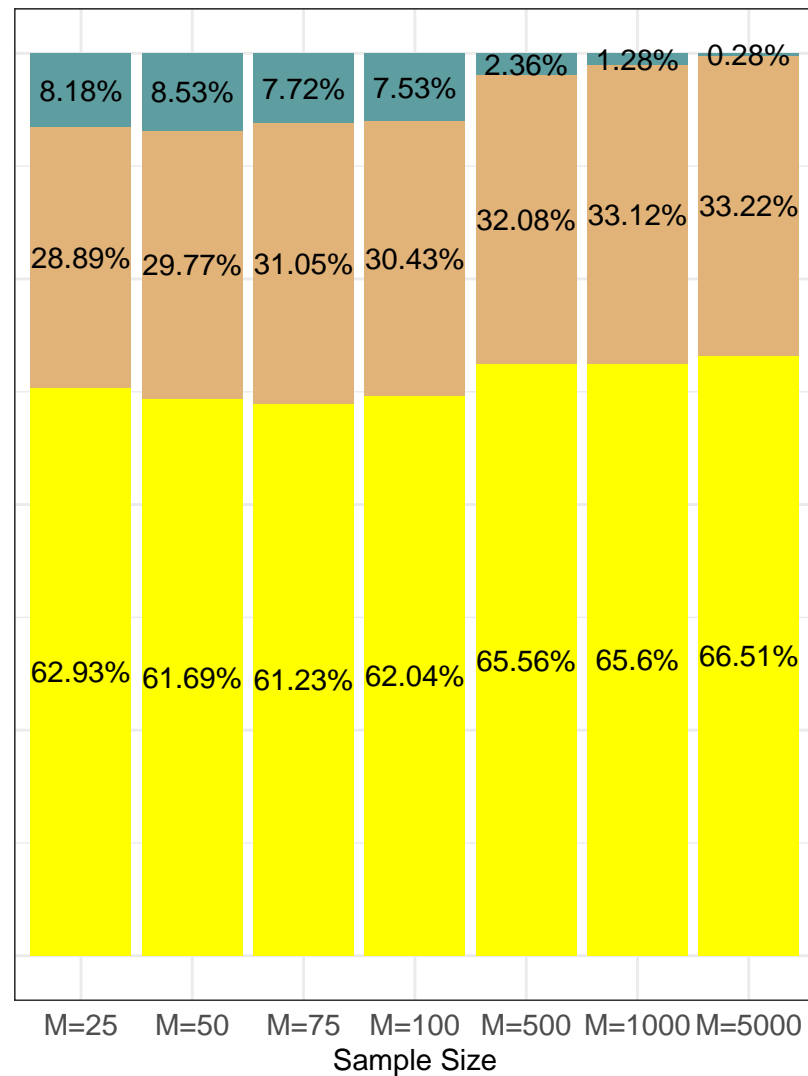

LSiSh

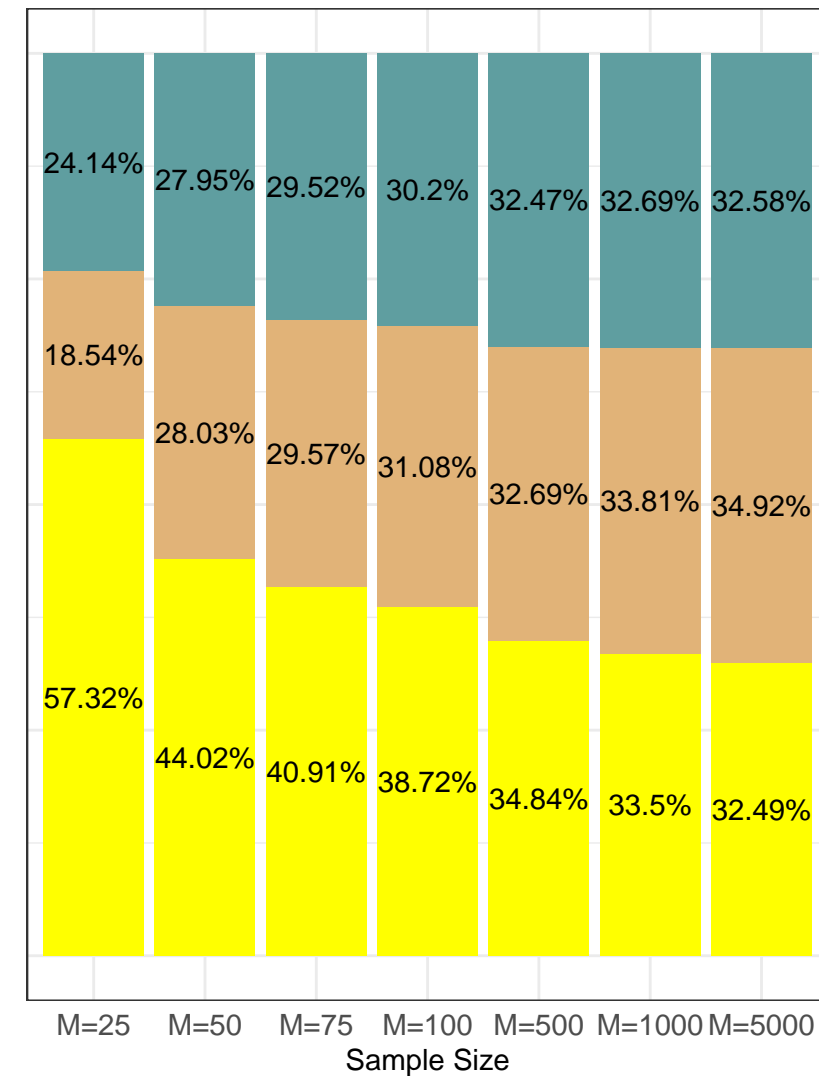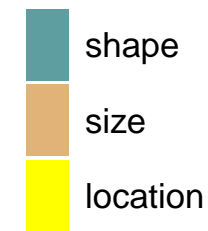

Supplement: btab226_Supplementary_Data [file btab226_supplementary_data.zip › Supplement_Revision2/FigA7.pdf]

**A**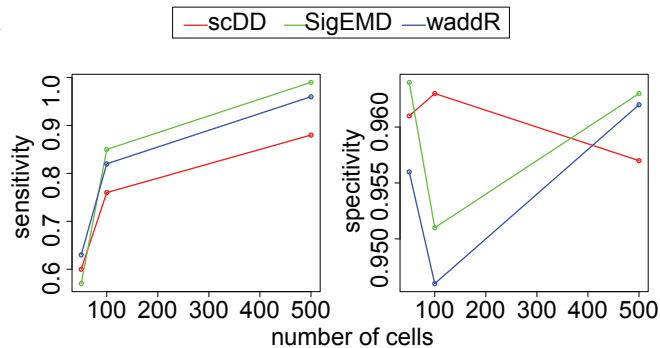**B**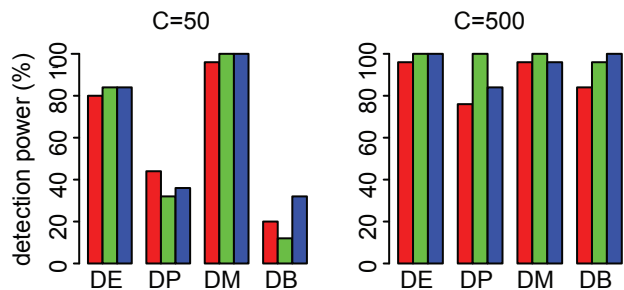**C**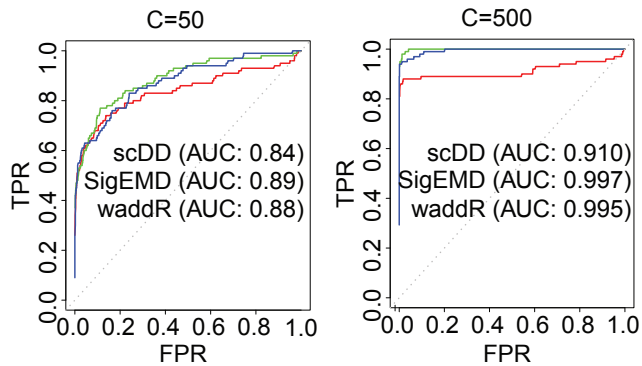**D**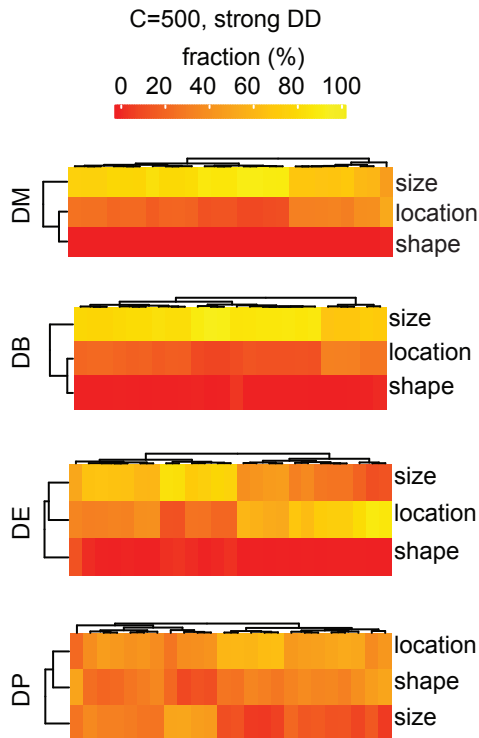**E**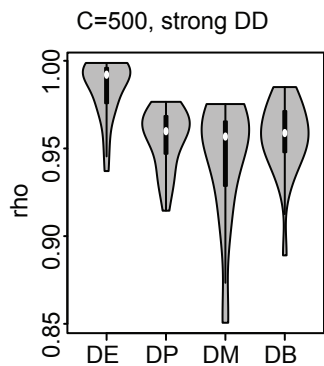

Supplement: btab226_Supplementary_Data [file btab226_supplementary_data.zip › Supplement_Revision2/Figure2.pdf]

**A**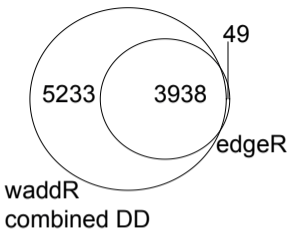**B**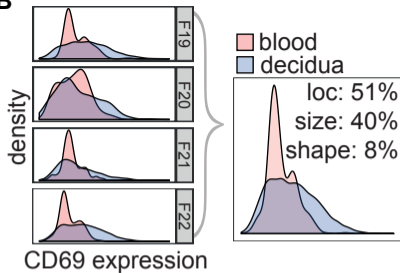**C**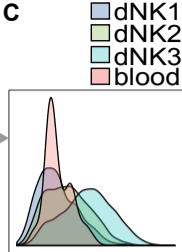**D**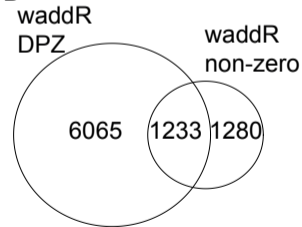**E**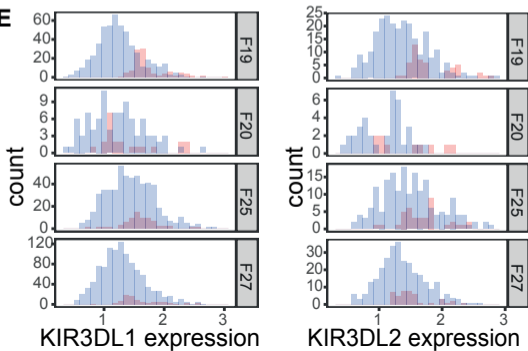

Supplement: btab226_Supplementary_Data [file btab226_supplementary_data.zip › Supplement_Revision2/Figure3.pdf]

**C=100, medium DD: Category DE**

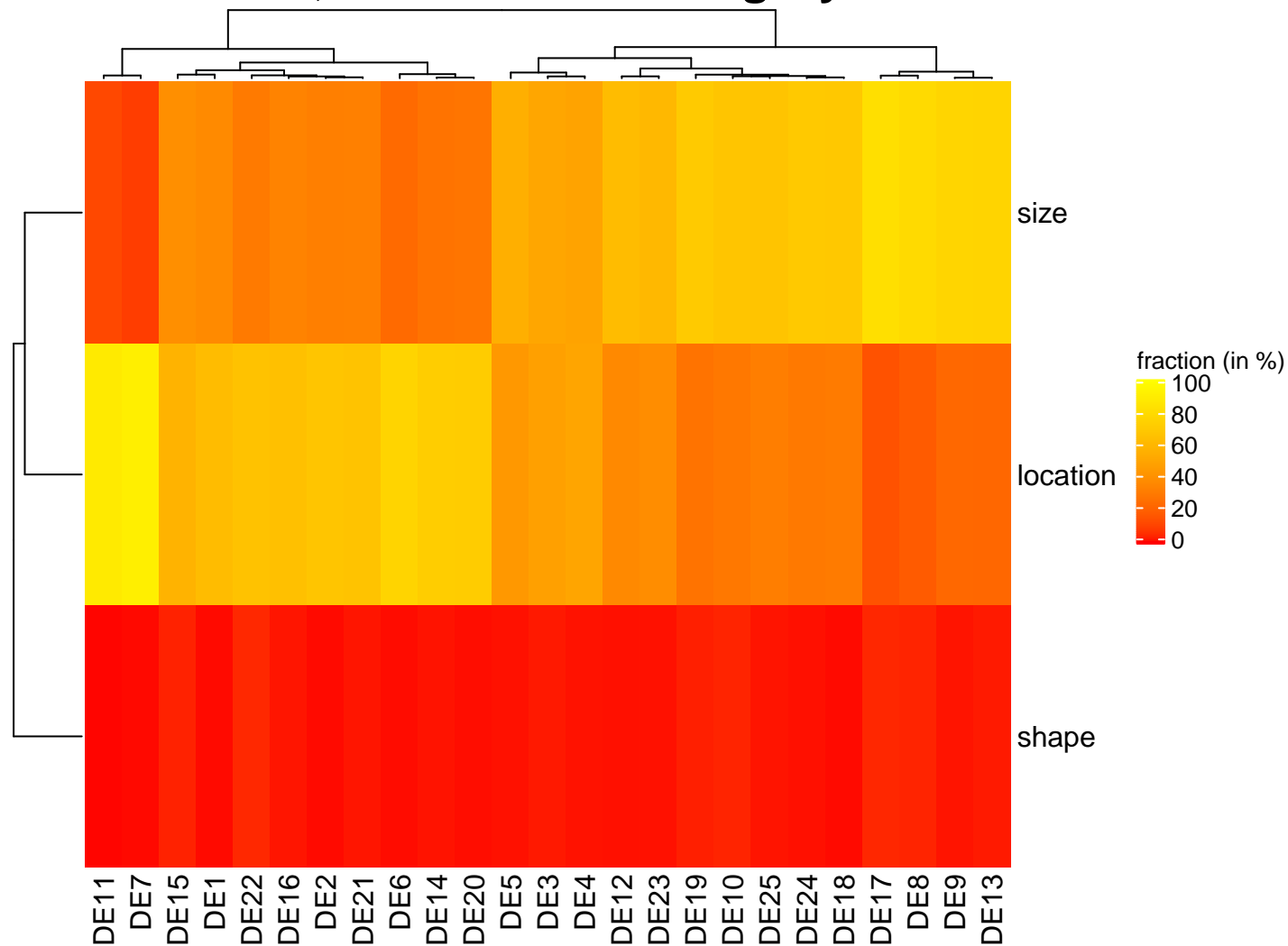

**C=100, medium DD: Category DP**

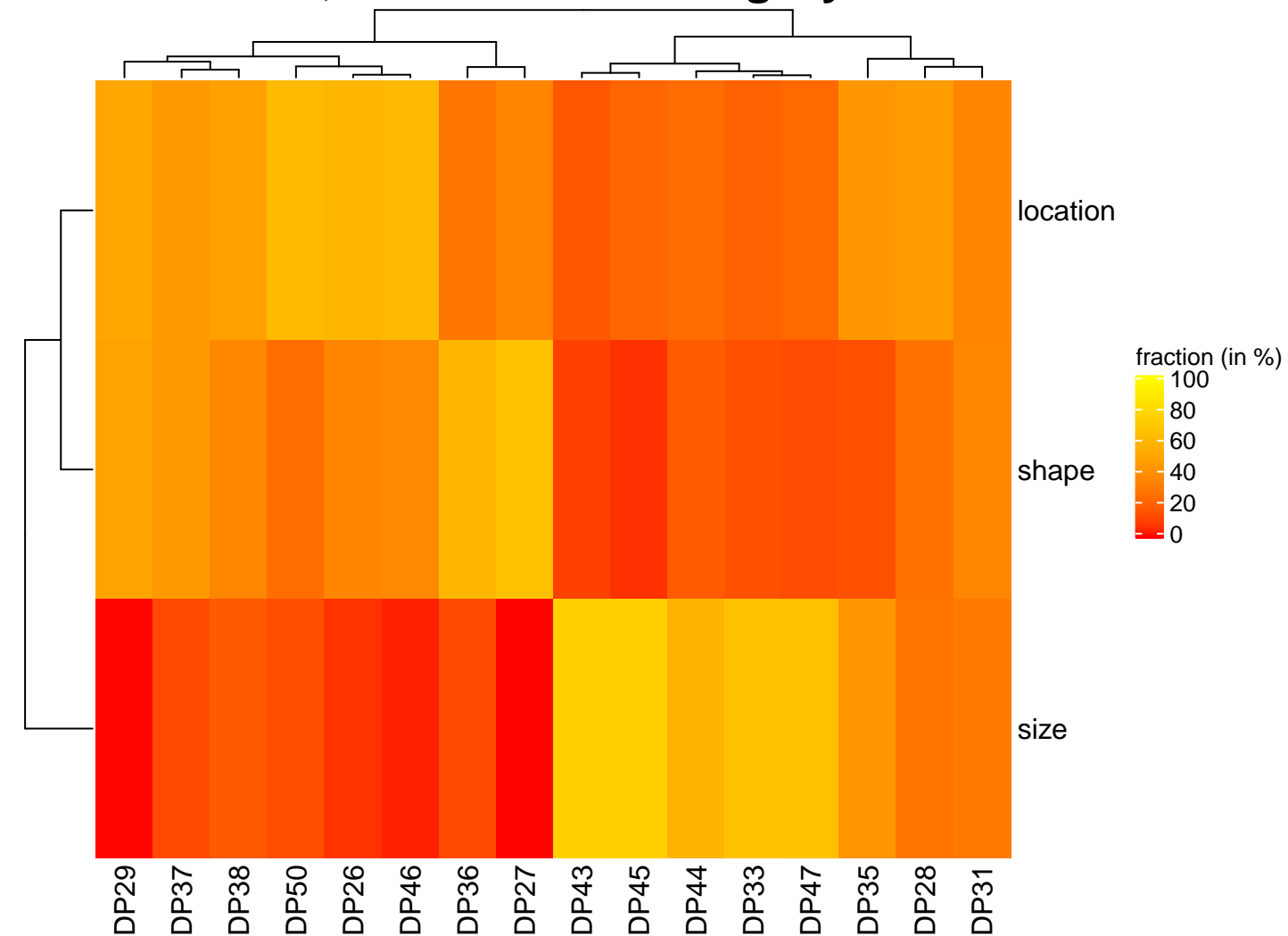

**C=100, medium DD: Category DM**

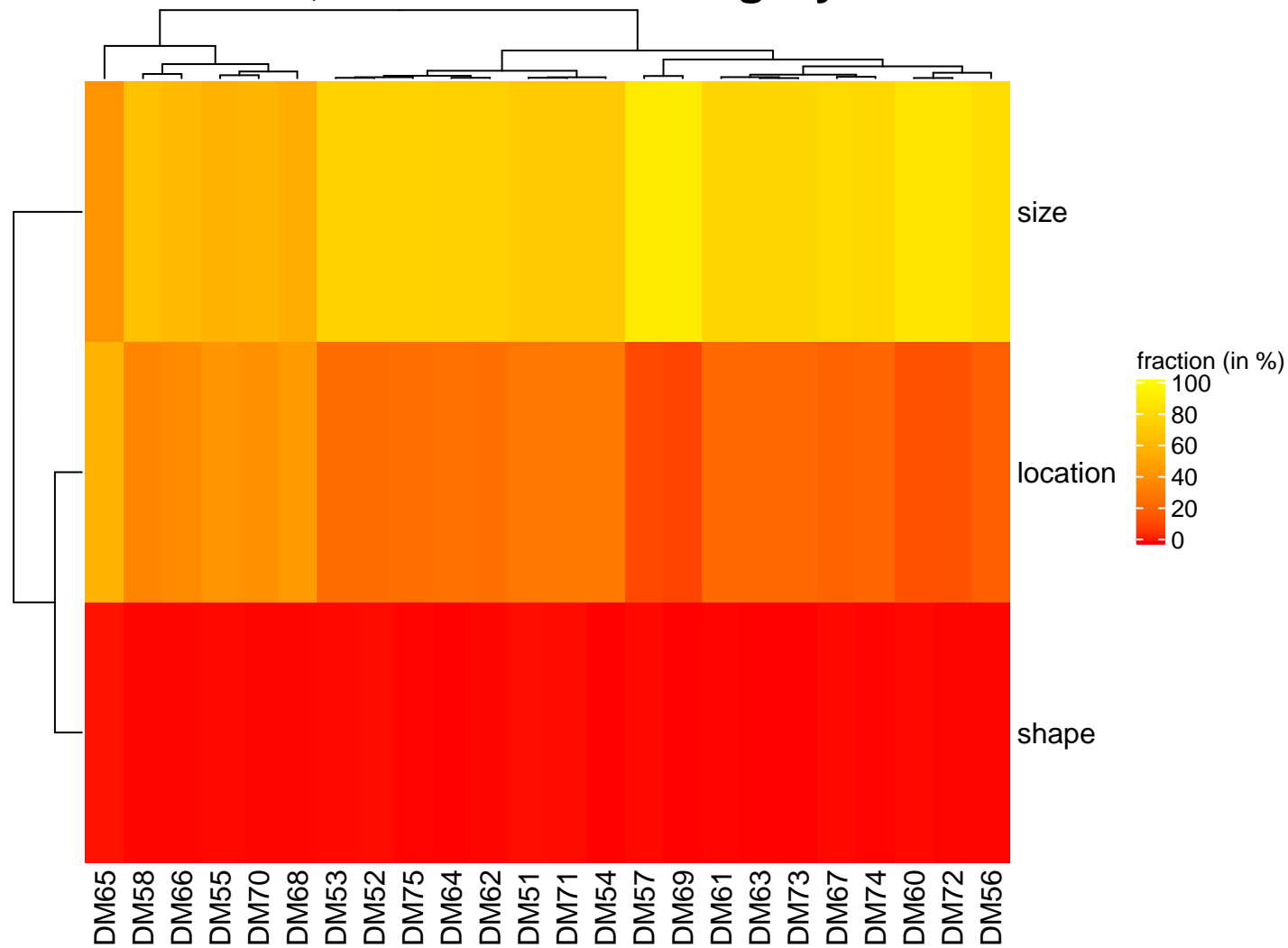

**C=100, medium DD: Category DB**

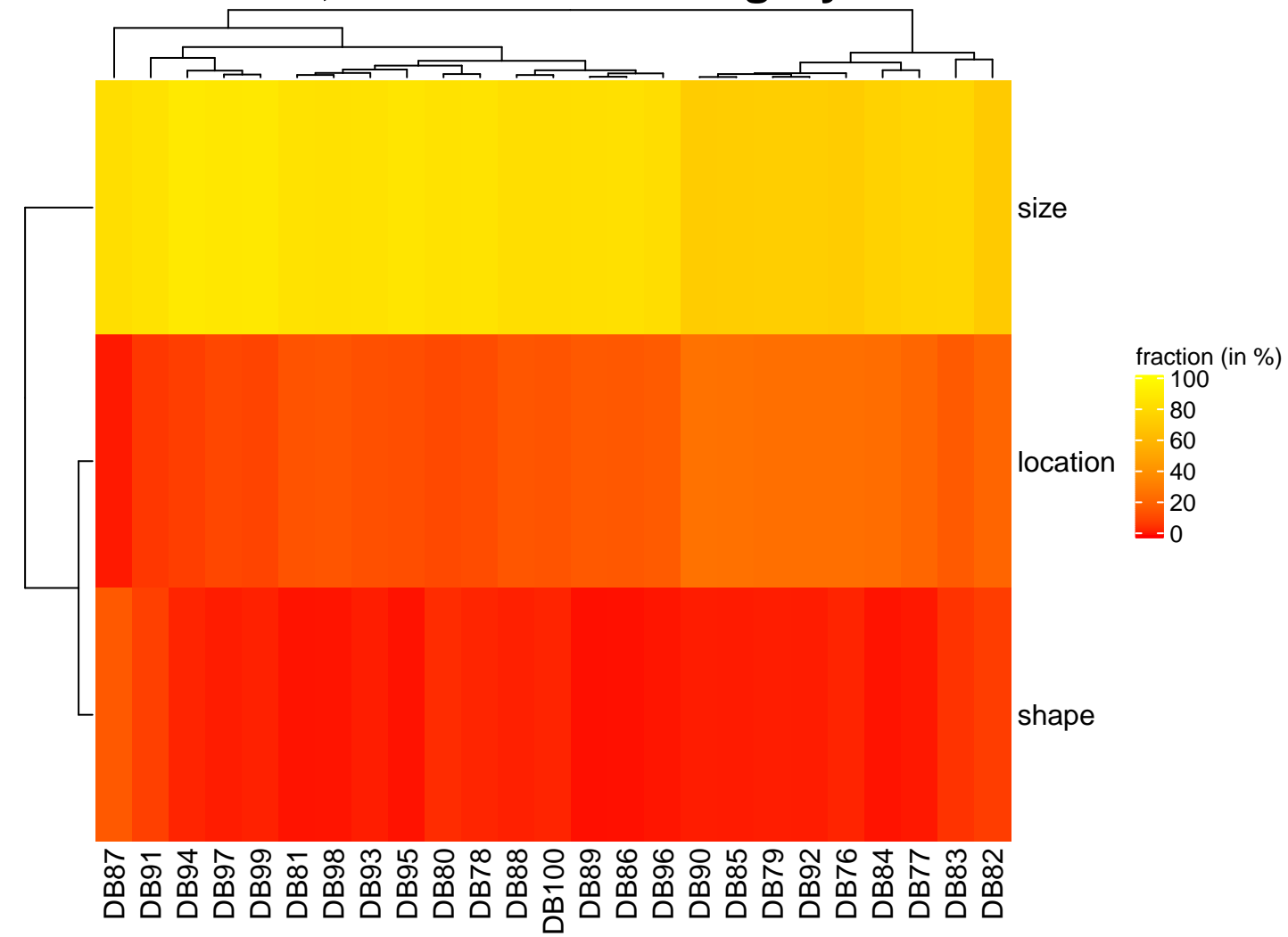

Supplement: btab226_Supplementary_Data [file btab226_supplementary_data.zip › Supplement_Revision2/Heatmaps_cells100_mediumDD.pdf]

**C=100, strong DD: Category DE**

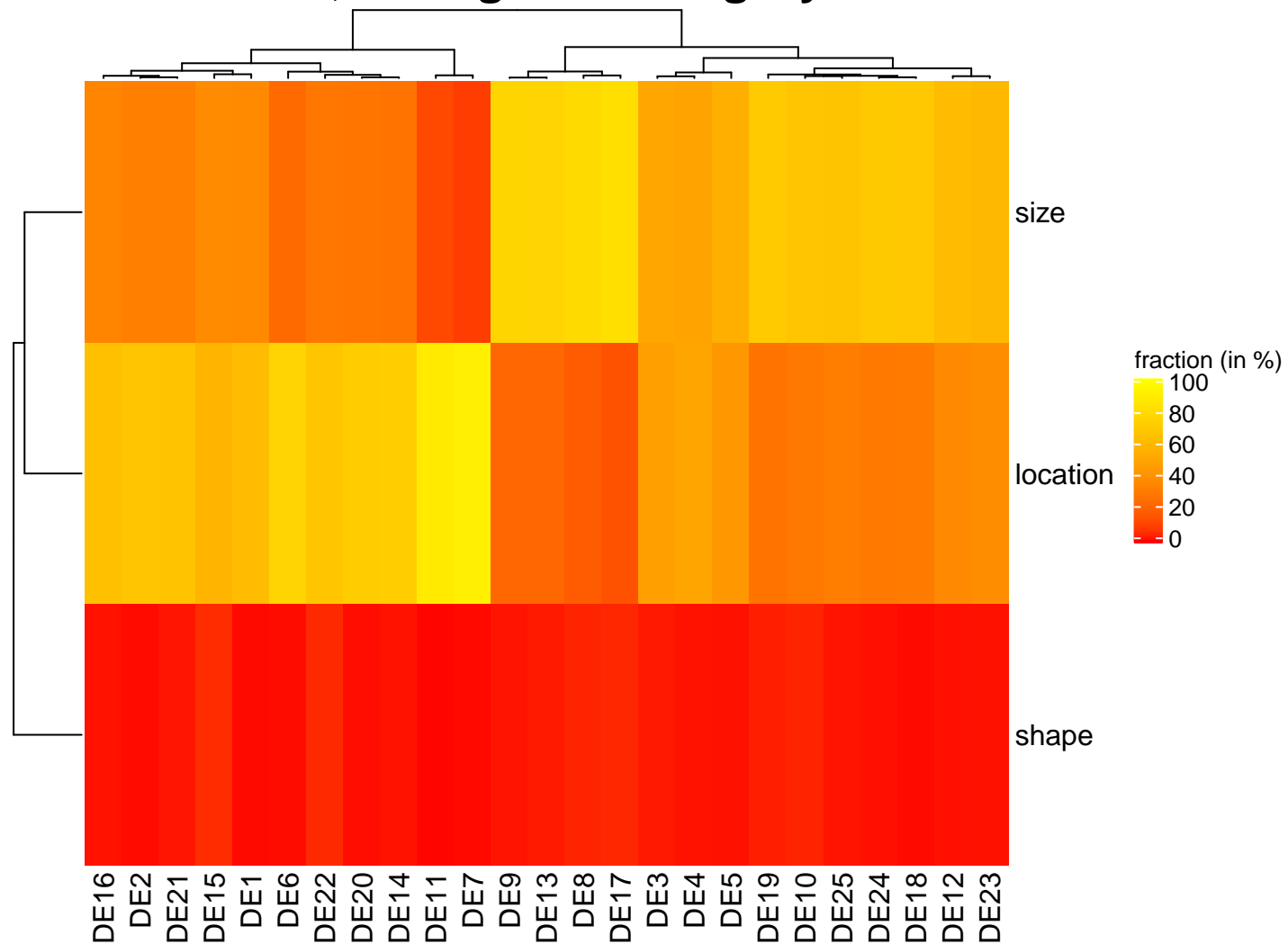

**C=100, strong DD: Category DP**

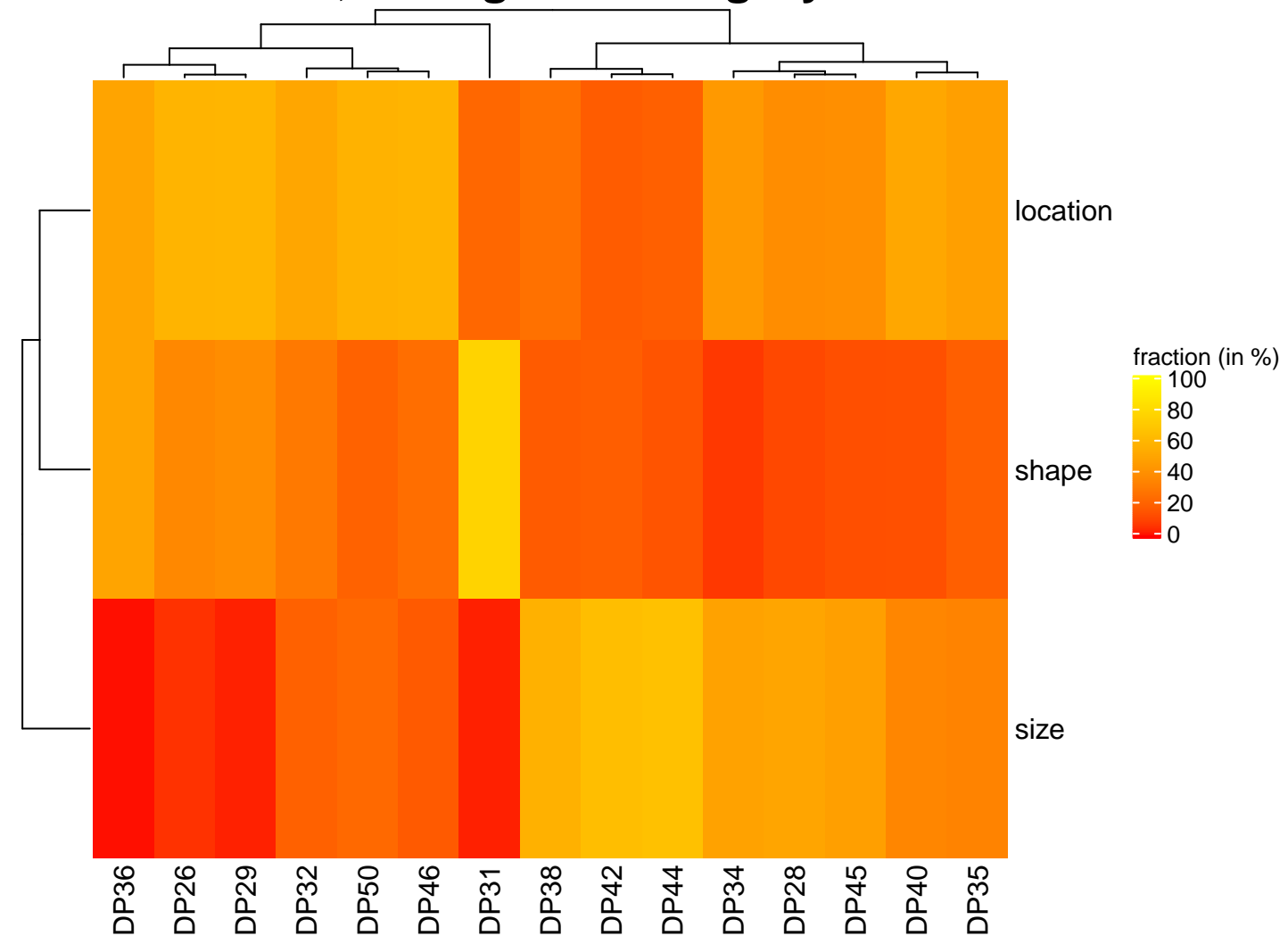

**C=100, strong DD: Category DM**

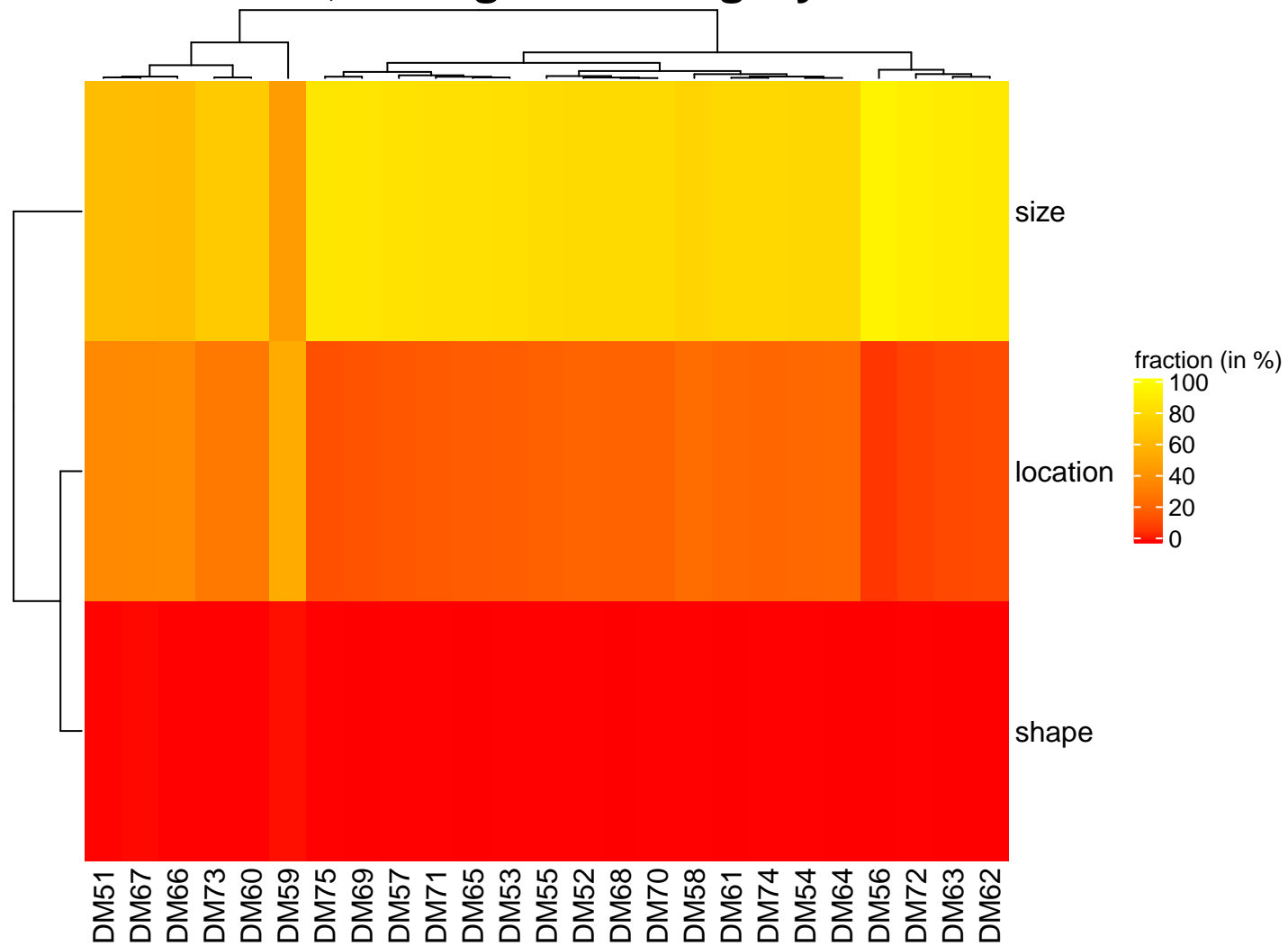

**C=100, strong DD: Category DB**

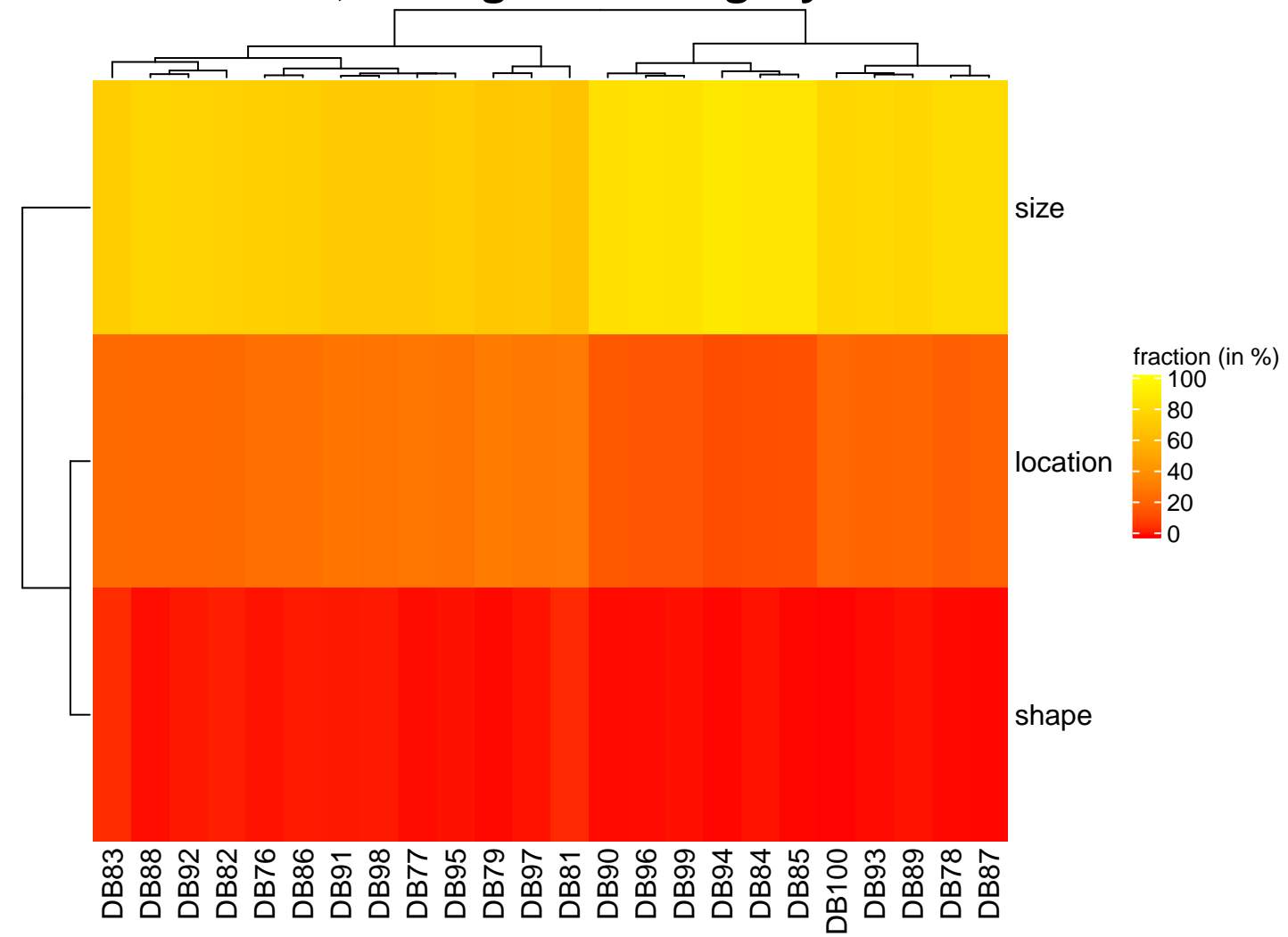

Supplement: btab226_Supplementary_Data [file btab226_supplementary_data.zip › Supplement_Revision2/Heatmaps_cells100_strongDD.pdf]

**C=100, weak DD: Category DE**

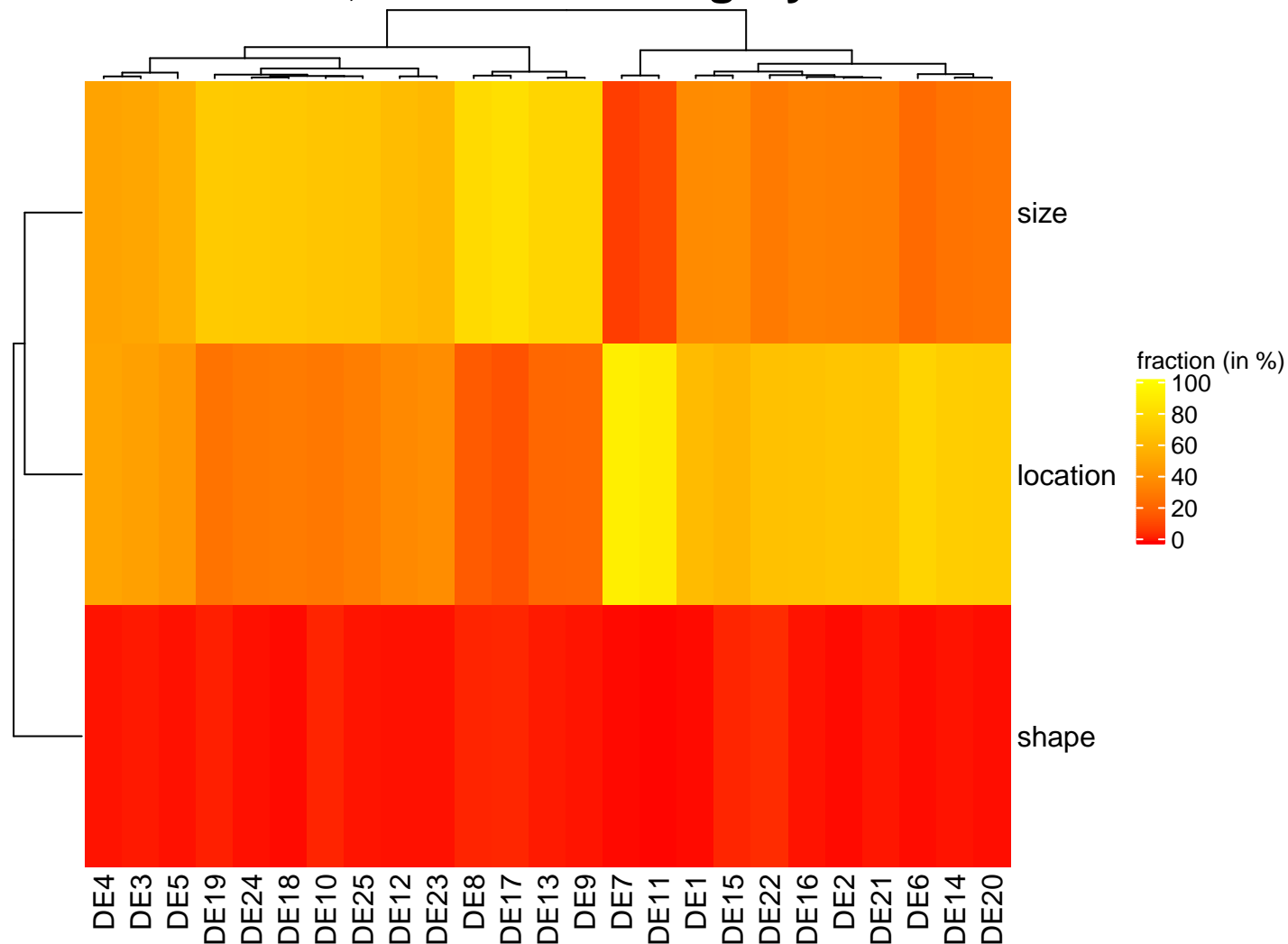

**C=100, weak DD: Category DP**

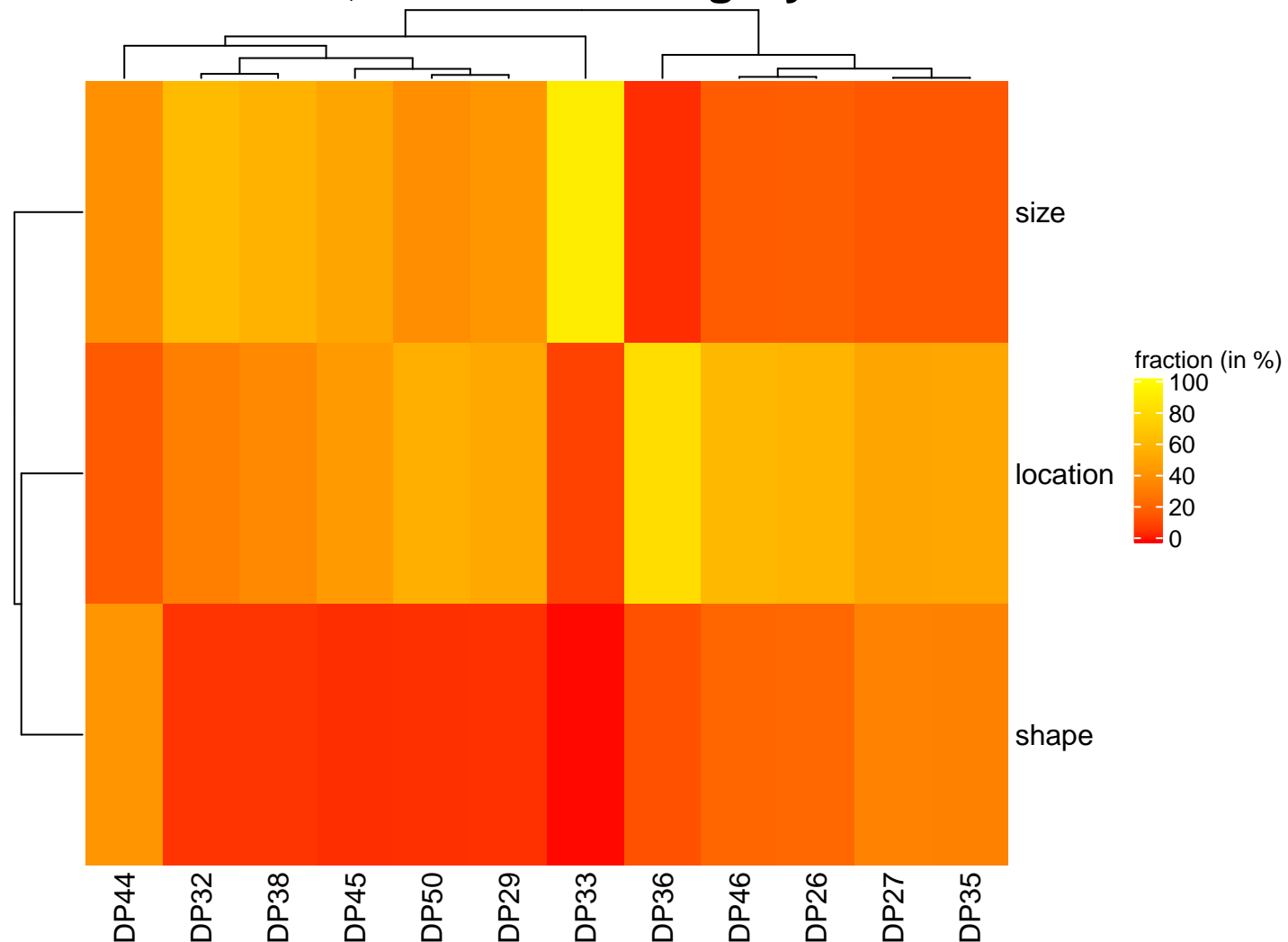

**C=100, weak DD: Category DM**

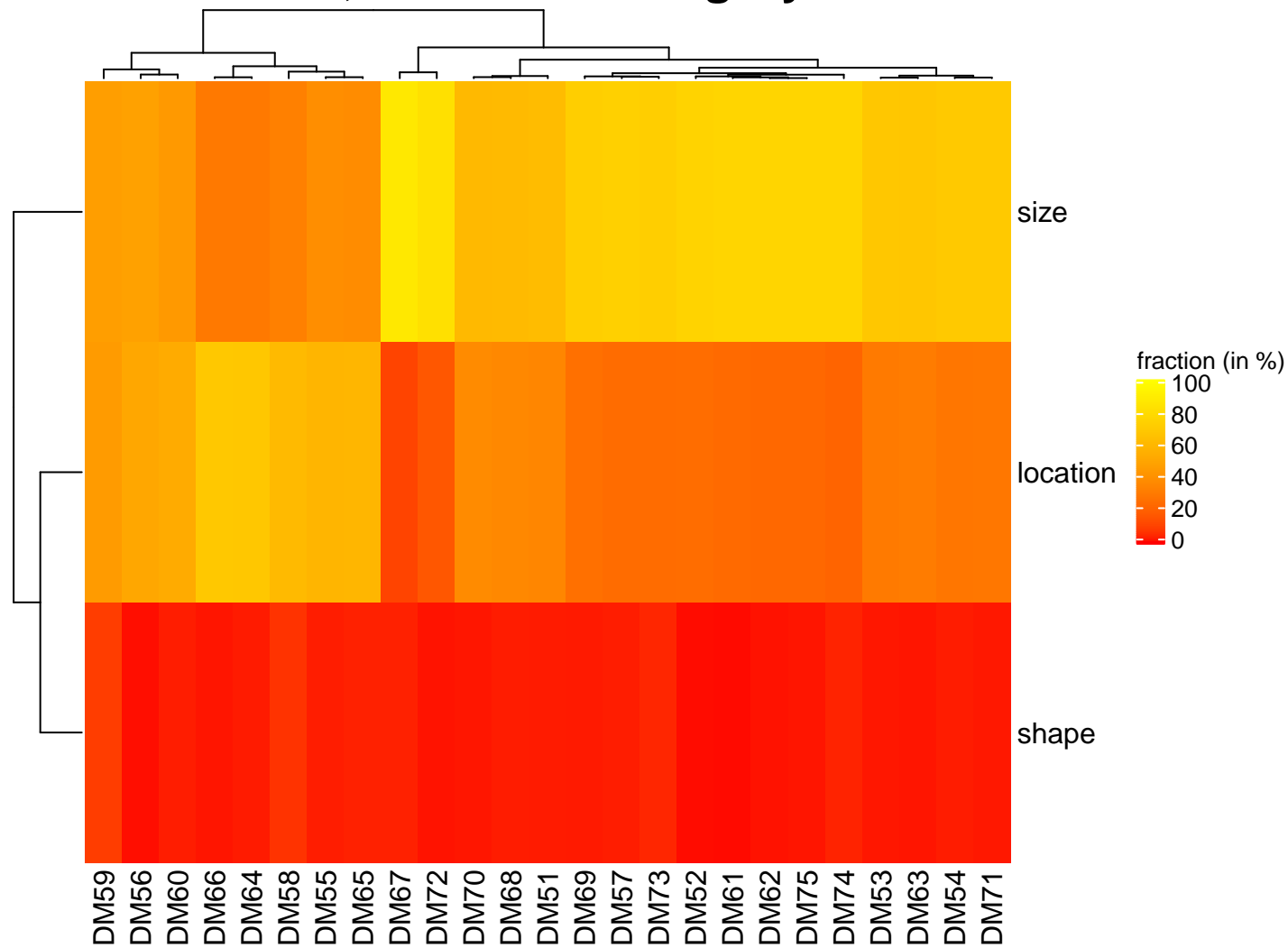

**C=100, weak DD: Category DB**

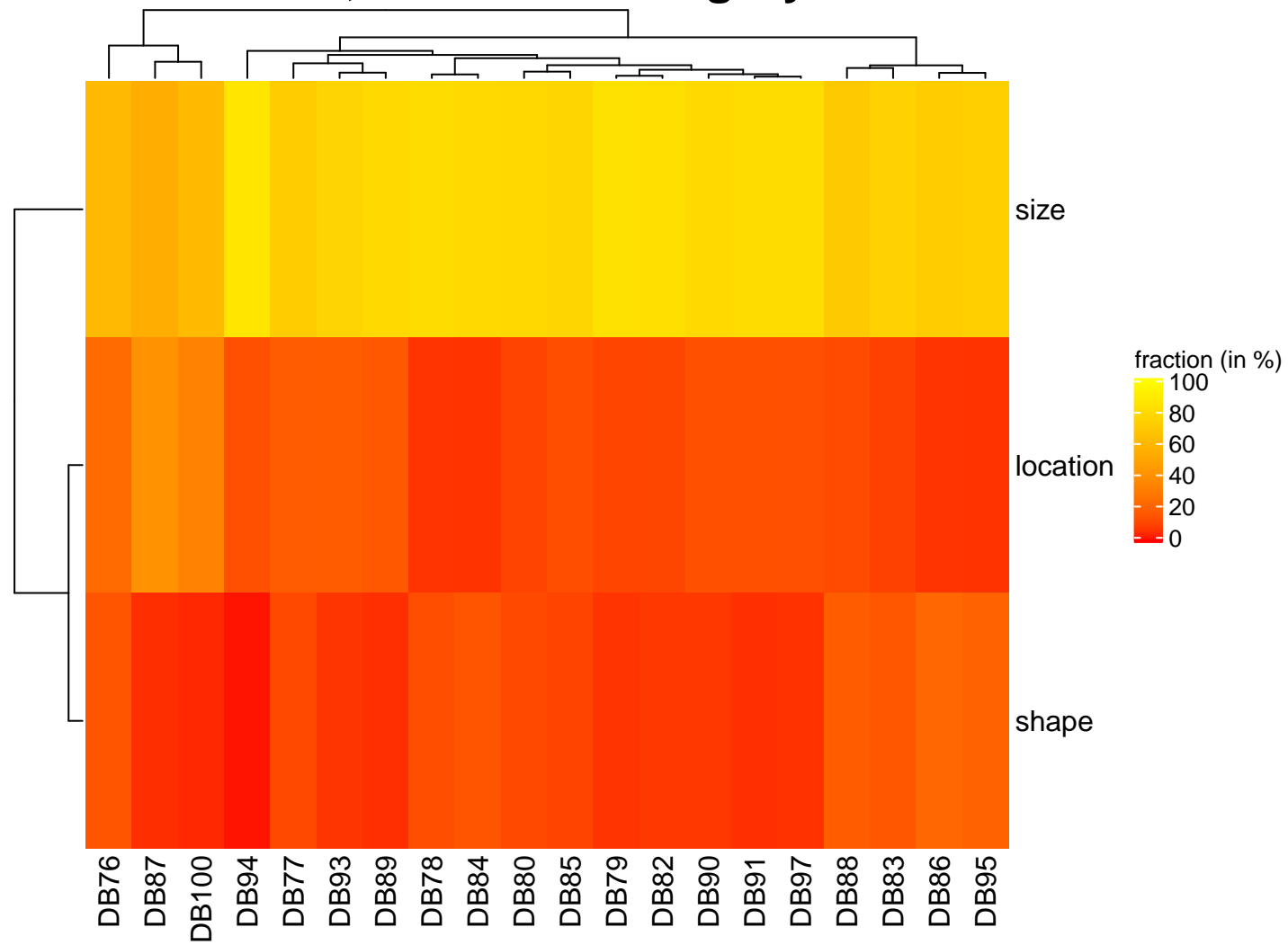

Supplement: btab226_Supplementary_Data [file btab226_supplementary_data.zip › Supplement_Revision2/Heatmaps_cells100_weakDD.pdf]

**C=500, medium DD: Category DE**

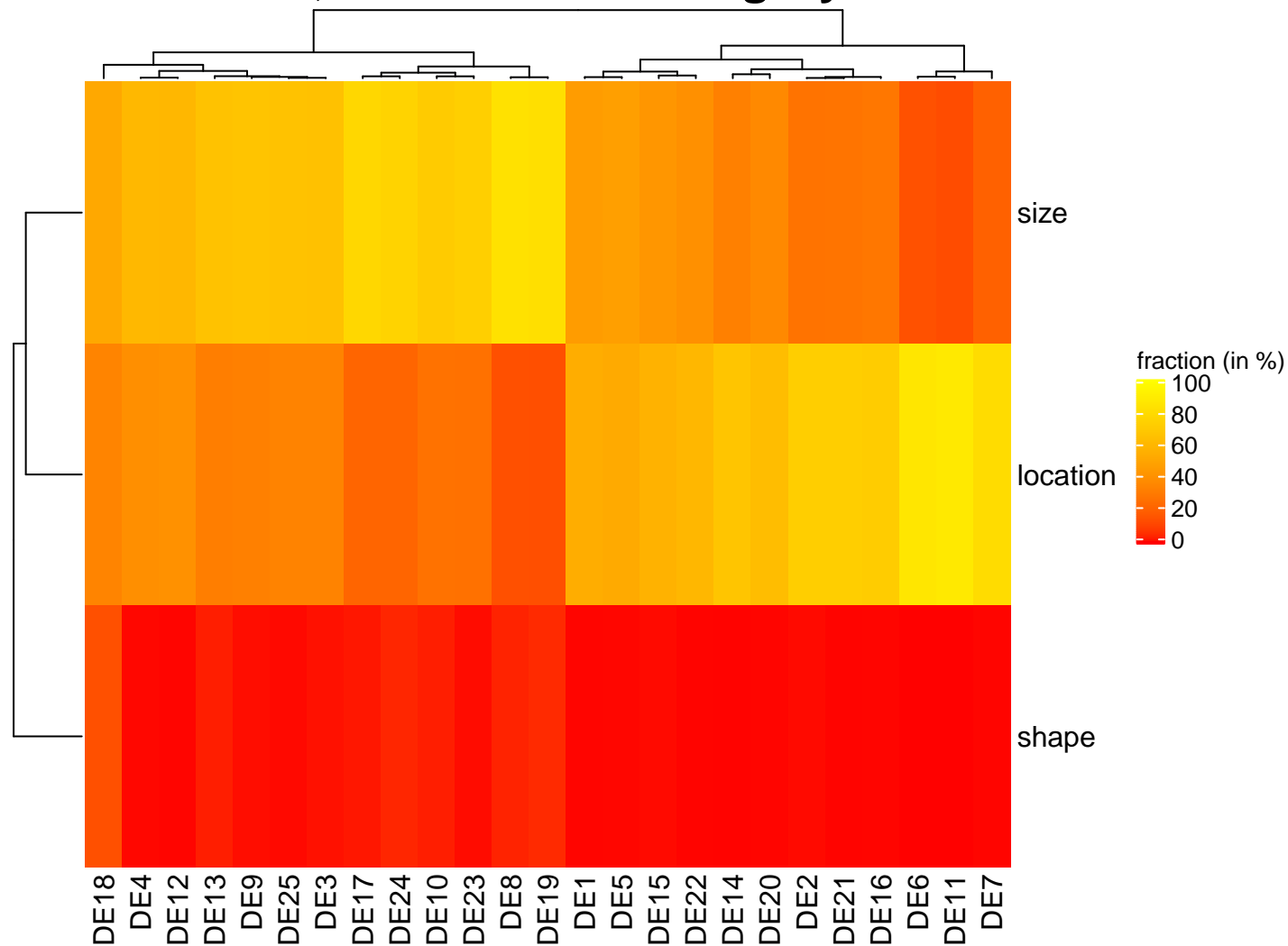

**C=500, medium DD: Category DP**

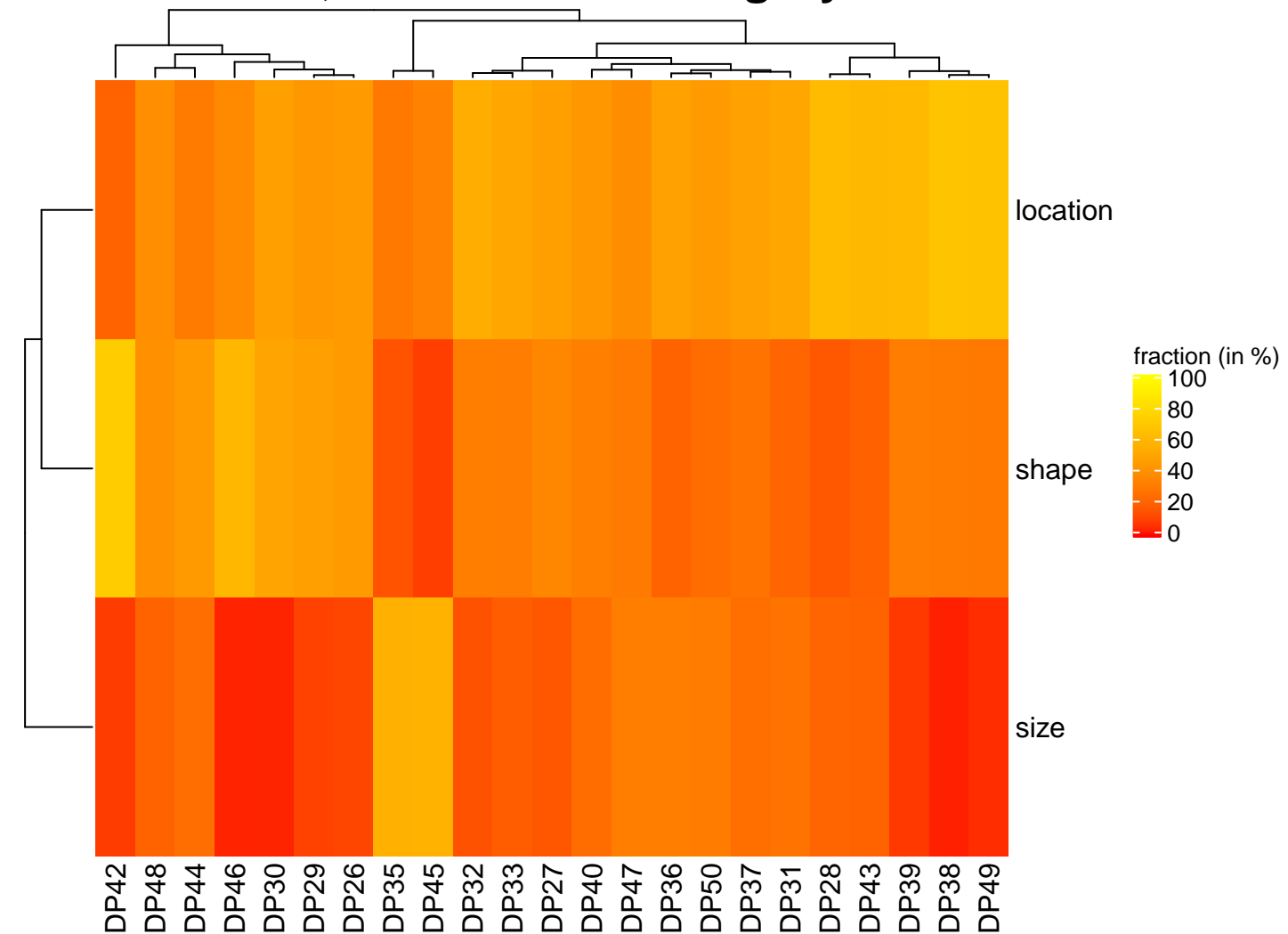

**C=500, medium DD: Category DM**

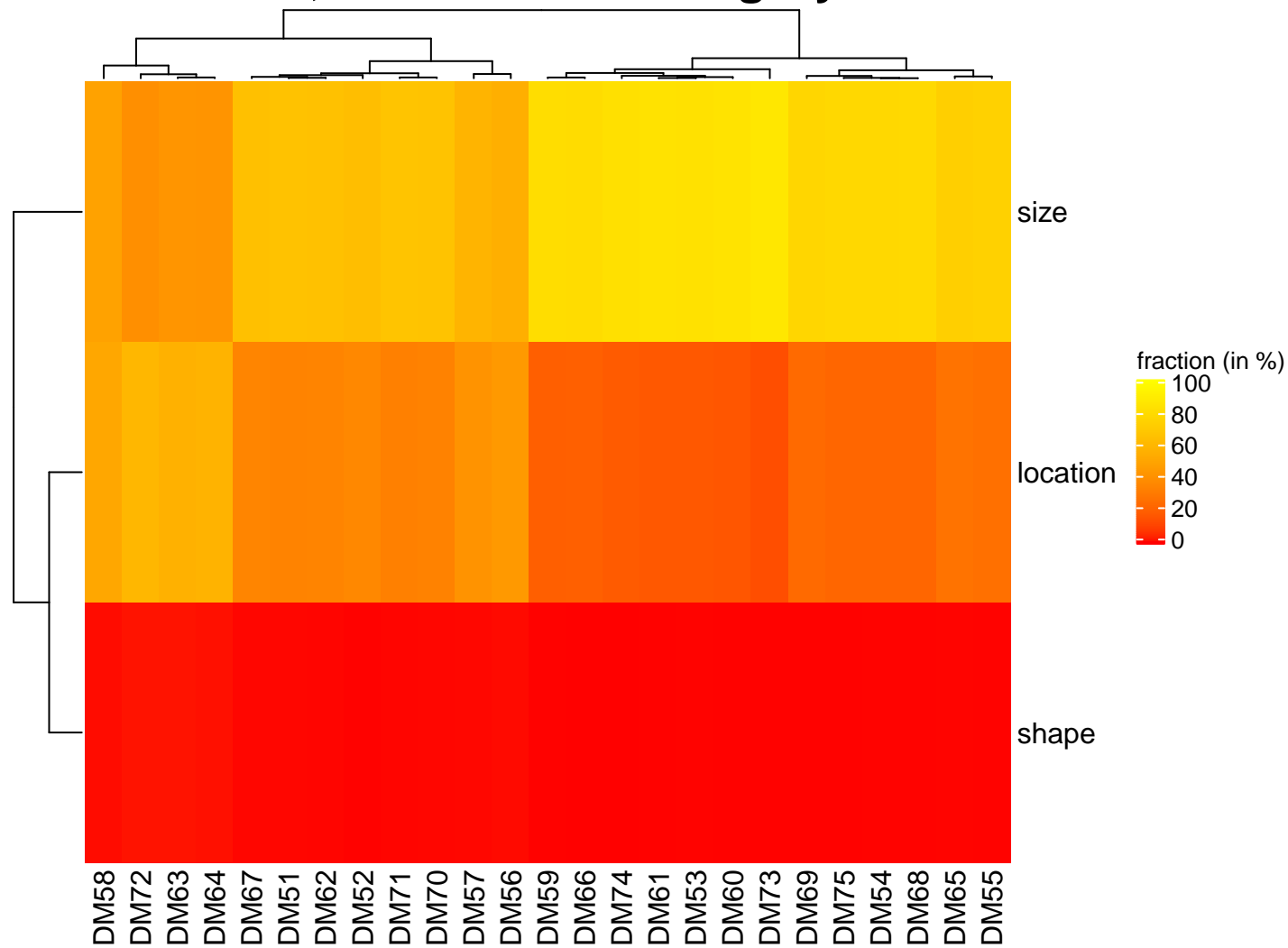

**C=500, medium DD: Category DB**

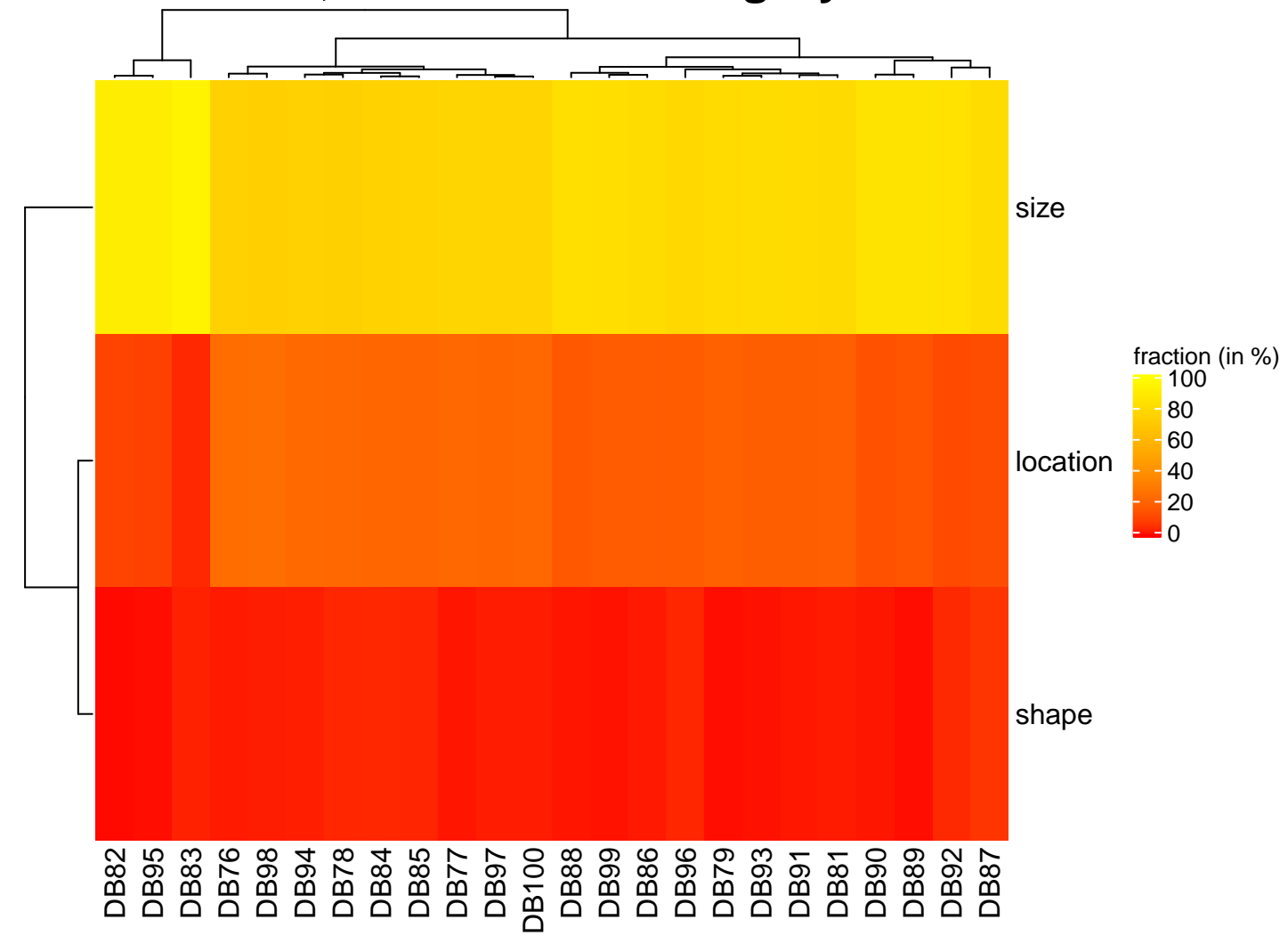

Supplement: btab226_Supplementary_Data [file btab226_supplementary_data.zip › Supplement_Revision2/Heatmaps_cells500_mediumDD.pdf]

**C=500, weak DD: Category DE**

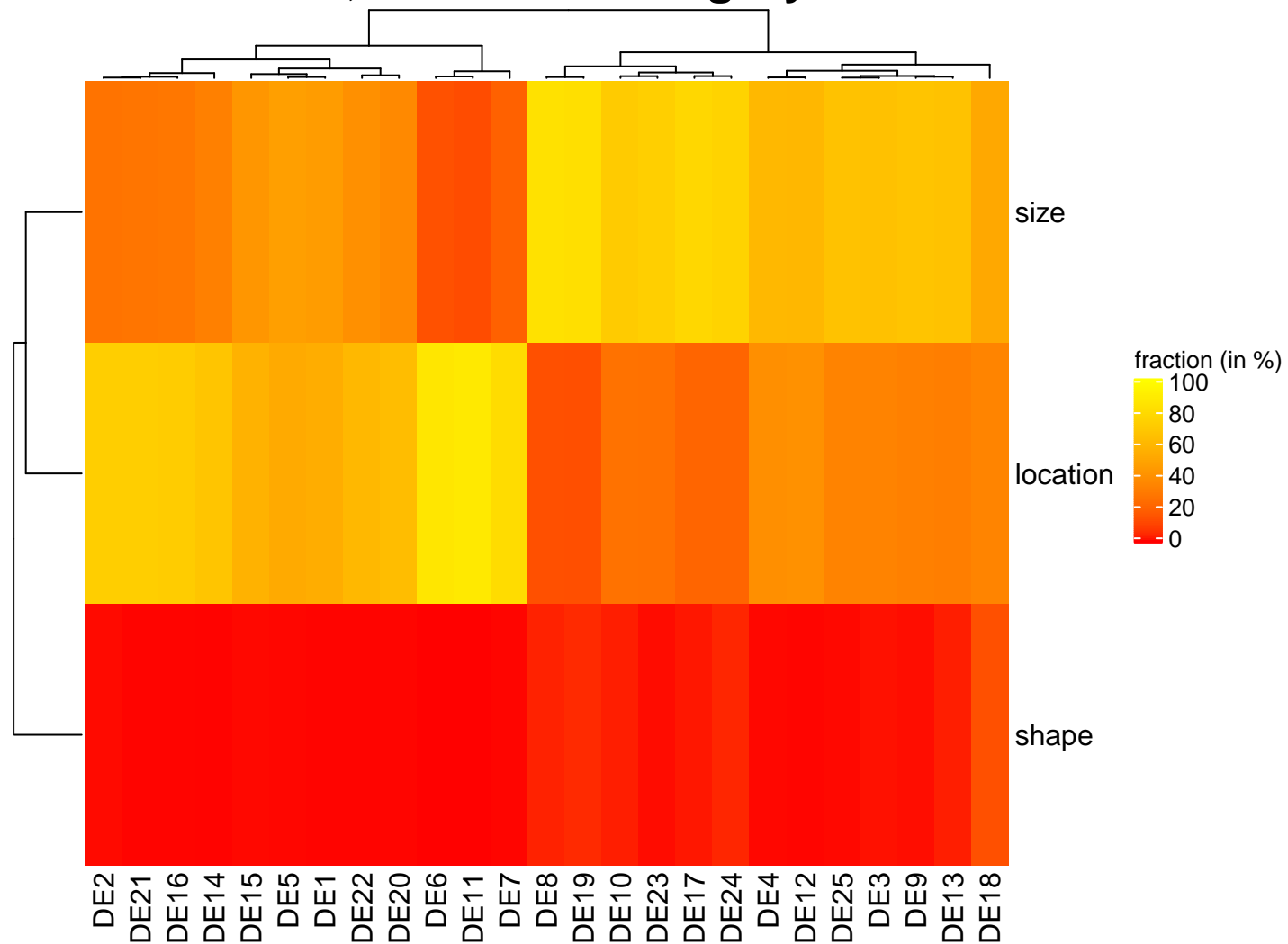

**C=500, weak DD: Category DP**

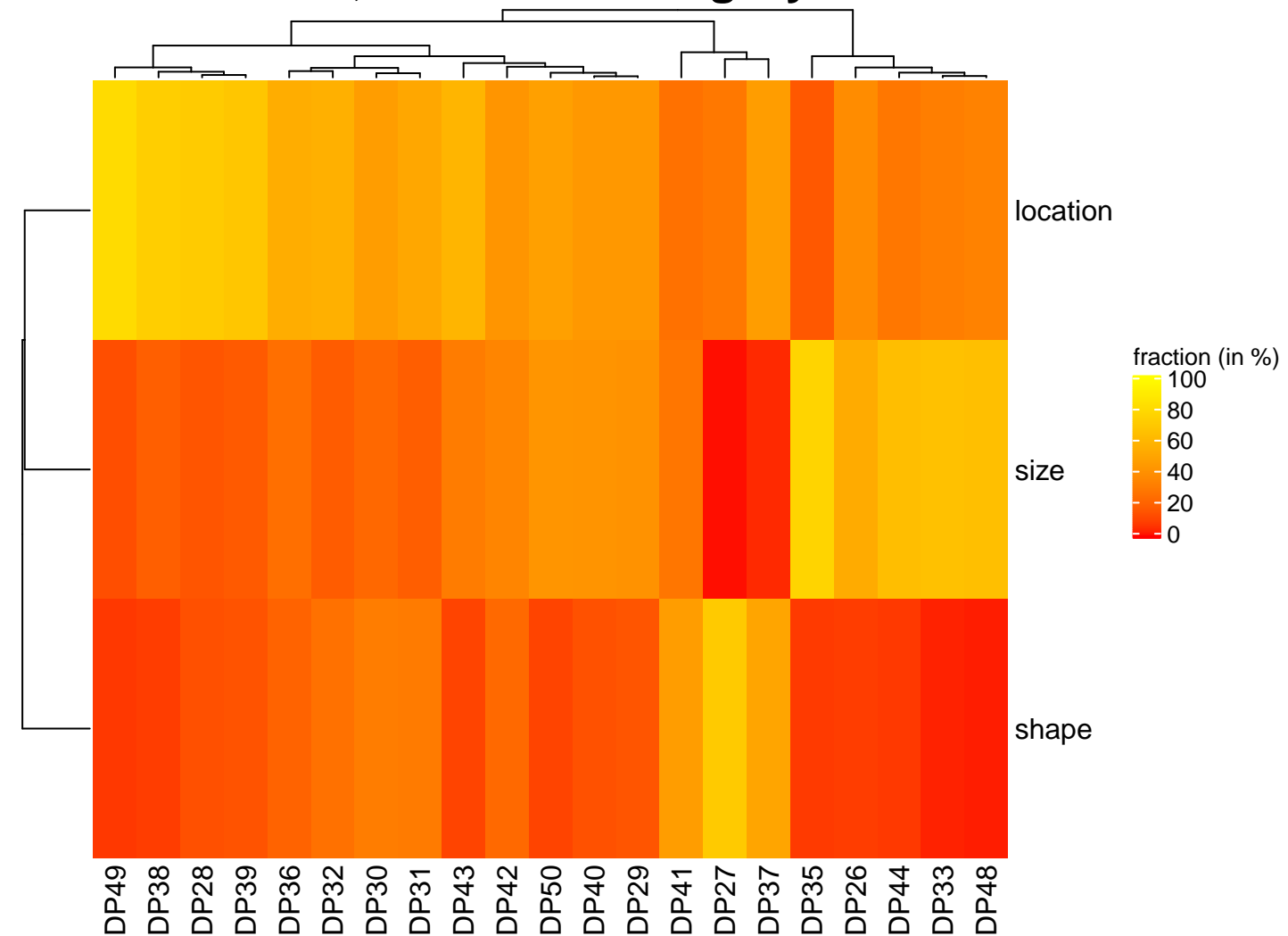

**C=500, weak DD: Category DM**

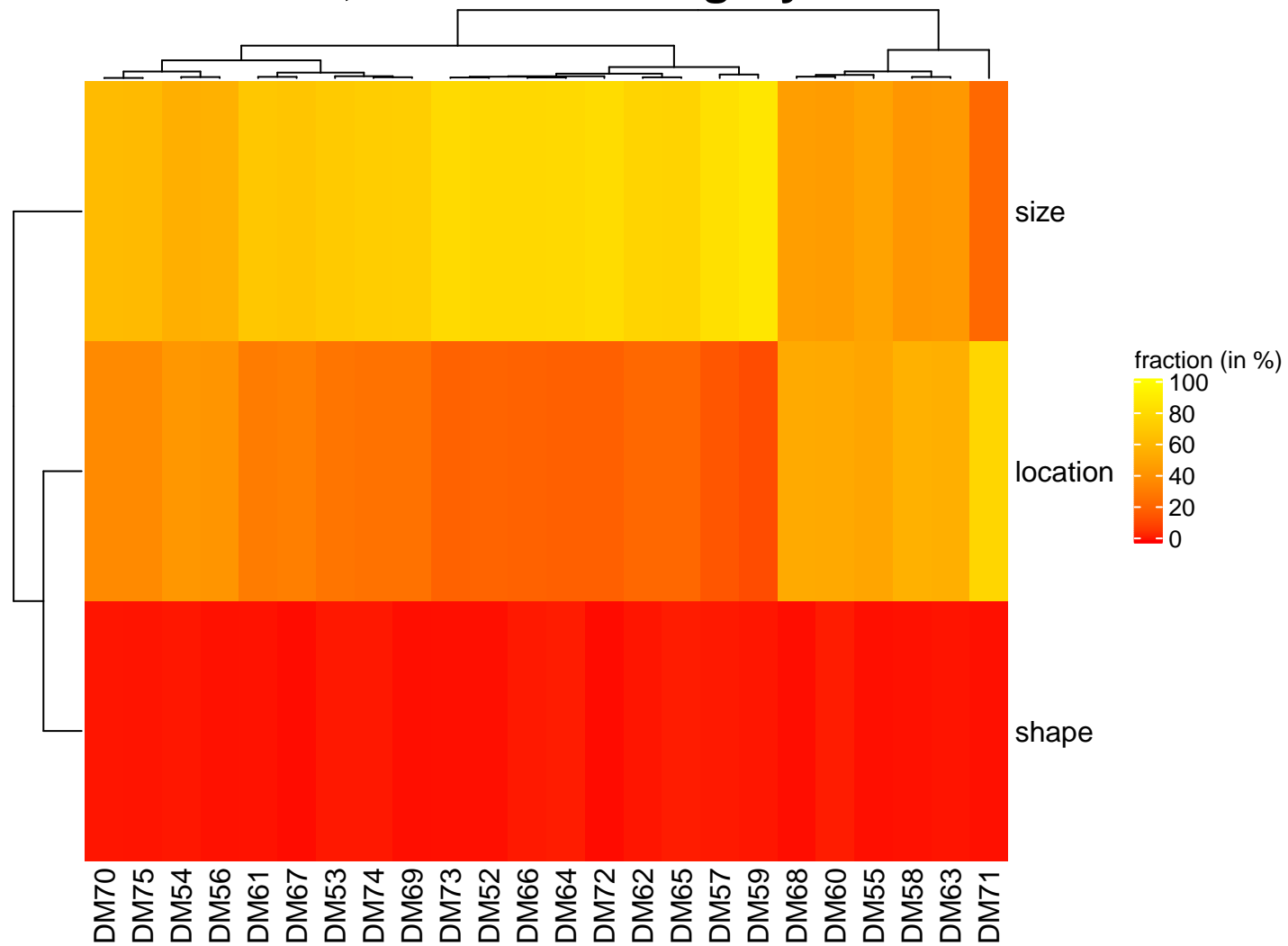

**C=500, weak DD: Category DB**

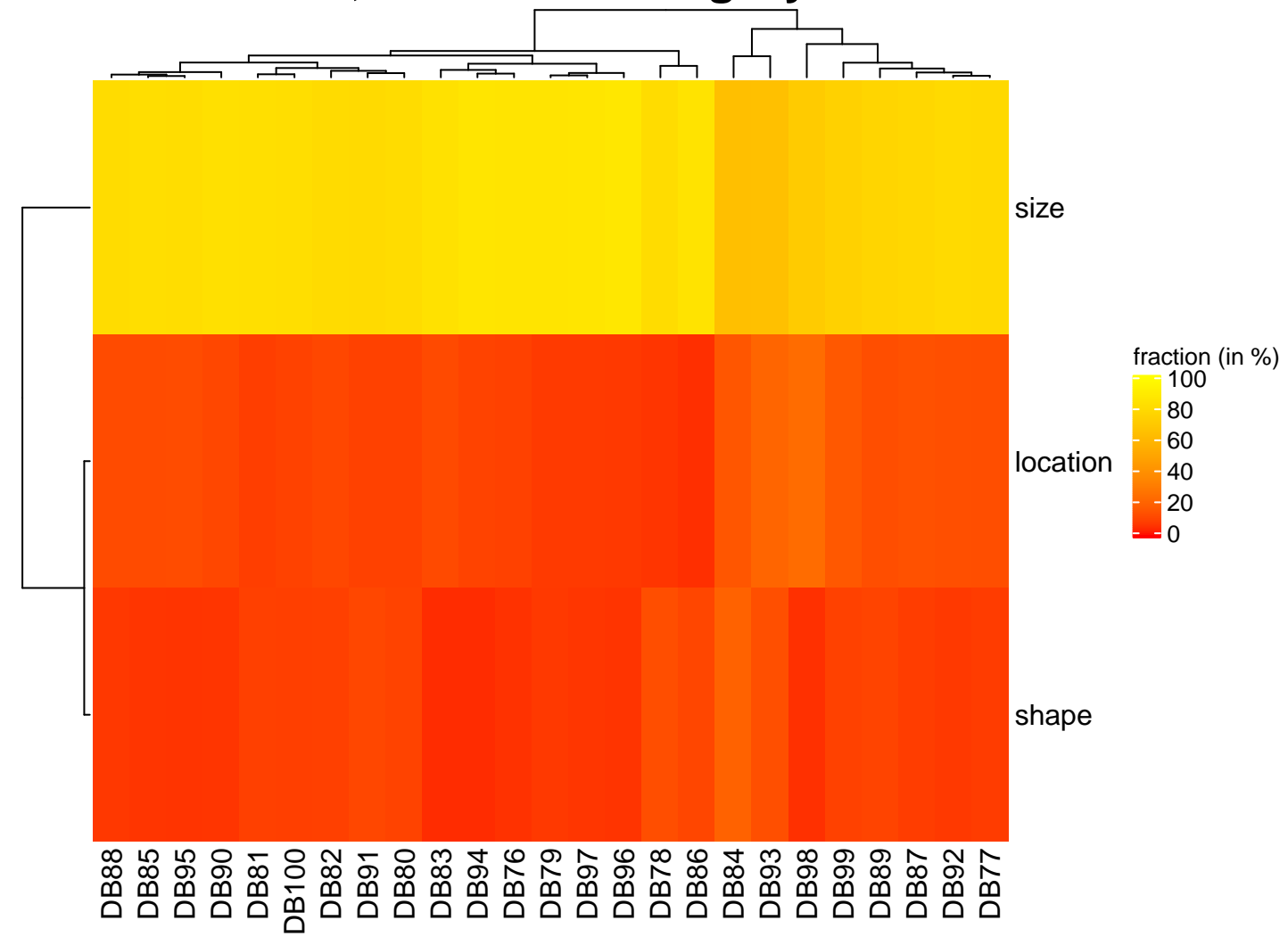

Supplement: btab226_Supplementary_Data [file btab226_supplementary_data.zip › Supplement_Revision2/Heatmaps_cells500_weakDD.pdf]

**C=50, medium DD: Category DE**

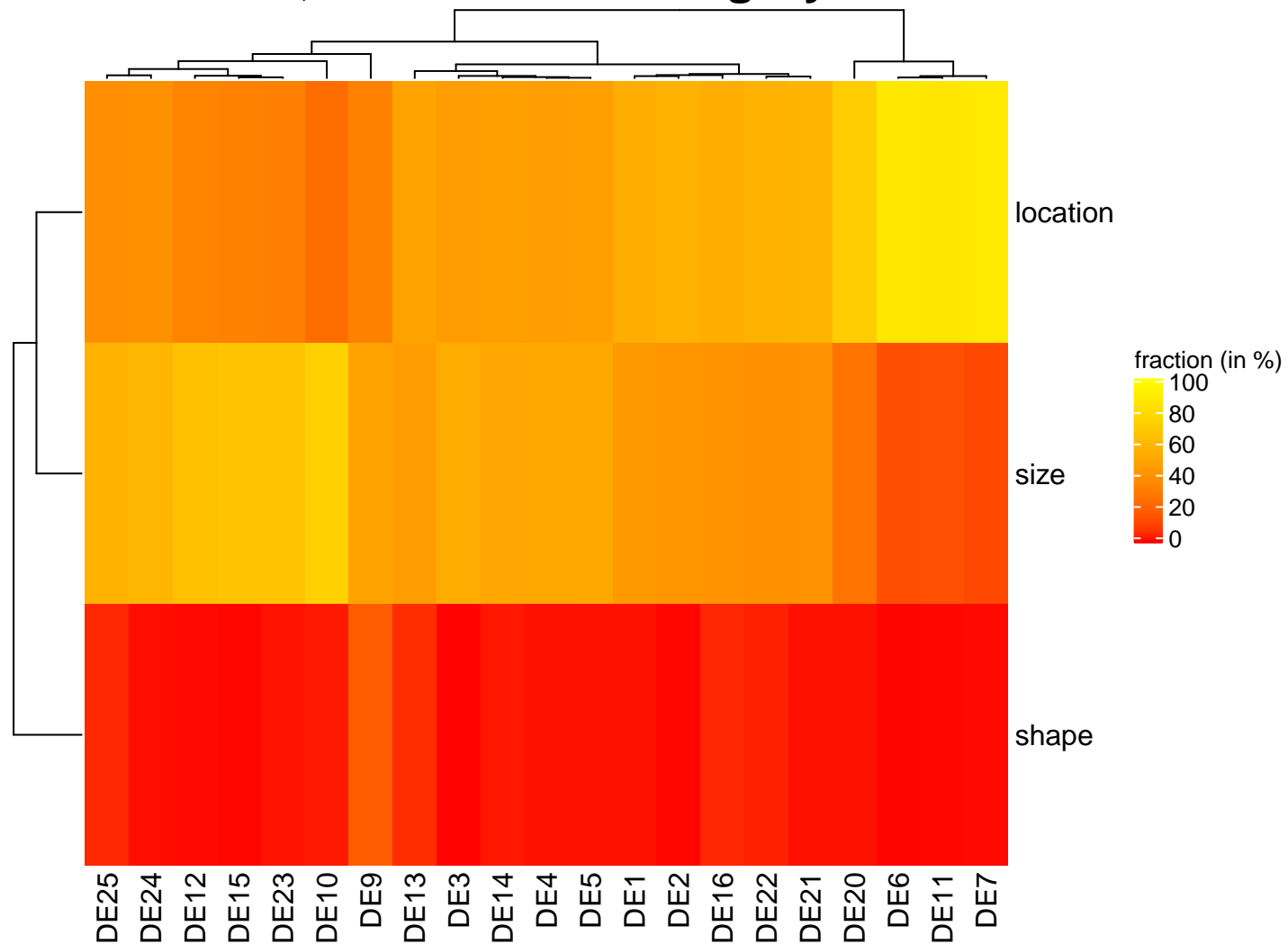

**C=50, medium DD: Category DP**

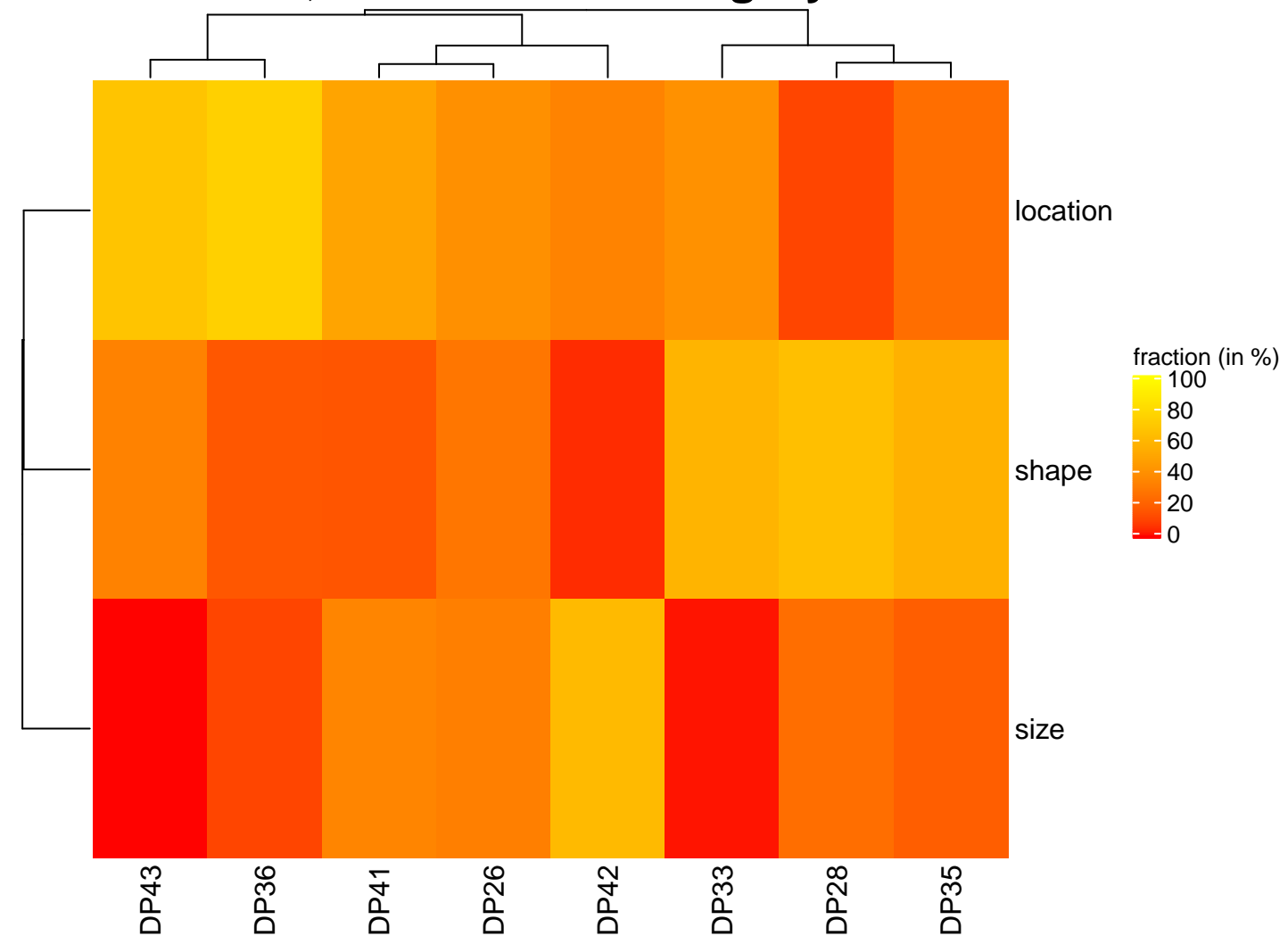

**C=50, medium DD: Category DM**

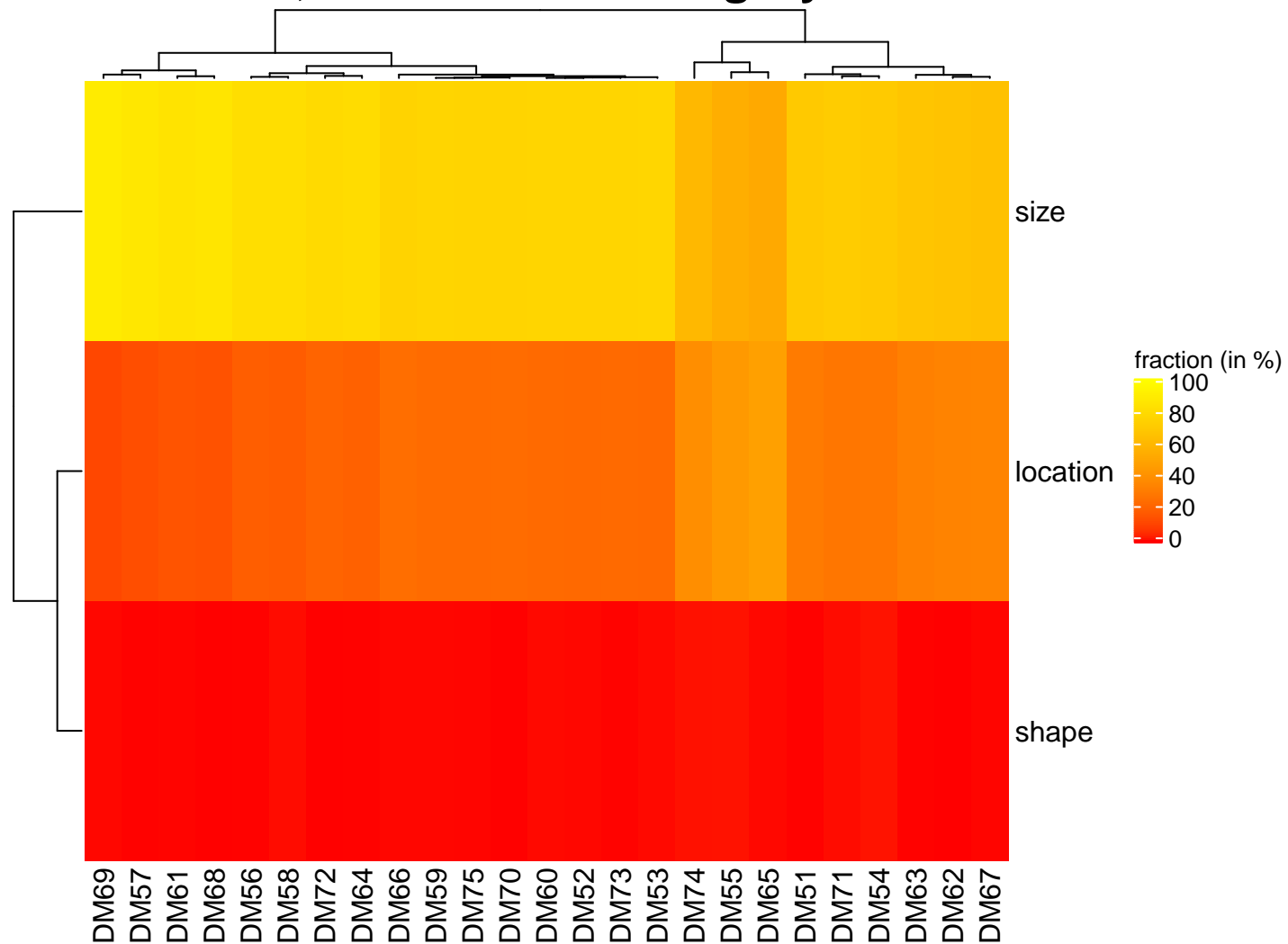

**C=50, medium DD: Category DB**

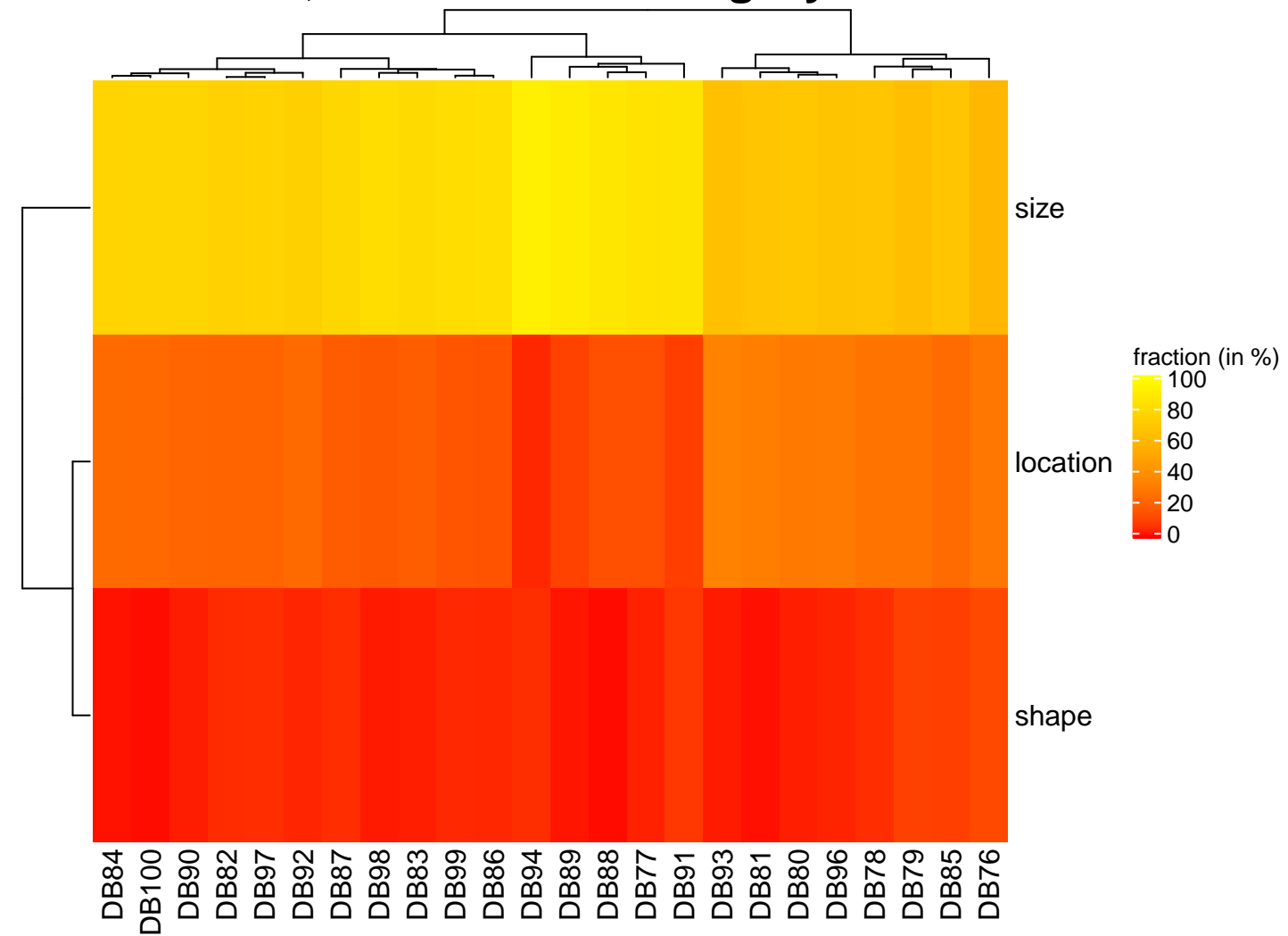

Supplement: btab226_Supplementary_Data [file btab226_supplementary_data.zip › Supplement_Revision2/Heatmaps_cells50_mediumDD.pdf]

**C=50, strong DD: Category DE**

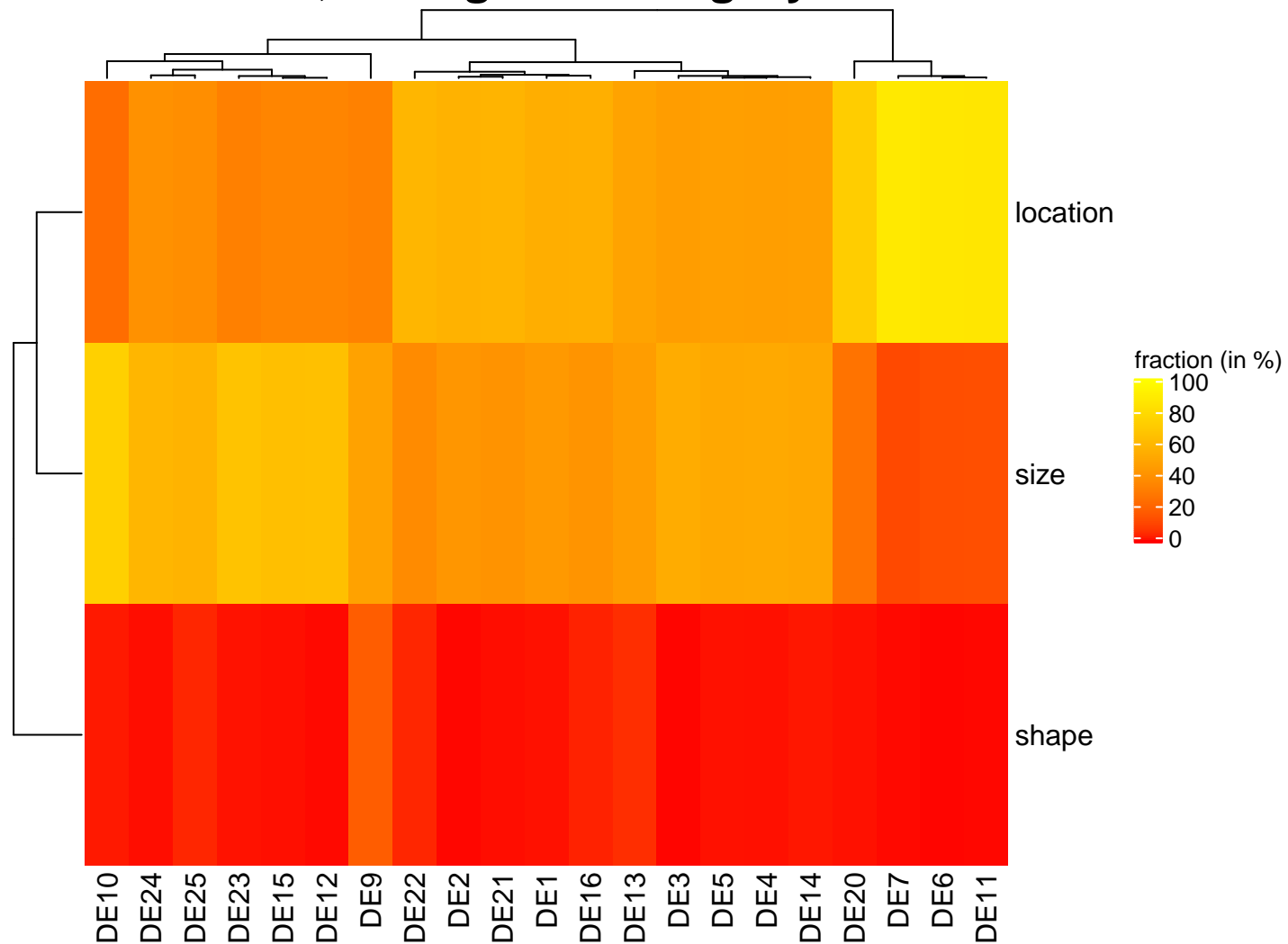

**C=50, strong DD: Category DP**

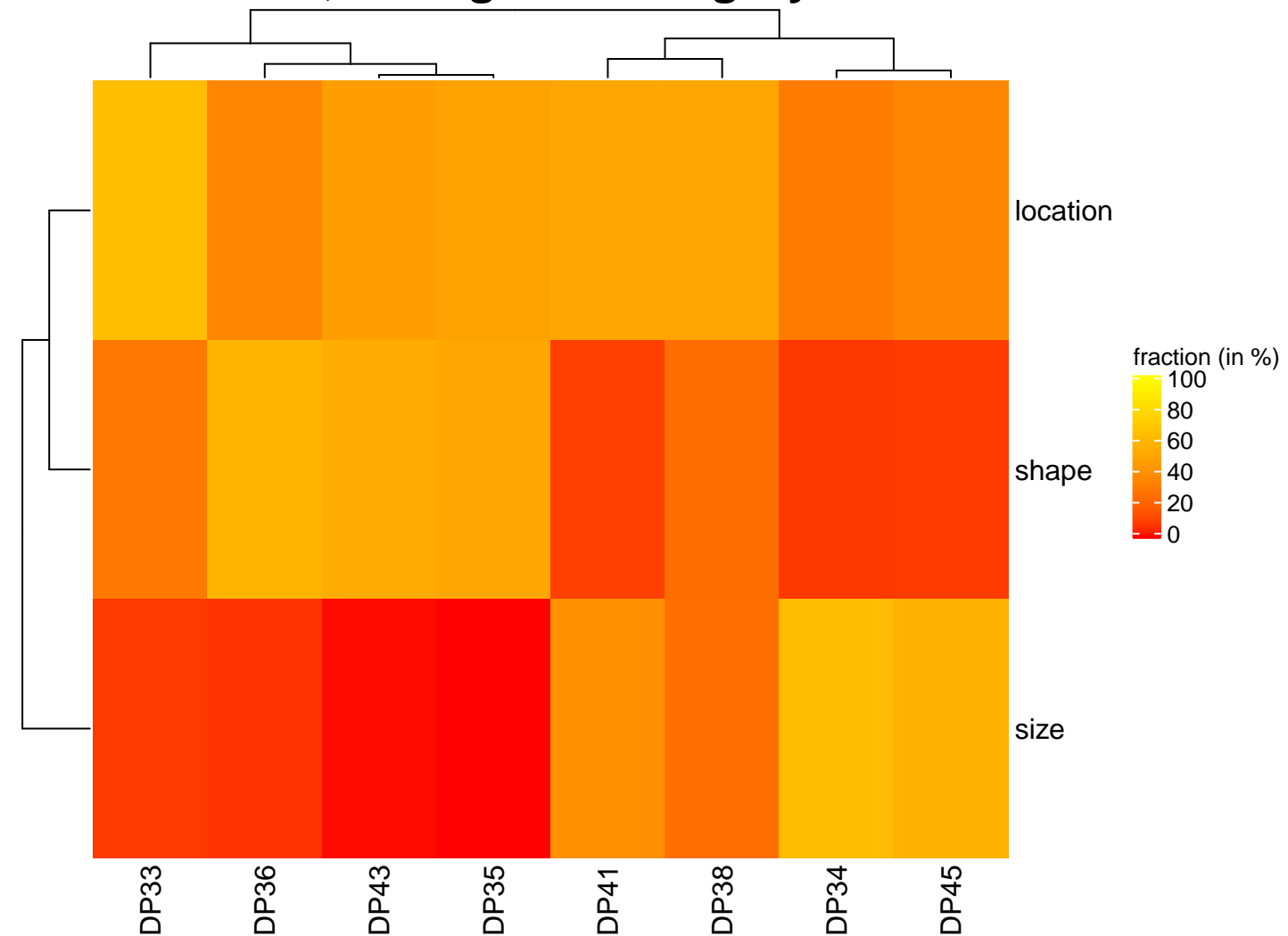

**C=50, strong DD: Category DM**

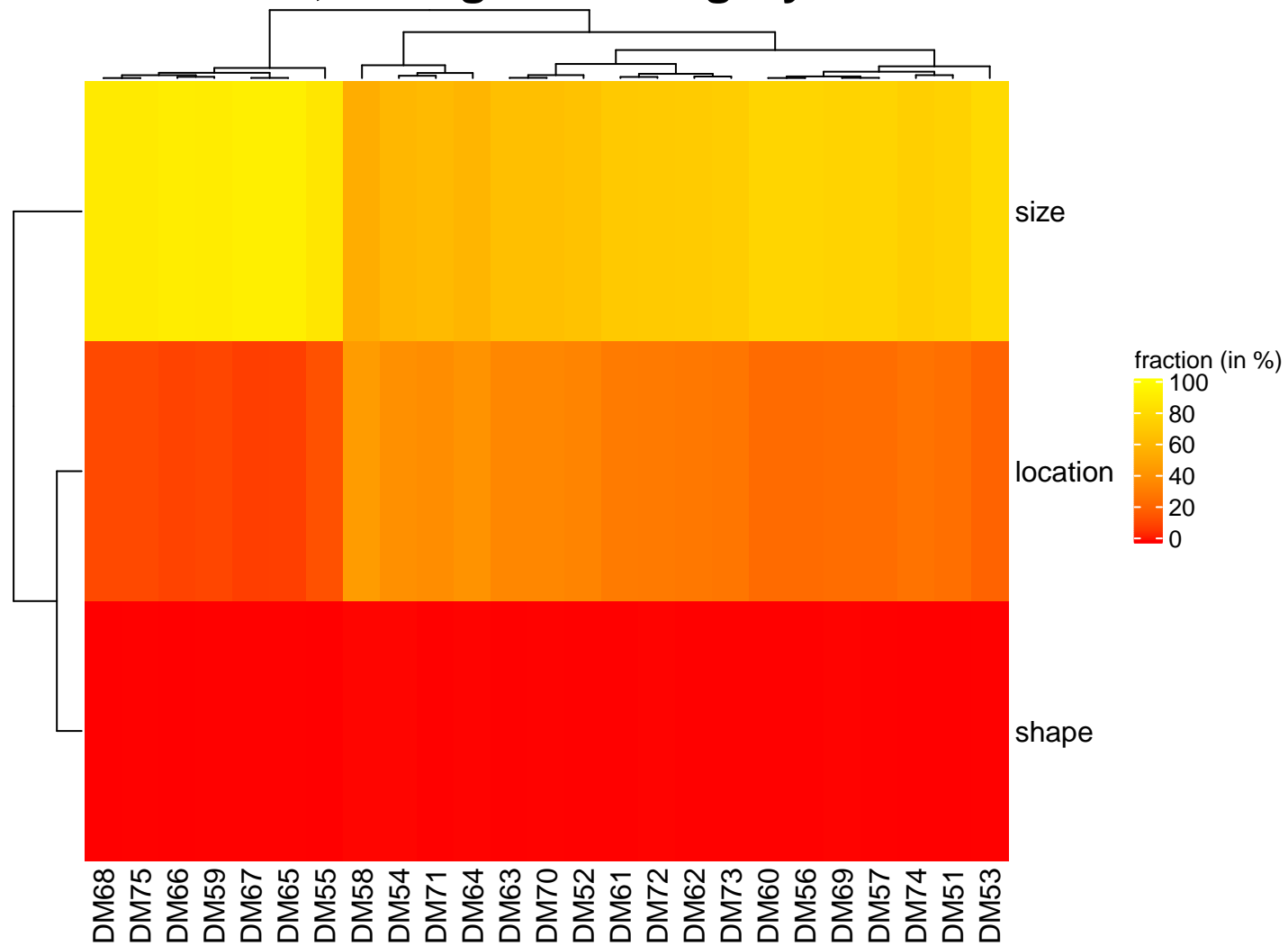

**C=50, strong DD: Category DB**

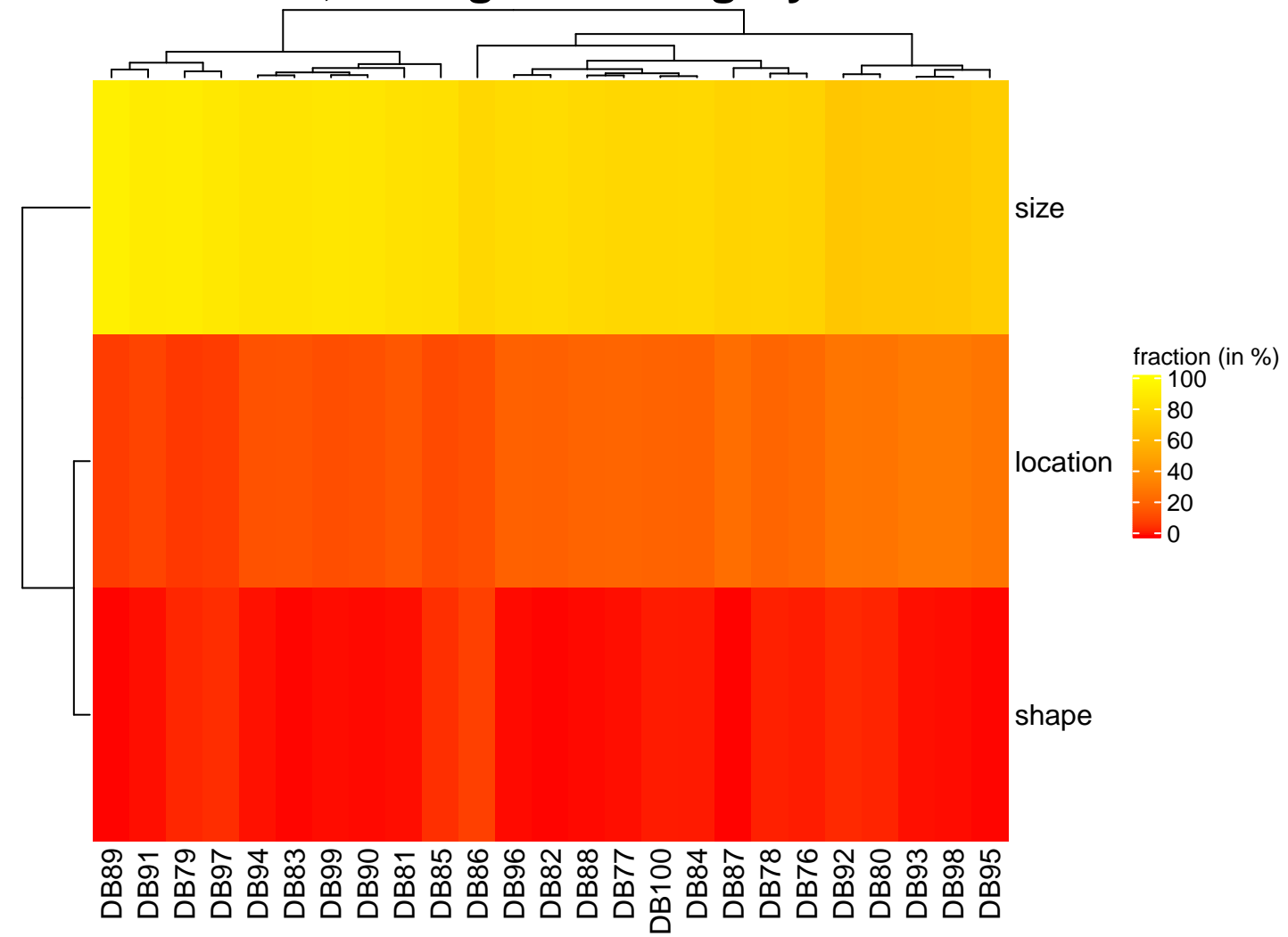

Supplement: btab226_Supplementary_Data [file btab226_supplementary_data.zip › Supplement_Revision2/Heatmaps_cells50_strongDD.pdf]

**C=50, weak DD: Category DE**

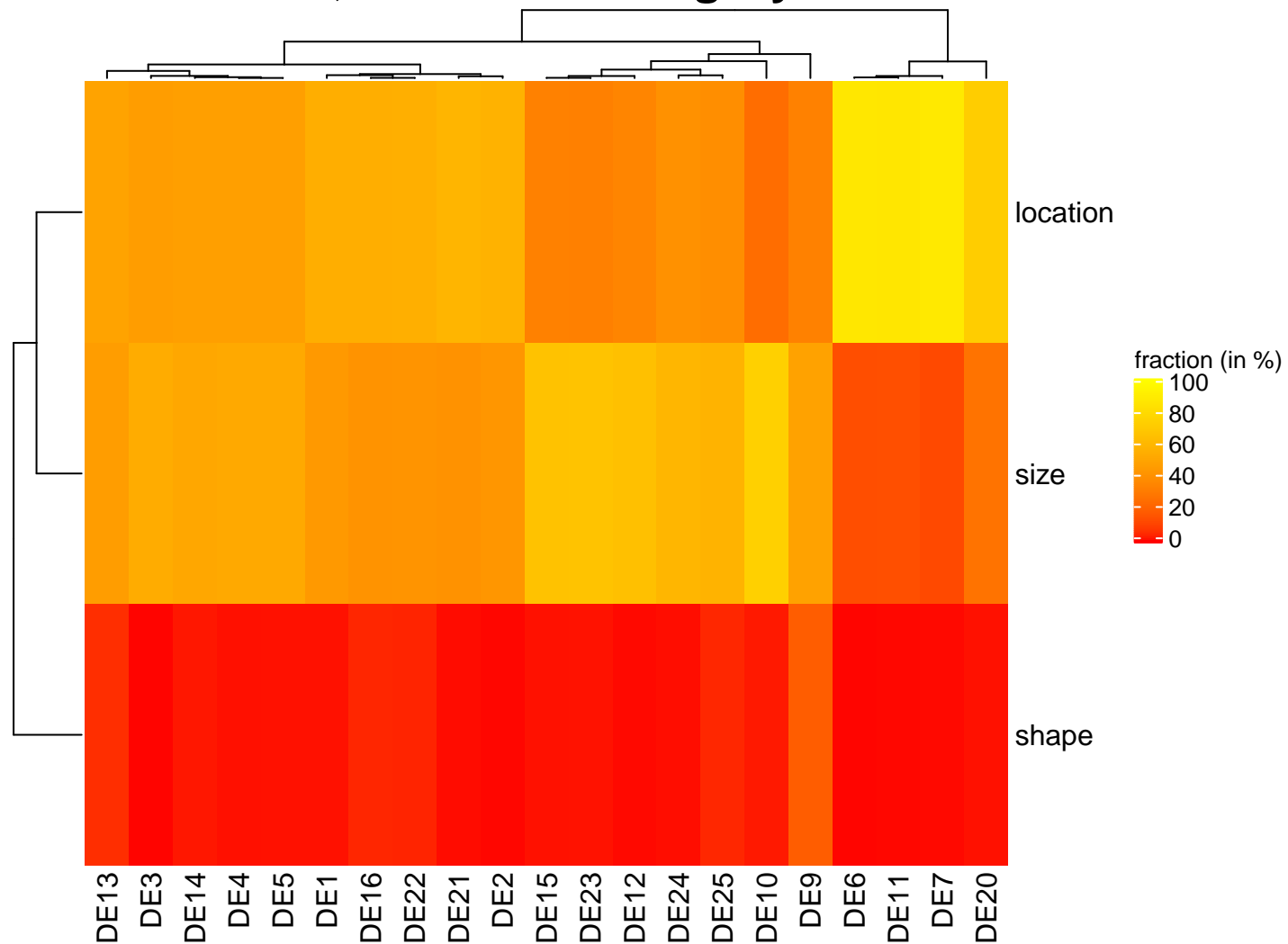

**C=50, weak DD: Category DP**

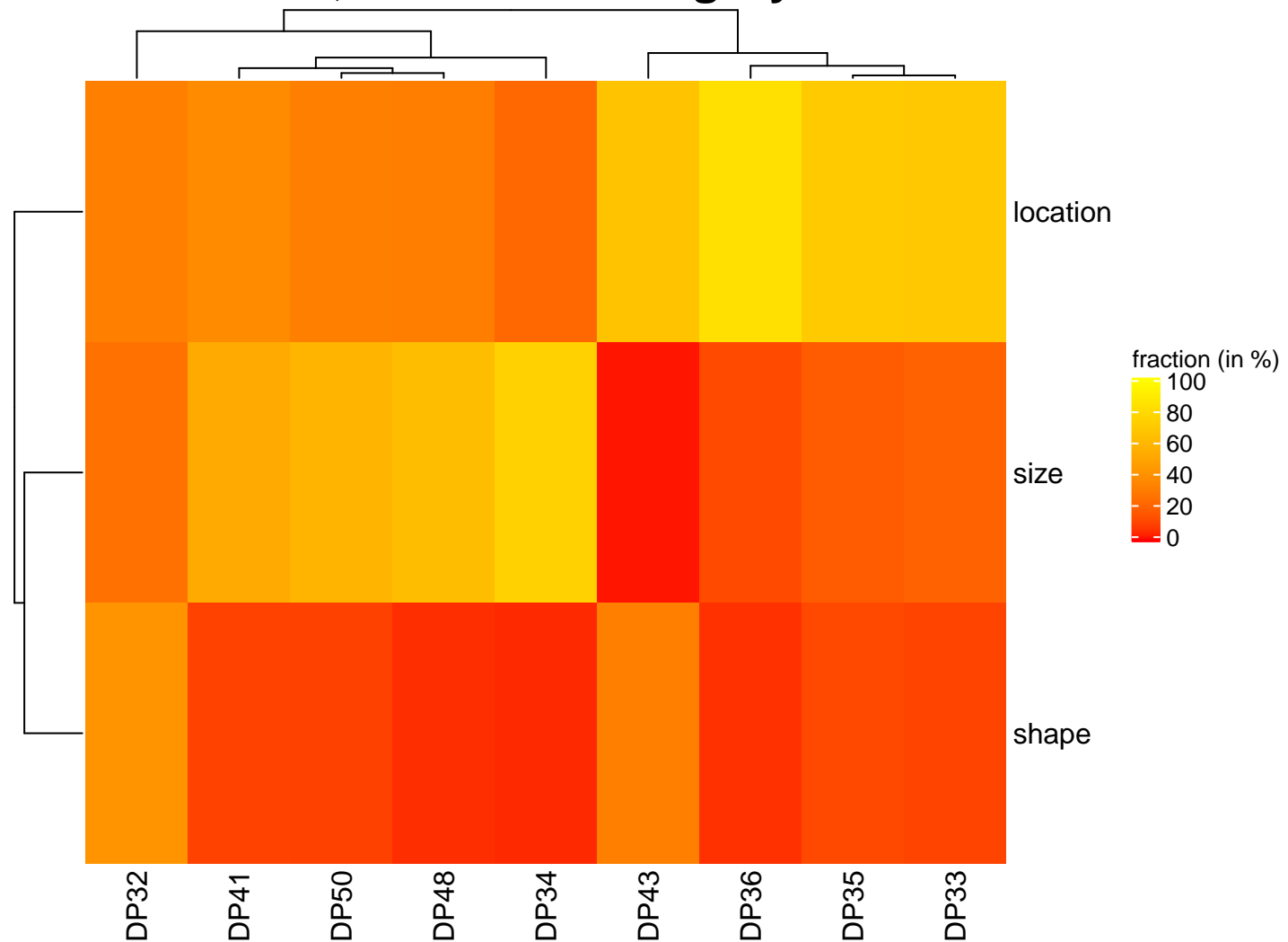

**C=50, weak DD: Category DM**

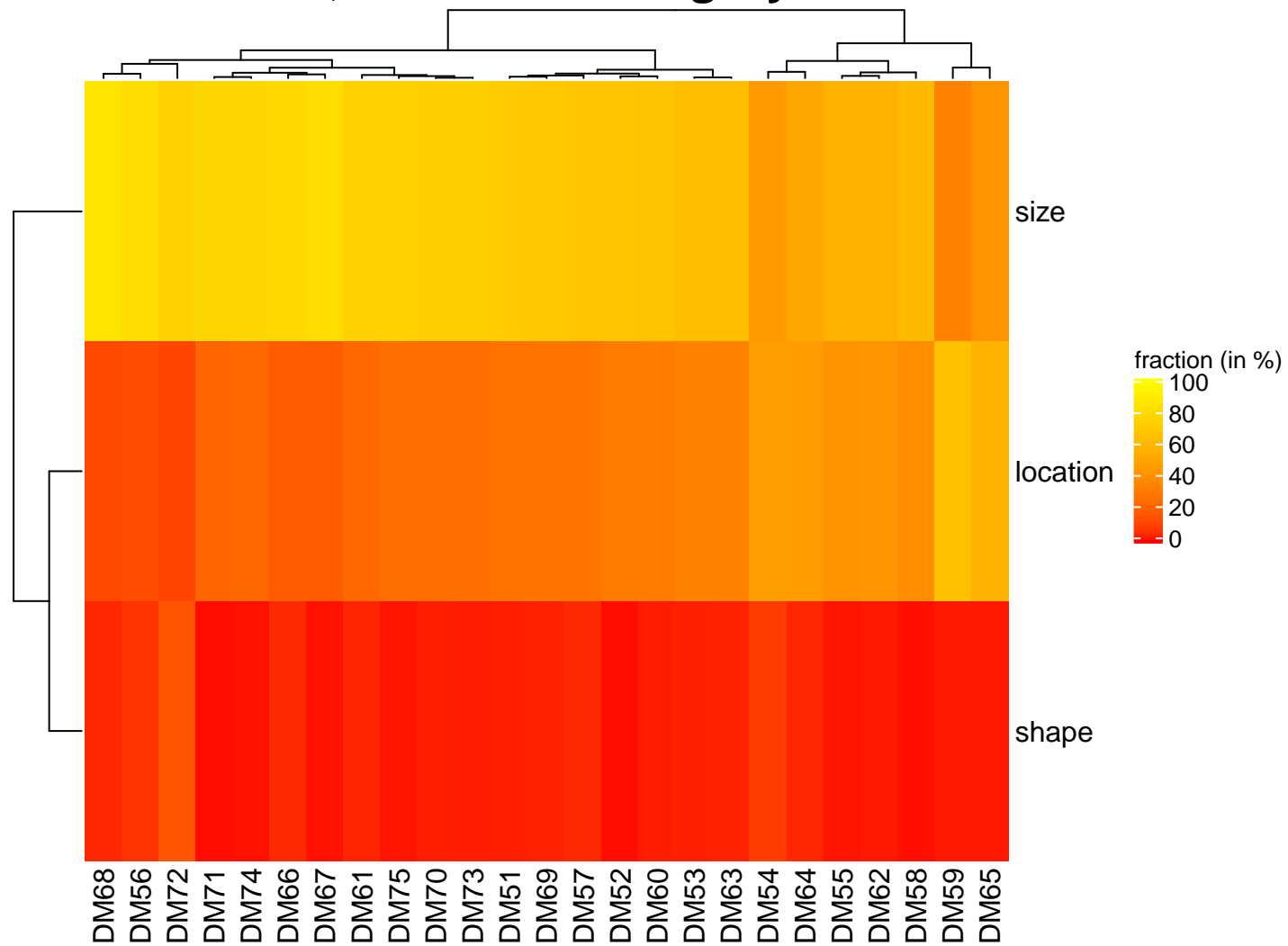

**C=50, weak DD: Category DB**

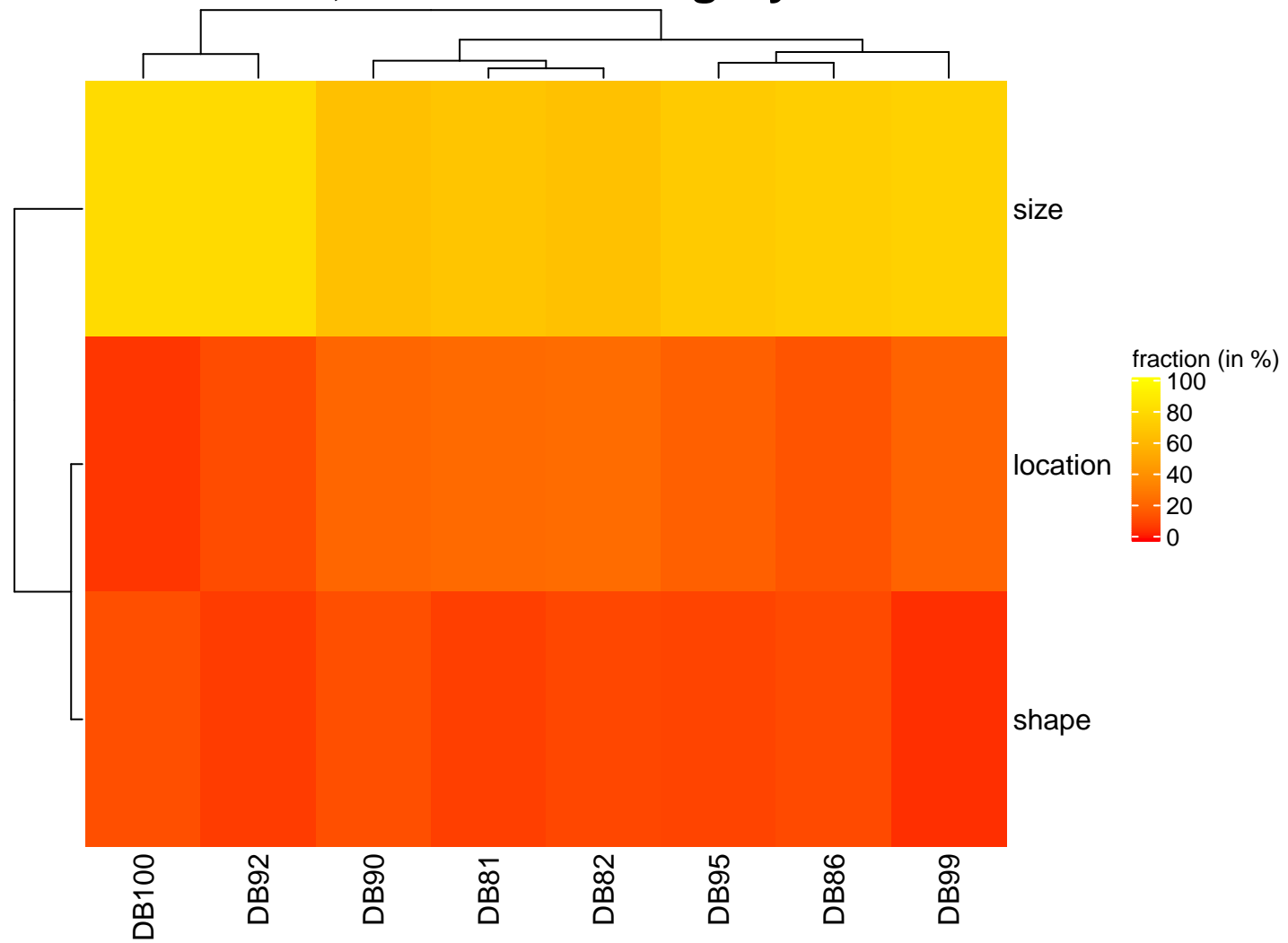

Supplement: btab226_Supplementary_Data [file btab226_supplementary_data.zip › Supplement_Revision2/Heatmaps_cells50_weakDD.pdf]

**LogNorm**

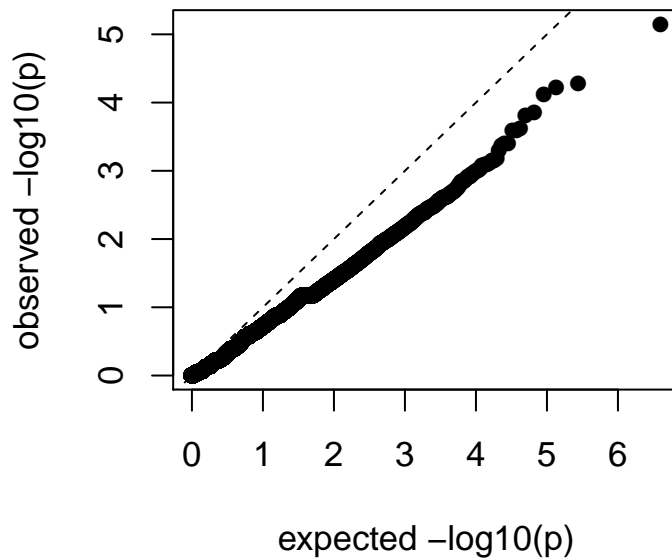

**CLRFeatNorm**

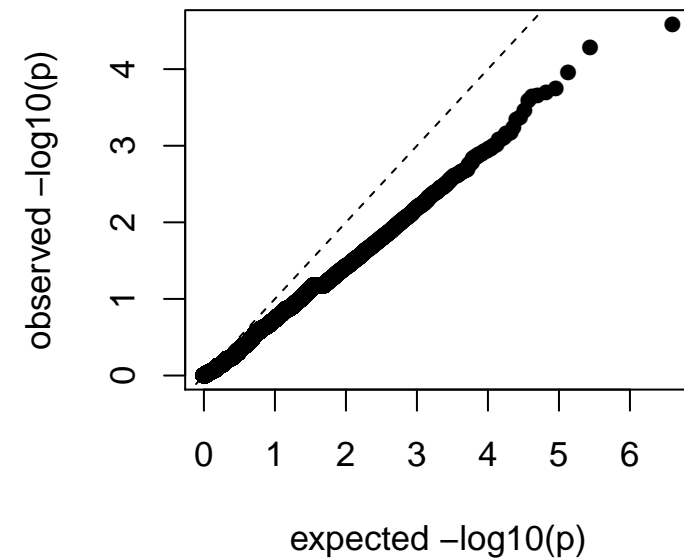

**CLRCellsNorm**

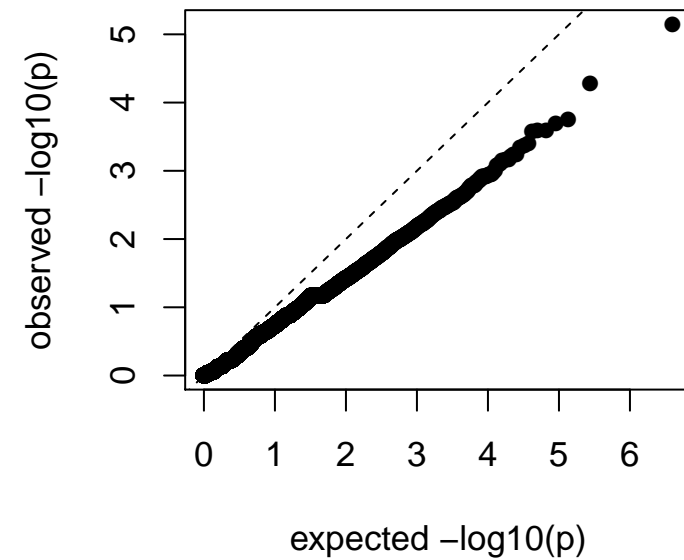

**RCNorm**

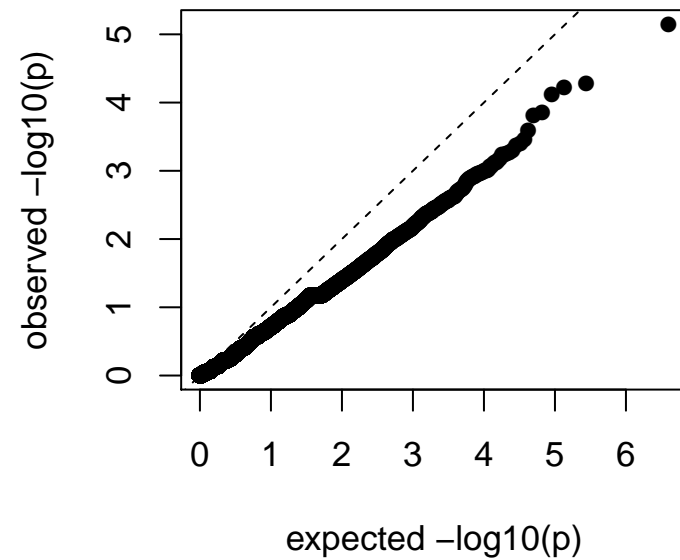

**DeconvNorm**

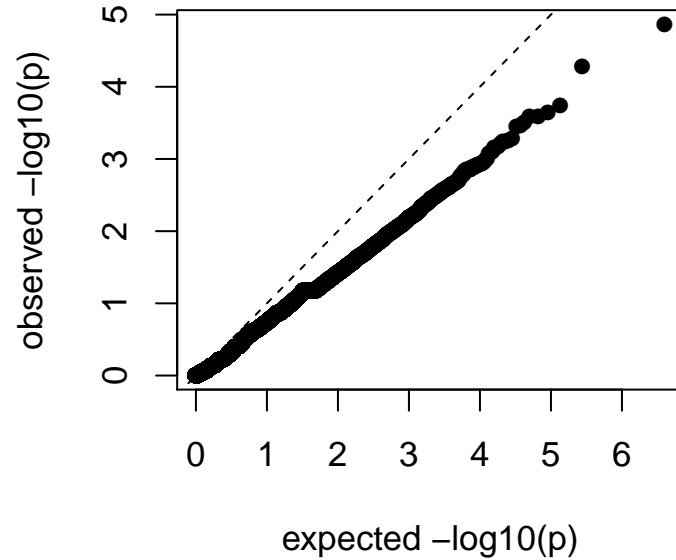

Supplement: btab226_Supplementary_Data [file btab226_supplementary_data.zip › Supplement_Revision2/QQPlots2Repl.pdf]

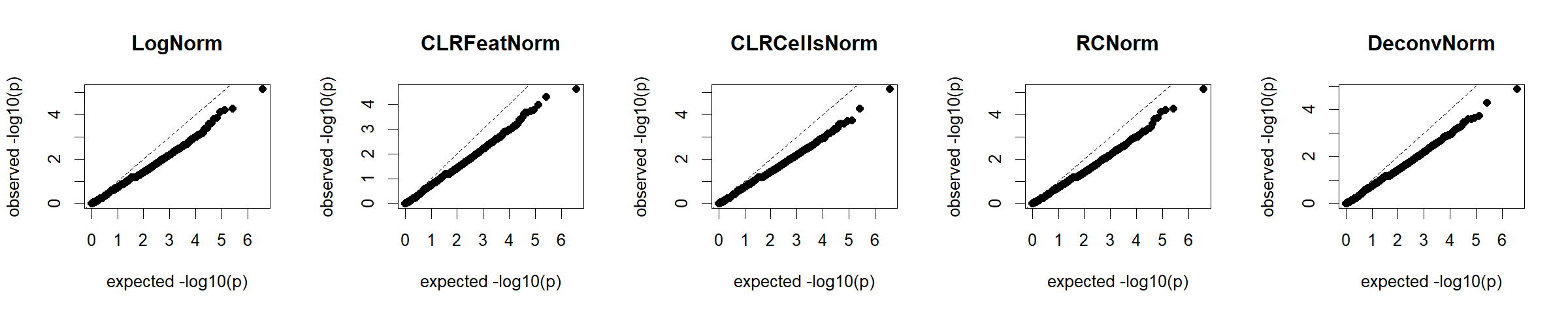

Supplement: btab226_Supplementary_Data [file btab226_supplementary_data.zip › Supplement_Revision2/QQPlots2Repl.png]

**LogNorm**

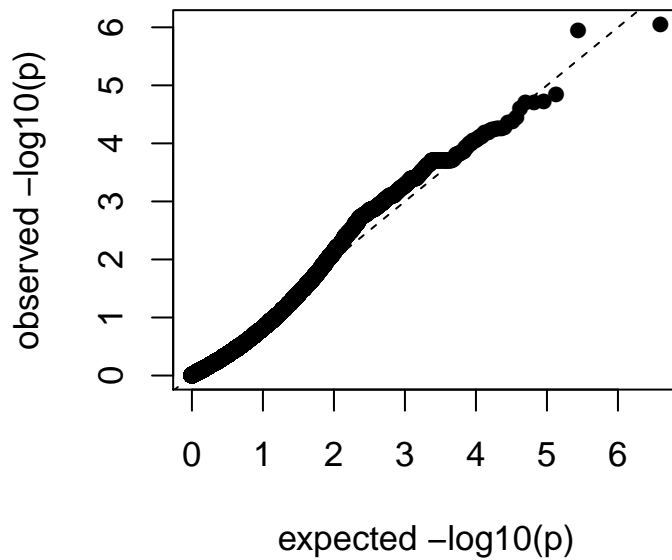

**CLRFeatNorm**

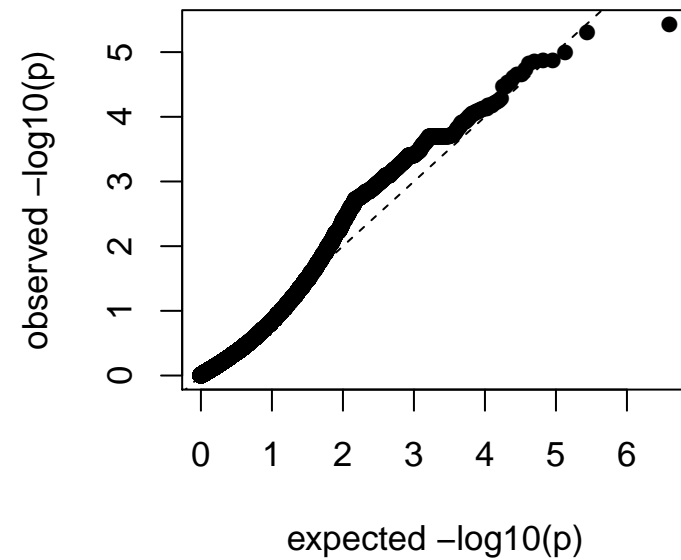

**CLRCellsNorm**

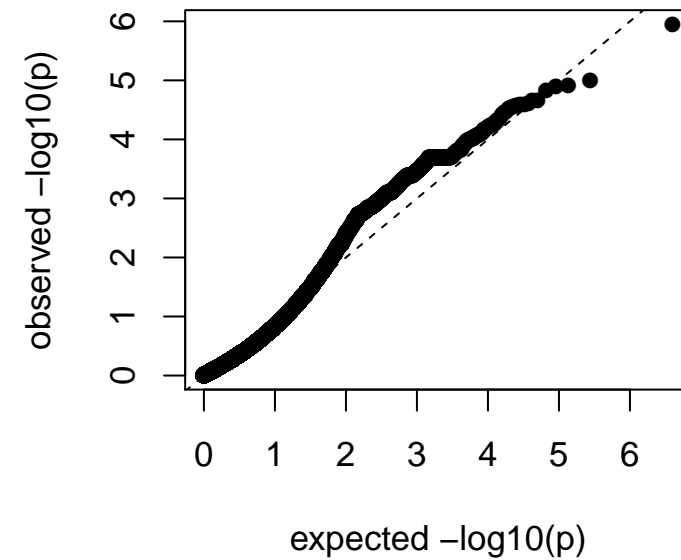

**RCNorm**

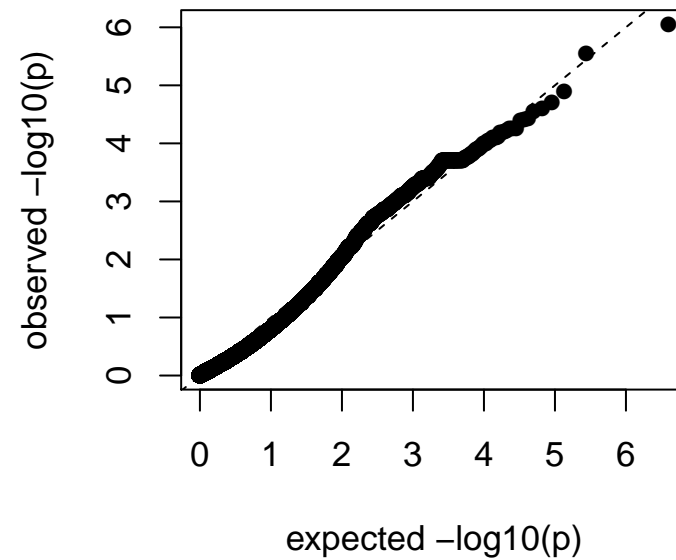

**DeconvNorm**

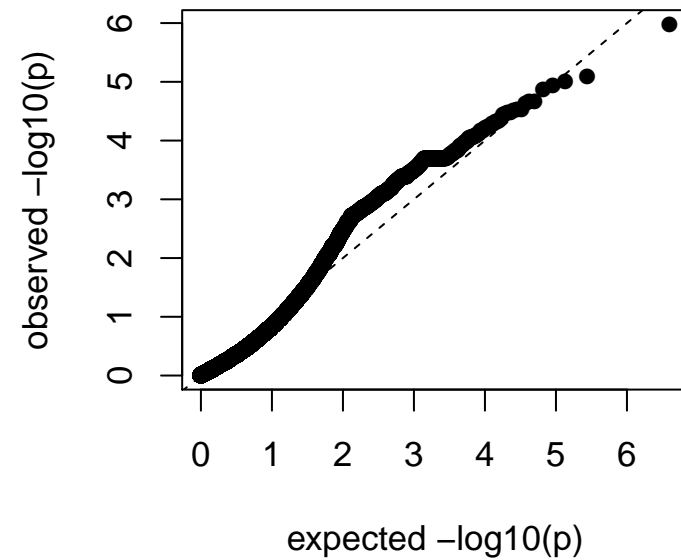

Supplement: btab226_Supplementary_Data [file btab226_supplementary_data.zip › Supplement_Revision2/QQPlots3Repl.pdf]

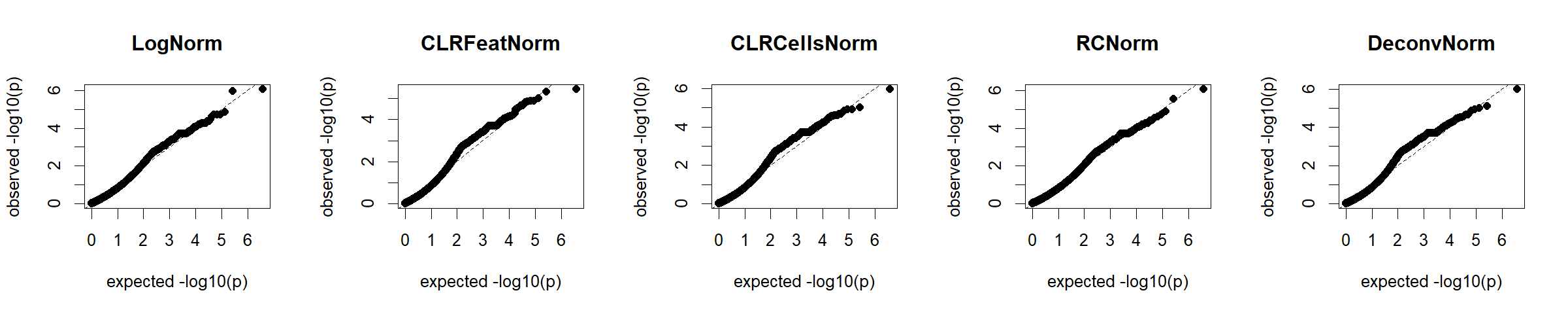

Supplement: btab226_Supplementary_Data [file btab226_supplementary_data.zip › Supplement_Revision2/QQPlots3Repl.png]

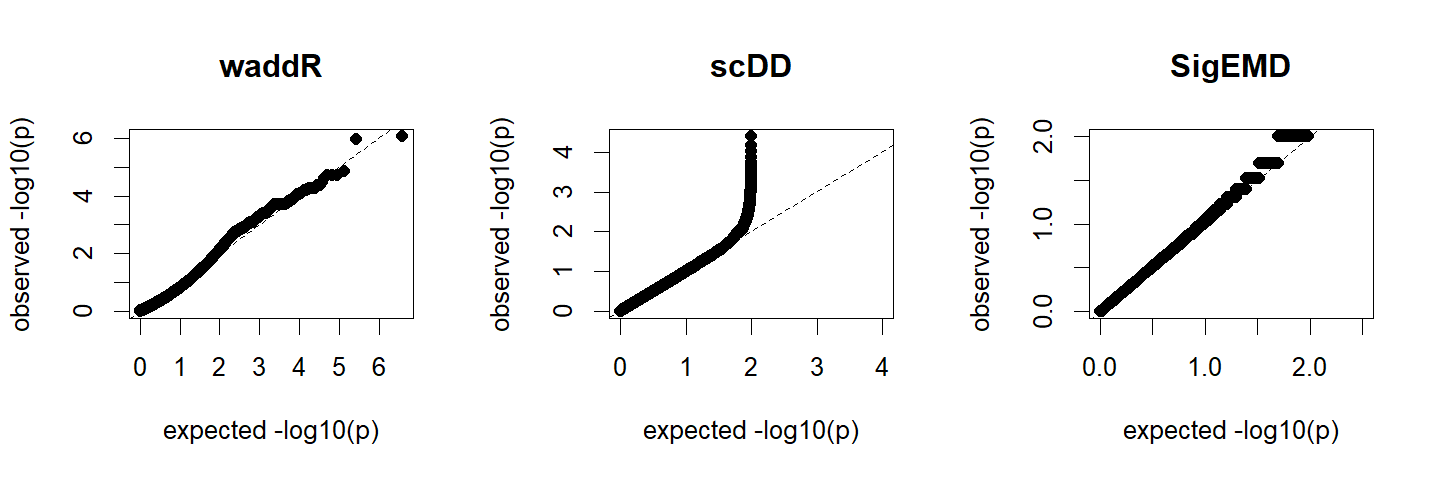

Supplement: btab226_Supplementary_Data [file btab226_supplementary_data.zip › Supplement_Revision2/QQPlots3Repl_RefMeth.png]

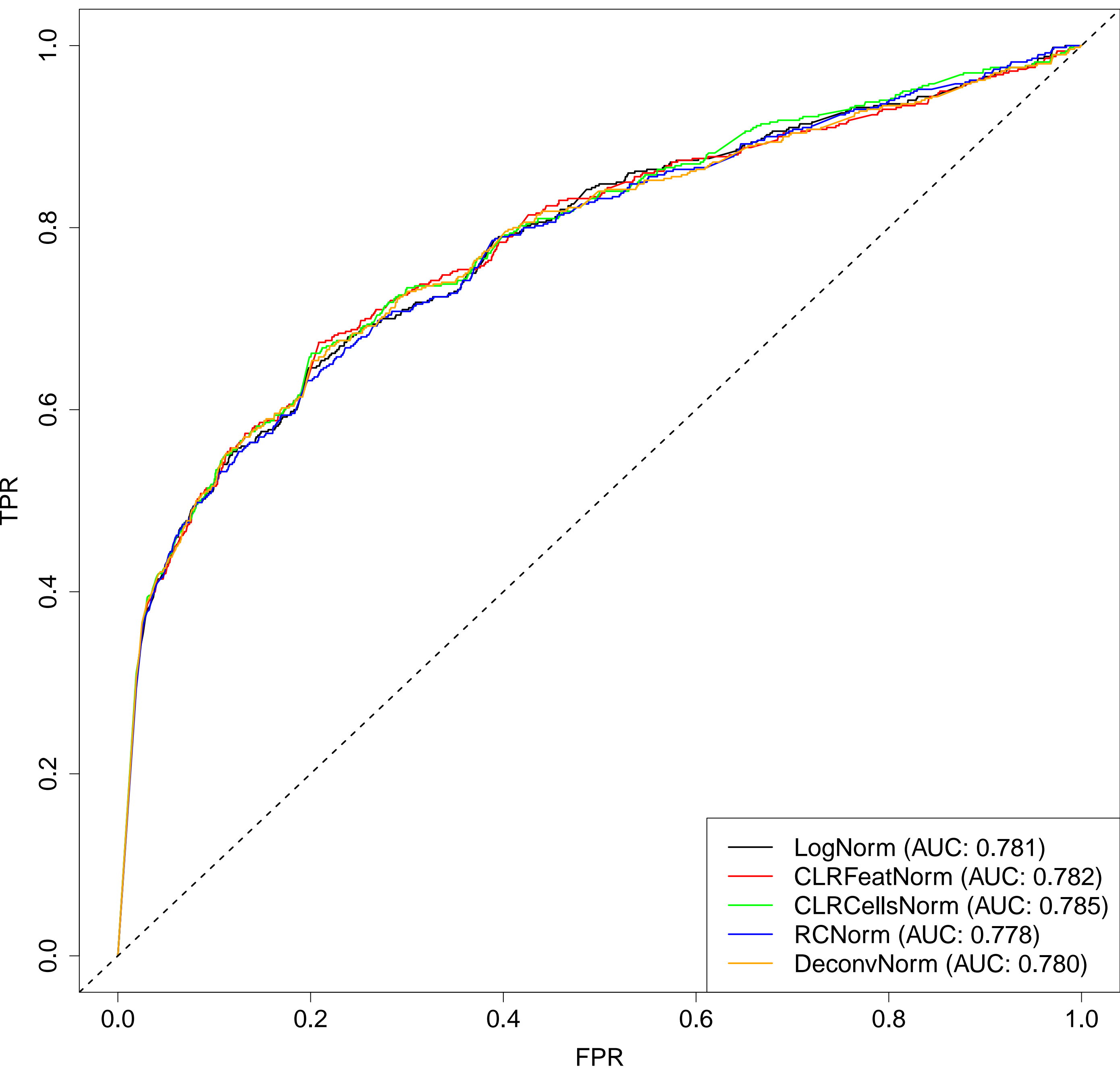

Supplement: btab226_Supplementary_Data [file btab226_supplementary_data.zip › Supplement_Revision2/ROC2Repl.pdf]

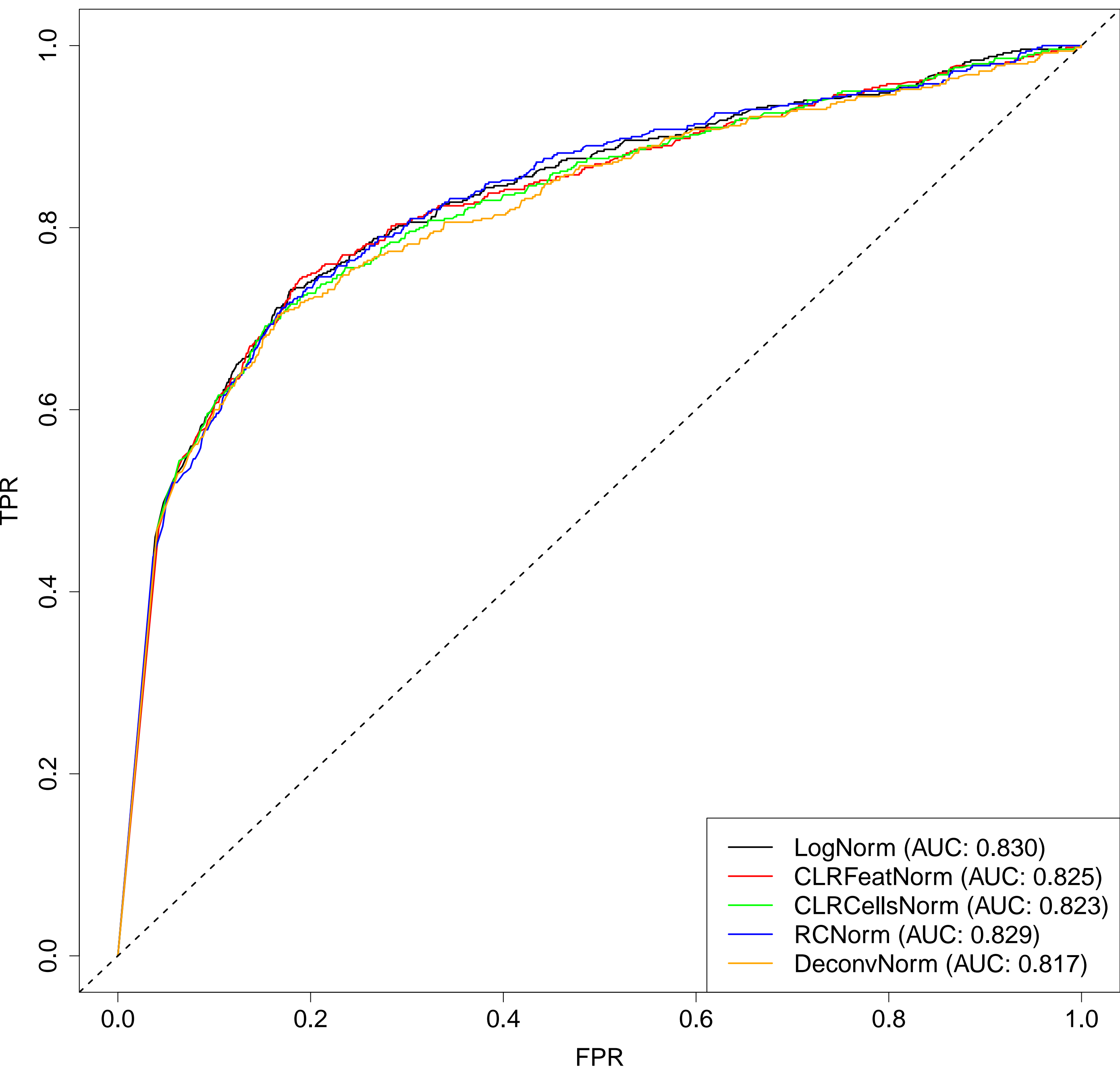

Supplement: btab226_Supplementary_Data [file btab226_supplementary_data.zip › Supplement_Revision2/ROC3Repl.pdf]

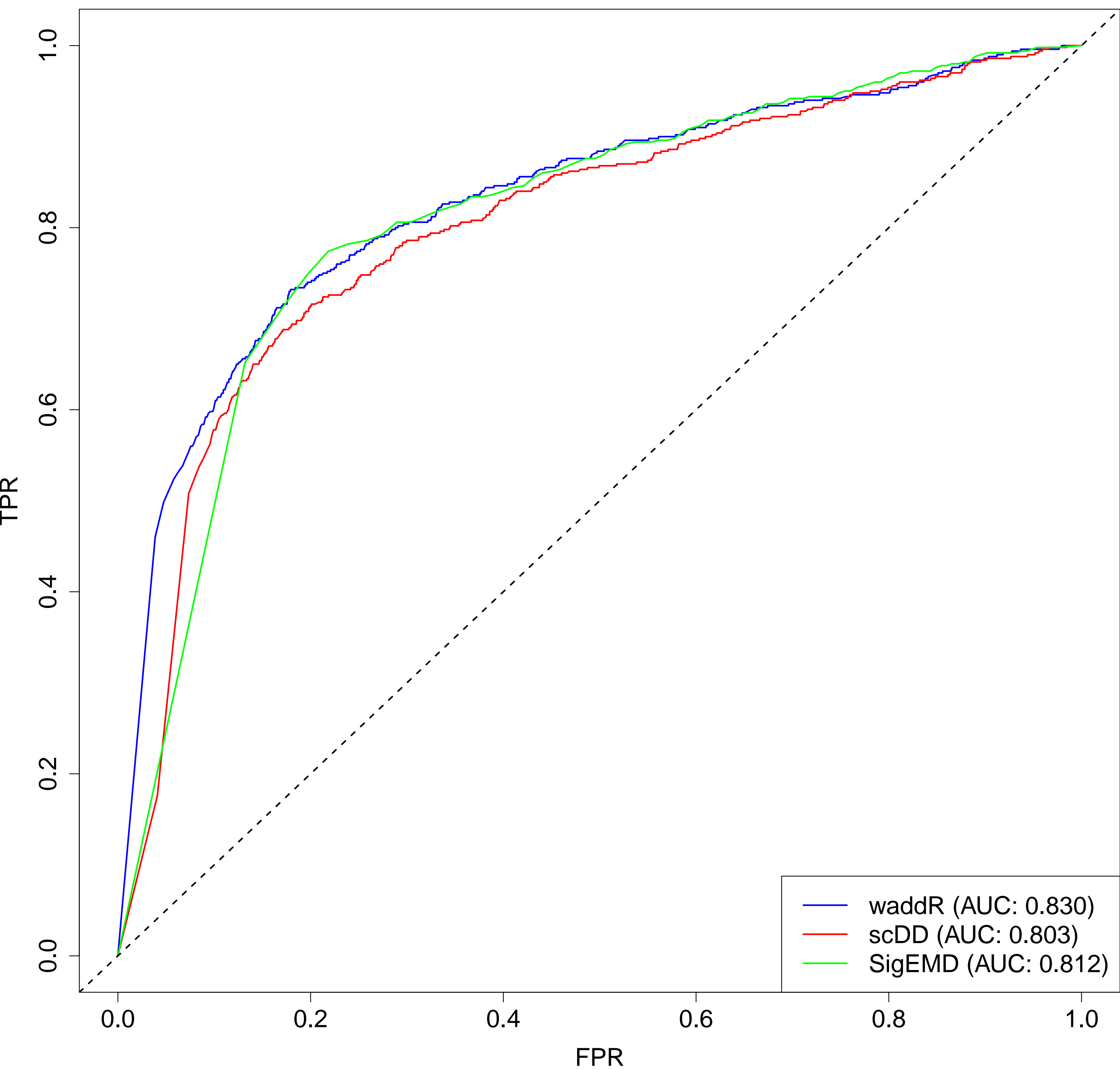

Supplement: btab226_Supplementary_Data [file btab226_supplementary_data.zip › Supplement_Revision2/ROC3Repl_RefMeth.pdf]

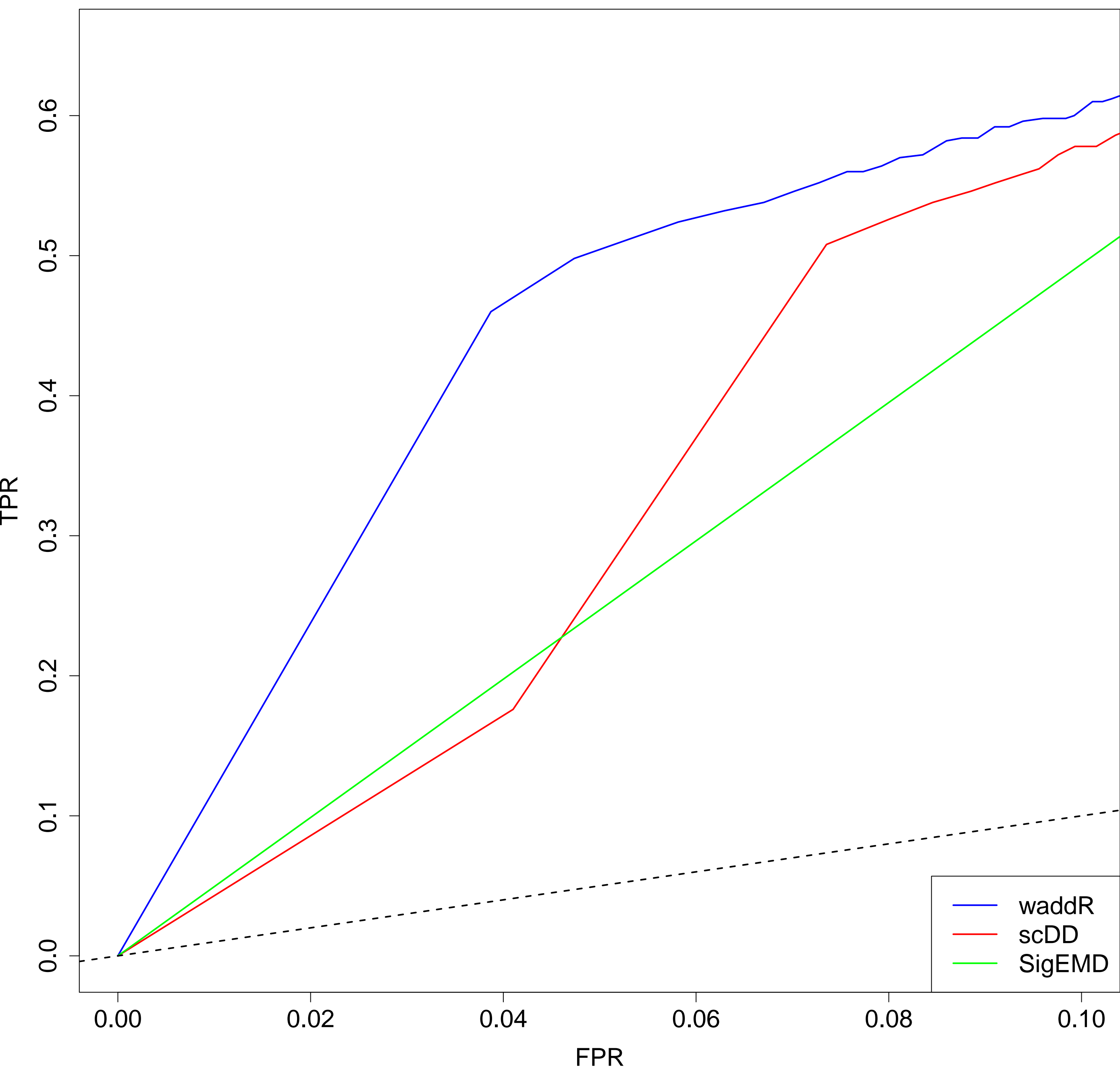

Supplement: btab226_Supplementary_Data [file btab226_supplementary_data.zip › Supplement_Revision2/ROC3Repl_RefMeth_Zoom.pdf]

fraction (in %)

100  
80  
40  
0

**C=50**  
**weak DD**

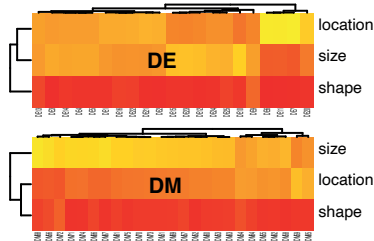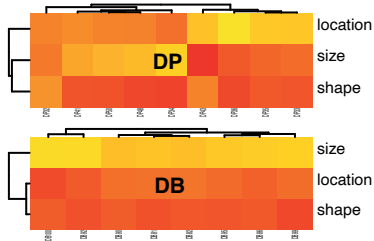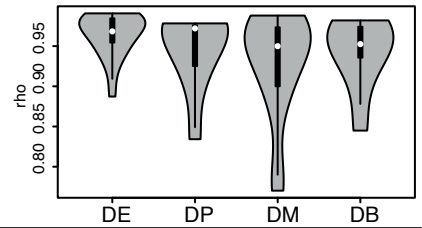

**C=50**  
**medium DD**

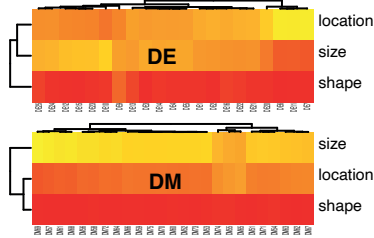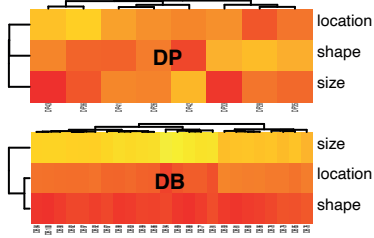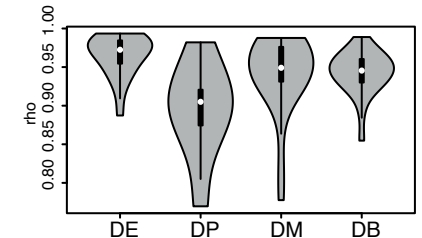

**C=50**  
**strong DD**

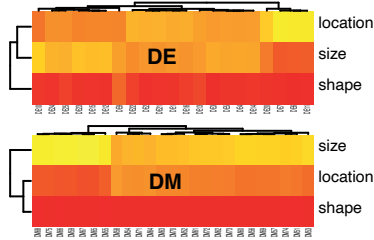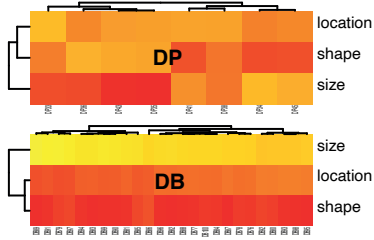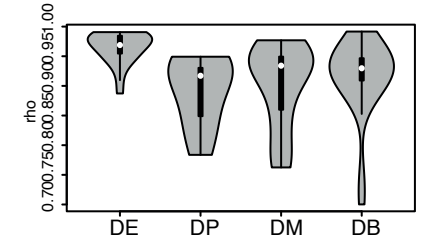

**C=100**  
**weak DD**

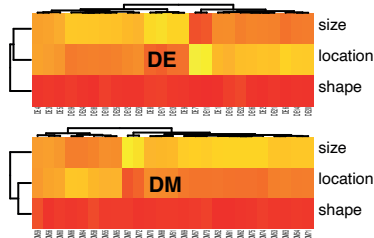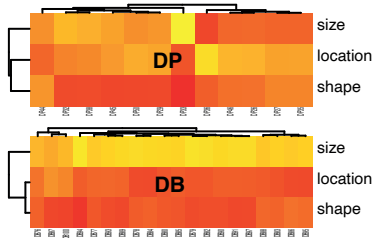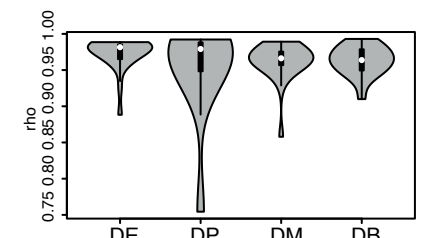

**C=100**  
**medium DD**

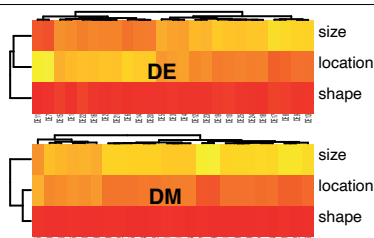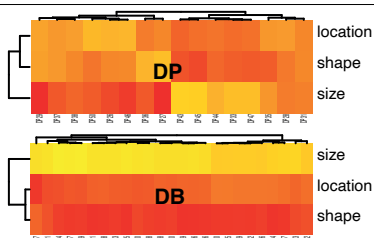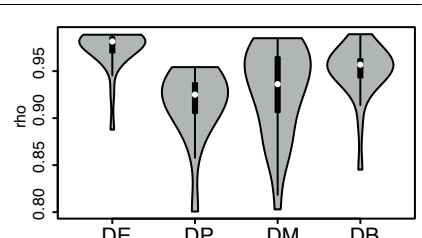

**C=100**  
**strong DD**

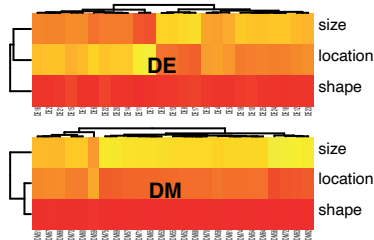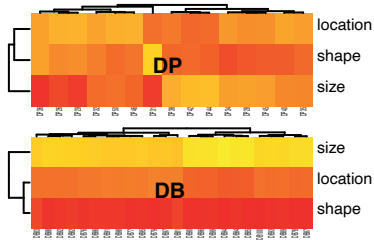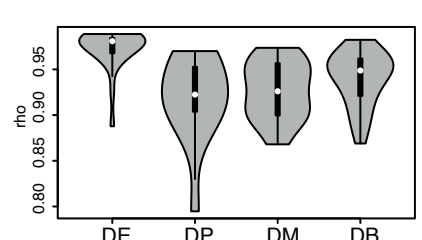

**C=500**  
**weak DD**

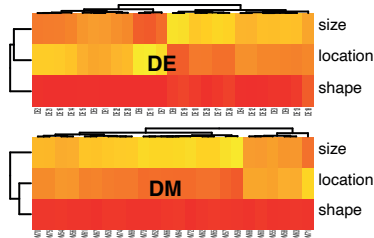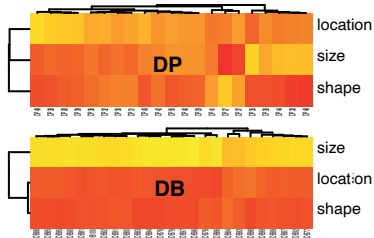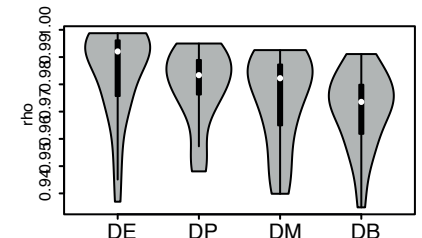

**C=500**  
**medium DD**

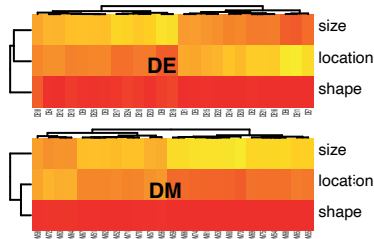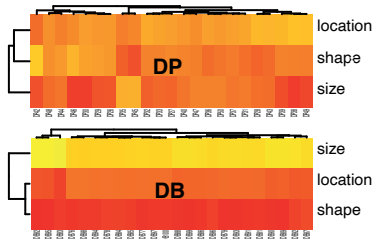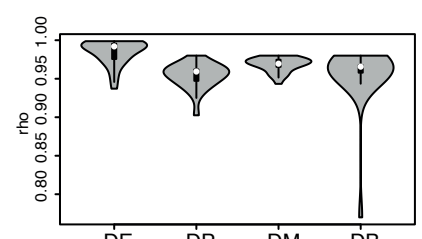

Supplement: btab226_Supplementary_Data [file btab226_supplementary_data.zip › Supplement_Revision2/SF_17_34.pdf]

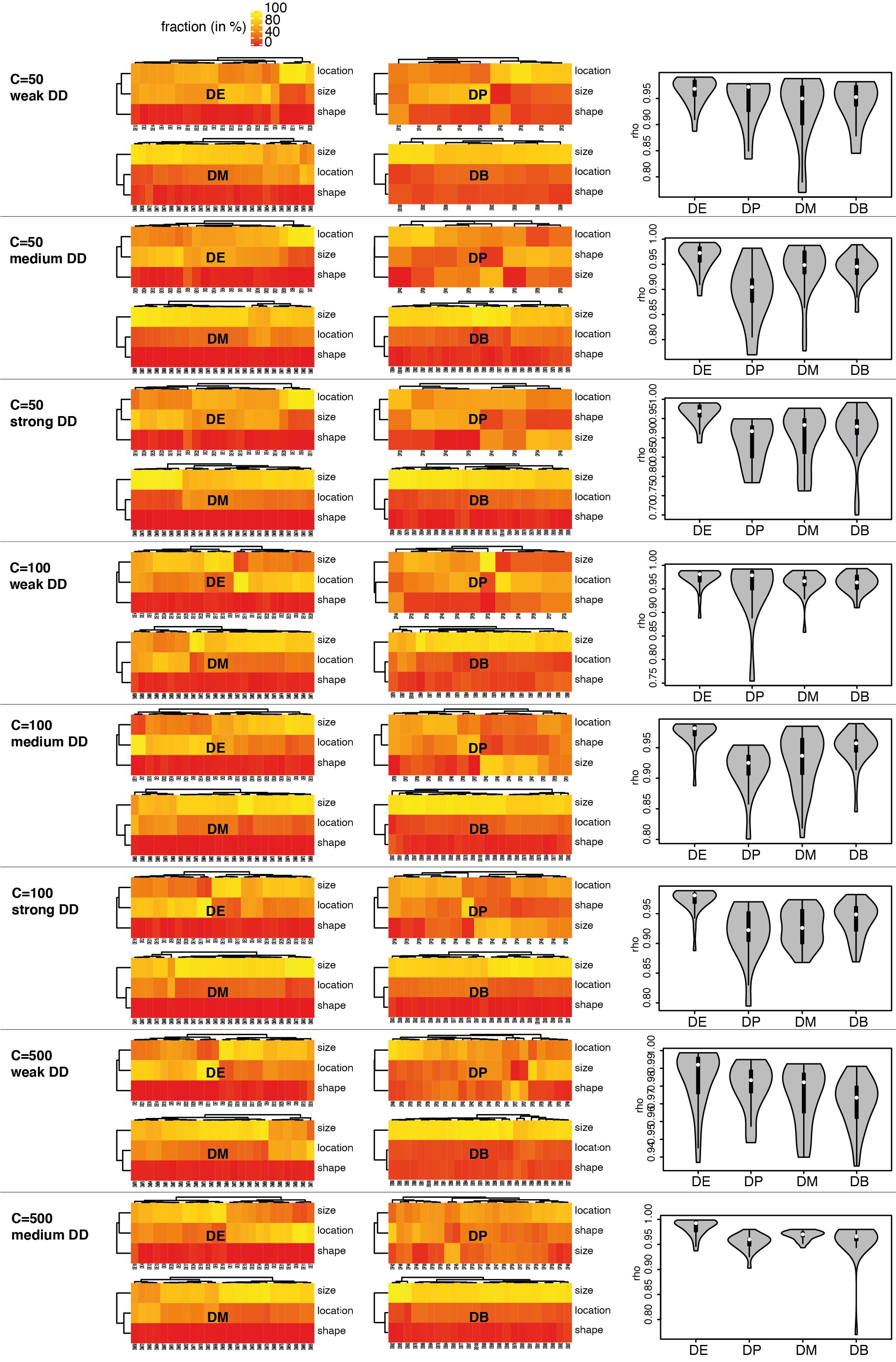

Supplement: btab226_Supplementary_Data [file btab226_supplementary_data.zip › Supplement_Revision2/SF_17_34.png]

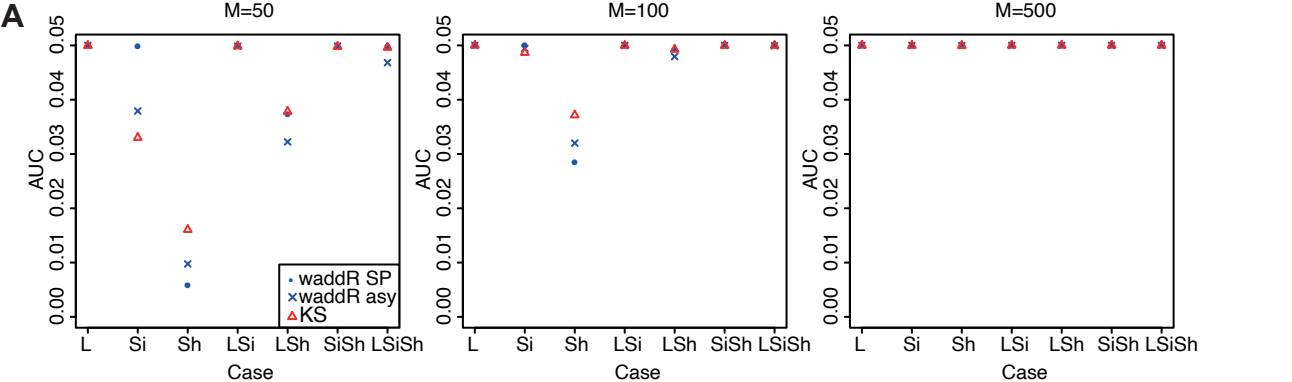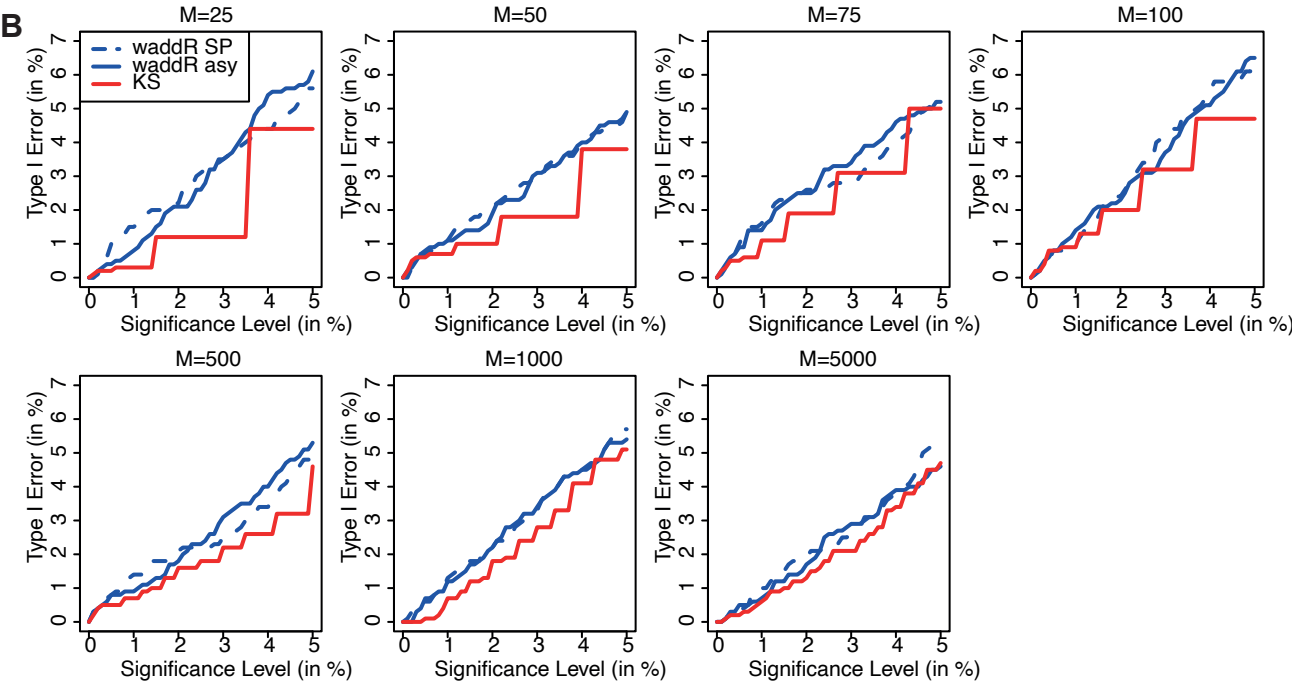

Supplement: btab226_Supplementary_Data [file btab226_supplementary_data.zip › Supplement_Revision2/SF_4_5.pdf]

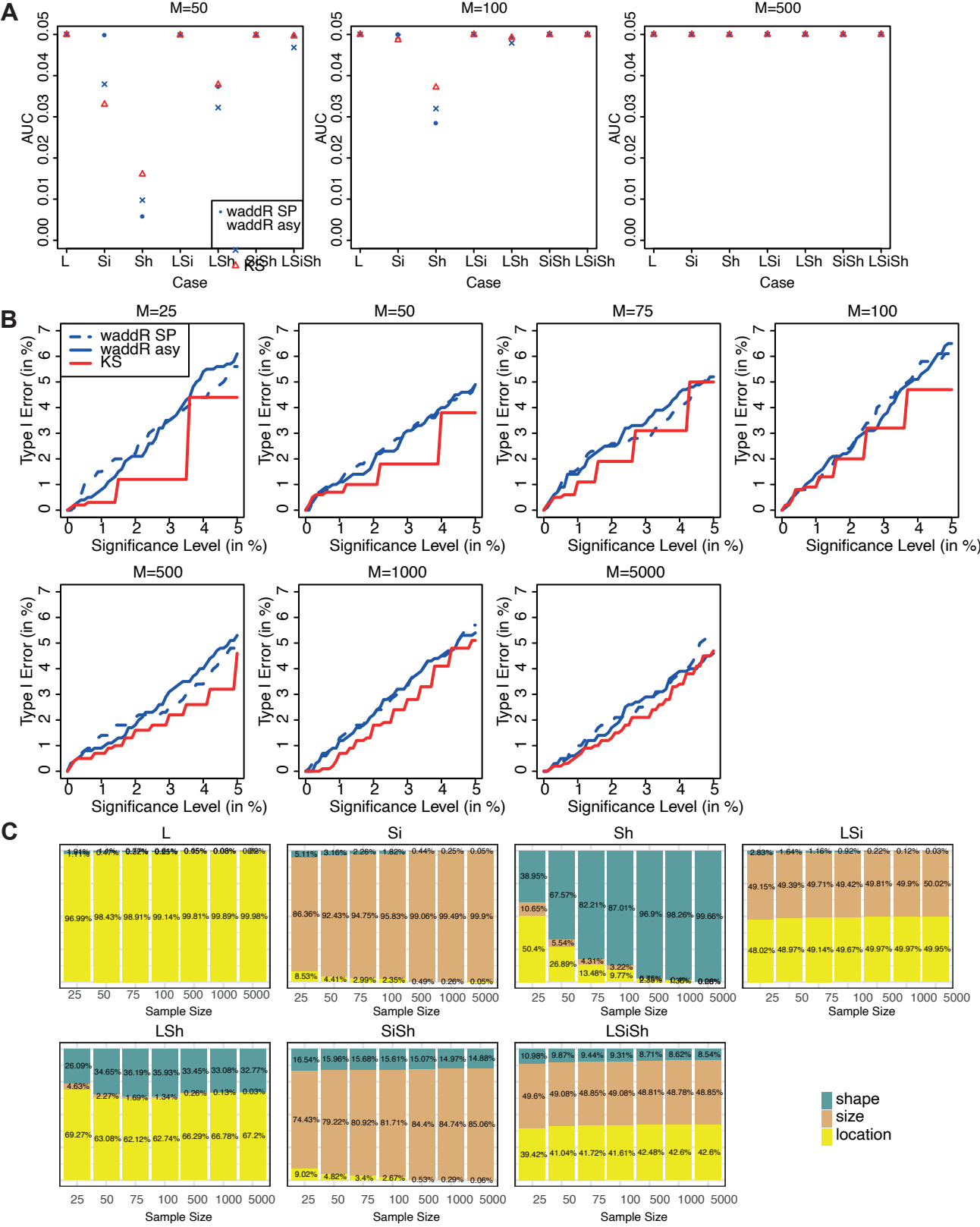

Supplement: btab226_Supplementary_Data [file btab226_supplementary_data.zip › Supplement_Revision2/SF_4_5_6.pdf]

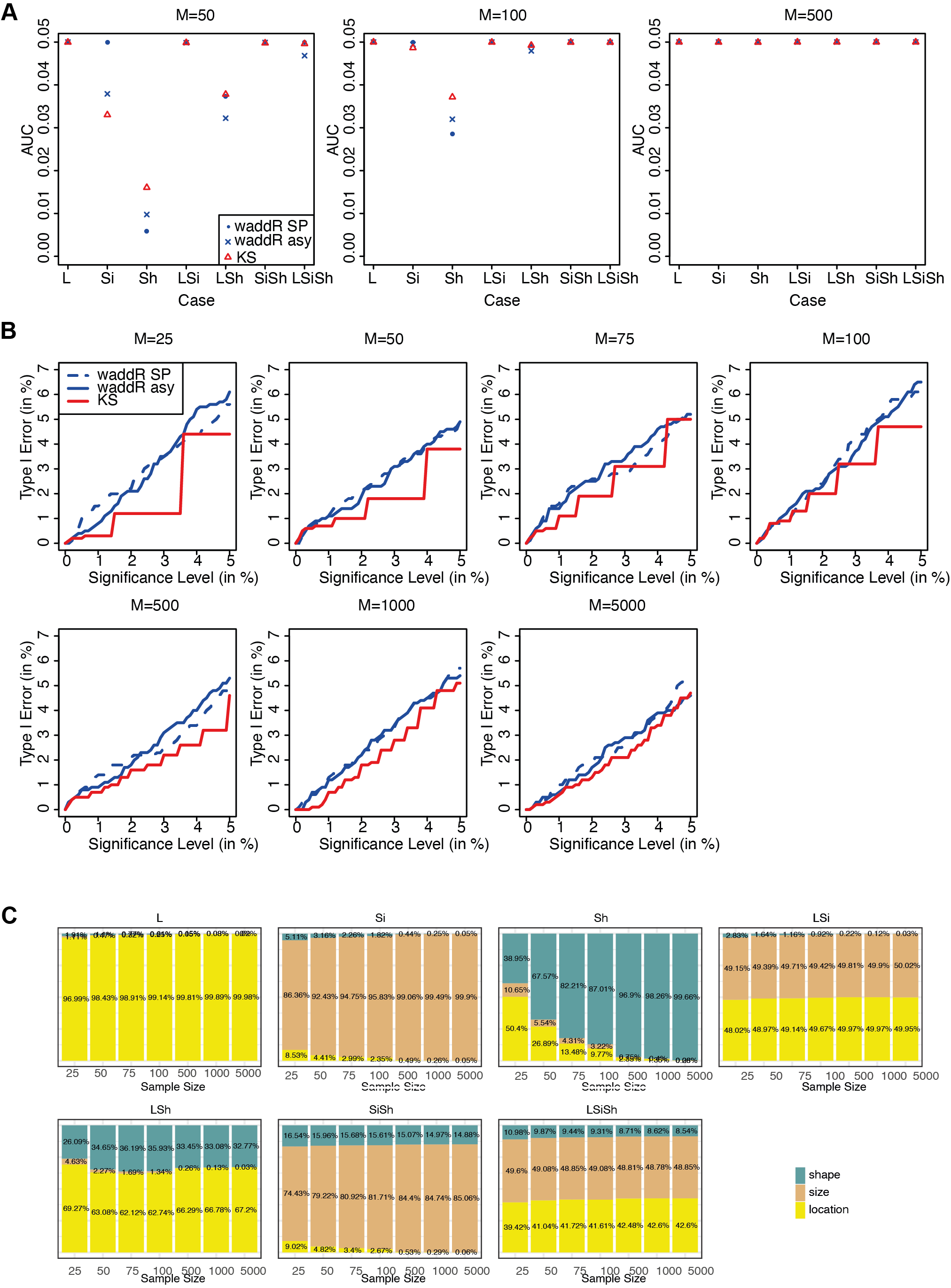

Supplement: btab226_Supplementary_Data [file btab226_supplementary_data.zip › Supplement_Revision2/SF_4_5_6.png]

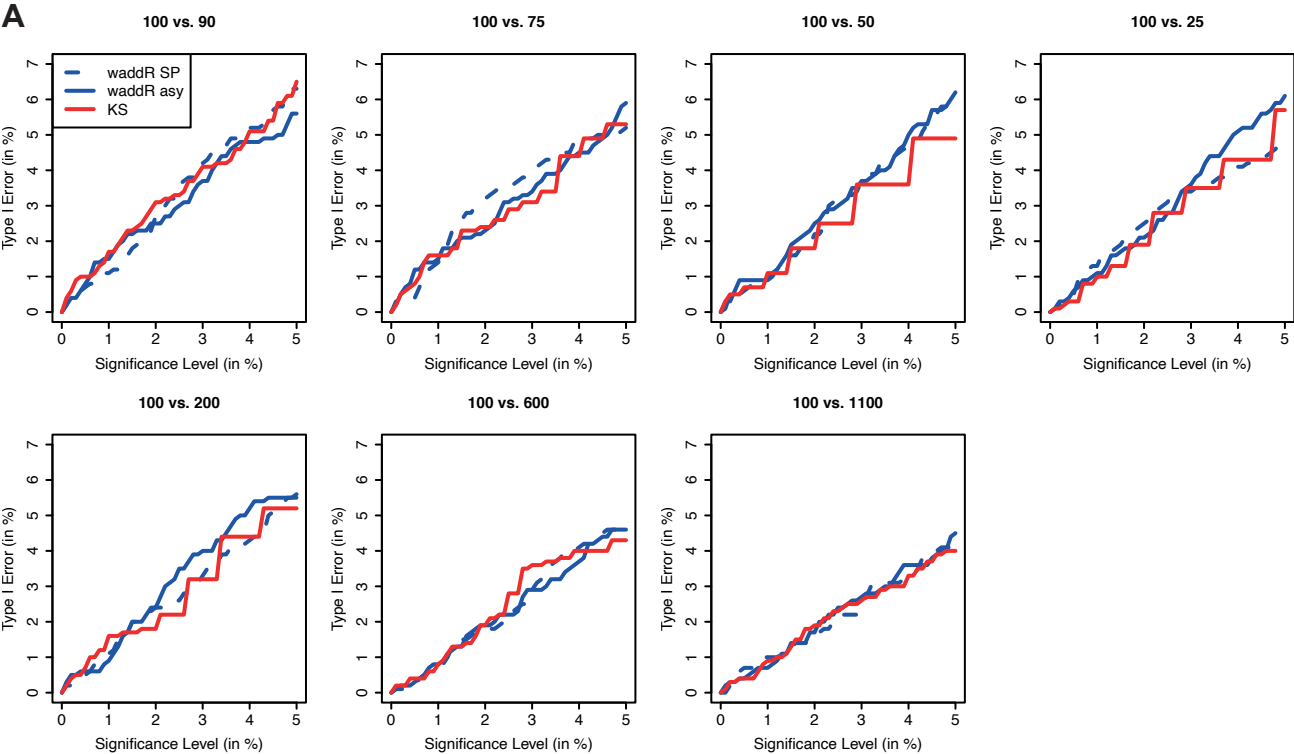

**B**

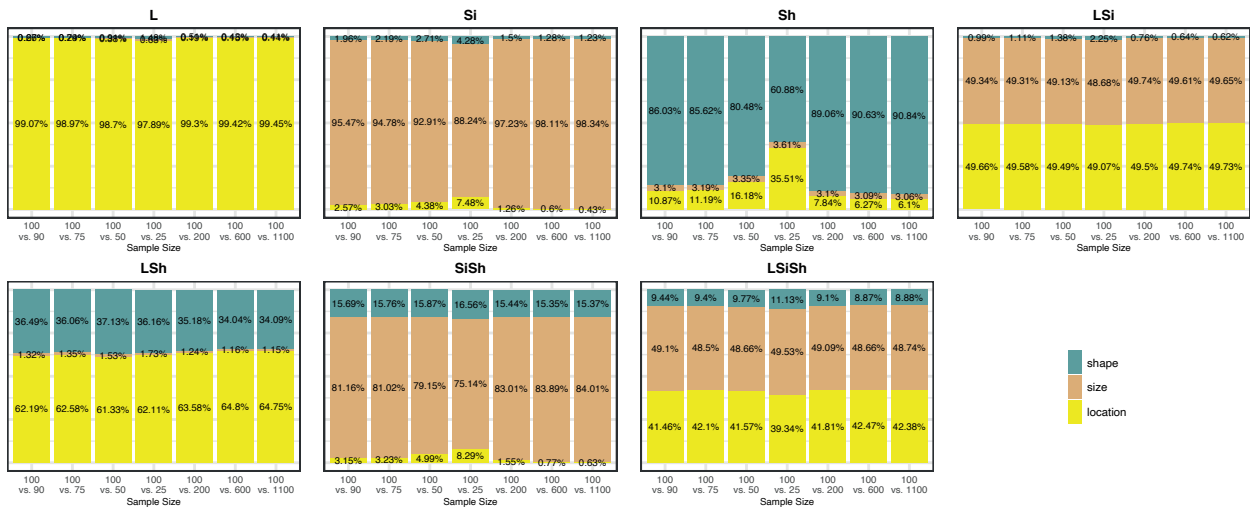

Supplement: btab226_Supplementary_Data [file btab226_supplementary_data.zip › Supplement_Revision2/SF_9_10.pdf]

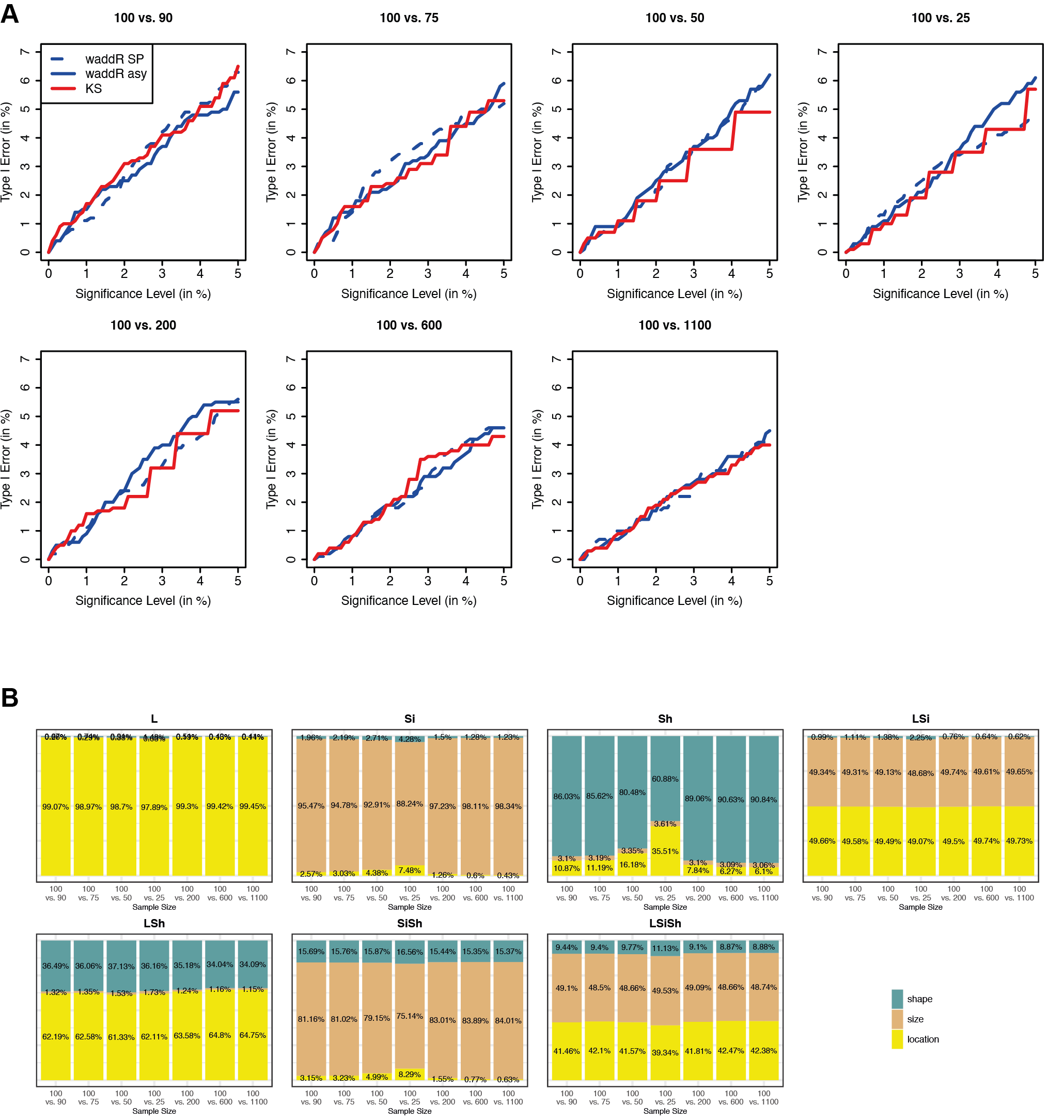

Supplement: btab226_Supplementary_Data [file btab226_supplementary_data.zip › Supplement_Revision2/SF_9_10.png]

**LogNorm**

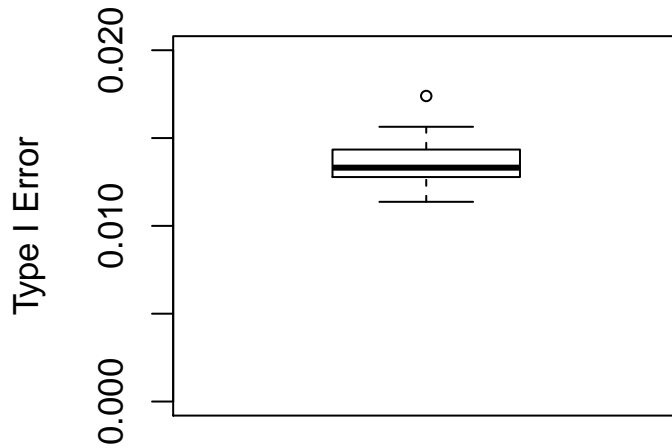

**CLRNormFeat**

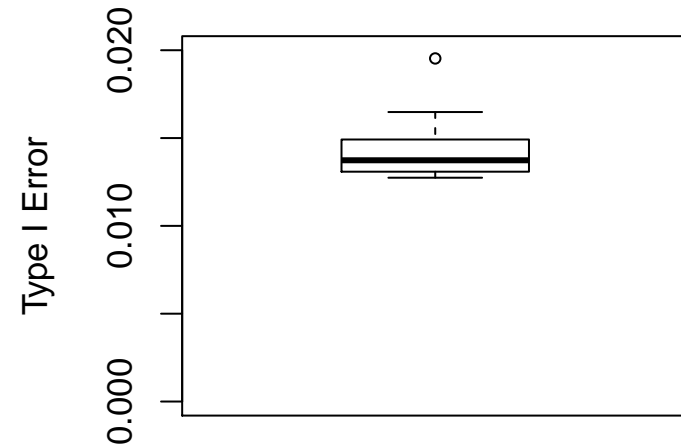

**CLRNormCells**

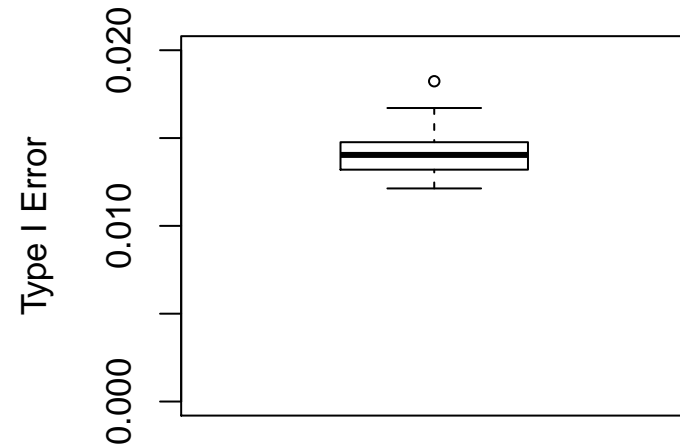

**RCNorm**

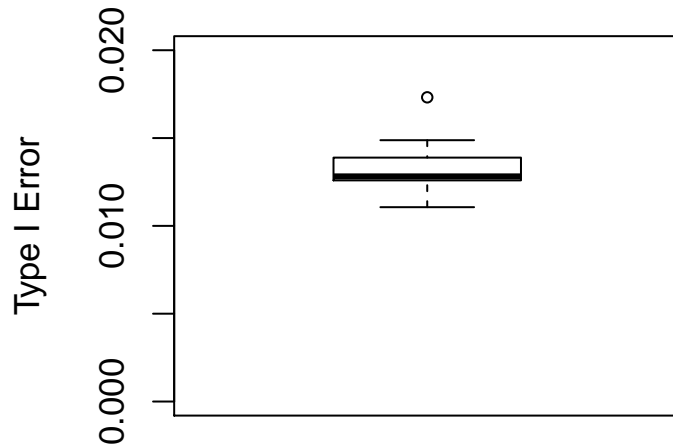

**DeconvNorm**

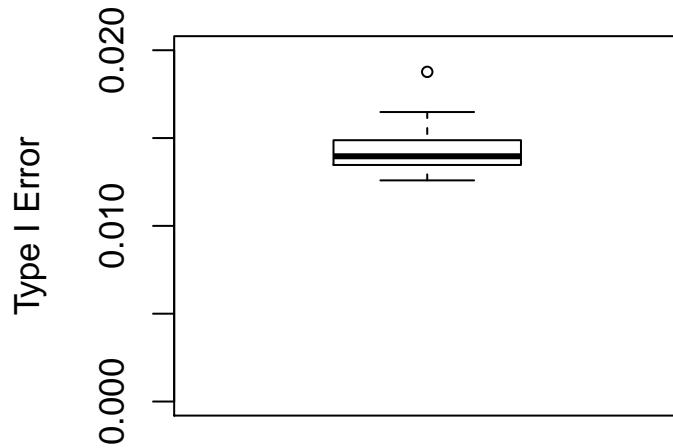

Supplement: btab226_Supplementary_Data [file btab226_supplementary_data.zip › Supplement_Revision2/TypeIError2Repl_V2.pdf]

**LogNorm**

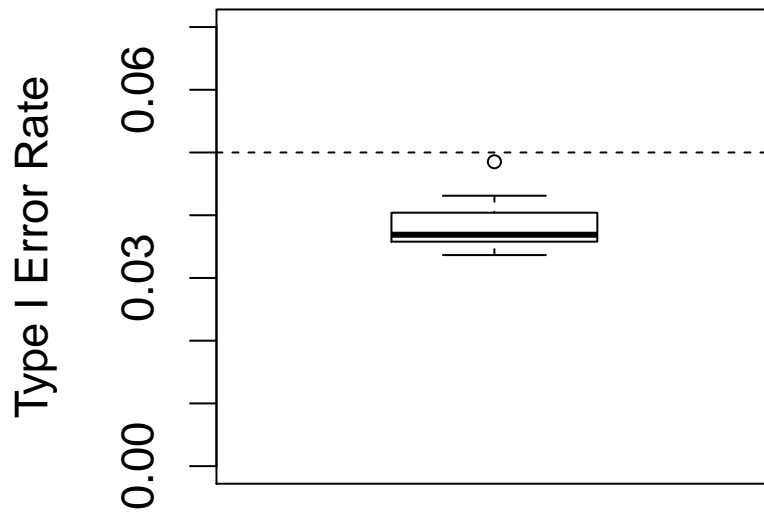

**CLRFeatNorm**

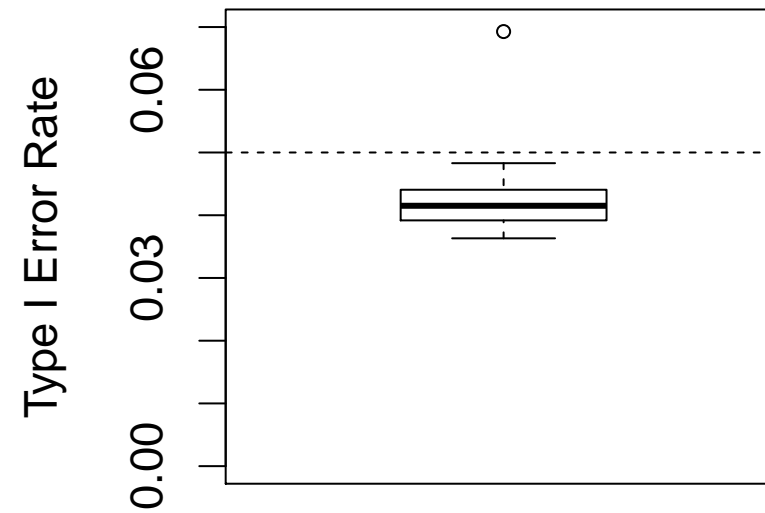

**CLRCellsNorm**

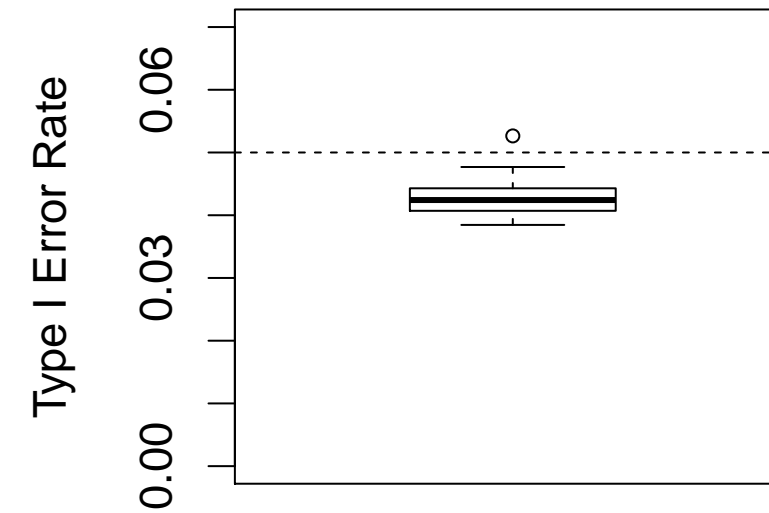

**RCNorm**

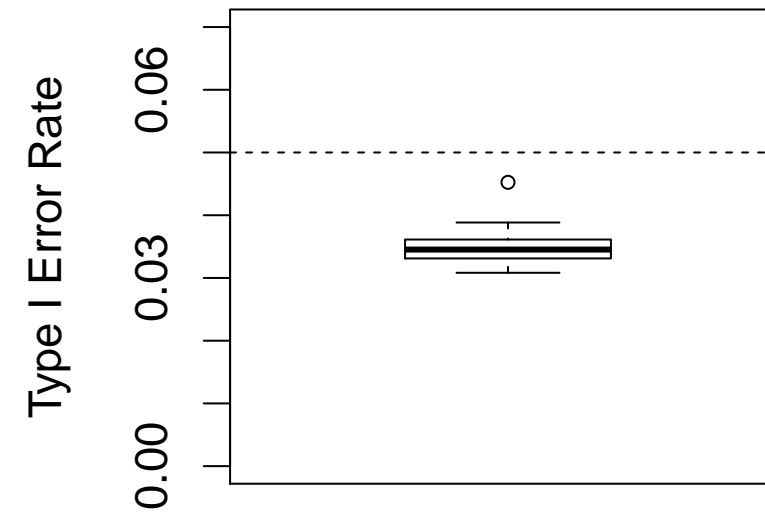

**DeconvNorm**

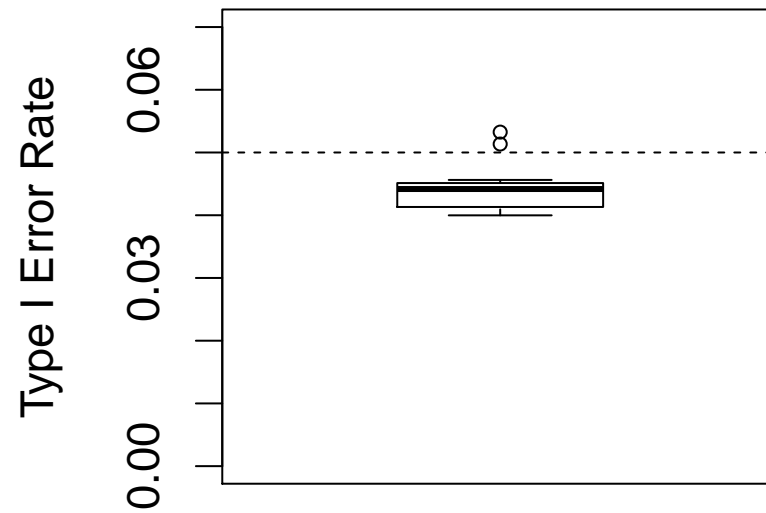

Supplement: btab226_Supplementary_Data [file btab226_supplementary_data.zip › Supplement_Revision2/TypeIError3Repl.pdf]

**waddR**

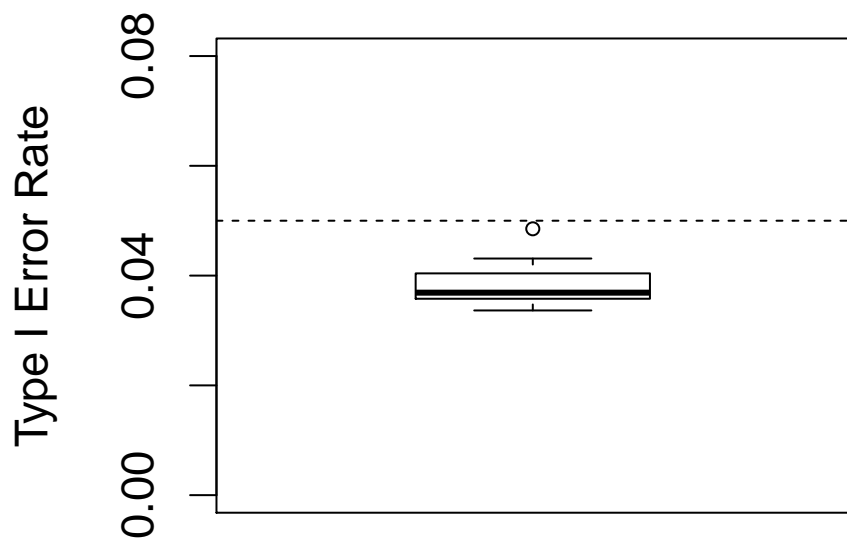

**scDD**

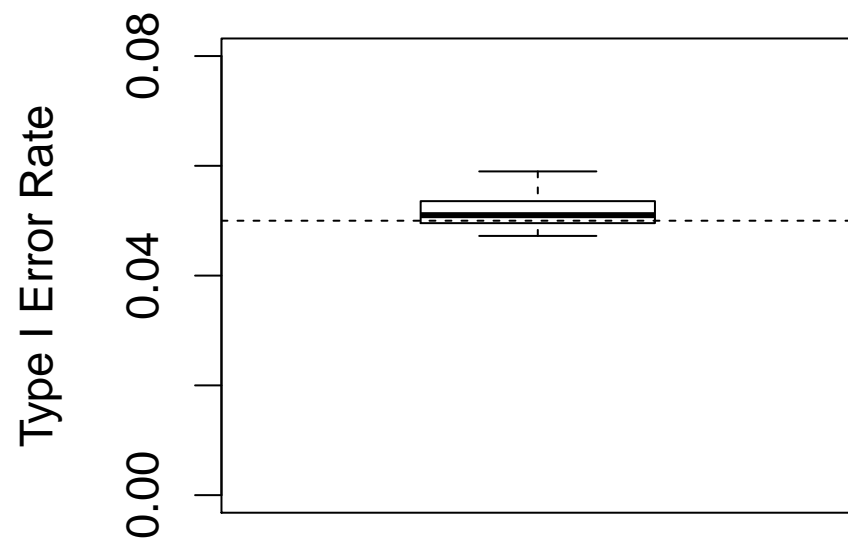

**SigEMD**

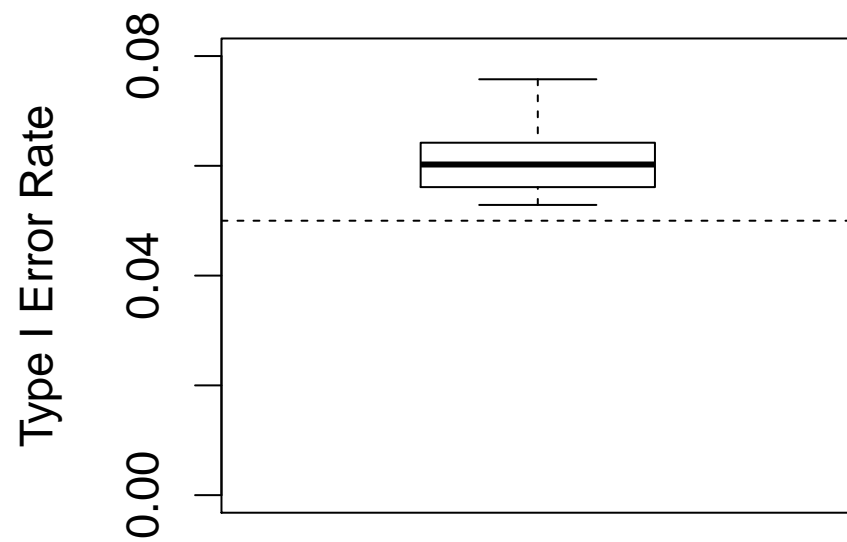

Supplement: btab226_Supplementary_Data [file btab226_supplementary_data.zip › Supplement_Revision2/TypeIError3Repl_RefMeth.pdf]

**C=100, medium DD**

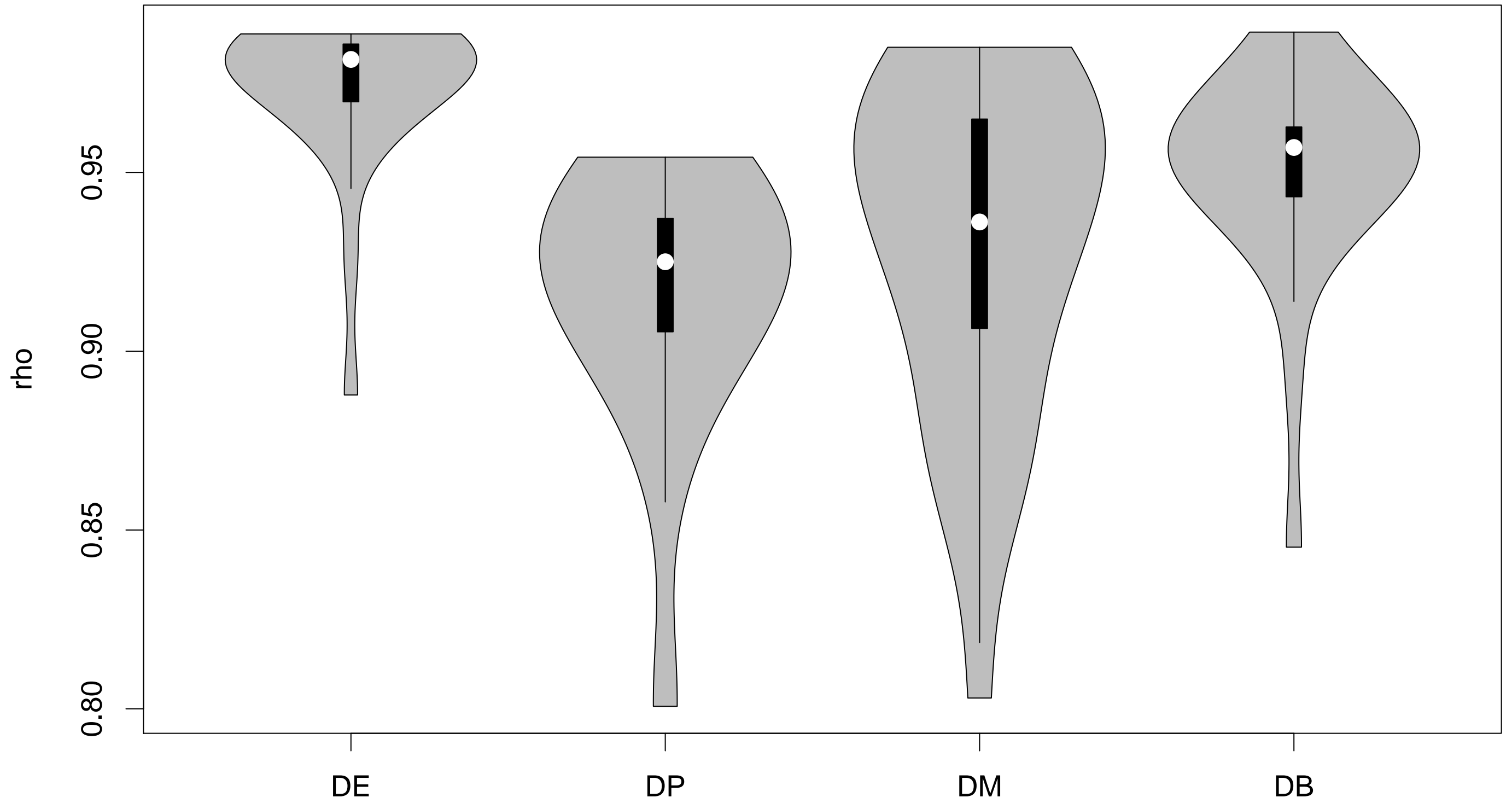

Supplement: btab226_Supplementary_Data [file btab226_supplementary_data.zip › Supplement_Revision2/Violins_cells100_mediumDD.pdf]

C=100, strong DD

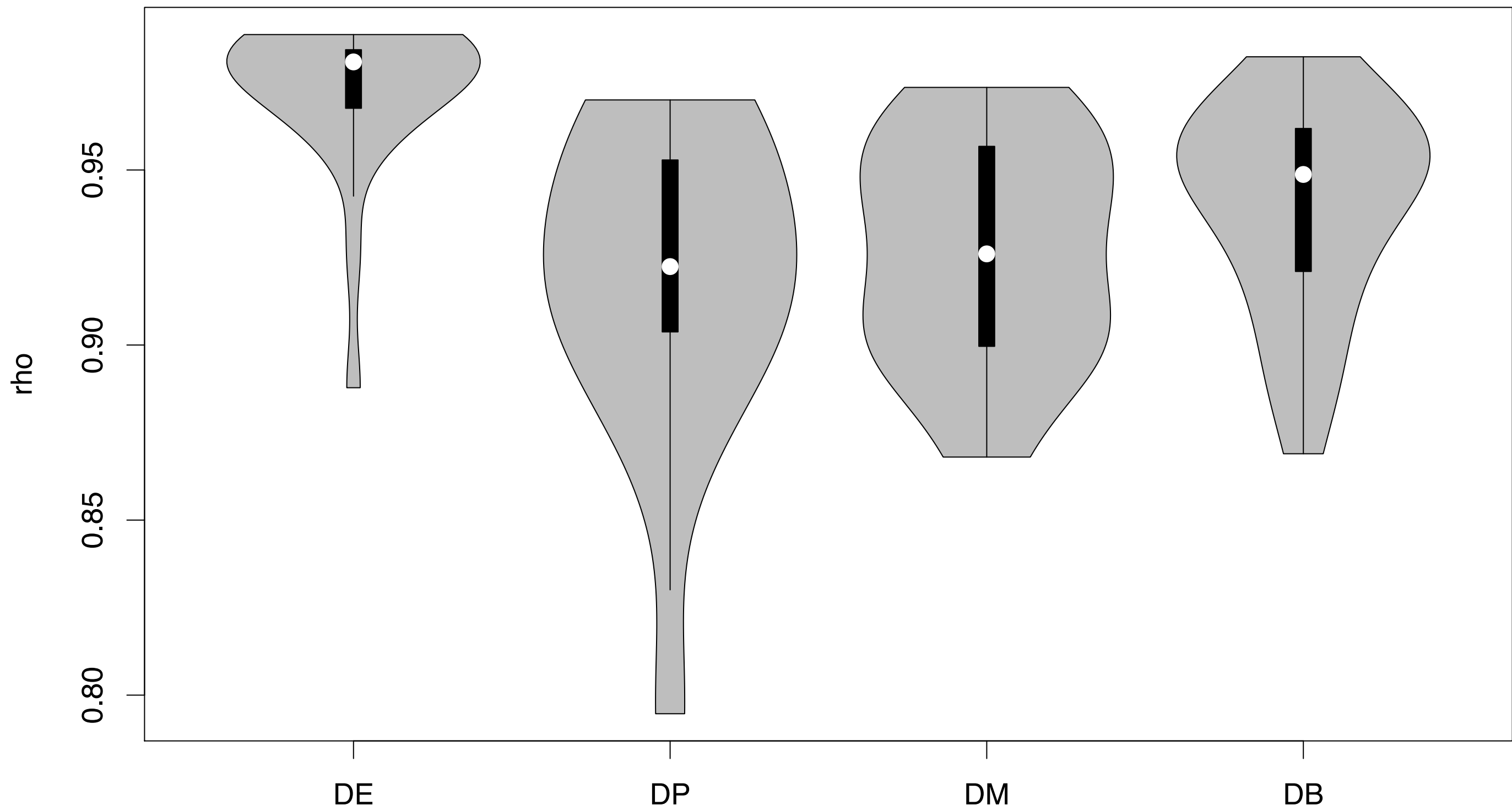

**C=500, weak DD**

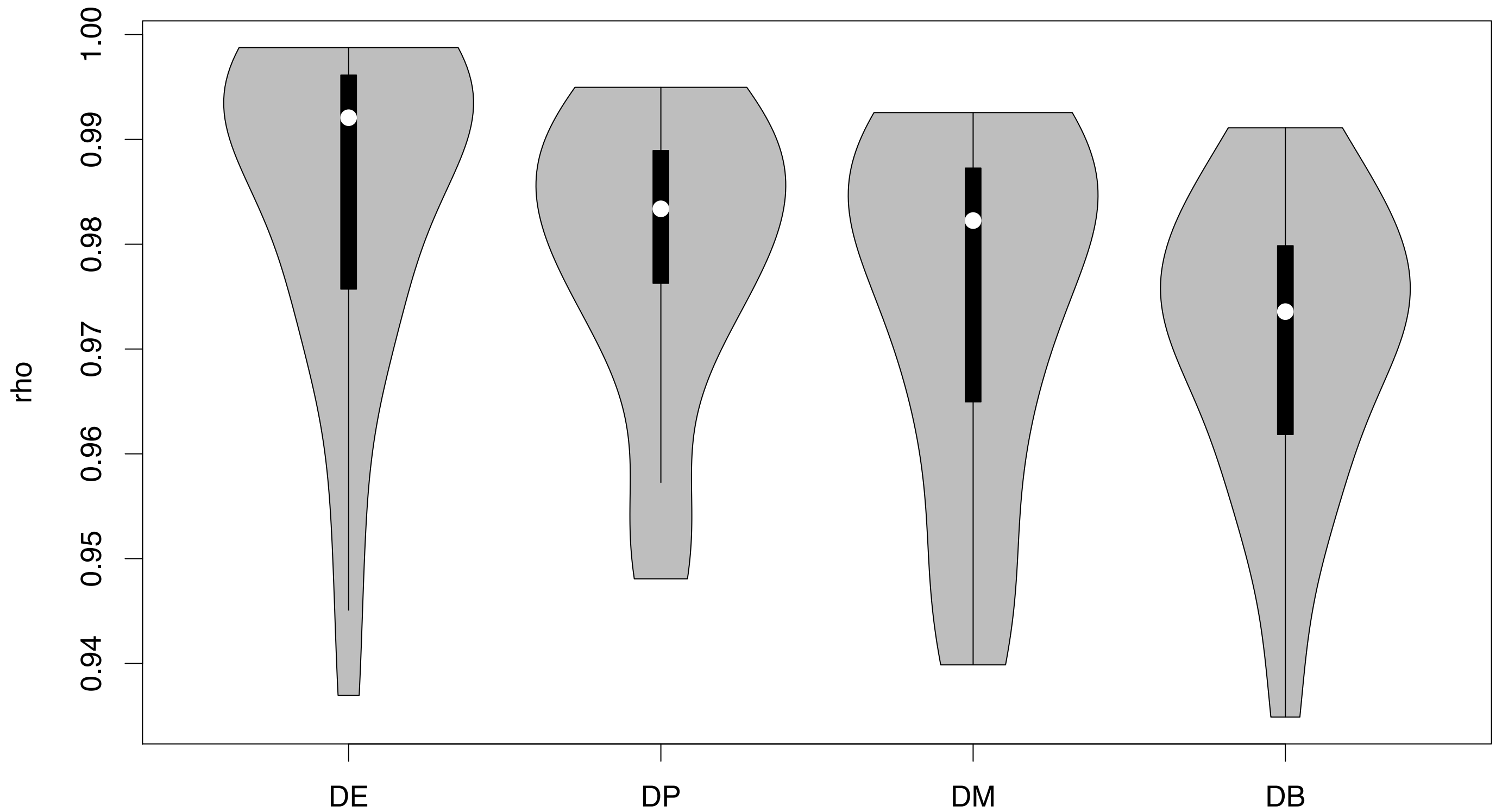

Supplement: btab226_Supplementary_Data [file btab226_supplementary_data.zip › Supplement_Revision2/Violins_cells100_strongDD.pdf]

**C=100, weak DD**

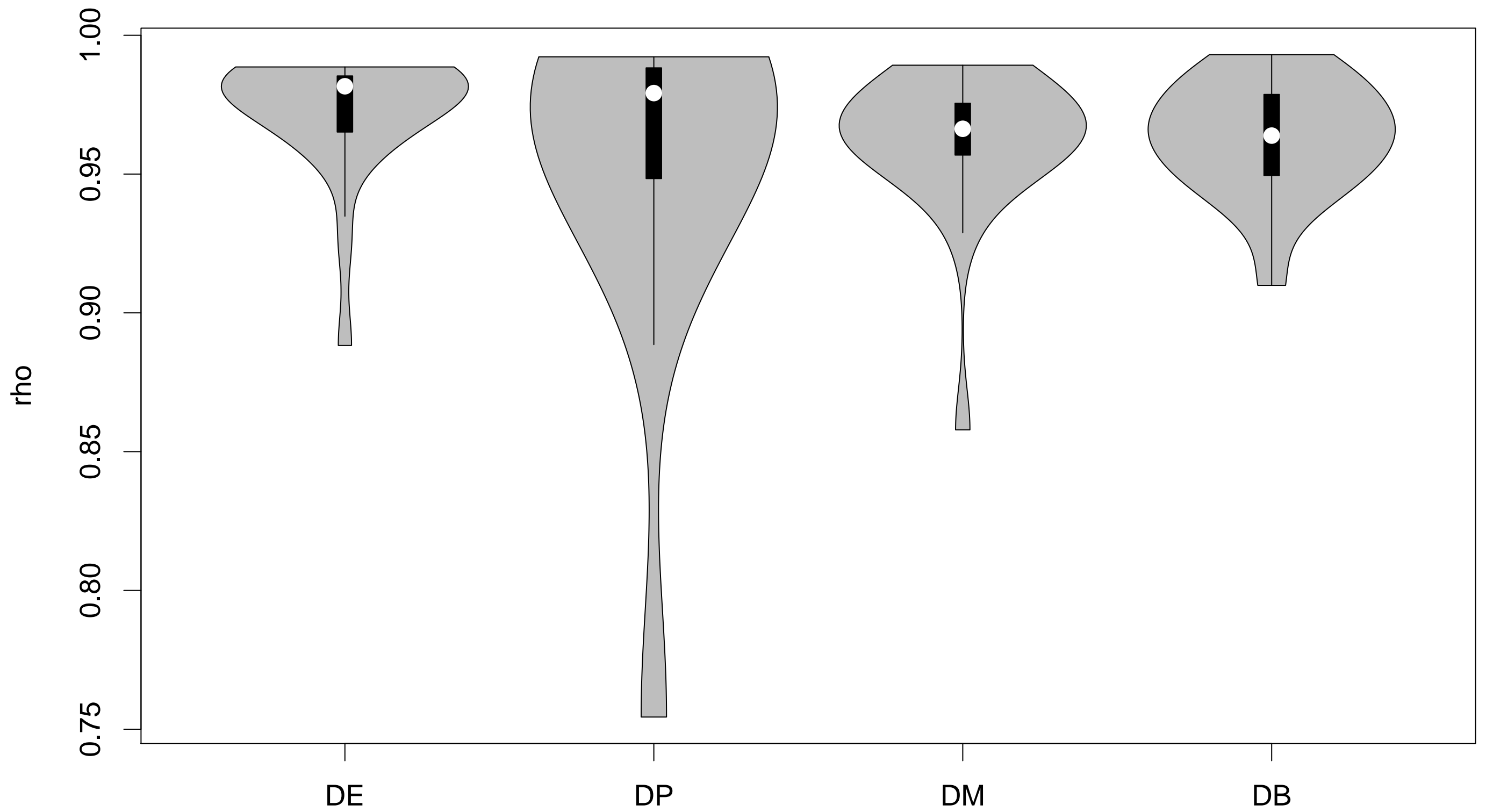

Supplement: btab226_Supplementary_Data [file btab226_supplementary_data.zip › Supplement_Revision2/Violins_cells100_weakDD.pdf]

**C=500, medium DD**

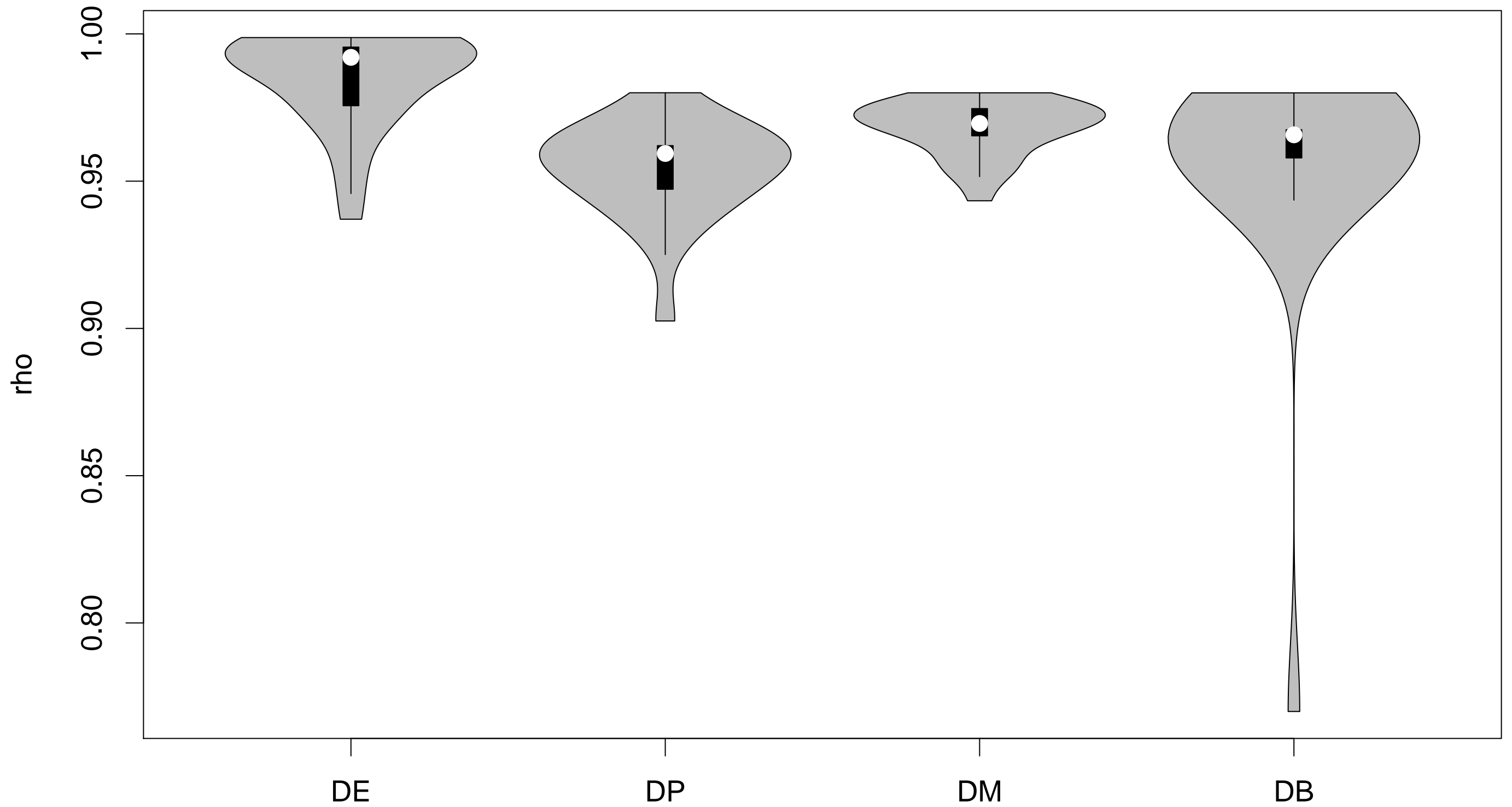

Supplement: btab226_Supplementary_Data [file btab226_supplementary_data.zip › Supplement_Revision2/Violins_cells500_mediumDD.pdf]

**C=500, weak DD**

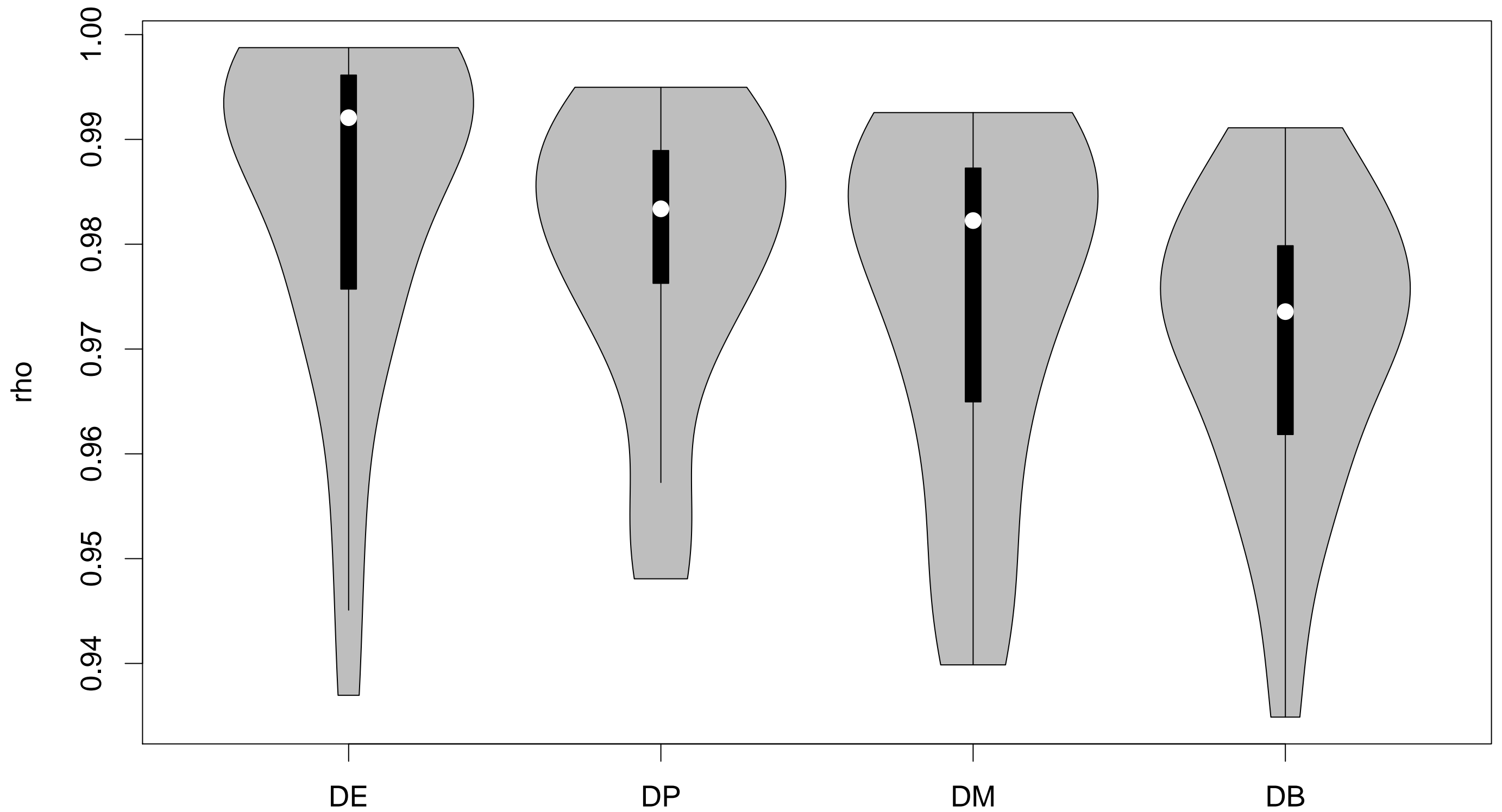

Supplement: btab226_Supplementary_Data [file btab226_supplementary_data.zip › Supplement_Revision2/Violins_cells500_weakDD.pdf]

C=50, medium DD

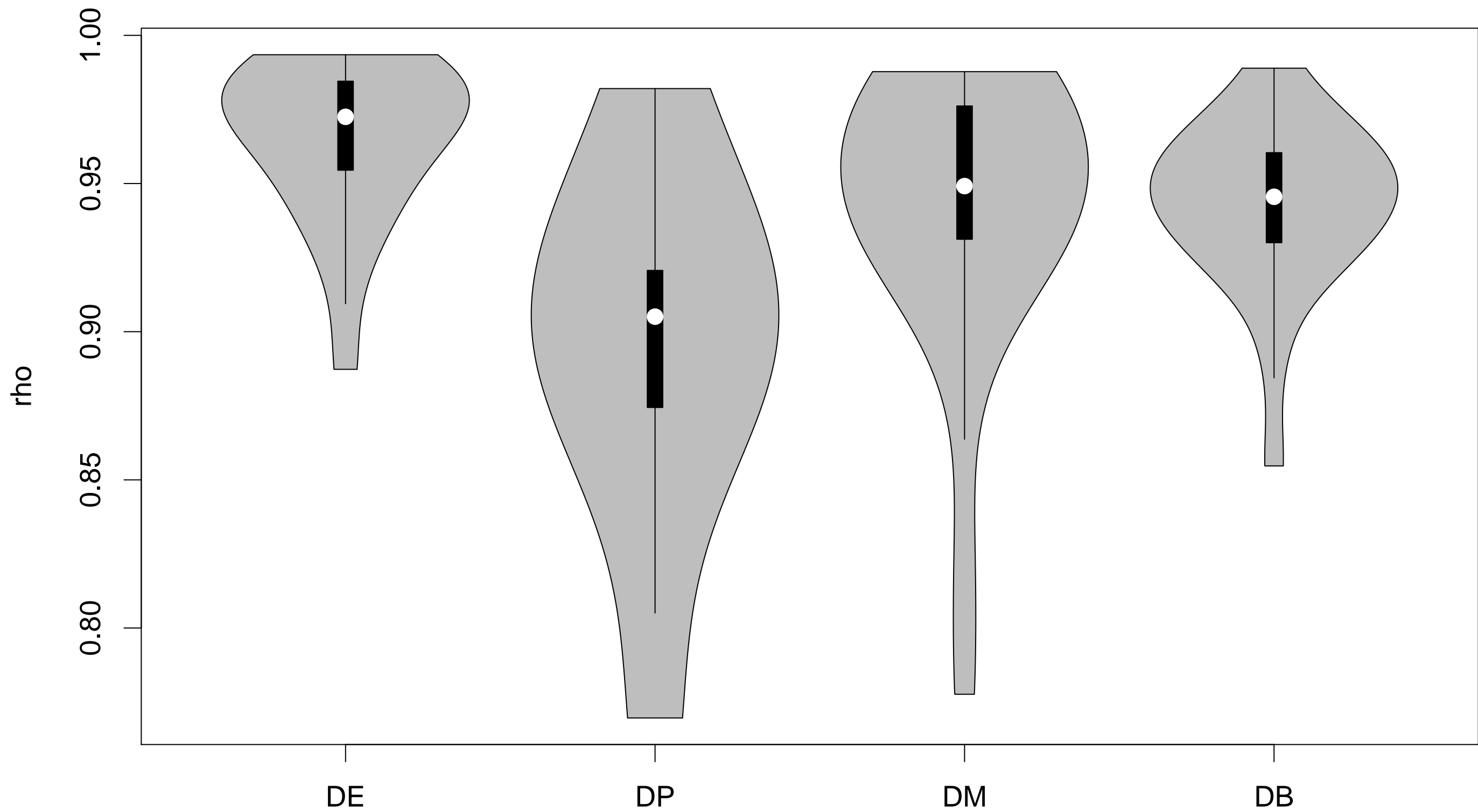

Supplement: btab226_Supplementary_Data [file btab226_supplementary_data.zip › Supplement_Revision2/Violins_cells50_mediumDD.pdf]

**C=50, strong DD**

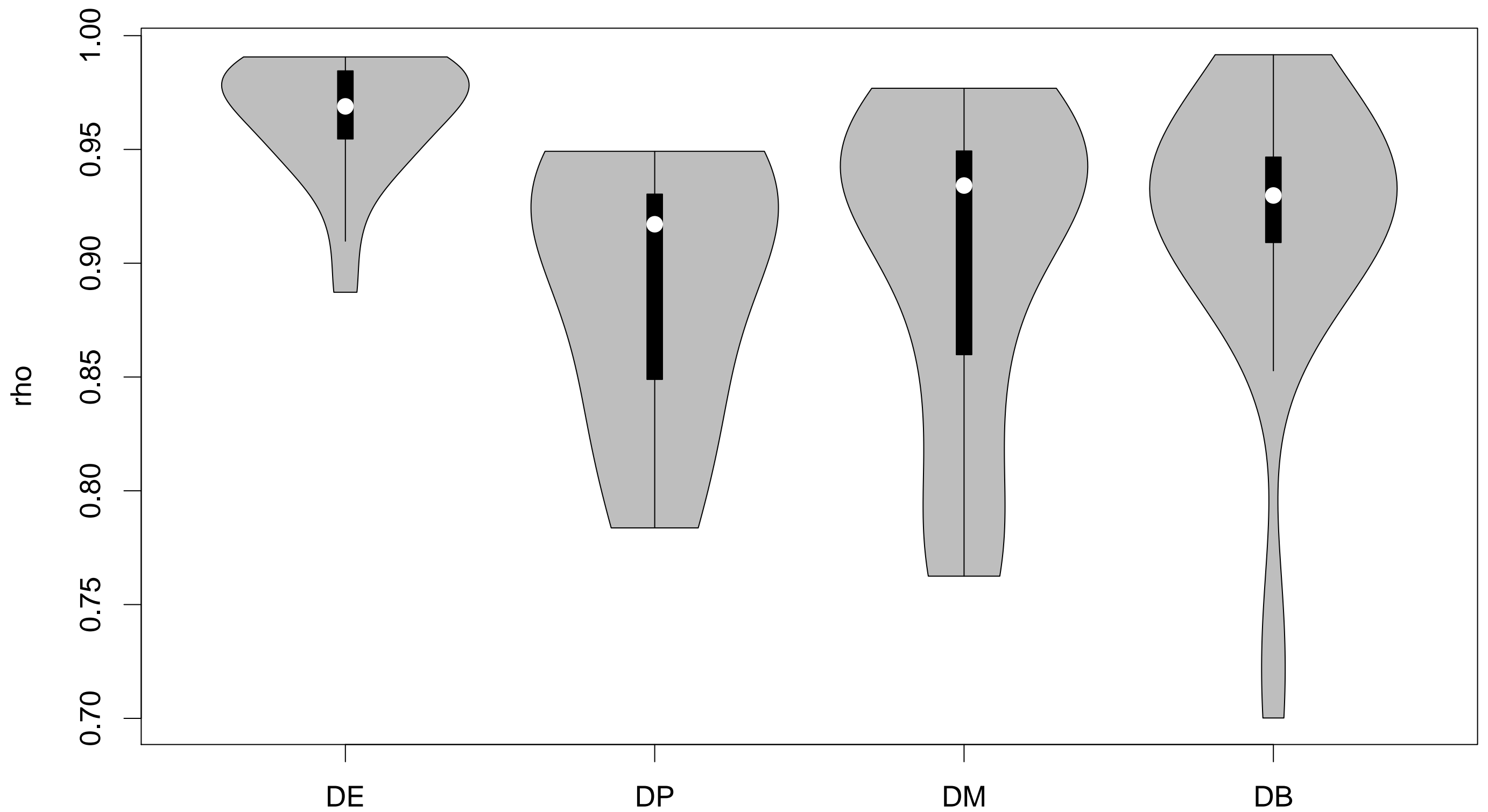

Supplement: btab226_Supplementary_Data [file btab226_supplementary_data.zip › Supplement_Revision2/Violins_cells50_strongDD.pdf]

**C=50, weak DD**

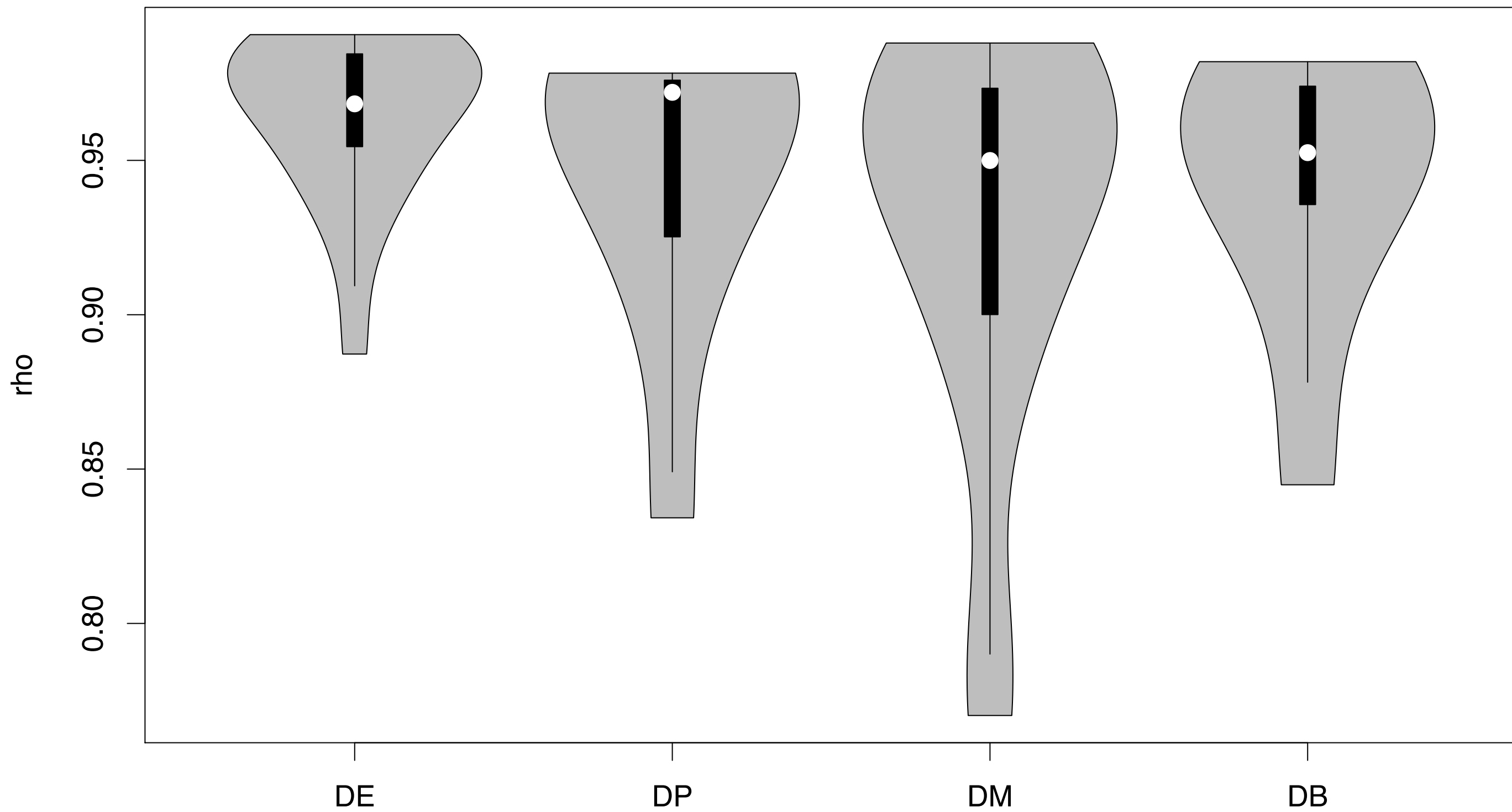

C=50, medium DD

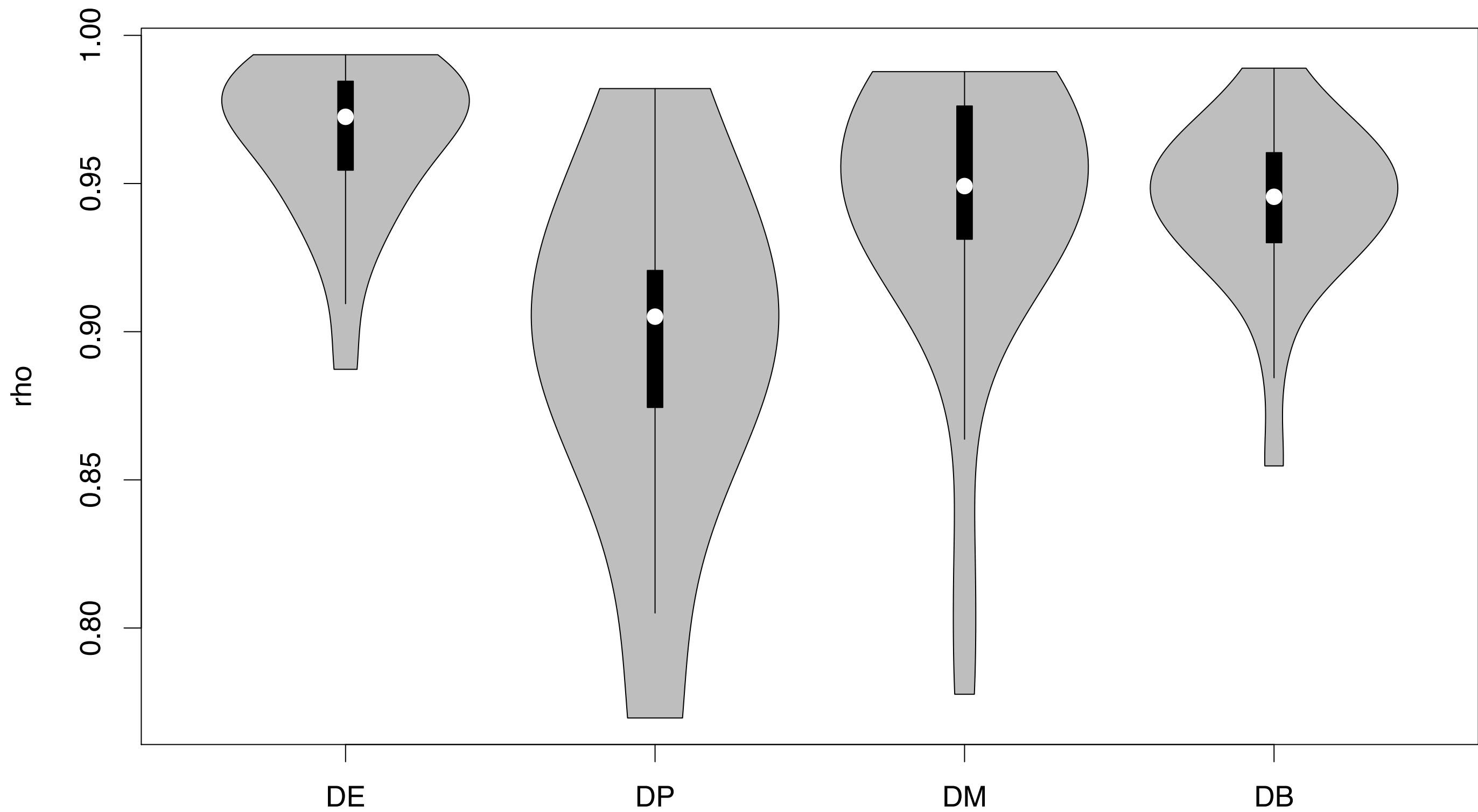

**C=50, strong DD**

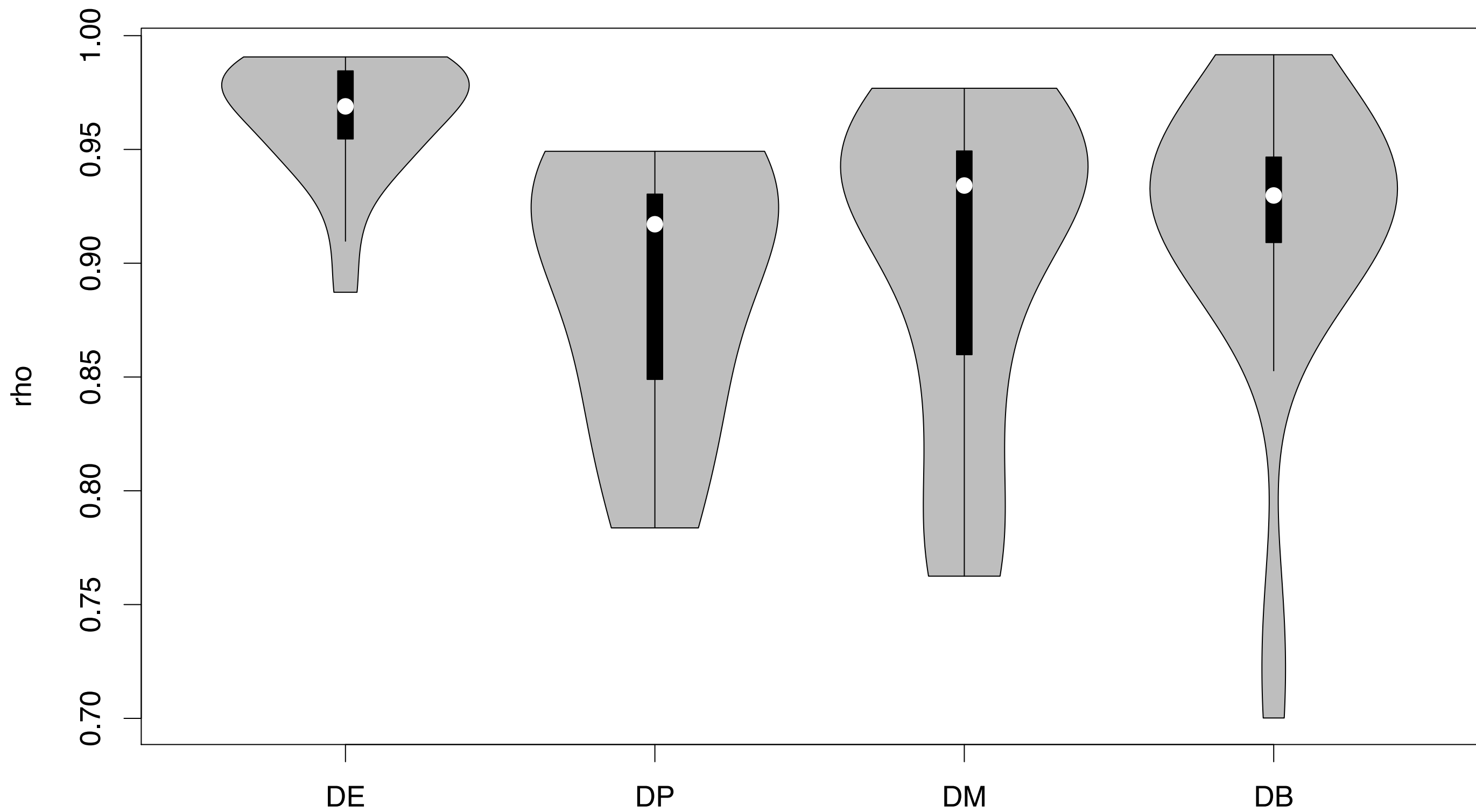

**C=100, weak DD**

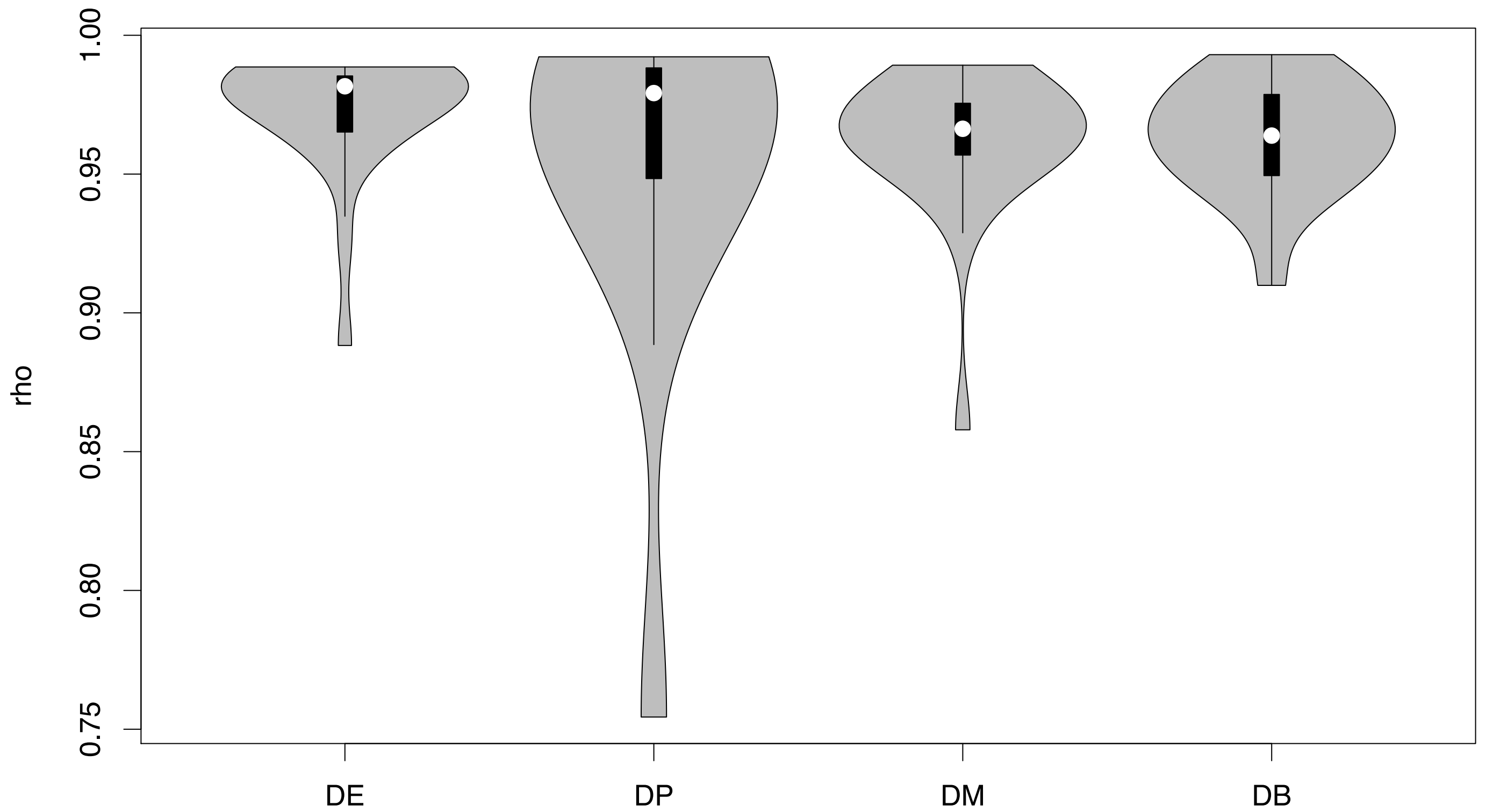

**C=100, medium DD**

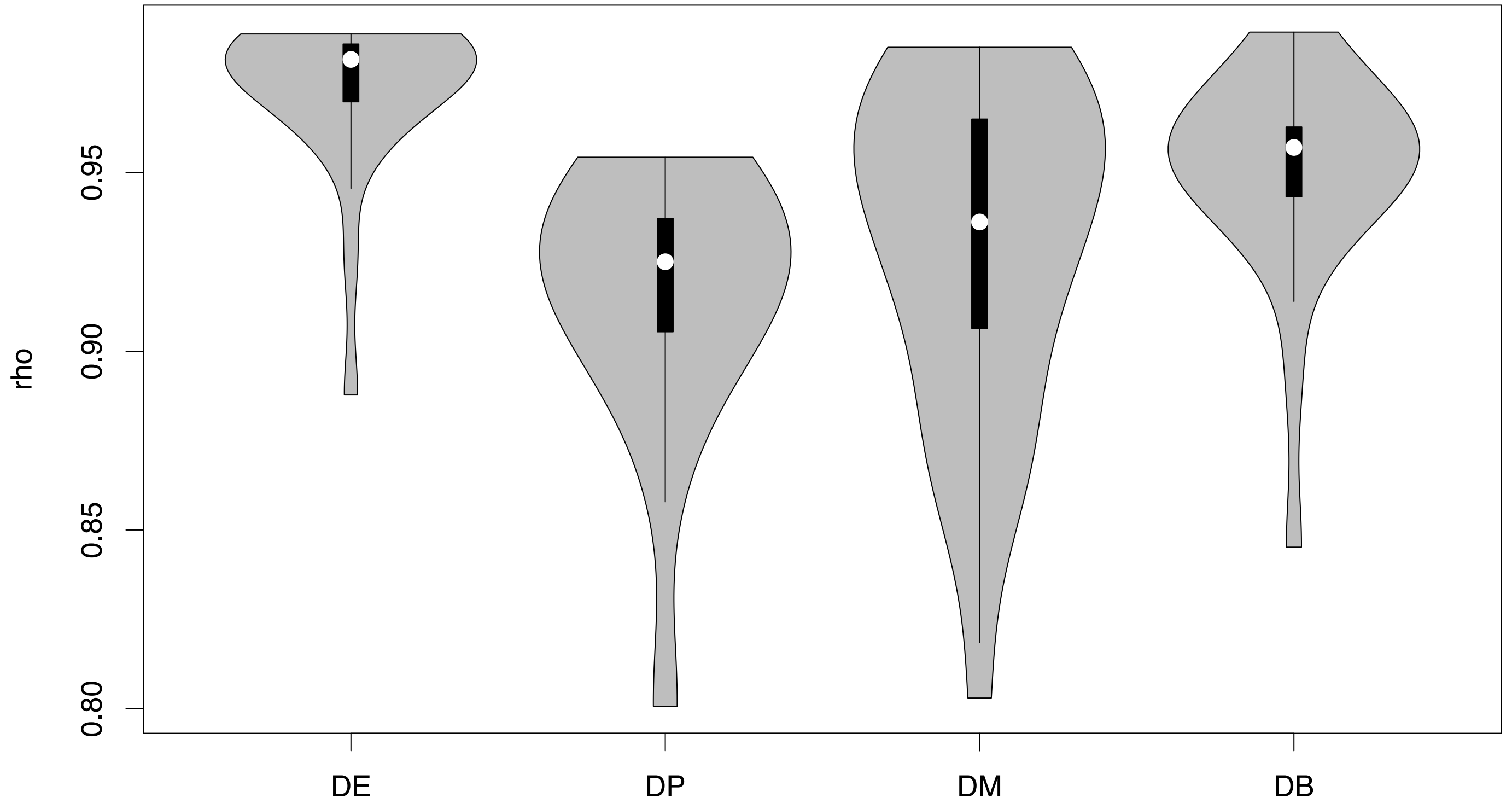

C=100, strong DD

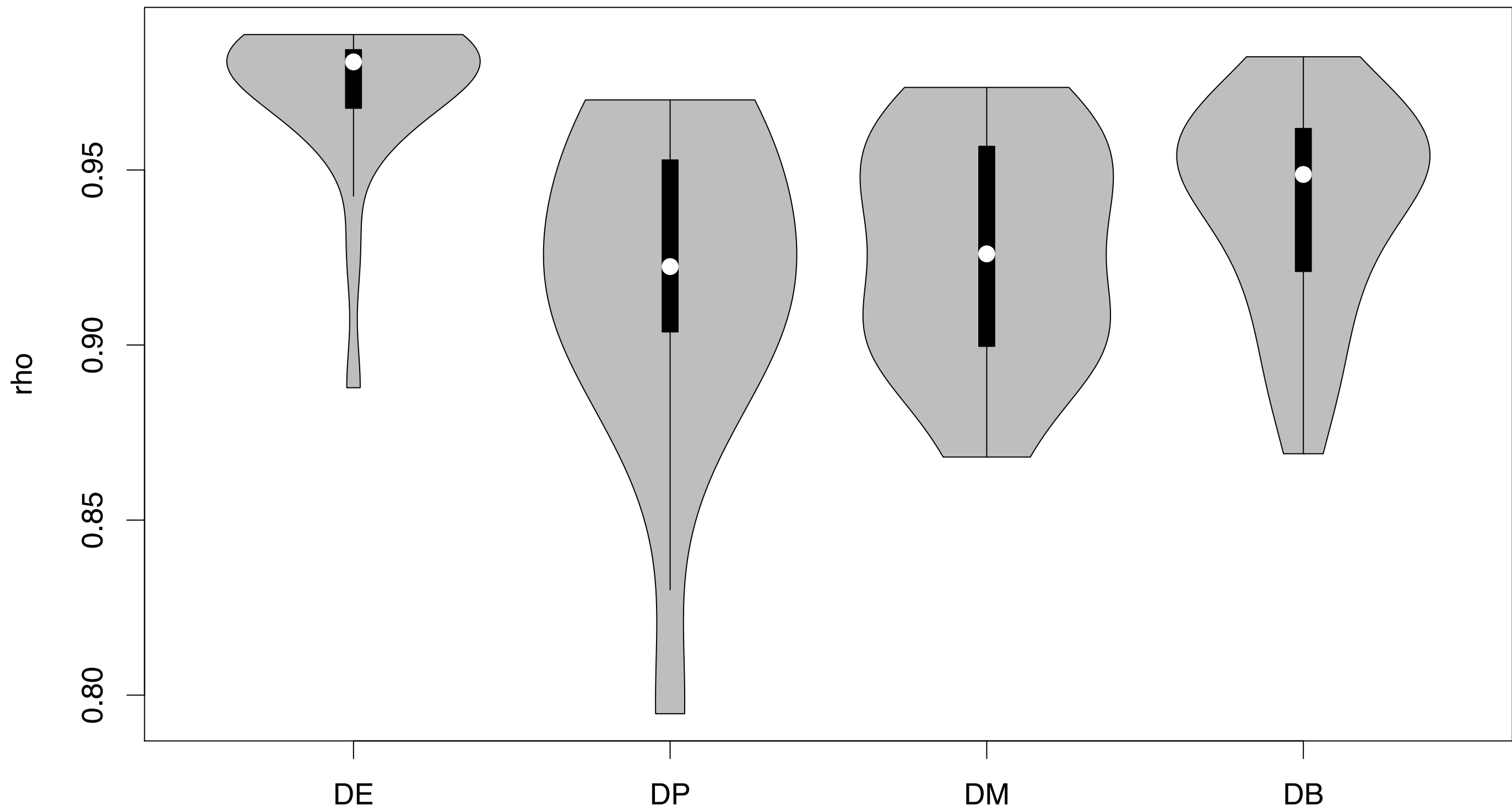

C=500, weak DD

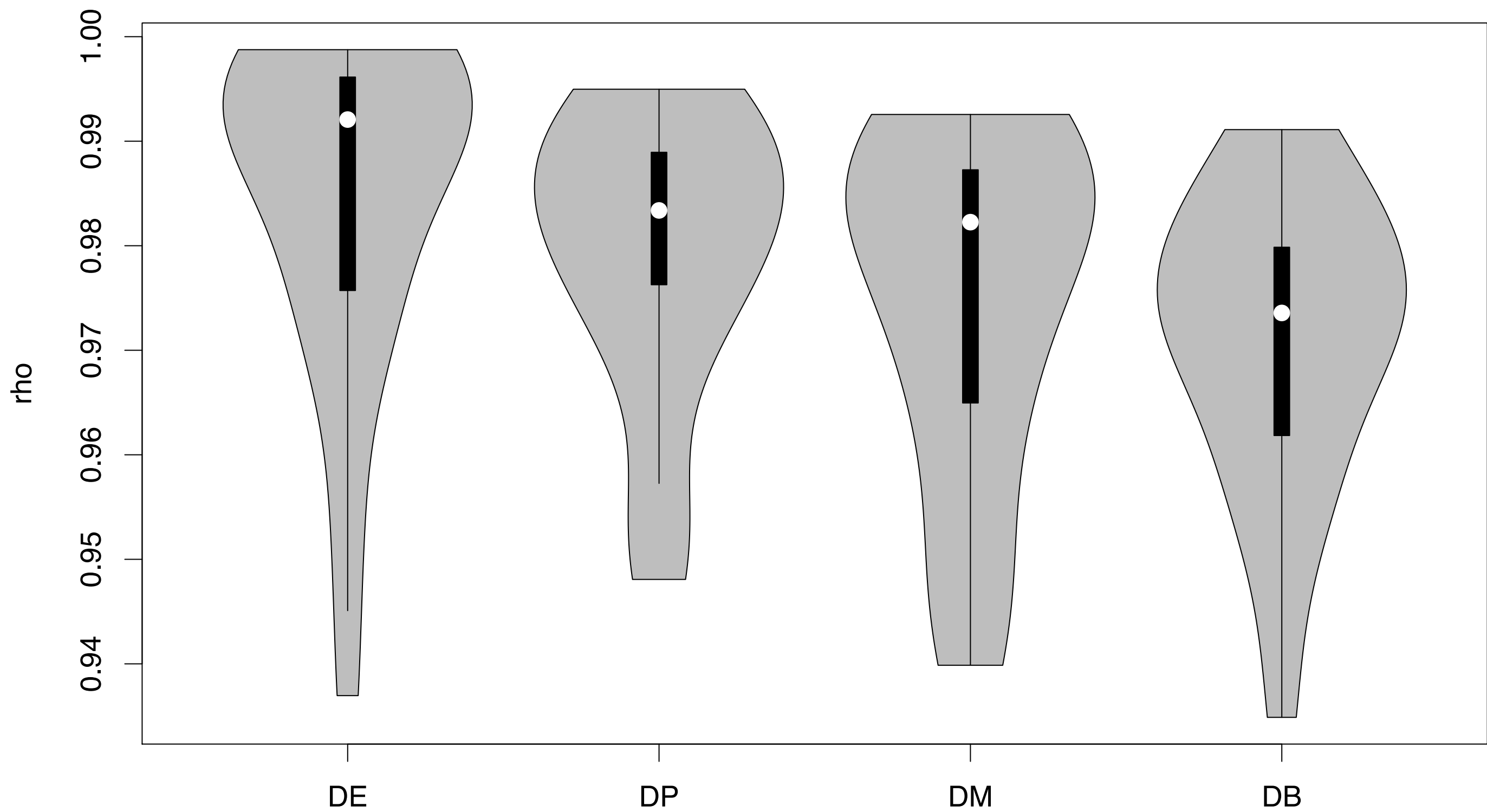

**C=500, medium DD**

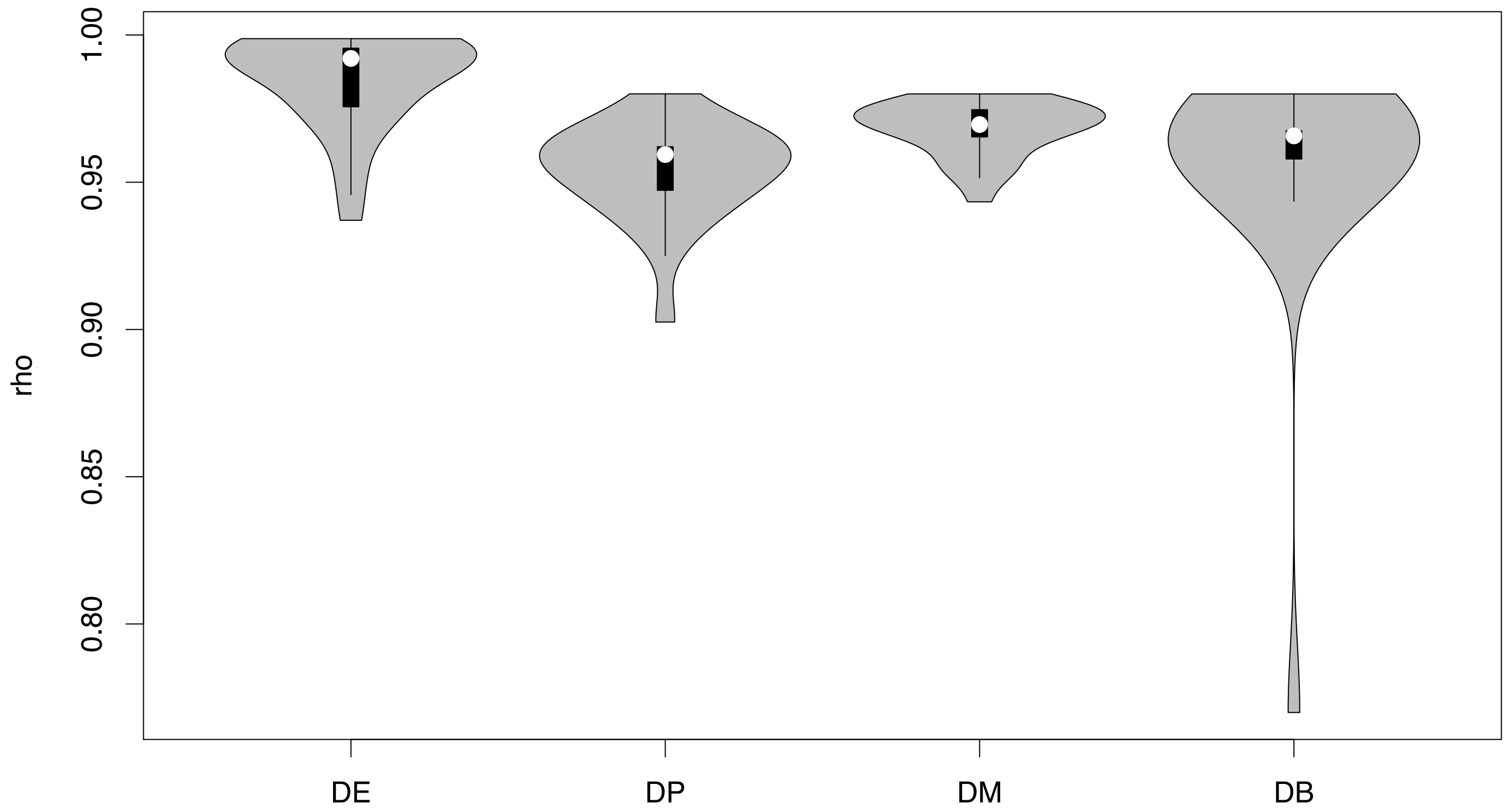

C=500, strong DD

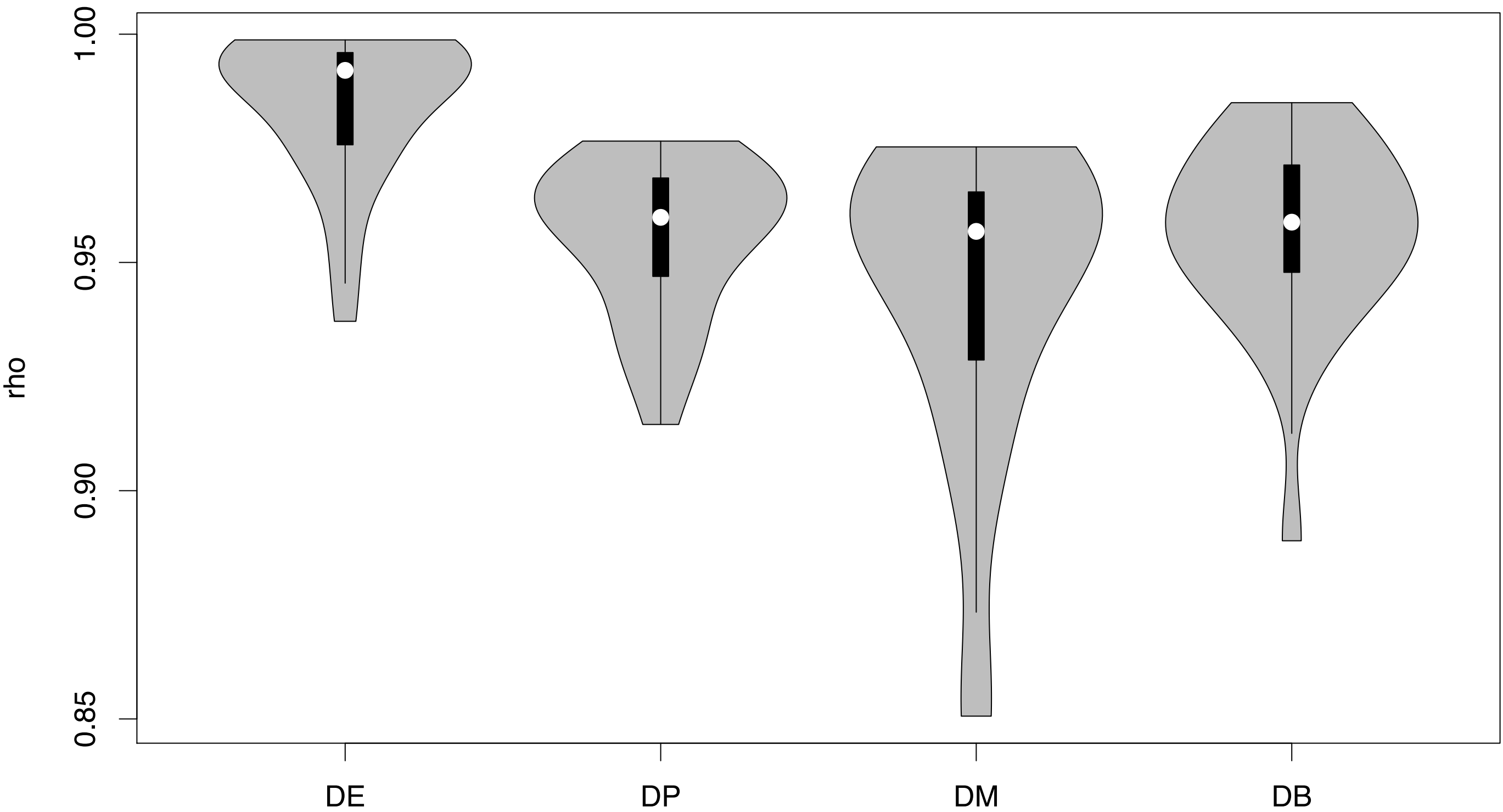

Supplement: btab226_Supplementary_Data [file btab226_supplementary_data.zip › Supplement_Revision2/Violins_cells50_weakDD.pdf]
